# Design, Synthesis and Biological Evaluation of Biphenylglyoxamide-Based Small Molecular Antimicrobial Peptide Mimics as Antibacterial Agents

**DOI:** 10.3390/ijms21186789

**Published:** 2020-09-16

**Authors:** Tsz Tin Yu, Rajesh Kuppusamy, Muhammad Yasir, Md. Musfizur Hassan, Amani Alghalayini, Satyanarayana Gadde, Evelyne Deplazes, Charles Cranfield, Mark D.P. Willcox, David StC Black, Naresh Kumar

**Affiliations:** 1 School of Chemistry, The University of New South Wales, Sydney, NSW 2052, Australia; tsztin.yu@unsw.edu.au (T.T.Y.); r.kuppusamy@unsw.edu.au (R.K.); md.m.hassan@unsw.edu.au (M.M.H.); s.gadde@unsw.edu.au (S.G.); 2 School of Optometry and Vision Science, University of New South Wales, Sydney, NSW 2052, Australia; m.yasir@unsw.edu.au (M.Y.); m.willcox@unsw.edu.au (M.D.P.W.); 3 School of Life Sciences, University of Technology Sydney, PO Box 123, Ultimo 2007, Australia; amani.alghalayini@uts.edu.au (A.A.); evelyne.deplazes@uts.edu.au (E.D.); charles.cranfield@uts.edu.au (C.C.)

**Keywords:** 5-phenylisatin, antimicrobial peptide mimics, biphenylglyoxamide, quaternary ammonium iodide, guanidinium hydrochloride, membrane disruption, antibiofilm

## Abstract

There has been an increasing interest in the development of antimicrobial peptides (AMPs) and their synthetic mimics as a novel class of antibiotics to overcome the rapid emergence of antibiotic resistance. Recently, phenylglyoxamide-based small molecular AMP mimics have been identified as potential leads to treat bacterial infections. In this study, a new series of biphenylglyoxamide-based small molecular AMP mimics were synthesised from the ring-opening reaction of *N*-sulfonylisatin bearing a biphenyl backbone with a diamine, followed by the conversion into tertiary ammonium chloride, quaternary ammonium iodide and guanidinium hydrochloride salts. Structure–activity relationship studies of the analogues identified the octanesulfonyl group as being essential for both Gram-positive and Gram-negative antibacterial activity, while the biphenyl backbone was important for Gram-negative antibacterial activity. The most potent analogue was identified to be chloro-substituted quaternary ammonium iodide salt **15c**, which possesses antibacterial activity against both Gram-positive (MIC against *Staphylococcus aureus* = 8 μM) and Gram-negative bacteria (MIC against *Escherichia coli* = 16 μM, *Pseudomonas aeruginosa* = 63 μM) and disrupted 35% of pre-established *S. aureus* biofilms at 32 μM. Cytoplasmic membrane permeability and tethered bilayer lipid membranes (tBLMs) studies suggested that **15c** acts as a bacterial membrane disruptor. In addition, in vitro toxicity studies showed that the potent compounds are non-toxic against human cells at therapeutic dosages.

## 1. Introduction

Rapid emergence of antibiotic-resistant bacteria is a major global health concern. The Gram-positive *Staphylococcus aureus* often has a high antibiotic-resistant rate, with approximately 65–85% of nosocomial *S. aureus* infections associated with a beta-lactam-resistant strain [1,2]. When taking other bacterial pathogens into account, there are more than 2.8 million individuals seriously infected by bacteria in the United States every year, and 700,000 individuals are killed by drug-resistant bacteria around the world annually [3,4]. Since the late 1980s, there have been no new classes of antibiotics entering the market, with new clinical drugs merely being the structural derivatives of existing antibiotics with the same scaffolds [5,6,7]. These derivatives are prone to rapid resistance development and so can only retard but not overcome the bacterial resistance dilemma. Hence, there is an urgent need to develop novel classes of antibiotics to treat bacterial infection.

Another challenge encountered in battling bacterial infections is bacterial biofilms. The resistance of bacteria in biofilms can be up to 1000 times higher than their planktonic from [8,9]. A biofilm is a complex multicellular exopolysaccharide matrix that acts to protect the bacteria against harmful conditions and also sequestrates a nutrient-rich area [8,9,10]. This exopolysaccharide matrix also significantly reduces the penetrance of antibiotics to the bacteria embedded in the biofilm [11,12,13]. Furthermore, the presence of different phenotypes of bacteria in a biofilm creates a heterogeneity in the growth rate and metabolism of the bacteria [14]. As conventional antibiotics mainly target metabolically active and growing cells, slow or non-growing bacteria that survive the antibiotic treatment can then reproduce and pass their resistance genes to their offspring. Moreover, the increased rate of horizontal gene transfer in biofilms compared to planktonic cells can accelerate the speed of resistance spread [8,15]. Approximately 65–80% bacterial infections are associated with biofilm formations, and one of the most common pathogens found in biofilms is *S. aureus* [14,16,17]. To combat biofilms, a few antibiofilm mechanisms have been developed in recent years such as to disrupt or degrade the membrane potential of bacterial cells embedded in biofilms [18].

In recent years, there has been increasing interest in the development of antimicrobial peptides (AMPs) as a new class of antibiotics. AMPs are naturally occurring peptides that serve as the first line of the innate immune defence system in humans. They exhibit a broad spectrum of antimicrobial properties against different microorganisms including bacteria, viruses and fungi [19,20,21,22]. The mechanism of action of AMPs is mainly attributed to their facially amphiphilic structure. It is thought that the cationic residues on one face of the AMP first bind electrostatically with the anionic bacterial membrane surface. Then, the hydrophobic residues on the opposite face of the AMP aid in the insertion of the entire AMP molecule by associating with the lipophilic interior of the bacterial cell membrane. This disrupts the bacterial membrane, leading to the loss of membrane potential and leakage of cellular contents, eventually killing the bacterial cell [19,23,24,25]. Unlike conventional antibiotics, AMPs act via non-receptor interactions. Since a complete restructuring of the cell membrane is required for the development of resistance, the chances of bacteria developing drug resistance to AMPs is low [19,26,27,28]. While AMPs possess high potency against bacterial cells, their deployment as drugs has been impeded by their poor bioavailability, low proteolytic stability, high manufacturing cost and poor yield from multi-step syntheses [19,29,30].

**Figure 1:**
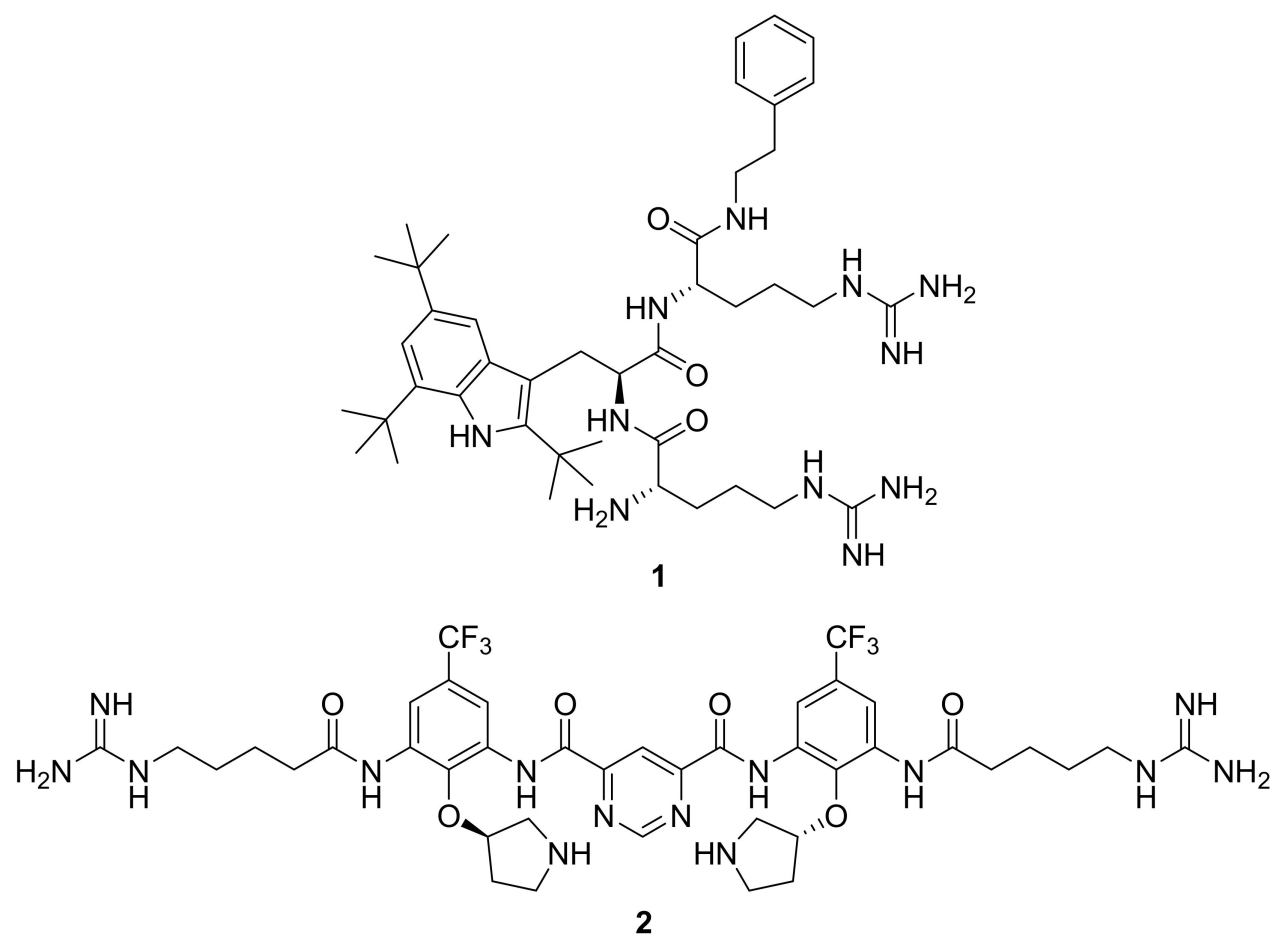
Structures of Lytixar (LTX-109) **1** and Brilacidin (PMX-30063) **2**.

The limitations of AMPs have stimulated the development of AMP mimics such as α-peptides [31], β-peptides [32,33] and peptoids [34,35]. In addition, there have been reports of several small molecular AMP mimics such as anthranilamides [36], cationic peptoids [35], cholic acid derivatives [37] and phenyleneethynylenes [38]. Similar to natural AMPs, these AMP mimics possess an amphiphilic structure with good spatial separation between the hydrophobic and cationic groups. Among these AMP mimics, Lytixar (LTX-109) **1** and Brilacidin (PMX-30063) **2** (Figure 1) have completed phase II human clinical trials, suggesting that AMP mimics could be potential therapeutic agents for treating bacterial infections [39].

**Figure 2:**
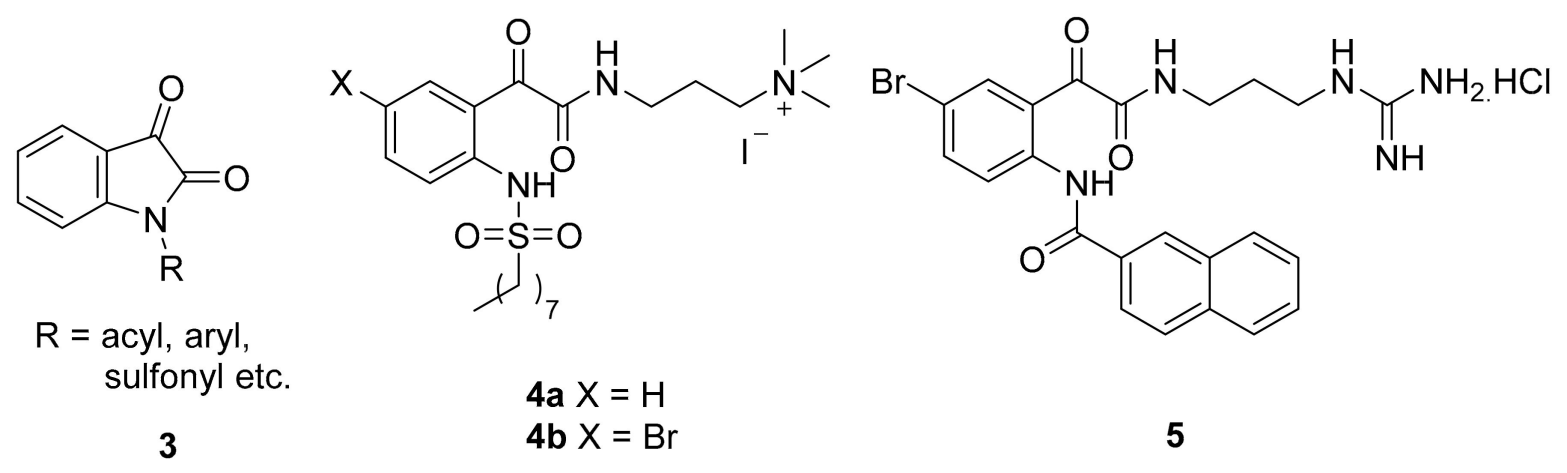
Structures of *N*-substituted isatin **3** and phenylglyoxamide derivatives **4–5**.

Isatin (indoline-2,3-dione) is a natural product found in plants of the genus *Isatis* [40]. Its derivatives have been reported to show a wide range of biological and pharmacological properties, such as being antimicrobial, anti-inflammatory, anticancer, antiviral and acting as analgesics [41]. Interestingly, *N*-acyl, *N*-aryl and *N*-sulfonylisatins **3** (Figure 2) can act as electrophiles and be ring-opened by amines and alcohols to afford the corresponding phenylglyoxamides and glyoxylic esters, respectively [40,42]. Since ring-opened phenylglyoxamide derivatives possess an amide bond, they are potential candidates for the development of AMP mimics. Our group has previously reported the synthesis of phenylglyoxamides (e.g., **4**–**5**) derived from *N*-acylisatins and *N*-sulfonylisatins [43,44,45]. Among these molecules, *N*-sulfonylphenylglyoxamide iodide salt **4b** and *N*-naphthoylphenylglyoxamide guanidinium salt **5** had moderate to good minimum inhibitory concentrations (MIC) of 63 and 12 μM respectively, against Gram-positive *S. aureus* [43,44]. However, these compounds gave no antibacterial activity against Gram-negative bacteria. Hence, we were interested to structurally modify these compounds as part of the optimisation process.

Biphenyl is an important scaffold in drug development and is found in many drug molecules and natural products [46,47]. Compounds containing the biphenyl moieties possess a wide variety of biological properties, including being antibacterial and antifungal [48]. However, the low solubility of biphenyl compounds in both water and common organic solvents is a major drawback which impedes their synthesis and development for pharmaceutical applications. To address this issue, Ol’khovik et al. incorporated the quaternary ammonium group in the development of antimicrobial biphenyl molecules [49].

Recently, our group has demonstrated the importance of the biphenyl moiety in the development of AMP mimics [50]. As the synthesis of biphenylglyoxamide derivatives has not been explored, we report for the first time the synthesis of novel biphenylglyoxamide-based antimicrobial peptide mimics from 5-phenylisatins. The antibacterial and antibiofilm activities of these compounds were evaluated against *S. aureus*, *Pseudomonas aeruginosa* and *Escherichia coli*. The mechanism of action and in vitro cytotoxicity of these compounds were also explored.

## 2. Results and Discussion

### 2.1. Design and Synthesis of Biphenyl Glyoxamide-Based Antimicrobial Peptide Mimics

In this work, a phenyl ring was installed at the 5-position of the phenylglyoxamide scaffold, giving the biphenylglyoxamide scaffold. The terminal phenyl ring of this scaffold was modified with electron-withdrawing halogen substituent (F and Cl) or a bulky naphthalenyl substituent to investigate their effect on antibacterial activity. Four series of compounds with different cationic or hydrophilic groups, namely glyoxamide derivatives (Series I), tertiary ammonium hydrochloride salts (Series II), quaternary ammonium iodide salts (Series III) and guanidinium hydrochloride salts (Series IV), were synthesised to compare the effect of cationic functionality on the biological activity of the AMP mimics. Moreover, the *N*-octanesulfonyl group was appended to these AMP mimics as the hydrophobic group, and this hydrophobic group was modified to the *N*-butanesulfonyl group or *N-*naphthoyl group to study their effect on antibacterial activity.

**Scheme 1:**
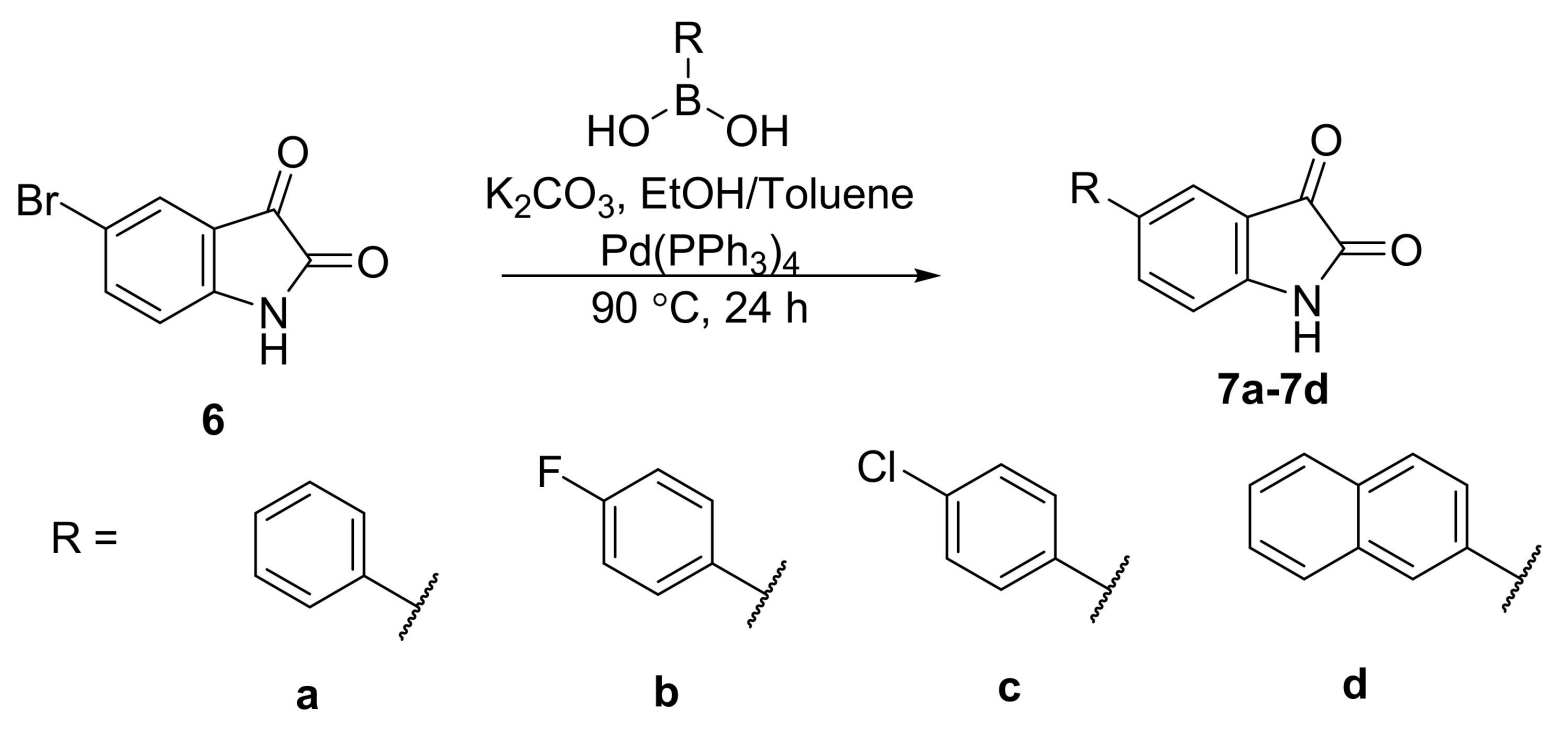
Synthesis of 5-arylisatins **7a–7d**.

**Scheme 2:**
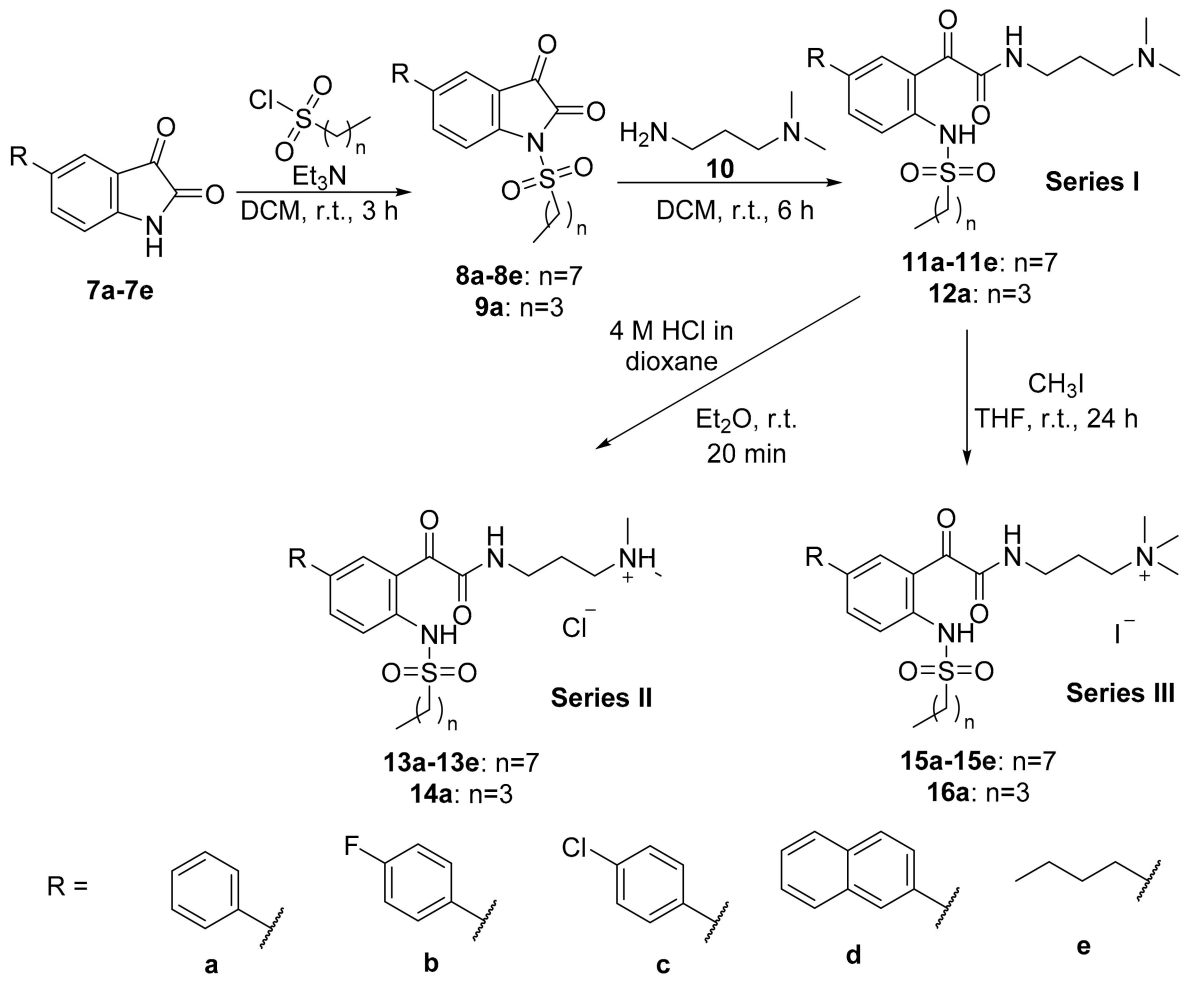
General synthetic scheme for the synthesis of series I glyoxamide derivatives **11–12**, series II tertiary ammonium chloride salts **13–14** and series III quaternary ammonium iodide salts **15–16**.

**Scheme 3:**
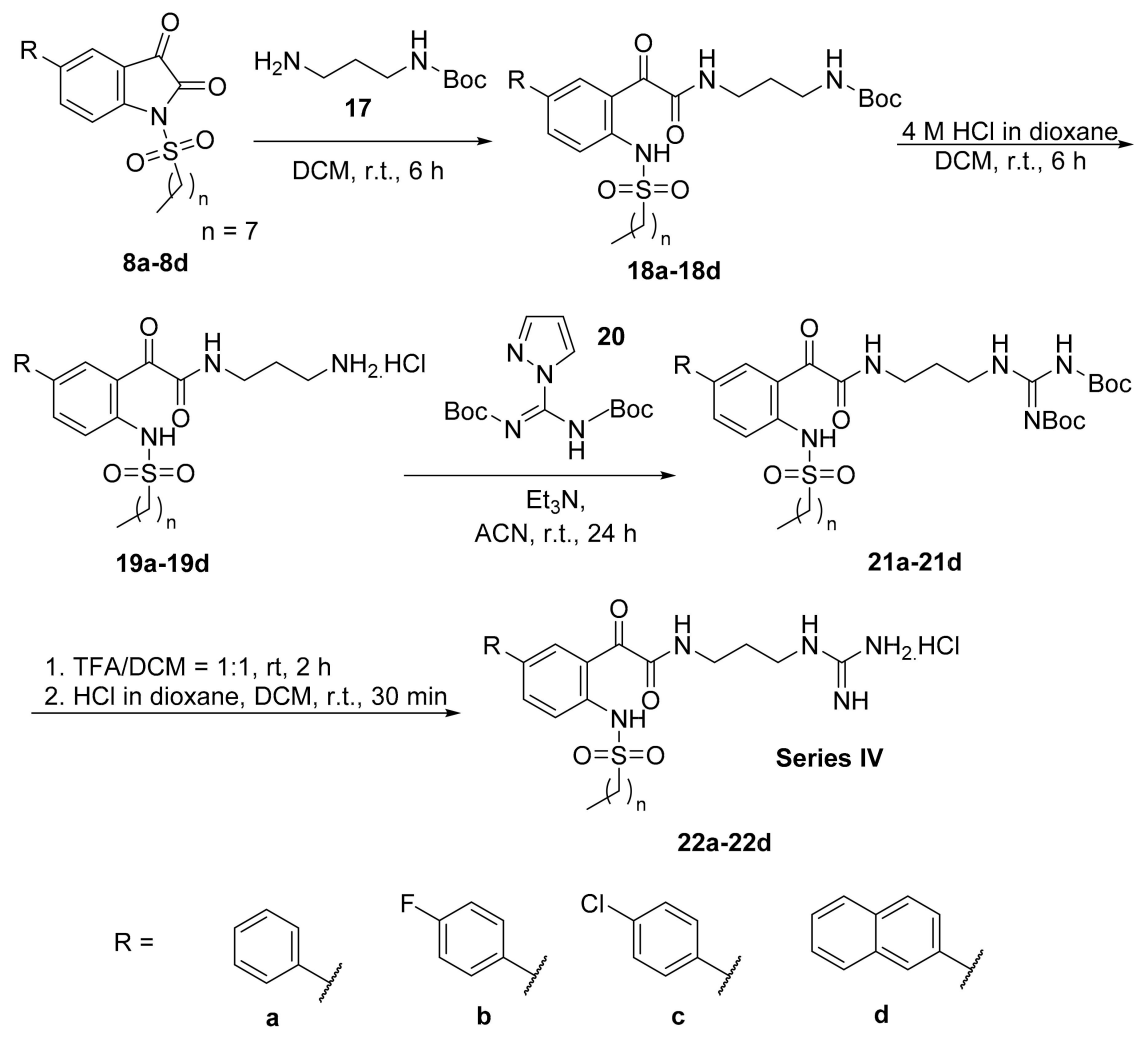
General synthetic scheme for the synthesis of series IV guanidinium hydrochloride salts **22**.

**Scheme 4:**
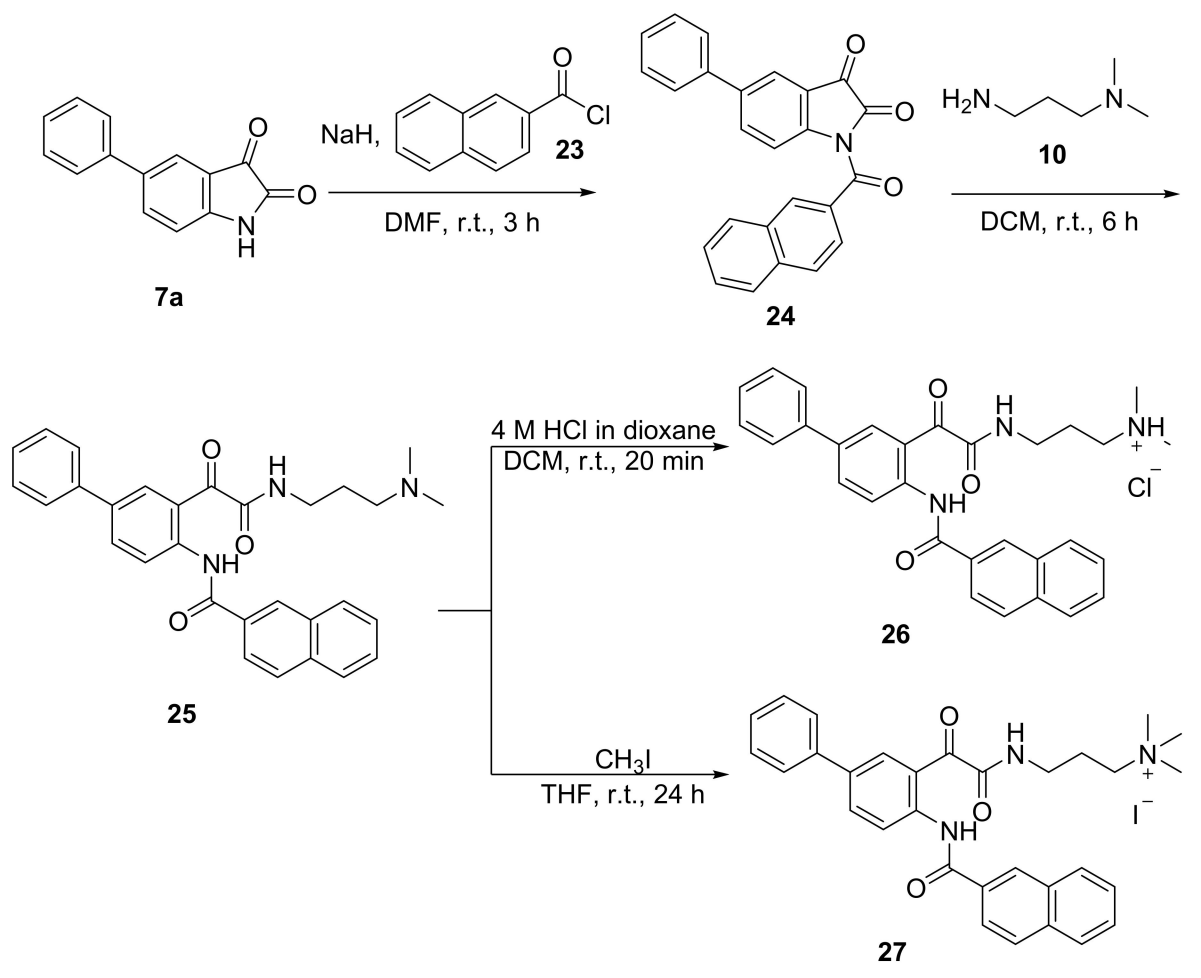
Synthesis of *N*-naphthoylglyoxamide derivative **25** and its corresponding tertiary ammonium chloride salt **26** and quaternary ammonium iodide salt **27**.

These biphenylglyoxamide-based antimicrobial peptide mimics were synthesised according to the pathways described in Scheme 1, Scheme 2, Scheme 3 and Scheme 4.

The biphenyl scaffold was incorporated into the molecules by the Suzuki-Miyaura cross-coupling reaction. This was achieved by reacting 5-bromisatin **6** with different commercially available arylboronic acids to afford 5-arylisatins **7** in good yields (57–86%) (Scheme 1).

A hydrophobic group was introduced into the scaffold by reacting 5-arylisatins **7a**–**7d** with an alkylsulfonyl chloride and triethylamine as base, furnishing *N*-alkylsulfonyl compounds **8a**–**8d** and **9a** in good yields (52–72%) (Scheme 2). Subsequent nucleophilic ring-opening reaction of *N*-alkylsulfonyl compounds **8a**–**8d** and **9a** with 3-dimethylaminopropylamine **10** afforded the corresponding glyoxamides **11a**–**11d** and **12a** as series I compounds in excellent yields (94–99%). The cationic group was installed by treating glyoxamides **11a**–**11d** and **12a** with 4 M HCl in dioxane to give tertiary ammonium chloride salts **13a**–**13d** and **14a** as series II compounds in 92–99% yields or with methyl iodide in tetrahydrofuran (THF) to give quaternary ammonium iodide salts **15a**–**15d** and **16a** as series III compounds in 90–95% yields. The analogous reactions with 5-butylisatin **7e** as the starting material generated the corresponding tertiary ammonium chloride salt **13e** and quaternary ammonium iodide salt **15e** in 99% and 85% yields, respectively.

Alternatively, *N*-octanesulfonyl compounds **8a**–**8d** were also ring-opened with *N*-Boc-1,3-propanediamine **17** to give Boc-protected glyoxamides **18a–18d** in excellent yields (95–97%) (Scheme 3). Boc-protected glyoxamides **18a**–**18d** were treated with 4 M HCl in dioxane to yield aminoglyoxamides **19a**–**19d** in good yields (64–90%). The subsequent guanylation reaction using *N*,*N’*-di-Boc-1*H*-pyrazole-1-carboxamide and triethylamine afforded the corresponding Boc-protected guanidine glyoxamides **21a**–**21d** in moderate to good yields (31–77%). Finally, treating the Boc-protected guanidine glyoxamides **21a**–**21d** with trifluoroacetic acid in dichloromethane (DCM) followed by 4 M HCl in dioxane provided the guanidinium hydrochloride salts **22a**–**22d** as series IV compounds in 50–77% yields.

Biphenyl derivatives **25**–**27** bearing a naphthoyl hydrophobic group in place of the alkylsulfonyl group were also synthesised. Naphthoylation was achieved by treating 5-phenylisatin **7a** with sodium hydride and 2-naphthoyl chloride **23** to give the 5-phenyl-*N*-naphthoylisatin **24** in 35% yield (Scheme 4). The nucleophilic ring-opening reaction of 5-phenyl-*N*-naphthoylisatin **24** with 3-dimethylaminopropylamine **10** gave amine **25**, which was followed by salt conversion into the corresponding tertiary ammonium chloride salt **26** and quaternary ammonium iodide salt **27** in 84% and 79% yields, respectively.

### 2.2. Structure–Activity Relationship Study

The antibacterial activities of the synthesised antimicrobial peptide mimics were evaluated by determining their minimum inhibitory concentration (MIC) against the Gram-positive *S. aureus* (SA38). Moreover, quaternary ammonium iodide salts **15a**–**15e** and guanidinium hydrochloride salts **22a**–**22d** were also tested against Gram-negative *P. aeruginosa* (PA01) and *E. coli* (K12) (Table 1). Generally, the tested compounds showed lower antibacterial activity against Gram-negative strains compared to Gram-positive *S. aureus*.

In the structure–activity relationship (SAR) analysis, the biphenyl system was beneficial for the antibacterial activity of the analogues. Against Gram-positive *S. aureus*, the previously synthesised unsubstituted parent and 5-bromosubstituted quaternary ammonium iodide salt **4** had an MIC value of 250 and 63 μM respectively [43]. When a phenyl ring was substituted at the 5-position to give the corresponding biphenyl analogue **15a**, the MIC value decreased to 16 μM, indicating that this analogue was nearly sixteen and four times as potent as the unsubstituted and 5-bromosubstituted compound, respectively. Interestingly, having an *n*-butyl group at the 5-position of the phenyl ring, as in analogue **15e**, also gave strong activity against *S. aureus* (MIC = 16 μM). Moreover, the biphenyl analogue **15a** showed MIC values of 125 and 32 μM against the Gram-negative *P. aeruginosa* and *E. coli* respectively, while the unsubstituted parent compound **4a**, 5-bromosubstituted **4b** and 5-butylsubstituted **15e** analogues showed no antibacterial activity against these strains even at the highest concentration tested (250 μM). This suggested that the biphenyl moiety is essential for the antibacterial activity against Gram-negative bacteria, as the antibacterial ability was lost once the phenyl ring was removed.

After the biphenyl moiety was identified to be essential for antibacterial activity, modifications were made to the terminal phenyl ring to investigate the effect of incorporating an electron-withdrawing halogen atom at the *para*-position of the terminal phenyl ring as well as replacing the terminal phenyl ring with a bulky naphthalene ring. Neither modification had a significant influence on the antibacterial activity of the analogues against Gram-positive *S. aureus*, as all cationic analogues (**13a**–**13d, 15a**–**15d, 22a**–**22d**) showed MIC values of 8 or 16 μM. Against the Gram-negative *P. aeruginosa* and *E. coli*, the introduction of an electron-withdrawing halogen atom had no significant influence or only slightly increased the antibacterial activity of the analogues. However, when the terminal phenyl ring was replaced by a bulky naphthalene ring, the antibacterial activity of the analogue against Gram-negative bacteria was significantly reduced (Table 1). Specifically, the activity of the naphthalenyl-substituted quaternary ammonium iodide salt **15d** was halved compared to **15a**, while the activity of the corresponding guanidinium hydrochloride salt **22d** was completely lost. This suggested that the steric hinderance arising from the bulky naphthalene group may reduce the activity of the analogues against Gram-negative bacteria.

The effect of modifying the terminal group of the glyoxamide chain was also studied. In general, Gram-positive antibacterial activity was weakest for the non-charged glyoxamide compounds **11a**–**11e**. Among the cationic compounds, the guanidinium hydrochloride salts **22a**–**22d** were slightly more potent against Gram-positive bacteria compared to the corresponding tertiary ammonium chloride salts **13a**–**13d** and quaternary ammonium iodide salts **15a**–**15d**. In this study, only quaternary ammonium iodide salts **15a**–**15e** and guanidinium hydrochloride salts **22a**–**22d** were tested against Gram-negative *P. aeruginosa* and *E. coli* owing to their lower cytotoxicity, while the corresponding glyoxamide derivatives **11a**–**11e** and tertiary ammonium chloride salts **13a**–**13e** were not tested against these Gram-negative strains due to their cytotoxicity against mammalian cells (see below). Against Gram-negative strains, the quaternary ammonium iodide salts **15a**–**15e** displayed slightly higher activities compared to their corresponding guanidinium hydrochloride salts **22a**–**22d** in most cases.

As the *N*-naphthoyl-phenylglyoxamide derivative **5** was previously reported to possess moderate to high antibacterial activity [45], the effect of replacing the octanesulfonyl group by a naphthoyl group was investigated. Upon replacing the octanesulfonyl group by a naphthoyl group, the antibacterial activity of the ammonium chloride salt **26** was lost, while the corresponding quaternary ammonium iodide salt **27** showed a two-fold decrease in activity (MIC = 32 μM against *S. aureus*; Table 1) compared to the corresponding octanesulfonyl compound **15a**. The tertiary ammonium chloride salt **14a** and quaternary ammonium iodide salt **16a** bearing a butanesulfonyl group were also synthesised in order to investigate the effect of alkyl chain length on antibacterial activity. Upon shortening the octanesulfonyl group to butylsulfonyl (**12a, 14a, 16a**), the antibacterial activity was completely lost (MIC > 250 μM against *S. aureus*; Table 1). These results show that the octanesulfonyl group was the preferred hydrophobic group for high antibacterial activity.

**Figure 3:**
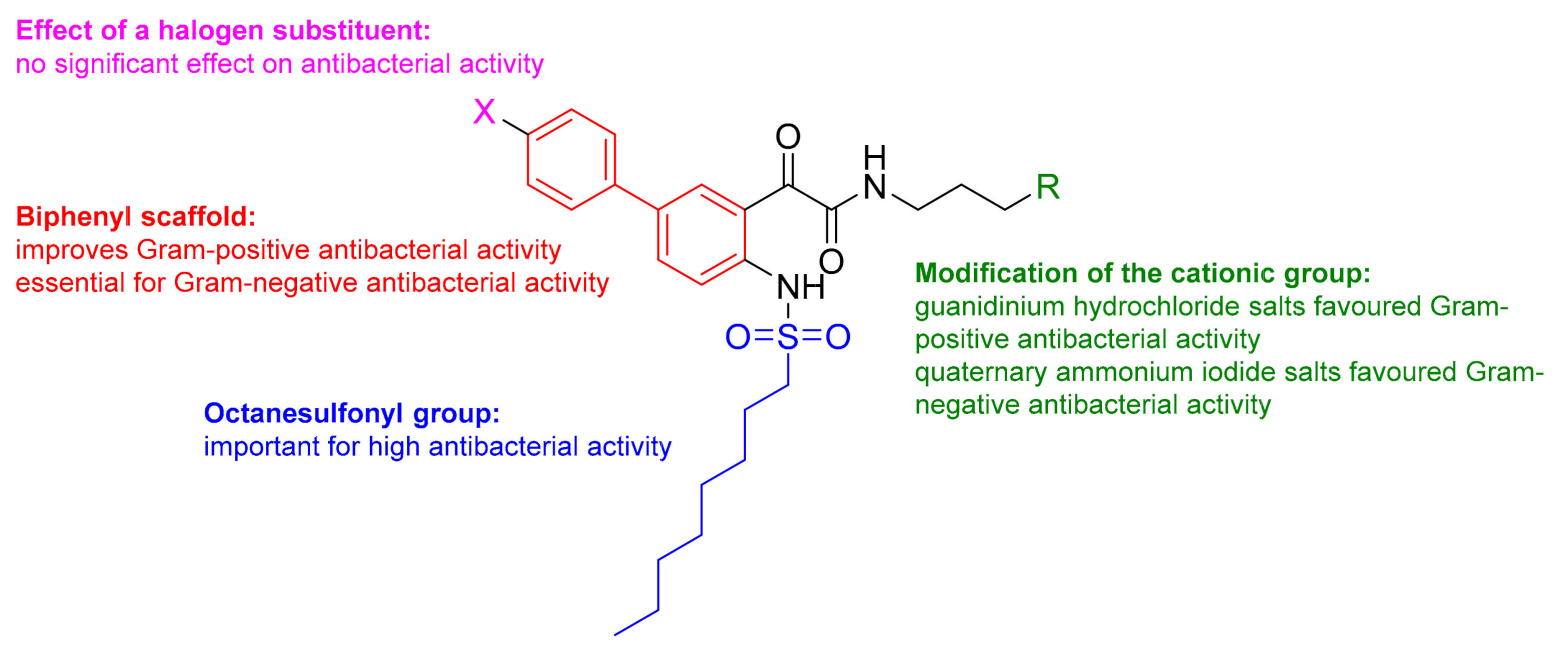
Summary of structure–activity relationships (SARs) for antibacterial activity.

Overall, the SAR analysis for these biphenylglyoxamide-based AMP mimics has demonstrated the importance of the octanesulfonyl group for high antibacterial activity against Gram-positive *S. aureus* (Figure 3). These biphenylglyoxamide-based AMP mimics showed excellent antibacterial activity against *S. aureus* regardless of the substitution or bulkiness of the terminal aryl ring. Against Gram-negative bacteria, the biphenyl scaffold is also essential for antibacterial activities against *P. aeruginosa* and *E. coli.* Out of the four series of compounds, the guanidinium hydrochloride series (series IV) showed the highest antibacterial activity against Gram-positive bacteria, while the quaternary ammonium iodide series (series III) showed the highest antibacterial activity against Gram-negative bacteria.

### 2.3. Antibiofilm Activity

**Figure 4:**
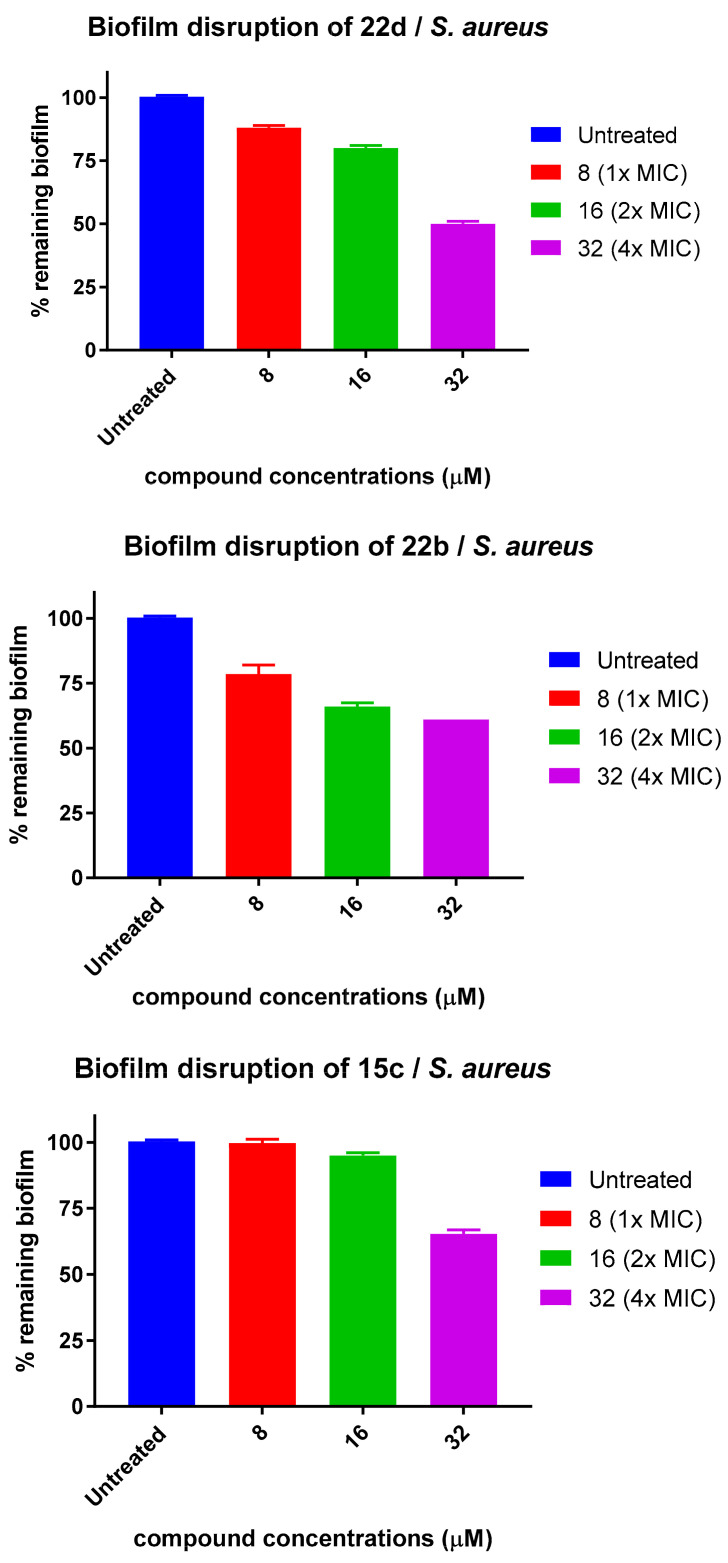
Percentage of remaining *S. aureus* biofilms after 24 h treatment with compounds **22d** (top), **22b** (middle) or **15c** (bottom) at 1×, 2× and 4× of their MIC. Error bars represent the standard error of triplicates (*n* = 3).

The ability of the more potent biphenyl-based antimicrobial peptide mimics (**15c**–**15d, 22a**–**22d**) to disrupt established *S. aureus* biofilms was investigated at 1×, 2× and 4× MIC of the mimics using a crystal violet staining assay (Figure 4). The naphthalene-bearing guanidinium hydrochloride salt **22d** showed the highest level of biofilm disruption among the tested compounds at 4× MIC (32 μM), disrupting 50% of preformed *S. aureus* biofilms, whereas the fluoro-substituted guanidinium hydrochloride salt **22b** and the chloro-substituted quaternary ammonium iodide salt **15c** disrupted 39% and 35% respectively, of preformed *S. aureus* biofilms at 4× MIC. The biofilm disruption ability of these three compounds (**15c, 22b, 22d**) are comparable to that of LL-37, a natural antimicrobial peptide that is being tested in phase II clinical trials that can disrupt mature *S. aureus* biofilms by approximately 40% at 4× its MIC (32 μM) [52].

The remaining three tested antimicrobial peptide mimics (**15d, 22a, 22c**) failed to disrupt any performed *S. aureus* biofilms at any tested concentrations (Supplementary Figure S1), suggesting that they are active against planktonic *S. aureus* cells but are unable to disrupt bacteria that are densely packed in matrices of extracellular polymeric communities.

The chloro-substituted quaternary ammonium iodide salt **15c** and the fluoro-substituted guanidinium hydrochloride salt **22b**, which had high antibacterial activity against *E. coli*, were also tested for their ability to disrupt preformed *E. coli* biofilm at 4× MIC (64 μM). However, these compounds only managed to disrupt an insignificant amount of the preformed *E. coli* biofilm (Supplementary Figure S2). This difference may be due to the different extracellular polymeric substances used by these bacteria to form biofilms. The biofilms of *S. aureus* are composed of β-1,6-*N*-acetyl-D-glucosamine polymer and extracellular DNA, whereas the biofilms of *E. coli* are composed of β-1,6-*N*-acetyl-D-glucosamine polymer, colanic acid and cellulose [53,54,55,56,57].

Overall, the guanidinium hydrochloride salts **22b, 22d** and the quaternary ammonium iodide salt **15c** were the most active antimicrobial peptide mimics against *S. aureus* biofilms.

### 2.4. Cytoplasmic Membrane Depolarisation

The mechanisms of action of the most active compounds were further explored using a membrane dye release assay. It was hypothesised that the mechanism of action of cationic AMP mimics arises from the electrostatic interactions between the cationic head group of the compounds and the negatively charged bacterial cell membrane. In order to verify this hypothesis, 3,3′-dipropylthiadicarbocyanine iodide (diSC3-5), a membrane potential sensitive dye, was employed to monitor bacterial cytoplasmic membrane integrity in the presence of the compounds. This dye readily partitions to and aggregates in the bacterial cell membrane, causing self-quenching of fluorescence when the bacterial cell membrane is intact. However, if the test compound disrupts or induces pore formation in the bacterial cell membrane, the membrane potential gradient would be lost and an increase in fluorescence intensity would be observed due to the release of the dye from the bacterial cell membrane.

**Figure 5:**
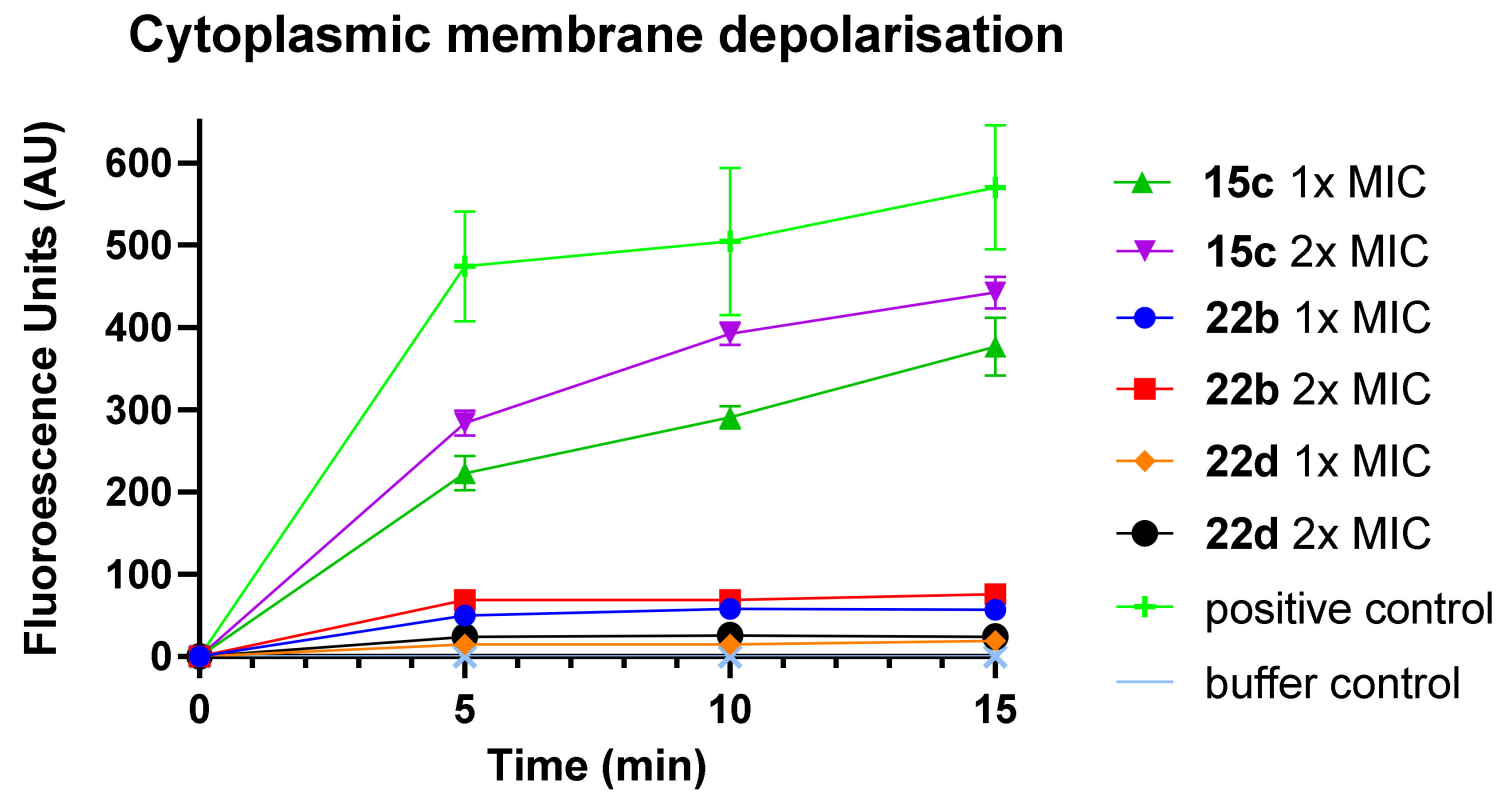
Cytoplasmic membrane disruption of *S. aureus* promoted by compounds **15c, 22b** and **22d** at 1× and 2× of their MIC. 10% DMSO was used as positive control.

As shown in Figure 5, compounds **15c, 22b** and **22d** induced disruption of the cytoplasmic membrane of *S. aureus*, as indicated by the increase of dye fluorescence in a time- and concentration-dependent manner. Out of these three compounds, the quaternary ammonium iodide salt **15c** was the most effective bacterial cytoplasmic membrane disruptor, as evidenced by the largest increase in fluorescence intensity at 1× and 2× MIC within 5 min. The increase in fluorescence intensity for the other two guanidinium hydrochloride salts **22b** and **22d** was modest when compared to that of the quaternary ammonium iodide salt **15c**, suggesting they are less effective in disrupting bacterial cytoplasmic membrane.

**Figure 6:**
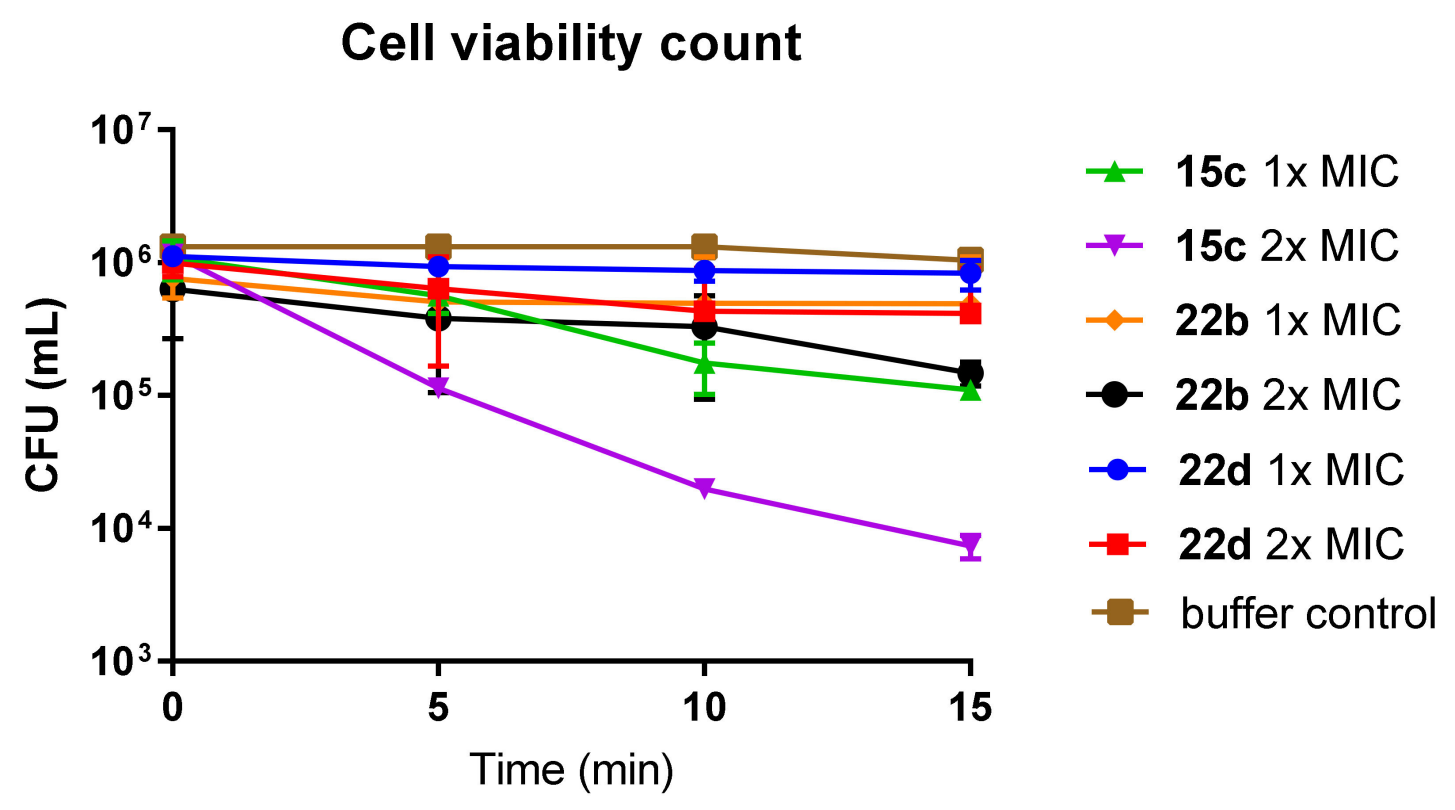
Cell viability count of *S. aureus* in the presence of compound **15c, 22b** and **22d** at 1× and 2× their MIC.

In addition to the cytoplasmic membrane dye release assay, the time-kill kinetic assay was utilised to further investigate the mechanism responsible for the bactericidal effect of the AMP mimics (Figure 6). The time-kill kinetic assay measures bacterial cell viability after the treatment of bacteria with a compound, and hence can indicate the bactericidal activity of a compound over time. The bacterial cell viability of all test compounds, **15c, 22b, 22d**, against *S. aureus* was observed to be time- and concentration-dependent in this assay, which resembled the results observed in the cytoplasmic membrane dye release assay. In this assay, quaternary ammonium iodide salt **15c** showed the highest reduction (2-log and 1-log reductions, respectively) in bacterial numbers at 2× and 1× MIC, while the other two guanidinium hydrochloride salts, **22b** and **22d**, showed smaller reductions (less than 1-log reductions) of bacterial numbers. The trend in the activity of these compounds are consistent with what was observed in the cytoplasmic membrane dye release assay.

Overall, these results suggested that the AMP mimics could exert their antibacterial action via permeating bacterial membranes, with the quaternary ammonium iodide salt **15c** being the most active AMP mimic. However, there might be other mechanisms of action as well, such as effect on intracellular components, and these will be explored in future studies [58].

### 2.5. Lipid Bilayer Membrane Conduction

**Figure 7:**
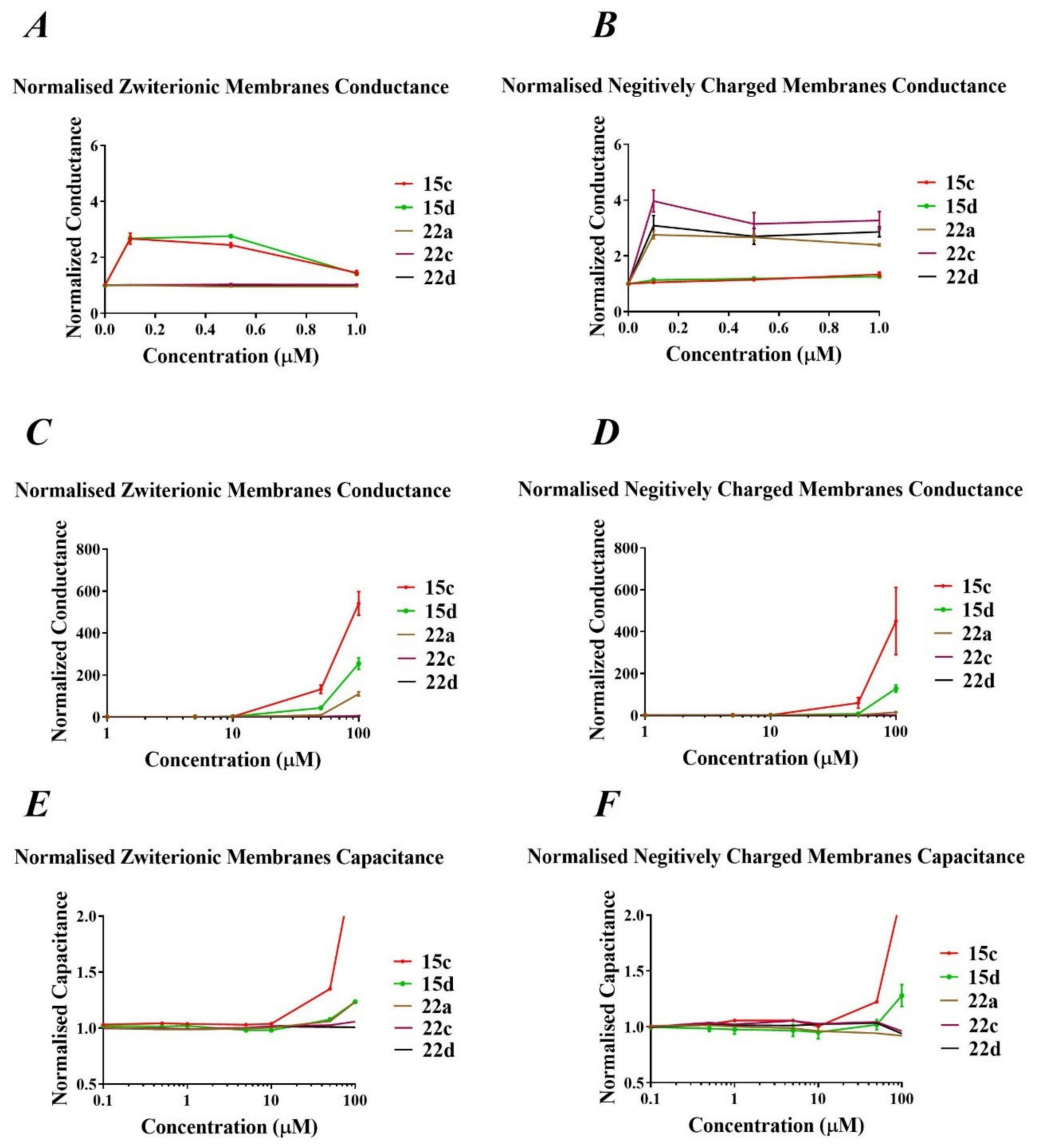
(**A**) Changes in membrane conductance caused by the addition of the compounds at concentrations less than 1 μM in (**A**) zwitterionic POPC tBLMs, and (**B**) tBLMs containing 30% negatively charged POPG lipids. Large increases in membrane conduction due to select compounds are also visible at higher concentrations (1–100 μM) in (**C**) zwitterionic POPC tBLMs, and (**D**) tBLMs containing 30% negatively charged POPG lipids. Large increases in membrane capacitance in (**E**) zwitterionic tBLMs, and (**F**) negatively charged tBLMs, suggests that high concentrations of compounds lead to massive disruption of the lipid bilayers. Error bars represent the standard errors from *n* = 3 replicates for each compound, with the exception of **15c**, which is *n* = 2.

Tethered bilayer lipid membranes (tBLM), in conjunction with AC electrical impedance spectroscopy, were used to assess the ability of selected potent compounds: **15c–15d, 22a,** and **22c**–**22d,** to interact with cell membranes. [59,60] In zwitterionic 1-palmitoyl-2-oleoyl-sn-glycero-3-phosphorylcholine (POPC) tBLMs, quaternary ammonium iodide salts **15c** and **15d** produced a 2.5× shift in membrane conductance at concentrations as low as 100 nM (Figure 7A). In contrast, these changes are notably absent for **15c** and **15d** in membranes that contain 30% negatively charged palmitoyl-oleoyl-phosphatidyglycerol (POPG) lipids, compared to other tested compounds (Figure 7B). Responses to low concentrations of the compounds (<1 μM) in zwitterionic membranes are thought to be due to the compounds producing changes in lipid packing as they insert. Such alterations in the area per lipid, according to the critical packing parameter model of antimicrobial-membrane interactions [61], are associated with changes in the diameter of intrinsic pores in the lipid bilayer.

At higher concentrations of the compounds, the changes of membrane conduction were more pronounced, especially for compounds **15c**, **15d** and **22a** in zwitterionic membrane (Figure 7C). Interestingly, these responses were lower in negatively charged membranes (Figure 7D). The significant increases in membrane conduction suggested that these compounds possess lytic or surfactant-like properties on membranes, in which these compounds sequester lipids from the lipid membrane bilayer via micellisation and eventually disintegrate the membrane [61].

These findings are supported by the large increase of membrane capacitance in both zwitterionic (Figure 7E) and negatively charged tBLMs (Figure 7F) upon treatment of the compounds **15c** and **15d**, suggesting that these membranes have been disintegrated due to the saturating concentrations of these particular compounds. Increases in membrane capacitance indicate a thinning of the lipid membrane and/or the incorporation of a high dielectric, such as water, into the membrane. These results suggest a diminishing of the membrane due to the removal of lipids from the lipid membrane bilayer brought by the surfactant-like effect of compounds.

### 2.6. Cytotoxicity Activity

The cytotoxicity of biphenyl-based AMP mimics was determined in order to evaluate their utility as antimicrobial agents. The in vitro toxicity of selected compounds (**11a, 11d, 13a, 13c**–**13e, 15a**–**15e, 22a**–**22d**) was assessed against MRC-5 normal human fibroblasts using the MTT assay. A dose-response curve for each test compound was generated and their IC_50_ values were determined. The IC_50_ values obtained were then used to calculate the therapeutic indices (IC_50_ value divided by MIC value) against *S. aureus*, *P. aeruginosa* and *E. coli* for each the compound (Table 2). A higher therapeutic index corresponds to a more selective antimicrobial agent.

The glyoxamide compounds **11a** and **11d** were found to possess high toxicity (IC_50_ < 30 μM) towards human cells, resulting in therapeutic indices of below 1.20 against *S. aureus*. This could be due to the lack of cationic charge and the higher hydrophobicity of the compounds allowing them to bind to the zwitterionic human cell membranes more easily [62]. The conversion of the glyoxamide compounds **11a** and **11d** into their corresponding tertiary ammonium chloride salts **13a** and **13d** gave no improvement in the toxicity of the compounds as they possess similar toxicity (IC_50_ < 30 μM) towards human cells. However, owing to their lower MIC values, their therapeutic indices slightly increased to 1.56–2.46 against *S. aureus* compared to the corresponding glyoxamide compounds. In contrast, quaternary ammonium iodide salts **15a–15d** and guanidinium hydrochloride salts **22a**–**22d** showed lower toxicity (IC_50_ > 50 μM), with therapeutic indices of 3.17–13.98 against *S. aureus*. In particular, compounds **15b, 15c, 15d, 22a, 22b** and **22c** with therapeutic indices above 9 against *S. aureus* are promising antimicrobial agents as they are likely to be non-toxic to human cells at the therapeutic dosages required to inhibit bacterial growth.

Owing to the lower potency of the quaternary ammonium iodide salts **15a**–**15d** and guanidinium hydrochloride salts **22a**–**22d** against Gram-negative bacteria, their therapeutic indices against *P. aeruginosa* and *E. coli* were significantly lower compared to that of *S. aureus*. Among these compounds, the fluoro- and chloro-substituted quaternary ammonium iodide salts **15b**–**15c** possessed the highest therapeutic indices of around 6 against *E. coli*. Meanwhile, the fluoro-substituted quaternary ammonium iodide **15b** showed a therapeutic index of 3.04 against *P. aeruginosa*. Notably, compound **15b** was also the least toxic (IC_50_ = 190 μM) against human cells among the compounds tested.

## 3. Materials and Methods

### 3.1. Synthesis of Analogues

#### 3.1.1. General Information

All commercially available reagents were purchased from standard suppliers (Sigma Aldrich, St Louis, MO, USA and Alfa-Aesar, Ward Hill, MA, USA) and used without further purification. All reactions were performed under anhydrous condition with anhydrous solvent unless otherwise specified, and anhydrous solvents were obtained using the PureSolv MD Solvent Purification System. Reactions were monitored by thin-layer chromatography precoated with Merck silica gel 60 F254 and visualisation was performed by using short or long wavelength of ultraviolet light. Flash chromatography was carried out using Grace Davisil LC60A silica.

Melting points were measured using an OptiMelt melting point apparatus and are uncorrected. ^1^H and ^13^C NMR spectra were obtained in the specified solvents on a Bruker Avance III HD 400 (Bruker, Sydney, NSW, Australia) or Bruker Avance III 600 Cryo spectrometer (Bruker, Sydney, NSW, Australia). Chemical shift (δ) are in parts per million (ppm) internally referenced to the solvent nuclei. Multiplicities are assigned as singlet (s), broad singlet (bs), doublet (d), triplet (t), quartet (q), multiplet (m) or a combination of these (e.g., dd, dt, td), and coupling constants (*J*) are reported in Hertz (Hz). Infrared (IR) spectra were recorded using a Cary 630 FTIR spectrometer or Nicolet^TM^ iS^TM^ 10 FTIR spectrometer (Thermo Nicolet, Waltham, MA, USA) fitted with a diamond attenuated total reflectance (ATR) sample interface. Low-resolution mass spectrometry was performed using a Thermo Fisher LCQ Mass Spectrometer (Thermo Scientific, Waltham, MA, USA), while high-resolution mass spectrometry (HRMS) was performed using a Thermo LTQ Orbitrap XL instrument (Thermo Scientific, Waltham, MA, USA).

#### 3.1.2. Synthetic Procedures and Experimental Characterisation Data

##### General Synthetic Procedure A for 5-arylisatins

To a solution mixture of 5-bromoisatin (1.0 equivalent) and the appropriate boronic acid (1.1 equivalents) in degassed (for 30 min) 1:1 toluene/ethanol solution (40 mL), 2 M potassium carbonate solution (2.0 equivalents; degassed for 30 min prior to addition) was added. The dark brown solution was degassed for 30 min. Pd(PPh_3_)_4_ (0.01 equivalents) was then added to the solution mixture and the reaction was heated at 90 °C under nitrogen atmosphere for 24 h. The brownish-black solution was concentrated in vacuo. Water was then added to the reaction mixture and the resulting solution was then acidified to pH 1 with HCl (2 M). The reddish-orange organic layer was extracted thrice with dichloromethane (3 × 30 mL), washed with brine, dried over sodium sulphate and concentrated in vacuo to give the crude product as a red solid. The crude product was purified by flash column chromatography on silica to afford the product.

##### 5-Phenylindoline-2,3-dione (**7a**)

The titled compound was synthesised from 5-bromoisatin (2.02 g, 8.92 mmol), phenylboronic acid (1.20 g, 9.83 mmol), potassium carbonate (2.50 g, 18.06 mmol) and Pd(PPh_3_)_4_ (116 mg, 0.100 mmol) following general synthetic procedure A. The product was obtained as a red solid (1.72 g, 86%); mp 250.3–250.4 °C; ^1^H NMR (400 MHz, DMSO-*d*_6_): δ 11.29 (bs, 1H, NH), 7.91 (dd, *J* = 8.2, 2.0 Hz, 1H, ArH), 7.76 (d, *J* = 1.9 Hz, 1H, ArH), 7.67–7.63 (m, 2H, ArH), 7.48–7.42 (m. 2H, ArH), 7.39–7.33 (m, 1H, ArH), 7.01 (d, *J* = 8.2 Hz, 1H, ArH); ^13^C NMR (100 MHz, DMSO-*d*_6_): δ 184.4 (CO), 159.6 (CO), 150.0 (ArC), 138.7 (ArC), 136.5 (ArCH), 134.9 (ArC), 129.0 (ArCH), 127.5 (ArCH), 126.2 (ArCH), 122.5 (ArCH), 118.4 (ArC), 112.7 (ArCH); IR (ATR): *ν*_max_ 3273, 1759, 1729, 1617, 1508, 1473, 1432, 1308, 1254, 1203, 1183, 1099, 1022, 966, 910, 846, 770, 752, 730, 697, 654, 576, 517, 456, 424 cm^−1^; MS (+ ESI): *m/z* 246.08, [M + Na]^+^.

##### 5-(4-Fluorophenyl)indoline-2,3-dione (**7b**)

The titled compound was synthesised from 5-bromoisatin (1.06 g, 4.70 mmol), 4-fluorophenylboronic acid (0.76 g, 5.40 mmol), potassium carbonate (1.31 g, 9.45 mmol) and Pd(PPh_3_)_4_ (55 mg, 0.048 mmol) following general synthetic procedure A. The product was obtained as a red solid (0.68 g, 60%); mp 238.9–239.3 °C; ^1^H NMR (400 MHz, DMSO-*d*_6_): δ 11.13 (bs, 1H, NH), 7.87 (dd, *J* = 8.2, 2.0 Hz, 1H, ArH), 7.74 (d, *J* = 1.8 Hz, 1H, ArH), 7.72–7.65 (m, 2H, ArH), 7.31–7.23 (m, 2H, ArH), 6.99 (d, *J* = 8.2 Hz, ArH); ^13^C NMR (100 MHz, DMSO-*d*_6_): δ 184.3 (CO), 161.8 (ArC), 159.5 (CO), 149.9 (ArC), 136.4 (ArCH), 135.2 (ArC), 133.9 (ArC), 128.3 (ArCH), 122.5 (ArCH), 118.4 (ArC), 115.8 (ArCH), 112.7 (ArCH); IR (ATR): *ν*_max_ 3324, 3273, 1765, 1739, 1621, 1601, 1589, 1505, 1475, 1454, 1366, 1349, 1307, 1275, 1225, 1192, 1159, 1127, 1098, 1013, 967, 935, 914, 891, 843, 821, 752, 703, 652, 590, 566, 514, 497, 460, 448 cm^−1^; MS (+ ESI): *m/z* 264.00, [M + Na]^+^.

##### 5-(4-Chlorophenyl)indoline-2,3-dione (**7c**)

The titled compound was synthesised from 5-bromoisatin (1.10 g, 4.87 mmol), 4-chlorophenylboronic acid (0.84 g, 5.40 mmol), potassium carbonate (1.37 g, 9.91 mmol) and Pd(PPh_3_)_4_ (78 mg, 0.068 mmol) following general synthetic procedure A. The product was obtained as a red solid (0.72 g, 57%); mp 244.2–244.3 °C; ^1^H NMR (400 MHz, DMSO-*d*_6_): δ 11.15 (bs, 1H, NH), 7.90 (dd, *J* = 8.2, 2.0 Hz, 1H, ArH), 7.78 (d, *J* = 1.9 Hz, 1H, ArH), 7.71–7.67 (m, 2H, ArH), 7.52–7.47 (m, 2H, ArH), 7.00 (d, *J* = 8.2 Hz, 1H, ArH); ^13^C NMR (100 MHz, DMSO-*d*_6_): δ 184.3 (CO), 159.5 (CO), 150.2 (ArC), 137.6 (ArC), 136.4 (ArCH), 133.5 (ArC), 132.3 (ArC), 128.9 (ArCH), 128.0 (ArCH), 122.5 (ArCH), 118.5 (ArC), 112.7 (ArCH); IR (ATR): *ν*_max_ 3327, 3279, 1763, 1742, 1620, 1506, 1474, 1452, 1367, 1349, 1308, 1272, 1258, 1220, 1194, 1160, 1122, 1090, 1012, 966, 915, 842, 821, 811, 753, 742, 703, 655, 584, 567, 541, 513, 497, 460, 448 cm^−1^; MS (+ESI): *m/z* 280.08, [M + Na]^+^.

##### 5-(Naphthalen-2-yl)indoline-2,3-dione (**7d**)

The titled compound was synthesised from 5-bromoisatin (1.05 g, 4.66 mmol), 4-naphthyllboronic acid (0.94 g, 5.19 mmol), potassium carbonate (1.32 g, 9.51 mmol) and Pd(PPh_3_)_4_ (58 mg, 0.050 mmol) following general synthetic procedure A. The product was obtained as a red solid (0.58 g, 46%); mp 293.9–294.0 °C; ^1^H NMR (600 MHz, DMSO-*d*_6_): δ 11.17 (bs, 1H, NH), 8.24 (d, *J* = 1.7 Hz, 1H, ArH), 8.07 (dd, *J* = 8.2, 2.1 Hz, 1H, ArH), 8.01–7.97 (m, 2H, ArH), 7.95–7.92 (m, 2H, ArH), 7.84 (dd, *J* = 8.6, 1.9 Hz, 1H, ArH), 7.56–7.50 (m, 2H, ArH), 7.05 (dd, *J* = 8.2, 0.5 Hz, 1H, ArH); ^13^C NMR (150 MHz, DMSO-*d*_6_): δ 184.4 (CO), 159.6 (CO), 150.0 (ArC), 136.7 (ArCH), 136.0 (ArC), 134.7 (ArC), 133.3 (ArC), 132.2 (ArC), 128.6 (ArCH), 128.2 (ArCH), 127.5 (ArCH), 126.5 (ArCH), 126.2 (ArCH), 124.7 (ArCH), 124.6 (ArCH), 122.7 (ArCH), 118.6 (ArC), 112.8 (ArCH); IR (ATR): *ν*_max_ 3252, 1753, 1732, 1616, 1490, 1459, 1429, 1389, 1344, 1305, 1270, 1250, 1195, 1154, 1119, 980, 905, 891, 863, 846, 808, 752, 698, 636, 622, 598, 577, 560, 525, 496, 474, 460, 443 cm^−1^; HRMS (+ ESI): Found *m/z* 296.0682 [M + Na]^+^, C_18_H_11_NO_2_Na required 296.0682.

##### General Synthetic Procedure B for *N*-sulfonylisatins

To a solution of 5-substituted isatin (1.0 equivalent) in dichloromethane (20 mL), triethylamine (1.1 equivalents) was added at 0 °C under nitrogen atmosphere. The reaction mixture was stirred at 0 °C for 20 min. 1-Octanesulfonyl chloride or 1-butanesulfonyl chloride (1.0 equivalent) was then added slowly dropwise to the reaction mixture at 0 °C with stirring. The reaction mixture was then stirred at room temperature for 3 h. After completion of the reaction, the resulting mixture was concentrated in vacuo and washed with methanol to afford the product.

##### 1-(Octylsulfonyl)-5-phenylindoline-2,3-dione (**8a**)

The titled compound was synthesised from 5-phenylindoline-2,3-dione **7a** (1.70 g, 7.62 mmol), triethylamine (1.20 mL, 8.61 mmol) and 1-octanesulfonyl chloride (1.50 mL, 7.67 mmol) following general synthetic procedure B. The product was obtained as a yellow solid (1.58 g, 52%); mp 131.9–132.2 °C; ^1^H NMR (400 MHz, DMSO-*d*_6_): δ 8.08 (dd, *J* = 8.5, 2.1 Hz, 1H, ArH), 7.98 (d, *J* = 2.0 Hz, 1H, ArH), 7.80 (d, *J* = 8.5 Hz, 1H, ArH), 7.75–7.71 (m, 2H, ArH), 7.50 (t, *J* = 7.8 Hz, 2H, ArH), 7.41 (t, *J* = 7.4 Hz, 1H, ArH), 3.67–3.59 (m, 2H, CH_2_), 1.86–1.76 (m, 2H, CH_2_), 1.43–1.15 (m, 10H, CH_2_), 0.83 (t, *J* = 7.0 Hz, 3H, CH_3_); ^13^C NMR (100 MHz, DMSO-*d*_6_): δ 178.7 (CO), 156.6 (CO), 146.1 (ArC), 138.0 (ArC), 137.0 (ArC), 135.9 (ArCH), 129.1 (ArCH), 128.1 (ArCH), 126.5 (ArCH), 122.4 (ArCH), 120.1 (ArC), 114.6 (ArCH), 53.7 (CH_2_), 31.1 (CH_2_), 28.4 (CH_2_), 28.3 (CH_2_), 27.3 (CH_2_), 22.2 (CH_2_), 22.0 (CH_2_), 13.9 (CH_3_); IR (ATR): *ν*_max_ 3326, 3274, 2915, 2049, 1765, 1737, 1615, 1505, 1472, 1454, 1373, 1349, 1308, 1259, 1222, 1191, 1175, 1160, 1120, 1096, 1012, 967, 945, 913, 850, 821, 762, 754, 703, 651, 609, 592, 566, 532, 513, 497, 461, 448 cm^−1^; HRMS (+ ESI): Found *m/z* 422.1398 [M + Na]^+^, C_22_H_25_NO_4_SNa required 422.1397.

##### 5-(4-Fluorophenyl)-1-(octylsulfonyl)indoline-2,3-dione (**8b**)

The titled compound was synthesised from 5-(4-fluorophenyl)indoline-2,3-dione **7b** (0.54 g, 2.23 mmol), triethylamine (0.35 mL, 2.51 mmol) and 1-octanesulfonyl chloride (0.44 mL, 2.25 mmol) following general synthetic procedure B. The product was obtained as a yellow solid (0.65 g, 70%); mp 142.3–142.7 °C; ^1^H NMR (400 MHz, DMSO-*d*_6_): δ 8.06 (dd, *J* = 8.6, 2.2 Hz, 1H, ArH), 7.98 (d, *J* = 2.0, 1H, ArH), 7.82–7.74 (m, 3H, ArH), 7.36–7.28 (m, 2H, ArH), 3.67–3.59 (m, 2H, CH_2_), 1.86–1.75 (m, 2H, CH_2_), 1.43–1.15 (m, 10H, CH_2_), 0.83 (t, *J* = 7.1 Hz, 3H, CH_3_); ^13^C NMR (100 MHz, DMSO-*d*_6_): δ 178.7 (CO), 162.2 (ArC), 156.6 (CO), 146.1 (ArC), 136.0 (ArC), 135.8 (ArCH), 134.5 (ArC), 128.7 (ArCH), 122.5 (ArCH), 120.1 (ArC), 115.9 (ArCH), 114.6 (ArCH), 53.7 (CH_2_), 31.1 (CH_2_), 28.4 (CH_2_), 28.3 (CH_2_), 27.3 (CH_2_), 22.2 (CH_2_), 22.0 (CH_2_), 13.9 (CH_3_); IR (ATR): *ν*_max_ 3675, 2970, 2900, 1766, 1739, 1615, 1573, 1519, 1475, 1406, 1393, 1373, 1308, 1292, 1232, 1175, 1139, 1066, 1057, 1027, 944, 891, 856, 831, 784, 763, 726, 714, 701, 619, 605, 590, 567, 532, 497, 484, 466, 447 cm^−1^; HRMS (+ ESI): Found *m/z* 440.1303 [M + Na]^+^, C_22_H_24_FNO_4_SNa required 440.1302.

##### 5-(4-Chlorophenyl)-1-(octylsulfonyl)indoline-2,3-dione (**8c**)

The titled compound was synthesised from 5-(4-chlorophenyl)indoline-2,3-dione **7c** (0.36 g, 1.41 mmol), triethylamine (0.22 mL, 1.58 mmol) and 1-octanesulfonyl chloride (0.28 mL, 1.43 mmol) following general synthetic procedure B. The product was obtained as a yellow solid (0.32 g, 52%); mp 171.7–172.1 °C; ^1^H NMR (400 MHz, DMSO-*d*_6_): δ 8.08 (dd, *J* = 8.6, 2.2 Hz, 1H, ArH), 8.01 (d, *J* = 2.0 Hz, 1H, ArH), 7.82–7.75 (m, 3H, ArH), 7.54 (d, *J* = 8.6 Hz, 2H, ArH), 3.67–3.59 (m, 2H, CH_2_), 1.86–1.75 (m, 2H, CH_2_), 1.43–1.15 (m, 10H, CH_2_), 0.83 (t, *J* = 7.0 Hz, 3H, CH_3_); ^13^C NMR (100 MHz, DMSO-*d*_6_): δ 178.6 (CO), 156.6 (CO), 146.3 (ArC), 136.8 (ArC), 135.8 (ArCH), 135.6 (ArC), 132.9 (ArC), 129.0 (ArCH), 128.4 (ArCH), 122.5 (ArCH), 120.2 (ArC), 114.6 (ArCH), 53.7 (CH_2_), 31.1 (CH_2_), 28.4 (CH_2_), 28.3 (CH_2_), 27.3 (CH_2_), 22.2 (CH_2_), 22.0 (CH_2_), 13.9 (CH_3_); IR (ATR): *ν*_max_ 3675, 2970, 2900, 1772, 1746, 1614, 1587, 1568, 1468, 1405, 1394, 1369, 1306, 1287, 1241, 1183, 1168, 1139, 1076, 1066, 1057, 1011, 945, 892, 851, 818, 804, 765, 727, 707, 688, 601, 533, 509, 463, 454, 437 cm^−1^; HRMS (+ ESI): Found *m/z* 456.1007 [M + Na]^+^, C_22_H_24_ClNO_4_SNa required 456.1007.

##### 5-(Naphthalen-2-yl)-1-(octylsulfonyl)indoline-2,3-dione (**8d**)

The titled compound was synthesised from 5-(naphthalen-2-yl)indoline-2,3-dione **7d** (0.42 g, 1.54 mmol), triethylamine (0.24 mL, 1.72 mmol) and 1-octanesulfonyl chloride (0.30 mL, 1.54 mmol) following general synthetic procedure B. The product was obtained as a yellow solid (0.50 g, 73%); mp 125.0-125.4 °C; ^1^H NMR (400 MHz, DMSO-*d*_6_): δ 8.33 (s, 1H, ArH), 8.23 (dd, *J* = 8.6, 2.1 Hz, 1H, ArH), 8.16 (d, *J* = 2.0 Hz, 1H, ArH), 8.05–8.00 (m, 2H, ArH), 7.98–7.94 (m, 1H, ArH), 7.93–7.88 (m, 1H, ArH), 7.85 (d, *J* = 8.6 Hz, 1H, ArH), 7.59–7.52 (m, 2H, ArH), 3.69–3.61 (m, 2H, CH_2_), 1.88-1.77 (m, 2H, CH_2_), 1.44–1.15 (m, 10H, CH_2_), 0.83 (t, *J* = 7.0 Hz, 3H, CH_3_); ^13^C NMR (100 MHz, DMSO-*d*_6_): δ 178.7 (CO), 156.7 (CO), 146.2 (ArC), 136.8 (ArC), 136.0 (ArCH), 135.2 (ArC), 133.3 (ArC), 132.4 (ArC), 128.7 (ArCH), 128.3 (ArCH), 127.5 (ArCH), 126.6 (ArCH), 126.5 (ArCH), 125.3 (ArCH), 124.6 (ArCH), 122.7 (ArCH), 120.2 (ArC), 114.7 (ArCH), 53.7 (CH_2_), 31.1 (CH_2_), 28.4 (CH_2_), 28.4 (CH_2_), 27.3 (CH_2_), 22.2 (CH_2_), 22.0 (CH_2_), 13.9 (CH_3_); IR (ATR): *ν*_max_ 2923, 2852, 1766, 1737, 1615, 1578, 1488, 1460, 1424, 1374, 1306, 1293, 1270, 1259, 1235, 1198, 1173, 1136, 1110, 1094, 1038, 1017, 998, 953, 937, 922, 893, 872, 852, 824, 783, 763, 751, 744, 721, 700, 659, 632, 613, 589, 560, 532, 497, 474, 446 cm^−1^; HRMS (+ ESI): Found *m/z* 472.1554 [M + Na]^+^, C_26_H_27_NO_4_SNa required 472.1553.

##### 5-Butyl-1-(octylsulfonyl)indoline-2,3-dione (**8e**)

The titled compound was synthesised from 5-butylindoline-2,3-dione **7e** (0.40 g, 1.95 mmol), triethylamine (0.30 mL, 2.15 mmol) and 1-octanesulfonyl chloride (0.39 mL, 1.99 mmol) following general synthetic procedure B. The product was obtained as a yellow solid (0.41 g, 55%); mp 113.2–113.6 °C; ^1^H NMR (400 MHz, DMSO-*d*_6_): δ 7.64–7.53 (m, 3H, ArH), 3.62–3.56 (m, 2H, CH_2_), 2.62 (t, *J* = 7.7 Hz, 2H, CH_2_), 1.82–1.72 (m, 2H, CH_2_), 1.59–1.49 (m, 2H, CH_2_), 1.41–1.16 (m, 12H, CH_2_), 0.89 (t, *J* = 7.5 Hz, 3H, CH_3_), 0.84 (t, *J* = 7.0 Hz, 3H, CH_3_); ^13^C NMR (100 MHz, DMSO-*d*_6_): δ 178.9 (CO), 156.7 (CO), 145.1 (ArC), 139.5 (ArC), 137.8 (ArCH), 124.4 (ArCH), 119.4 (ArC), 114.0 (ArCH), 53.6 (CH_2_), 33.7 (CH_2_), 32.9 (CH_2_), 31.1 (CH_2_), 28.4 (CH_2_), 28.3 (CH_2_), 27.3 (CH_2_), 22.2 (CH_2_), 22.0 (CH_2_), 21.6 (CH_2_), 13.9 (CH_3_), 13.7 (CH_3_); IR (ATR): *ν*_max_ 3854, 3675, 2968, 2911, 2362, 1762, 1737, 1615, 1584, 1483, 1467, 1406, 1393, 1372, 1293, 1250, 1241, 1223, 1177, 1152, 1134, 1116, 1077, 1066, 1057, 1027, 954, 868, 851, 782, 724, 706, 653, 605, 565, 532, 484, 462, 446 cm^−1^; HRMS (+ ESI): Found *m/z* 402.1711 [M + Na]^+^, C_20_H_29_NO_4_SNa required 402.1710.

##### 1-(Butylsulfonyl)-5-phenylindoline-2,3-dione (**9a**)

The titled compound was synthesised from 5-phenylindoline-2,3-dione **7a** (0.45 g, 1.92 mmol), triethylamine (0.30 mL, 2.15 mmol) and 1-butanesulfonyl chloride (0.25 mL, 1.93 mmol) following general synthetic procedure B. The product was obtained as a yellow sticky solid (0.23 g, 34%); ^1^H NMR (400 MHz, DMSO-*d*_6_): δ 8.08 (dd, *J* = 8.6, 2.2 Hz, 1H, ArH), 7.98 (d, *J* = 2.1 Hz, 1H, ArH), 7.80 (d, *J* = 8.6 Hz, 1H, ArH), 7.75–7.71 (m, 2H, ArH), 7.50 (t, *J* = 7.8 Hz, 2H, ArH), 7.41 (t, *J* = 7.3 Hz, 1H, ArH), 3.67–3.60 (m, 2H, CH_2_), 1.85–1.76 (m, 2H, CH_2_), 1.47–1.36 (m, 2H, CH_2_), 0.88 (t, *J* = 7.4 Hz, 3H, CH_3_); ^13^C NMR (100 MHz, DMSO-*d*_6_): δ 178.7 (CO), 156.6 (CO), 146.1 (ArC), 138.0 (ArC), 137.0 (ArC), 135.9 (ArCH), 129.1 (ArCH), 128.0 (ArCH), 126.5 (ArCH), 122.4 (ArCH), 120.1 (ArC), 114.6 (ArCH), 53.5 (CH_2_), 24.1 (CH_2_), 20.7 (CH_2_), 13.3 (CH_3_); IR (ATR): *ν*_max_ 3196, 2961, 2873, 1777, 1736, 1648, 1615, 1588, 1508, 1485, 1472, 1455, 1399, 1368, 1339, 1310, 1293, 1270, 1241, 1183, 1171, 1139, 1117, 1040, 1000, 982, 949, 917, 842, 758, 734, 694, 674, 651, 620, 602, 579, 556, 532, 516, 464, 426 cm^−1^; HRMS (+ ESI): Found *m/z* 366.0771 [M + Na]^+^, C_18_H_17_NO_4_SNa required 366.0770.

##### General Synthetic Procedure C for Glyoxamide Derivatives

To a solution of *N*-sulfonylisatin (1.0 equivalent) in dichloromethane (5 mL), 3-dimethylaminopropylamine (1.0 equivalent) was added at 0 °C. The reaction mixture was stirred at room temperature for 6 h. After completion of the reaction, water was added to the reaction mixture and the product was extracted into dichloromethane (3 × 30 mL), washed with brine, dried over anhydrous sodium sulphate and concentrated in vacuo to afford the product.

##### *N*-(3-(Dimethylamino)propyl)-2-(4-(octylsulfonamido)-[1,1’-biphenyl]-3-yl)-2-oxoacetamide (**11a**)

The titled compound was synthesised from 1-(octylsulfonyl)-5-phenylindoline-2,3-dione **8a** (0.11 g, 0.28 mmol) and 3-dimethylaminopropylamine (35 μL, 0.28 mmol) following general synthetic procedure C. The product was obtained as a yellow oil (0.13 g, 96%); ^1^H NMR (400 MHz, CDCl_3_): δ 8.78 (bs, 1H, NH), 8.72 (d, *J* = 1.9 Hz, 1H, ArH), 7.87–7.80 (m, 2H, ArH), 7.60–7.55 (m, 2H, ArH), 7.47–7.41 (m, 2H, ArH), 7.39–7.34 (m, 1H, ArH), 3.53 (t, *J* = 6.0 Hz, 2H, CH_2_), 3.21–3.15 (m, 2H, CH_2_), 2.55 (t, *J* = 6.2 Hz, 2H, CH_2_), 2.33 (s, 6H, CH_3_), 1.86–1.76 (m, 4H, CH_2_), 1.43–1.15 (m, 10H, CH_2_), 0.85 (t, *J* = 6.6 Hz, 3H, CH_3_); ^13^C NMR (100 MHz, CDCl_3_): δ 192.0 (CO), 162.8 (CO), 140.9 (ArC), 139.1 (ArC), 135.8 (ArC), 134.9 (ArCH), 133.6 (ArCH), 129.1 (ArCH), 127.9 (ArCH), 127.0 (ArCH), 120.0 (ArC), 118.6 (ArCH), 58.6 (CH_2_), 52.7 (CH_2_), 45.2 (CH_3_), 39.8 (CH_2_), 31.8 (CH_2_), 29.1 (CH_2_), 29.0 (CH_2_), 28.2 (CH_2_), 25.3 (CH_2_), 23.5 (CH_2_), 22.7 (CH_2_), 14.2 (CH_3_); IR (ATR): *ν*_max_ 3327, 3274, 2924, 2854, 1765, 1738, 1616, 1508, 1476, 1395, 1367, 1308, 1261, 1225, 1195, 1143, 1098, 1013, 967, 935, 821, 760, 698, 655, 585, 566, 514, 497, 460, 448 cm^−1^; HRMS (+ ESI): Found *m/z* 502.2734 [M + H]^+^, C_27_H_40_N_3_O_4_S required 502.2734.

##### *N*-(3-(Dimethylamino)propyl)-2-(4’-fluoro-4-(octylsulfonamido)-[1,1’-biphenyl]-3-yl)-2-oxoacetamide (**11b**)

The titled compound was synthesised from 5-(4-fluorophenyl)-1-(octylsulfonyl)indoline-2,3-dione **8b** (0.13 g, 0.32 mmol) and 3-dimethylaminopropylamine (40 μL, 0.32 mmol) following general synthetic procedure C. The product was obtained as a yellow oil (0.16 g, 99%); ^1^H NMR (400 MHz, CDCl_3_): δ 8.86 (bs, 1H, NH), 8.71 (d, *J* = 2.2 Hz, 1H, ArH), 7.84 (d, *J* = 8.7 Hz, 1H, ArH), 7.77 (dd, *J* = 8.7, 2.2 Hz, 1H, ArH), 7.56–7.50 (m, 2H, ArH), 7.16–7.09 (m, 2H, ArH), 3.53 (t, *J* = 6.0 Hz, 2H, CH_2_), 3.20–3.14 (m, 2H, CH_2_), 2.52 (t, *J* = 6.1 Hz, 2H, CH_2_), 2.30 (s, 6H, CH_3_), 1.86–1.74 (m, 4H, CH_2_), 1.43–1.16 (m, 10H, CH_2_), 0.85 (t, *J* = 7.2 Hz, 3H, CH_3_); ^13^C NMR (100 MHz, CDCl_3_): δ 191.8 (CO), 162.8 (ArC), 162.6 (CO), 140.9 (ArC), 135.3 (ArC), 134.8 (ArC), 134.7 (ArCH), 133.5 (ArCH), 128.6 (ArCH), 119.6 (ArC), 118.6 (ArCH), 116.1 (ArCH), 58.8 (CH_2_), 52.7 (CH_2_), 45.3 (CH_3_), 40.0 (CH_2_), 31.8 (CH_2_), 29.1 (CH_2_), 29.0 (CH_2_), 28.2 (CH_2_), 25.3 (CH_2_), 23.5 (CH_2_), 22.7 (CH_2_), 14.2 (CH_3_); IR (ATR): *ν*_max_ 3675, 2970, 2923, 1659, 1635, 1571, 1491, 1393, 1338, 1261, 1222, 1200, 1139, 1066, 1057, 921, 821, 770, 724, 677, 595, 563, 520, 488, 419 cm^−1^; HRMS (+ ESI): Found *m/z* 520.2641 [M + H]^+^, C_27_H_39_FN_3_O_4_S required 520.2640.

##### 2-(4’-Chloro-4-(octylsulfonamido)-[1,1’-biphenyl]-3-yl)-*N*-(3-(dimethylamino)propyl)-2-oxoacetamide (**11c**)

The titled compound was synthesised from 5-(4-chlorophenyl)-1-(octylsulfonyl)indoline-2,3-dione **8c** (0.10 g, 0.24 mmol) and 3-dimethylaminopropylamine (30 μL, 0.24 mmol) following general synthetic procedure C. The product was obtained as a yellow oil (0.12 g, 94%); ^1^H NMR (400 MHz, CDCl_3_): δ 8.88 (bs, 1H, NH), 8.75 (d, *J* = 2.2 Hz, 1H, ArH), 7.85 (d, *J* = 8.8 Hz, 1H, ArH), 7.78 (dd, *J* = 8.8, 2.3 Hz, 1H, ArH), 7.54–7.47 (m, 2H, ArH), 7.43-7.38 (m, 2H, ArH), 3.52 (t, *J* = 6.0 Hz, 2H, CH_2_), 3.21–3.14 (m, 2H, CH_2_), 2.53–2.48 (m, 2H, CH_2_), 2.29 (s, 6H, CH_3_), 1.86–1.74 (m, 4H, CH_2_), 1.42–1.16 (m, 10H, CH_2_), 0.85 (t, *J* = 7.1 Hz, 3H, CH_3_); ^13^C NMR (100 MHz, CDCl_3_): δ 191.7 (CO), 162.5 (CO), 141.2 (ArC), 137.6 (ArC), 134.6 (ArCH), 134.4 (ArC), 134.0 (ArC), 133.5 (ArCH), 129.3 (ArCH), 128.2 (ArCH), 119.6 (ArC), 118.6 (ArCH), 58.9 (CH_2_), 52.8 (CH_2_), 45.4 (CH_3_), 40.1 (CH_2_), 31.8 (CH_2_), 29.1 (CH_2_), 29.0 (CH_2_), 28.2 (CH_2_), 25.3 (CH_2_), 23.5 (CH_2_), 22.7 (CH_2_), 14.2 (CH_3_); IR (ATR): *ν*_max_ 3675, 2969, 2922, 1773, 1746, 1636, 1615, 1507, 1482, 1467, 1394, 1338, 1260, 1198, 1140, 1076, 1066, 1012, 919, 852, 817, 773, 697, 600, 564, 539, 509, 492, 436, 426 cm^−1^; HRMS (+ ESI): Found *m/z* 536.2344 [M + H]^+^, C_27_H_39_ClN_3_O_4_S required 536.2344.

##### *N*-(3-(Dimethylamino)propyl)-2-(5-(naphthalen-2-yl)-2-(octylsulfonamido)phenyl)-2-oxoacetamide (**11d**)

The titled compound was synthesised from 5-(naphthalen-2-yl)-1-(octylsulfonyl)indoline-2,3-dione **8d** (0.15 g, 0.33 mmol) and 3-dimethylaminopropylamine (42 μL, 0.33 mmol) following general synthetic procedure C. The product was obtained as a yellow oil (0.18 g, 96%); ^1^H NMR (400 MHz, CDCl_3_): δ 8.87 (d, *J* = 2.1 Hz, 1H, ArH), 8.85 (bs, 1H, NH), 8.02 (s, 1H, ArH), 7.98–7.84 (m, 5H, ArH), 7.71 (dd, *J* = 8.5, 1.8 Hz, 1H, ArH), 7.55–7.47 (m, 2H, ArH), 3.55 (t, *J* = 5.9 Hz, 2H, CH_2_), 3.23–3.16 (m, 2H, CH_2_), 2.52 (t, *J* = 6.2 Hz, 2H, CH_2_), 2.30 (s, 6H, CH_3_), 1.87–1.75 (m, 4H, CH_2_), 1.43–1.17 (m, 10H, CH_2_), 0.85 (t, *J* = 7.1 Hz, 3H, CH_3_); ^13^C NMR (100 MHz, CDCl_3_): δ 191.9 (CO), 162.7 (CO), 141.0 (ArC), 136.4 (ArC), 135.7 (ArC), 135.2 (ArCH), 133.9 (ArCH), 133.7 (ArC), 132.9 (ArC), 128.9 (ArCH), 128.4 (ArCH), 127.8 (ArCH), 126.7 (ArCH), 126.4 (ArCH), 125.7 (ArCH), 125.1 (ArCH), 119.7 (ArC), 118.7 (ArCH), 58.9 (CH_2_), 52.7 (CH_2_), 45.4 (CH_3_), 40.1 (CH_2_), 31.8 (CH_2_), 29.1 (CH_2_), 29.0 (CH_2_), 28.2 (CH_2_), 25.3 (CH_2_), 23.6 (CH_2_), 22.7 (CH_2_), 14.2 (CH_3_); IR (ATR): *ν*_max_ 3675, 2924, 2855, 1767, 1739, 1641, 1616, 1577, 1492, 1462, 1394, 1333, 1262, 1234, 1201, 1142, 1098, 918, 892, 815, 747, 720, 670, 593, 561, 516, 475 cm^−1^; HRMS (+ ESI): Found *m/z* 552.2895 [M + H]^+^, C_31_H_42_N_3_O_4_S required 552.2891.

##### 2-(5-Butyl-2-(octylsulfonamido)phenyl)-*N*-(3-(dimethylamino)propyl)-2-oxoacetamide (**11e**)

The titled compound was synthesised from 5-butyl-1-(octylsulfonyl)indoline-2,3-dione **8e** (0.15 g, 0.40 mmol) and 3-dimethylaminopropylamine (50 μL, 0.40 mmol) following general synthetic procedure C. The product was obtained as a yellow oil (0.18 g, 95%); ^1^H NMR (400 MHz, CDCl_3_): δ 8.65 (bs, 1H, NH), 8.21 (d, *J* = 1.8 Hz, 1H, ArH), 7.67 (d, *J* = 8.5 Hz, 1H, ArH), 7.41 (dd, *J* = 8.6, 2.0 Hz, 1H, ArH), 3.56–3.48 (m, 2H, CH_2_), 3.14–3.08 (m, 2H, CH_2_), 2.63–2.52 (m, 4H, CH_2_), 2.34 (s, 6H, CH_3_), 1.86–1.71 (m, 4H, CH_2_), 1.63–1.52 (m, 2H, CH_2_), 1.41–1.17 (m, 12H, CH_2_), 0.92 (t, *J* = 7.3 Hz, 3H, CH_3_), 0.85 (t, *J* = 7.0 Hz, 3H, CH_3_); ^13^C NMR (100 MHz, CDCl_3_): δ 192.1 (CO), 163.0 (CO), 139.5 (ArC), 137.6 (ArC), 136.7 (ArCH), 134.6 (ArCH), 119.6 (ArC), 118.5 (ArCH), 58.6 (CH_2_), 52.4 (CH_2_), 45.2 (CH_3_), 39.6 (CH_2_), 34.9 (CH_2_), 33.6 (CH_2_), 31.8 (CH_2_), 29.1 (CH_2_), 29.0 (CH_2_), 28.2 (CH_2_), 25.3 (CH_2_), 23.5 (CH_2_), 22.7 (CH_2_), 22.4 (CH_2_), 14.2 (CH_3_), 14.0 (CH_3_); IR (ATR): *ν*_max_ 3674, 2968, 2922, 1609, 1497, 1458, 1405, 1393, 1331, 1256, 1141, 1066, 1057, 934, 834, 781, 732, 668, 562, 520 cm^−1^; HRMS (+ ESI): Found *m/z* 482.3047 [M + H]^+^, C_25_H_44_N_3_O_4_S required 482.3047.

##### 2-(4-(Butylsulfonamido)-[1,1’-biphenyl]-3-yl)-*N*-(3-(dimethylamino)propyl)-2-oxoacetamide (**12a**)

The titled compound was synthesised from 1-(butylsulfonyl)-5-phenylindoline-2,3-dione **9a** (0.15 g, 0.44 mmol) and 3-dimethylaminopropylamine (55 μL, 0.44 mmol) following general synthetic procedure C. The product was obtained as a yellow oil (0.18 g, 93%); ^1^H NMR (400 MHz, CDCl_3_): δ 8.81 (bs, 1H, NH), 8.73 (d, *J* = 2.0 Hz, 1H, ArH), 7.87–7.80 (m, 2H, ArH), 7.60–7.55 (m, 2H, ArH), 7.44 (t, *J* = 8.0 Hz, 2H, ArH), 7.39–7.33 (m, 1H, ArH), 3.53 (t, *J* = 6.2 Hz, 2H, CH_2_), 3.22–3.15 (m, 2H, CH_2_), 2.51 (t, *J* = 6.2 Hz, 2H, CH_2_), 2.29 (s, 6H, CH_3_), 1.84–1.74 (m, 4H, CH_2_), 1.47–1.36 (m, 2H, CH_2_), 0.90 (t, *J* = 7.3 Hz, 3H, CH_3_); ^13^C NMR (100 MHz, CDCl_3_): δ 192.0 (CO), 162.7 (CO), 140.9 (ArC), 139.1 (ArC), 135.8 (ArC), 134.9 (ArCH), 133.6 (ArCH), 129.1 (ArCH), 127.9 (ArCH), 127.0 (ArCH), 119.6 (ArC), 118.6 (ArCH), 58.8 (CH_2_), 52.4 (CH_2_), 45.3 (CH_3_), 40.0 (CH_2_), 25.5 (CH_2_), 25.3 (CH_2_), 21.5 (CH_2_), 13.6 (CH_3_); IR (ATR): *ν*_max_ 2960, 2871, 2363, 2345, 2183, 2160, 2049, 1978, 1870, 1773, 1734, 1710, 1701, 1685, 1670, 1663, 1654, 1647, 1636, 1617, 1578, 1570, 1560, 1541, 1534, 1522, 1508, 1482, 1466, 1459, 1449, 1395, 1330, 1259, 1194, 1142, 1096, 1025, 921, 794, 761, 733, 697, 681, 669, 616, 583, 555, 535, 477, 428 cm^−1^; HRMS (+ ESI): Found *m/z* 466.2109 [M + H]^+^, C_23_H_32_N_3_O_4_S required 466.2108.

##### General Synthetic Procedure D for Tertiary Ammonium Chloride Salts

To a solution of glyoxamide derivative (1.0 equivalent) in diethyl ether (5 mL), 4 M HCl/dioxane (5.0 equivalents) was added. The reaction mixture was stirred at room temperature for 20 min. After completion of reaction, the reaction mixture was concentrated in vacuo, washed thrice with diethyl ether and freeze-dried to afford the product.

##### *N*,*N*-Dimethyl-3-(2-(4-(octylsulfonamido)-[1,1’-biphenyl]-3-yl)-2-oxoacetamido)propan-1-aminium chloride (**13a**)

The titled compound was synthesised from *N*-(3-(dimethylamino)propyl)-2-(4-(octylsulfonamido)-[1,1’-biphenyl]-3-yl)-2-oxoacetamide **11a** (33 mg, 0.066 mmol) and 4 M HCl/dioxane (0.10 mL, 0.40 mmol) following general synthetic procedure D. The product was obtained as a yellow sticky solid (33 mg, 93%); ^1^H NMR (600 MHz, DMSO-*d*_6_): δ 10.25 (bs, 2H, NH), 9.03 (t, *J* = 5.8 Hz, 1H, NH), 8.01–7.98 (m, 2H, ArH), 7.67–7.60 (m, 3H, ArH), 7.52–7.48 (m, 2H, ArH), 7.40 (t, *J* = 7.4 Hz, 1H, ArH), 3.32 (t, *J* = 6.5 Hz, 2H, CH_2_), 3.24–3.20 (m, 2H, CH_2_), 3.12–3.07 (m, 2H, CH_2_), 2.73 (s, 6H, CH_3_), 1.97–1.90 (m, 2H, CH_2_), 1.70–1.64 (m, 2H, CH_2_), 1.37–1.16 (m, 10H, CH_2_), 0.82 (t, *J* = 7.1 Hz, 3H, CH_3_); ^13^C NMR (150 MHz, DMSO-*d*_6_): δ 192.1 (CO), 163.8 (CO), 138.3 (ArC), 137.9 (ArC), 135.9 (ArC), 132.9 (ArCH), 130.3 (ArCH), 129.2 (ArCH), 127.9 (ArCH), 126.5 (ArCH), 125.8 (ArC), 122.3 (ArCH), 54.4 (CH_2_), 51.3 (CH_2_), 42.0 (CH_3_), 36.0 (CH_2_), 31.1 (CH_2_), 28.4 (CH_2_), 28.3 (CH_2_), 27.3 (CH_2_), 23.9 (CH_2_), 22.9 (CH_2_), 22.0 (CH_2_), 13.9 (CH_3_); IR (ATR): *ν*_max_ 3382, 2924, 2854, 2703, 1647, 1581, 1508, 1483, 1395, 1331, 1267, 1197, 1144, 1075, 974, 919, 842, 761, 697, 681, 616, 586, 509 cm^−1^; HRMS (+ ESI): Found *m/z* 502.2731 [M + H]^+^, C_27_H_40_N_3_O_4_S required 502.2734.

##### 3-(2-(4’-Fluoro-4-(octylsulfonamido)-[1,1’-biphenyl]-3-yl)-2-oxoacetamido)-*N*,*N*-dimethylpropan-1-aminium chloride (**13b**)

The titled compound was synthesised from *N*-(3-(dimethylamino)propyl)-2-(4’-fluoro-4-(octylsulfonamido)-[1,1’-biphenyl]-3-yl)-2-oxoacetamide **11b** (30 mg, 0.058 mmol) and 4 M HCl/dioxane (0.10 mL, 0.40 mmol) following general synthetic procedure D. The product was obtained as a yellow sticky solid (32 mg, 99%); ^1^H NMR (600 MHz, DMSO-*d*_6_): δ 10.43 (bs, 1H, NH), 10.18 (bs, 1H, NH), 9.02 (t, *J* = 6.0 Hz, NH), 8.00–7.95 (m, 2H, ArH), 7.73–7.68 (m, 2H, ArH), 7.60 (d, *J* = 9.0 Hz, 1H, ArH), 7.36–7.31 (m, 2H, ArH), 3.33-3.29 (m, 2H, CH_2_), 3.21 (t, *J* = 7.9 Hz, 2H, CH_2_), 3.12–3.07 (m, 2H, CH_2_), 2.73 (s, 6H, CH_3_), 1.98–1.90 (m, 2H, CH_2_), 1.70–1.63 (m, 2H, CH_2_), 1.37–1.15 (m, 10H, CH_2_), 0.82 (t, *J* = 7.2 Hz, 3H, CH_3_); ^13^C NMR (150 MHz, DMSO-*d*_6_): δ 192.0 (CO), 163.7 (CO), 162.1 (ArC), 137.8 (ArC), 135.0 (ArC), 134.8 (ArC), 132.8 (ArCH), 130.1 (ArCH), 128.6 (ArCH), 126.1 (ArC), 122.5 (ArCH), 116.0 (ArCH), 54.4 (CH_2_), 51.3 (CH_2_), 42.0 (CH_3_), 42.0 (CH_3_), 36.0 (CH_2_), 31.1 (CH_2_), 28.4 (CH_2_), 28.3 (CH_2_), 27.3 (CH_2_), 23.9 (CH_2_), 22.9 (CH_2_), 22.0 (CH_2_), 13.9 (CH_3_); IR (ATR): *ν*_max_ 3358, 2925, 2855, 2690, 2360, 1647, 1603, 1515, 1488, 1400, 1332, 1222, 1196, 1143, 1099, 1012, 975, 919, 828, 725, 670, 597, 559, 520, 418 cm^−1^; HRMS (+ESI): Found *m/z* 520.2641 [M + H]^+^, C_27_H_39_FN_3_O_4_S required 520.2640.

##### 3-(2-(4’-Chloro-4-(octylsulfonamido)-[1,1’-biphenyl]-3-yl)-2-oxoacetamido)-*N*,*N*-dimethylpropan-1-aminium chloride (**13c**)

The titled compound was synthesised from 2-(4’-chloro-4-(octylsulfonamido)-[1,1’-biphenyl]-3-yl)-*N*-(3-(dimethylamino)propyl)-2-oxoacetamide **11c** (30 mg, 0.056 mmol) and 4 M HCl/dioxane (0.10 mL, 0.40 mmol) following general synthetic procedure D. The product was obtained as a yellow sticky solid (29 mg, 92%); ^1^H NMR (600 MHz, DMSO-*d*_6_): δ 10.37 (bs, 1H, NH), 10.25 (bs, 1H, NH), 9.02 (t, *J* = 6.0 Hz, 1H, NH), 8.02–7.98 (m, 2H, ArH), 7.71–7.68 (m, 2H, ArH), 7.63–7.60 (m, 1H, ArH), 7.57–7.54 (m, 2H, ArH), 3.34–3.30 (m, 2H, CH_2_), 3.21 (t, *J* = 7.8 Hz, 2H, CH_2_), 3.12–3.07 (m, 2H, CH_2_), 2.73 (s, 6H, CH_3_), 1.98-1.90 (m, 2H, CH_2_), 1.70–1.63 (m, 2H, CH_2_), 1.36–1.15 (m, 10H, CH_2_), 0.82 (t, *J* = 7.2 Hz, 3H, CH_3_); ^13^C NMR (150 MHz, DMSO-*d*_6_): δ 191.9 (CO), 163.6 (CO), 138.1 (ArC), 137.1 (ArC), 134.6 (ArC), 132.8 (ArC), 132.7 (ArCH), 130.2 (ArCH), 129.2 (ArCH), 128.3 (ArCH), 126.1 (ArC), 122.5 (ArCH), 54.4 (CH_2_), 51.4 (CH_2_), 42.1 (CH_3_), 36.0 (CH_2_), 31.1 (CH_2_), 28.4 (CH_2_), 28.3 (CH_2_), 27.3 (CH_2_), 23.9 (CH_2_), 22.9 (CH_2_), 22.0 (CH_2_), 13.9 (CH_3_); IR (ATR): *ν*_max_ 3372, 2924, 2854, 2704, 2359, 1643, 1578, 1507, 1481, 1400, 1333, 1273, 1196, 1143, 1092, 1012, 974, 918, 818, 758, 697, 668, 593, 511, 488 cm^−1^; HRMS (+ ESI): Found *m/z* 536.2346 [M + H]^+^, C_27_H_39_ClN_3_O_4_S required 536.2344.

##### *N*,*N*-Dimethyl-3-(2-(5-(naphthalen-2-yl)-2-(octylsulfonamido)phenyl)-2-oxoacetamido)propan-1-aminium chloride (**13d**)

The titled compound was synthesised from *N*-(3-(dimethylamino)propyl)-2-(5-(naphthalen-2-yl)-2-(octylsulfonamido)phenyl)-2-oxoacetamide **11d** (32 mg, 0.058 mmol) and 4 M HCl/dioxane (0.10 mL, 0.40 mmol) following general synthetic procedure D. The product was obtained as a yellow sticky solid (33 mg, 96%); ^1^H NMR (600 MHz, DMSO-*d*_6_): δ 10.26 (bs, 2H, NH), 9.03 (t, *J* = 5.9 Hz, 1H, NH), 8.23 (s, 1H, ArH), 8.16–8.13 (m, 2H, ArH), 8.06–8.01 (m, 2H, ArH), 7.96 (d, *J* = 7.6 Hz, 1H, ArH), 7.83 (dd, *J* = 8.5, 1.4 Hz, 1H, ArH), 7.65 (d, *J* = 9.1 Hz, 1H, ArH), 7.60–7.52 (m, 2H, ArH), 3.35–3.29 (m, 2H, CH_2_), 3.22 (t, *J* = 7.7 Hz, 2H, CH_2_), 3.14–3.09 (m, 2H, CH_2_), 2.74 (s, 6H, CH_3_), 1.99–1.92 (m, 2H, CH_2_), 1.72–1.64 (m, 2H, CH_2_), 1.39–1.14 (m, 10H, CH_2_), 0.82 (t, *J* = 7.1 Hz, 3H, CH_3_); ^13^C NMR (150 MHz, DMSO-*d*_6_): δ 192.0 (CO), 163.7 (CO), 137.8 (ArC), 135.9 (ArC), 135.6 (ArC), 133.2 (ArC), 133.0 (ArCH), 132.4 (ArC), 130.3 (ArCH), 128.7 (ArCH), 128.2 (ArCH), 127.5 (ArCH), 126.6 (ArCH), 126.6 (ArC), 126.4 (ArCH), 125.2 (ArCH), 124.6 (ArCH), 122.8 (ArCH), 54.5 (CH_2_), 51.3 (CH_2_), 42.0 (CH_3_), 36.0 (CH_2_), 31.1 (CH_2_), 28.4 (CH_2_), 28.3 (CH_2_), 27.3 (CH_2_), 23.9 (CH_2_), 22.9 (CH_2_), 22.0 (CH_2_), 13.9 (CH_3_); IR (ATR): *ν*_max_ 3675, 2924, 2854, 2698, 1641, 1494, 1466, 1397, 1331, 1269, 1236, 1201, 1143, 1086, 919, 892, 861, 815, 747, 720, 670, 557, 516, 476 cm^−1^; HRMS (+ ESI): Found *m/z* 552.2893 [M + H]^+^, C_31_H_42_N_3_O_4_S required 552.2891.

##### 3-(2-(5-Butyl-2-(octylsulfonamido)phenyl)-2-oxoacetamido)-*N*,*N*-dimethylpropan-1-aminium chloride (**13e**)

The titled compound was synthesised from 2-(5-butyl-2-(octylsulfonamido)phenyl)-*N*-(3-(dimethylamino)propyl)-2-oxoacetamide **11e** (33 mg, 0.069 mmol) and 4 M HCl/dioxane (0.10 mL, 0.40 mmol) following general synthetic procedure D. The product was obtained as a yellow sticky solid (35 mg, 99%); ^1^H NMR (600 MHz, DMSO-*d*_6_): δ 10.29 (bs, 1H, NH), 10.04 (bs, 1H, NH), 8.92 (t, *J* = 6.0 Hz, 1H, NH), 7.54–7.50 (m, 2H, ArH), 7.41 (dd, *J* = 7.7, 0.9 Hz, 1H, ArH), 3.32–3.27 (m, 2H, CH_2_), 3.15–3.06 (m, 4H, CH_2_), 2.74 (s, 6H, CH_3_), 2.61 (t, *J* = 7.8 Hz, 2H, CH_2_), 1.96–1.89 (m, 2H, CH_2_), 1.66–1.50 (m, 4H, CH_2_), 1.34–1.15 (m, 12H, CH_2_), 0.89 (t, *J* = 7.3 Hz, 3H, CH_3_), 0.84 (t, *J* = 7.3 Hz, 3H, CH_3_); ^13^C NMR (150 MHz, DMSO-*d*_6_): δ 192.6 (CO), 164.2 (CO), 138.6 (ArC), 136.4 (ArC), 134.8 (ArCH), 131.7 (ArCH), 125.6 (ArCH), 122.0 (ArCH), 54.5 (CH_2_), 51.0 (CH_2_), 42.1 (CH_3_), 35.9 (CH_2_), 33.8 (CH_2_), 32.9 (CH_2_), 31.1 (CH_2_), 28.3 (CH_2_), 28.3 (CH_2_), 27.3 (CH_2_), 23.9 (CH_2_), 22.9 (CH_2_), 22.0 (CH_2_), 32.7 (CH_2_), 13.9 (CH_3_), 13.7 (CH_3_); IR (ATR): *ν*_max_ 3379, 2953, 2922, 2855, 2674, 2360, 1670, 1578, 1524, 1496, 1465, 1443, 1400, 1334, 1253, 1179, 1153, 1086, 978, 918, 906, 874, 836, 799, 759, 677, 604, 570, 544, 516, 475, 432 cm^−1^; HRMS (+ ESI): Found *m/z* 482.3046 [M + H]^+^, C_25_H_44_N_3_O_4_S required 482.3047.

##### 3-(2-(4-(Butylsulfonamido)-[1,1’-biphenyl]-3-yl)-2-oxoacetamido)-*N*,*N*-dimethylpropan-1-aminium chloride (**14a**)

The titled compound was synthesised from 2-(4-(butylsulfonamido)-[1,1’-biphenyl]-3-yl)-*N*-(3-(dimethylamino)propyl)-2-oxoacetamide **12a** (35 mg, 0.079 mmol) and 4 M HCl/dioxane (0.10 mL, 0.40 mmol) following general synthetic procedure D. The product was obtained as a yellow sticky solid (34 mg, 91%); ^1^H NMR (600 MHz, DMSO-*d*_6_): δ 10.30 (bs, 1H, NH), 10.18 (bs, 1h, NH), 9.03 (t, *J* = 6.0 Hz, 1H, NH), 8.01–7.98 (m, 2H, ArH), 7.67–7.64 (m, 2H, ArH), 7.63–7.60 (m, 1H, ArH), 7.52–7.48 (m, 2H, ArH), 7.42–7.38 (m, 1H, ArH), 3.34–3.30 (m, 2H, CH_2_), 3.24–3.20 (m, 2H, CH_2_), 3.13–3.07 (m, 2H, CH_2_), 2.74 (s, 6H, CH_3_), 1.97–1.91 (m, 2H, CH_2_), 1.70–1.63 (m, 2H, CH_2_), 1.41–1.33 (m, 2H, CH_2_), 0.85 (t, *J* = 7.5 Hz, 3H, CH_3_); ^13^C NMR (150 MHz, DMSO-*d*_6_): δ 192.1 (CO), 163.8 (CO), 138.3 (ArC), 137.8 (ArC), 136.0 (ArC), 132.9 (ArCH), 130.2 (ArCH), 129.2 (ArCH), 127.9 (ArCH), 126.5 (ArCH), 125.9 (ArC), 122.4 (ArCH), 54.5 (CH_2_), 51.1 (CH_2_), 42.1 (CH_3_), 36.0 (CH_2_), 24.9 (CH_2_), 23.9 (CH_2_), 20.7 (CH_2_), 13.4 (CH_3_); IR (ATR): *ν*_max_ 3363, 2960, 2872, 2690, 2361, 1643, 1581, 1508, 1483, 1394, 1330, 1266, 1239, 1196, 1144, 1076, 974, 921, 843, 800, 761, 698, 681, 616, 585, 539 cm^−1^; HRMS (+ ESI): Found *m/z* 446.2109 [M + H] ^+^, C_23_H_32_N_3_O_4_S required 446.2108.

##### General Synthetic Procedure E for Quaternary Ammonium Iodide Salts

To a solution of glyoxamide derivative (1.0 equivalent) in THF (5 mL), iodomethane (2.5 equivalents) was added. The reaction mixture was stirred at room temperature for 24 h. After completion of reaction, the reaction mixture was concentrated in vacuo, washed thrice with diethyl ether and freeze-dried to afford the product.

##### *N*,*N*,*N*-Trimethyl-3-(2-(4-(octylsulfonamido)-[1,1’-biphenyl]-3-yl)-2-oxoacetamido)propan-1-aminium iodide (**15a**)

The titled compound was synthesised from *N*-(3-(Dimethylamino)propyl)-2-(4-(octylsulfonamido)-[1,1’-biphenyl]-3-yl)-2-oxoacetamide **11a** (33 mg, 0.066 mmol) and iodomethane (10 μL, 0.17 mmol) following general synthetic procedure E. The product was obtained as a yellow sticky solid (40 mg, 94%); ^1^H NMR (600 MHz, DMSO-*d*_6_): δ 10.10 (bs, 1H, NH), 8.98 (t, *J* = 5.9 Hz, 1H, NH), 8.03–7.97 (m, 2H, ArH), 7.68–7.64 (m, 2H, ArH), 7.59–7.56 (m, 1H, ArH), 7.52–7.48 (m, 2H, ArH), 7.43–7.39 (m, 1H, ArH), 3.39–3.30 (m, 4H, CH_2_), 3.21–3.16 (m, 2H, CH_2_), 3.06 (s, 9H, CH_2_), 2.02–1.95 (m, 2H, CH_2_), 1.70–1.63 (m, 2H, CH_2_), 1.39–1.15 (m, 10H, CH_3_), 0.83 (t, *J* = 7.1 Hz, 3H, CH_3_); ^13^C NMR (150 MHz, DMSO-*d*_6_): δ 191.8 (CO), 163.7 (CO), 138.3 (ArC), 132.7 (ArCH), 130.1 (ArCH), 129.2 (ArC), 129.2 (ArCH), 128.0 (ArCH), 126.8 (ArC), 126.5 (ArCH), 126.2 (ArC), 122.9 (ArCH), 63.5 (CH_2_), 52.3 (CH_3_), 51.2 (CH_2_), 35.9 (CH_2_), 31.1 (CH_2_), 28.4 (CH_2_), 28.3 (CH_2_), 27.3 (CH_2_), 22.9 (CH_2_), 22.6 (CH_2_), 22.0 (CH_2_), 13.9 (CH_3_); IR (ATR): *ν*_max_ 3396, 3032, 2925, 2854, 1647, 1582, 1508, 1483, 1394, 1330, 1265, 1195, 1140, 1076, 915, 841, 761, 698, 681, 617, 586, 564, 505 cm^−1^; HRMS (+ ESI): Found *m/z* 516.2892 [M] ^+^, C_28_H_42_N_3_O_4_S required 516.2891.

##### 3-(2-(4’-Fluoro-4-(octylsulfonamido)-[1,1’-biphenyl]-3-yl)-2-oxoacetamido)-*N*,*N*,*N*-trimethylpropan-1-aminium iodide (**15b**)

The titled compound was synthesised from *N*-(3-(dimethylamino)propyl)-2-(4’-fluoro-4-(octylsulfonamido)-[1,1’-biphenyl]-3-yl)-2-oxoacetamide **11b** (32 mg, 0.062 mmol) and iodomethane (10 μL, 0.16 mmol) following general synthetic procedure E. The product was obtained as a yellow sticky solid (39 mg, 95%); ^1^H NMR (600 MHz, DMSO-*d*_6_): δ 10.07 (bs, 1H, NH), 8.97 (t, *J* = 6.0 Hz, 1H, NH), 7.99–7.94 (m, 2H, ArH), 7.73–7.69 (m, 2H, ArH), 7.56 (d, *J* = 8.1 Hz, 1H, ArH), 7.35–7.30 (m, 2H, ArH), 3.39–3.30 (m, 4H, CH_2_), 3.17 (t, *J* = 7.8 Hz, 2H, CH_2_), 3.07 (s, 9H, CH_3_), 2.02–1.95 (m, 2H, CH_2_), 1.71–1.62 (m, 2H, CH_2_), 1.39–1.14 (m, 10H, CH_2_), 0.82 (t, *J* = 7.2 Hz, 3H, CH_3_); ^13^C NMR (150 MHz, DMSO-*d*_6_): δ 191.7 (CO), 163.6 (CO), 162.1 (ArC), 137.4 (ArC), 135.3 (ArC), 134.8 (ArC), 132.6 (ArCH), 130.0 (ArCH), 128.6 (ArCH), 127.2 (ArC), 123.1 (ArCH), 116.0 (ArCH), 63.5 (CH_2_), 52.3 (CH_3_), 51.2 (CH_2_), 35.9 (CH_2_), 31.1 (CH_2_), 28.4 (CH_2_), 28.3 (CH_2_), 27.3 (CH_2_), 22.9 (CH_2_), 22.6 (CH_2_), 22.0 (CH_2_), 13.9 (CH_3_); IR (ATR): *ν*_max_ 3410, 2925, 2855, 2359, 1647, 1603, 1516, 1488, 1400, 1331, 1262, 1221, 1196, 1141, 1100, 1013, 915, 882, 828, 725, 670, 559, 520 cm^−1^; HRMS (+ ESI): Found *m/z* 534.2798 [M] ^+^, C_28_H_41_FN_3_O_4_S required 534.2796.

##### 3-(2-(4’-Chloro-4-(octylsulfonamido)-[1,1’-biphenyl]-3-yl)-2-oxoacetamido)-*N*,*N*,*N*-trimethylpropan-1-aminium iodide (**15c**)

The titled compound was synthesised from 2-(4’-chloro-4-(octylsulfonamido)-[1,1’-biphenyl]-3-yl)-*N*-(3-(dimethylamino)propyl)-2-oxoacetamide **11c** (35 mg, 0.065 mmol) and iodomethane (10 μL, 0.16 mmol) following general synthetic procedure E. The product was obtained as a yellow sticky solid (41 mg, 93%); ^1^H NMR (600 MHz, DMSO-*d*_6_): δ 10.09 (bs, 1H, NH), 8.97 (bs, 1H, NH), 8.03–7.96 (m, 2H, ArH), 7.72–7.67 (m, 2H, ArH), 7.60–7.53 (m, 3H, ArH), 3.39–3.30 (m, 4H, CH_2_), 3.17 (t, *J* = 7.7 Hz, 2H, CH_2_), 3.07 (s, 9H, CH_3_), 2.02–1.95 (m, 2H, CH_2_), 1.70–1.63 (m, 2H, CH_2_), 1.38–1.14 (m, 10H, CH_2_), 0.82 (t, *J* = 7.2 Hz, 3H, CH_3_); ^13^C NMR (150 MHz, DMSO-*d*_6_): δ 191.6 (CO), 163.6 (CO), 137.1 (ArC), 132.8 (ArC), 132.6 (ArCH), 130.0 (ArCH), 129.2 (ArC), 129.1 (ArCH), 128.3 (ArCH), 128.0 (ArC), 127.1 (ArC), 123.1 (ArCH), 63.5 (CH_2_), 52.3 (CH_3_), 51.2 (CH_2_), 35.9 (CH_2_), 31.1 (CH_2_), 28.4 (CH_2_), 28.3 (CH_2_), 27.3 (CH_2_), 22.9 (CH_2_), 22.6 (CH_2_), 22.0 (CH_2_), 13.9 (CH_3_); IR (ATR): *ν*_max_ 3854, 3807, 3691, 3650, 3629, 2924, 2854, 2360, 1978, 1654, 1648, 1578, 1560, 1508, 1481, 1400, 1330, 1274, 1195, 1141, 1092, 1012, 915, 818, 762, 719, 697, 669, 542, 512, 460, 452, 444, 435, 428, 420 cm^−1^; HRMS (+ ESI): Found *m/z* 550.2503 [M] ^+^, C_28_H_41_ClN_3_O_4_S required 550.2501.

##### *N*,*N*,*N*-Trimethyl-3-(2-(5-(naphthalen-2-yl)-2-(octylsulfonamido)phenyl)-2-oxoacetamido)propan-1-aminium iodide (**15d**)

The titled compound was synthesised from *N*-(3-(dimethylamino)propyl)-2-(5-(naphthalen-2-yl)-2-(octylsulfonamido)phenyl)-2-oxoacetamide **11d** (35 mg, 0.063 mmol) and iodomethane (10 μL, 0.16 mmol) following general synthetic procedure E. The product was obtained as a yellow sticky solid (40 mg, 90%); ^1^H NMR (600 MHz, DMSO-*d*_6_): δ 10.12 (bs, 1H, NH), 9.00 (t, *J* = 6.1 Hz, 1H, NH), 8.24 (d, *J* = 1.5 Hz, 1H, ArH), 8.17–8.13 (m, 2H, ArH), 8.06–8.00 (m, 2H, ArH), 7.98–7.95 (m, 1H, ArH), 7.84 (dd, *J* = 8.5, 1.9 Hz, 1H, ArH), 7.62 (d, *J* = 8.4 Hz, 1H, ArH), 7.59–7.54 (m, 2H, ArH), 3.41–3.33 (m, 4H, CH_2_), 3.19 (t, *J* = 7.7 Hz, 2H, CH_2_), 3.07 (s, 9H, CH_3_), 2.03–1.97 (m, 2H, CH_2_), 1.72–1.65 (m, 2H, CH_2_), 1.38–1.30 (m, 2H, CH_2_), 1.26–1.15 (m, 8H, CH_2_), 0.82 (t, *J* = 7.1 Hz, 3H, CH_3_); ^13^C NMR (150 MHz, DMSO-*d*_6_): δ 191.7 (CO), 163.7 (CO), 137.4 (ArC), 136.3 (ArC), 135.6 (ArC), 133.2 (ArC), 132.9 (ArCH), 132.4 (ArC), 130.2 (ArCH), 128.8 (ArCH), 128.2 (ArCH), 127.6 (ArCH), 127.5 (ArC), 126.7 (ArCH), 126.5 (ArCH), 125.3 (ArCH), 124.7 (ArCH), 123.3 (ArCH), 63.5 (CH_2_), 52.3 (CH_3_), 51.2 (CH_2_), 35.9 (CH_2_), 31.1 (CH_2_), 28.4 (CH_2_), 28.3 (CH_2_), 27.3 (CH_2_), 22.9 (CH_2_), 22.6 (CH_2_), 22.0 (CH_2_), 13.9 (CH_3_); IR (ATR): *ν*_max_ 3422, 3051, 2924, 2853, 2360, 1642, 1492, 1466, 1396, 1329, 1263, 1234, 1202, 1189, 1142, 1088, 915, 892, 860, 816, 749, 720, 669, 593, 560, 516, 476, 428 cm^−1^; HRMS ( + ESI): Found *m/z* 566.3048 [M] ^+^, C_32_H_44_N_3_O_4_S required 566.3047.

##### 3-(2-(5-Butyl-2-(octylsulfonamido)phenyl)-2-oxoacetamido)-*N*,*N*,*N*-trimethylpropan-1-aminium iodide (**15e**)

The titled compound was synthesised from 2-(5-butyl-2-(octylsulfonamido)phenyl)-*N*-(3-(dimethylamino)propyl)-2-oxoacetamide **11e** (32 mg, 0.066 mmol) and iodomethane (10 μL, 0.17 mmol) following general synthetic procedure E. The product was obtained as a yellow sticky solid (35 mg, 85%); ^1^H NMR (600 MHz, DMSO-*d*_6_): δ 9.95 (bs, 1H, NH), 8.88 (t, *J* = 6.0 Hz, 1H, NH), 7.55–7.51 (m, 2H, ArH), 7.40–7.36 (m, 1H, ArH), 3.40–3.27 (m, 4H, CH_2_), 3.12–3.05 (m, 11H, CH_2_, CH_3_), 2.61 (t, *J* = 7.6 Hz, 2H, CH_2_), 2.01–1.94 (m, 2H, CH_2_), 1.66–1.59 (m, 2H, CH_2_), 1.58–1.50 (m, 2H, CH_2_), 1.35–1.15 (m, 12H, CH_2_), 0.89 (t, *J* = 7.5 Hz, 3H, CH_3_), 0.84 (t, *J* = 7.3 Hz, 3H, CH_3_); ^13^C NMR (150 MHz, DMSO-*d*_6_): δ 192.3 (CO), 164.1 (CO), 139.1 (ArC), 135.9 (ArC), 134.6 (ArCH), 131.5 (ArCH), 126.9 (ArC), 122.7 (ArCH), 63.5 (CH_2_), 52.3 (CH_3_), 50.9 (CH_2_), 35.8 (CH_2_), 33.8 (CH_2_), 32.9 (CH_2_), 31.1 (CH_2_), 28.3 (CH_2_), 28.3 (CH_2_), 27.3 (CH_2_), 22.9 (CH_2_), 22.6 (CH_2_), 22.0 (CH_2_), 21.7 (CH_2_), 13.9 (CH_3_), 13.7 (CH_3_); IR (ATR): *ν*_max_ 3424, 2954, 2925, 2855, 2358, 1670, 1577, 1529, 1492, 1466, 1397, 1328, 1233, 1178, 1141, 1074, 914, 837, 775, 723, 668, 553, 509, 424 cm^−1^; HRMS (+ ESI): Found *m/z* 496.3202 [M] ^+^, C_26_H_44_N_3_O_4_S required 496.3204.

##### 3-(2-(4-(Butylsulfonamido)-[1,1’-biphenyl]-3-yl)-2-oxoacetamido)-*N*,*N*,*N*-trimethylpropan-1-aminium iodide (**16a**)

The titled compound was synthesised from 2-(4-(butylsulfonamido)-[1,1’-biphenyl]-3-yl)-*N*-(3-(dimethylamino)propyl)-2-oxoacetamide **12a** (34 mg, 0.076 mmol) and iodomethane (12 μL, 0.19 mmol) following general synthetic procedure E. The product was obtained as a yellow sticky solid (26 mg, 58%); ^1^H NMR (600 MHz, DMSO-*d*_6_): δ 10.08 (bs, 1H, NH), 8.98 (t, *J* = 5.8 Hz, 1H, NH), 8.02–7.98 (m, 2H, ArH), 7.68–7.64 (m, 2H, ArH), 7.60–7.56 (m, 1H, ArH), 7.52–7.47 (m, 2H, ArH), 7.43–7.39 (m, 1H, ArH), 3.39–3.30 (m, 4H, CH_2_), 3.21–3.16 (m, 2H, CH_2_), 3.07 (s, 9H, CH_2_), 2.02–1.95 (m, 2H, CH_2_), 1.70–1.63 (m, 2H, CH_2_), 1.41–1.33 (m, 2H, CH_3_), 0.85 (t, *J* = 7.4 Hz, 3H, CH_3_); ^13^C NMR (150 MHz, DMSO-*d*_6_): δ 191.8 (CO), 163.7 (CO), 138.3 (ArC), 132.7 (ArCH), 130.1 (ArCH), 129.2 (ArC), 129.2 (ArCH), 127.9 (ArCH), 126.9 (ArC), 126.5 (ArCH), 126.3 (ArC), 123.0 (ArCH), 63.5 (CH_2_), 52.3 (CH_3_), 51.0 (CH_2_), 35.9 (CH_2_), 25.0 (CH_2_), 22.6 (CH_2_), 20.7 (CH_2_), 13.5 (CH_3_); IR (ATR): *ν*_max_ 3195, 3032, 2959, 2872, 2359, 1979, 1644, 1582, 1508, 1482, 1452, 1394, 1329, 1268, 1195, 1140, 1076, 920, 842, 762, 699, 681, 616, 584, 536, 428 cm^−1^; HRMS (+ ESI): Found *m/z* 460.2264 [M] ^+^, C_24_H_34_N_3_O_4_S required 460.2265.

##### General Synthetic Procedure F for Boc-Protected Glyoxamide Derivatives

To a solution of *N*-sulfonylisatin (1.0 equivalent) in dichloromethane (10 mL), *N*-Boc-1,3-propandiamine (1.0 equivalent) in dichloromethane (5 mL) was added dropwise with stirring at 0 °C. The reaction mixture was stirred at room temperature for 6 h. After completion of the reaction, water was added to the reaction mixture and the product was extracted into dichloromethane (3 × 30 mL), washed with brine, dried over anhydrous sodium sulphate and concentrated in vacuo to afford the product.

##### *tert-*Butyl (3-(2-(4-(octylsulfonamido)-[1,1’-biphenyl]-3-yl)-2-oxoacetamido)propyl)carbamate (**18a**)

The titled compound was synthesised from 1-(octylsulfonyl)-5-phenylindoline-2,3-dione **8a** (0.32 g, 0.81 mmol) and *N*-Boc-1,3-propandiamine (0.15 g, 0.82 mmol) following general synthetic procedure F. The product was obtained as a yellow solid (0.45 g, 97%); mp 127.3–127.4 °C; ^1^H NMR (600 MHz, CDCl_3_): δ 10.48 (bs, 1H, NH), 8.75 (s, 1H, ArH), 7.88–7.81 (m, 2H, ArH), 7.71 (bs, 1H, NH), 7.59–7.55 (m, 2H, ArH), 7.47–7.43 (m, 2H, ArH), 7.39–7.35 (m, 1H, ArH), 4.81 (bs, 1H, NH), 3.48 (q, *J* = 6.4 Hz, 2H, CH_2_), 3.27–3.21 (m, 2H, CH_2_), 3.21–3.16 (m, 2H, CH_2_), 1.85–1.78 (m, 2H, CH_2_), 1.78–1.73 (m, 2H, CH_2_), 1.44 (s, 9H, CH_3_), 1.42–1.34 (m, 2H, CH_2_), 1.30–1.16 (m, 8H, CH_2_), 0.85 (t, *J* = 7.2 Hz, 3H, CH_3_); ^13^C NMR (150 MHz, CDCl_3_): δ 191.5 (CO), 162.9 (CO), 156.9 (CO), 141.1 (ArC), 139.1 (ArC), 135.7 (ArC), 135.1 (ArCH), 133.7 (ArCH), 129.2 (ArCH), 127.9 (ArCH), 126.9 (ArCH), 119.3 (ArC), 118.5 (ArCH), 79.9 (C), 52.8 (CH_2_), 37.3 (CH_2_), 36.4 (CH_2_), 31.8 (CH_2_), 30.2 (CH_2_), 29.1 (CH_2_), 29.0 (CH_2_), 28.5 (CH_3_), 28.2 (CH_2_), 23.5 (CH_2_), 22.7 (CH_2_), 14.2 (CH_3_); IR (ATR): *ν*_max_ 3352, 3310, 2919, 2359, 1682, 1666, 1582, 1518, 1483, 1443, 1386, 1364, 1346, 1325, 1294, 1246, 1197, 1171, 1155, 1124, 1069, 1041, 1011, 977, 968, 906, 894, 851, 830, 804, 760, 741, 714, 684, 639, 609, 554, 537, 491, 453, 423 cm^−1^; HRMS (+ ESI): Found *m/z* 596.2764 [M + Na] ^+^, C_30_H_43_N_3_O_6_SNa required 596.2764.

##### *tert-*Butyl (3-(2-(4’-fluoro-4-(octylsulfonamido)-[1,1’-biphenyl]-3-yl)-2-oxoacetamido)propyl) carbamate (**18b**)

The titled compound was synthesised from 5-(4-fluorophenyl)-1-(octylsulfonyl)indoline-2,3-dione **8b** (0.28 g, 0.67 mmol) and *N*-Boc-1,3-propandiamine (0.12 g, 0.67 mmol) following general synthetic procedure F. The product was obtained as a yellow solid (0.38 g, 96%); mp 138.0–140.3 °C; ^1^H NMR (400 MHz, CDCl_3_): δ 10.46 (bs, 1H, NH), 8.72 (s, 1H, ArH), 7.85 (d, *J* = 8.7 Hz, 1H, ArH), 7.81–7.69 (m, 2H, NH, ArH), 7.56–7.50 (m, 2H, ArH), 7.17–7.09 (m, 2H, ArH), 4.81 (t, *J* = 5.9 Hz, 1H, NH), 3.47 (q, *J* = 6.4 Hz, 2H, CH_2_), 3.29–3.14 (m, 4H, CH_2_), 1.86–1.71 (m, 4H, CH_2_), 1.44 (s, 9H, CH_3_), 1.42–1.33 (m, 2H, CH_2_), 1.31–1.18 (m, 8H, CH_2_), 0.85 (t, *J* = 7.1 Hz, 3H, CH_3_); ^13^C NMR (100 MHz, CDCl_3_): δ 191.3 (CO), 162.8 (CO), 162.8 (ArC), 156.9 (CO), 141.1 (ArC), 135.2 (ArC), 134.9 (ArCH), 134.7 (ArC), 133.5 (ArCH), 128.6 (ArCH), 119.3 (ArC), 118.6 (ArCH), 116.1 (ArCH), 79.9 (C), 52.8 (CH_2_), 37.3 (CH_2_), 36.4 (CH_2_), 31.8 (CH_2_), 30.2 (CH_2_), 29.1 (CH_2_), 29.0 (CH_2_), 28.5 (CH_3_), 28.2 (CH_2_), 23.5 (CH_2_), 22.7 (CH_2_), 14.2 (CH_3_); IR (ATR): *ν*_max_ 3357, 3313, 2923, 2856, 2303, 1887, 1680, 1645, 1519, 1487, 1388, 1344, 1247, 1155, 1070, 1011, 976, 906, 854, 826, 771 cm^−1^; HRMS (+ ESI): Found *m/z* 614.2670 [M + Na] ^+^, C_30_H_42_FN_3_O_6_SNa required 614.2671.

##### *tert*-Butyl (3-(2-(4’-chloro-4-(octylsulfonamido)-[1,1’-biphenyl]-3-yl)-2-oxoacetamido)propyl) carbamate (**18c**)

The titled compound was synthesised from 5-(4-chlorophenyl)-1-(octylsulfonyl)indoline-2,3-dione **8c** (0.30 g, 0.69 mmol) and *N*-Boc-1,3-propandiamine (0.13 g, 0.71 mmol) following general synthetic procedure F. The product was obtained as a yellow solid (0.41 g, 97%); mp 134.7–135.1 °C; ^1^H NMR (600 MHz, CDCl_3_): δ 10.49 (bs, 1H, NH), 8.74 (s, 1H, ArH), 7.85 (d, *J* = 8.7 Hz, 1H, ArH), 7.78 (dd, *J* = 8.7, 2.3 Hz, 1H, ArH), 7.77 (bs, 1H, NH), 7.51–7.48 (m, 2H, ArH), 7.43–7.40 (m, 2H, ArH), 4.81 (bs, 1H, NH), 3.47 (q, *J* = 6.4 Hz, 2H, CH_2_), 3.27–3.22 (m, 2H, CH_2_), 3.20–3.16 (m, 2H, CH_2_), 1.84–1.72 (m, 4H, CH_2_), 1.44 (s, 9H, CH_3_), 1.41–1.34 (m, 2H, CH_2_), 1.29–1.18 (m, 8H, CH_2_), 0.85 (t, *J* = 7.2 Hz, 3H, CH_3_); ^13^C NMR (150 MHz, CDCl_3_): δ 191.3 (CO), 162.7 (CO), 156.9 (CO), 141.3 (ArC), 137.5 (ArC), 134.8 (ArCH), 134.4 (ArC), 134.1 (ArC), 133.5 (ArCH), 129.3 (ArCH), 128.2 (ArCH), 119.3 (ArC), 118.6 (ArCH), 79.9 (C), 52.8 (CH_2_), 37.3 (CH_2_), 36.4 (CH_2_), 31.8 (CH_2_), 30.2 (CH_2_), 29.1 (CH_2_), 29.0 (CH_2_), 28.5 (CH_3_), 28.2 (CH_2_), 23.5 (CH_2_), 22.7 (CH_2_), 14.2 (CH_3_); IR (ATR): *ν*_max_ 3352, 3311, 2922, 2855, 2359, 1979, 1682, 1665, 1643, 1581, 1519, 1483, 1443, 1388, 1364, 1345, 1246, 1197, 1155, 1138, 1093, 1070, 1041, 1010, 975, 907, 895, 882, 864, 817, 759, 741, 714, 685, 643, 609, 555, 537, 492, 471, 418 cm^−1^; HRMS (+ ESI): Found *m/z* 630.2379 [M + Na] ^+^, C_30_H_42_ClN_3_O_6_SNa required 630.2375.

##### *tert-*Butyl (3-(2-(5-(naphthalen-2-yl)-2-(octylsulfonamido)phenyl)-2-oxoacetamido)propyl) carbamate (**18d**)

The titled compound was synthesised from 5-(naphthalen-2-yl)-1-(octylsulfonyl)indoline-2,3-dione **8d** (0.31 g, 0.70 mmol) and *N*-Boc-1,3-propandiamine (0.13 g, 0.70 mmol) following general synthetic procedure F. The product was obtained as a yellow solid (0.41 g, 95%); mp 78.9–80.0 °C; ^1^H NMR (600 MHz, CDCl_3_): δ 10.51 (bs, 1H, NH), 8.87 (s, 1H, ArH), 8.01 (s, 1H, ArH), 7.99–7.83 (m, 5H, ArH), 7.79–7.67 (m, 2H, NH, ArH), 7.55–7.46 (m, 2H, ArH), 4.84 (bs, 1H, NH), 3.49 (q, *J* = 6.5 Hz, 2H, CH_2_), 3.30–3.16 (m, 4H, CH_2_), 1.88–1.72 (m, 4H, CH_2_), 1.44 (s, 9H, CH_3_), 1.46–1.34 (m, 2H, CH_2_), 1.31–1.16 (m, 8H, CH_2_), 0.85 (t, *J* = 7.1 Hz, 3H, CH_3_); ^13^C NMR (150 MHz, CDCl_3_): δ 191.5 (CO), 162.9 (CO), 156.9 (CO), 141.1 (ArC), 136.3 (ArC), 135.6 (ArC), 135.4 (ArCH), 133.8 (ArCH), 133.7 (ArC), 132.9 (ArC), 128.9 (ArCH), 128.4 (ArCH), 127.8 (ArCH), 126.7 (ArCH), 126.4 (ArCH), 125.7 (ArCH), 125.0 (ArCH), 119.4 (ArC), 118.6 (ArCH), 79.9 (C), 52.8 (CH_2_), 37.3 (CH_2_), 36.5 (CH_2_), 31.8 (CH_2_), 30.2 (CH_2_), 29.1 (CH_2_), 29.0 (CH_2_), 28.5 (CH_3_), 28.2 (CH_2_), 23.6 (CH_2_), 22.7 (CH_2_), 14.2 (CH_3_); IR (ATR): *ν*_max_ 3346, 3055, 2925, 2855, 2285, 2081, 1911, 1683, 1636, 1572, 1497, 1466, 1393, 1365, 1336, 1247, 1140, 1012, 917, 890, 813, 747 cm^−1^; HRMS (+ ESI): Found *m/z* 646.2919 [M + Na] ^+^, C_34_H_45_N_3_O_6_SNa required 646.2921.

##### General Synthetic Procedure G for Aminoglyoxamides

To a solution of Boc-protected glyoxamide (1.0 equivalent) in dichloromethane (10 mL), 4 M HCl/dioxane (3 mL) was added. The reaction mixture was stirred at room temperature for 6 h. After completion of reaction, the reaction mixture was concentrated in vacuo, washed thrice with diethyl ether and dried under high vacuum to afford the product.

##### *N*-(3-Aminopropyl)-2-(4-(octylsulfonamido)-[1,1’-biphenyl]-3-yl)-2-oxoacetamide hydrochloride (**19a**)

The titled compound was synthesised from *tert*-butyl (3-(2-(4-(octylsulfonamido)-[1,1’-biphenyl]-3-yl)-2-oxoacetamido)propyl)carbamate **18a** (0.35 g, 0.61 mmol) following general synthetic procedure G. The product was obtained as a yellow solid (0.20 g, 64%); mp 126.2–128.7 °C; ^1^H NMR (400 MHz, DMSO-*d*_6_): δ 10.20 (bs, 1H, NH), 9.05 (t, *J* = 6.0 Hz, 1H, NH), 8.19–7.91 (m, 5H, NH, ArH), 7.66–7.61 (m, 3H, ArH), 7.51 (t, *J* = 7.8 Hz, 2H, ArH), 7.41 (t, *J* = 7.2 Hz, 1H, ArH), 3.38–3.29 (m, 2H, CH_2_), 3.29–3.22 (m, 2H, CH_2_), 2.91–2.82 (m, 2H, CH_2_), 1.89–1.79 (m, 2H, CH_2_), 1.72–1.62 (m, 2H, CH_2_), 1.39–1.14 (m, 10H, CH_2_), 0.82 (t, *J* = 7.0 Hz, 3H, CH_3_); ^13^C NMR (100 MHz, DMSO-*d*_6_): δ 192.4 (CO), 163.8 (CO), 138.3 (ArC), 138.2 (ArC), 135.6 (ArC), 133.2 (ArCH), 130.5 (ArCH), 129.2 (ArCH), 127.9 (ArCH), 126.4 (ArCH), 124.5 (ArC), 121.7 (ArCH), 51.4 (CH_2_), 36.7 (CH_2_), 35.9 (CH_2_), 31.1 (CH_2_), 28.4 (CH_2_), 28.3 (CH_2_), 27.3 (CH_2_), 26.9 (CH_2_), 22.9 (CH_2_), 22.0 (CH_2_), 14.2 (CH_3_); IR (ATR): *ν*_max_ 3031, 2921, 2853, 2045, 1961, 1635, 1509, 1482, 1393, 1335, 1264, 1196, 1138, 1025, 917, 838, 759, 696 cm^−1^; HRMS (+ ESI): Found *m/z* 474.2419 [M + H] ^+^, C_25_H_36_N_3_O_4_S required 474.2421.

##### *N*-(3-Aminopropyl)-2-(4’-fluoro-4-(octylsulfonamido)-[1,1’-biphenyl]-3-yl)-2-oxoacetamide hydrochloride (**19b**)

The titled compound was synthesised from *tert-*butyl (3-(2-(4’-fluoro-4-(octylsulfonamido)-[1,1’-biphenyl]-3-yl)-2-oxoacetamido)propyl) carbamate **18b** (0.34 g, 0.58 mmol) following general synthetic procedure G. The product was obtained as a yellow solid (0.27 g, 90%); mp 155.2–157.8 °C; ^1^H NMR (400 MHz, DMSO-*d*_6_): δ 10.19 (bs, 1H, NH), 9.05 (t, *J* = 5.9 Hz, 1H, NH), 8.14–7.93 (m, 5H, NH, ArH), 7.72–7.65 (m, 2H, ArH), 7.65–7.59 (m, 1H, ArH), 7.34 (t, *J* = 8.9 Hz, 2H, ArH), 3.37–3.29 (m, 2H, CH_2_), 3.28–3.21 (m, 2H, CH_2_), 2.92–2.80 (m, 2H, CH_2_), 1.90–1.79 (m, 2H, CH_2_), 1.72–1.61 (m, 2H, CH_2_), 1.38–1.14 (m, 10H, CH_2_), 0.82 (t, *J* = 7.1 Hz, 3H, CH_3_); ^13^C NMR (100 MHz, DMSO-*d*_6_): δ 192.3 (CO), 163.7 (CO), 162.1 (ArC), 138.2 (ArC), 134.7 (ArC), 134.6 (ArC), 133.0 (ArCH), 130.3 (ArCH), 128.5 (ArCH), 124.7 (ArC), 121.8 (ArCH), 116.0 (ArCH), 51.4 (CH_2_), 36.6 (CH_2_), 35.9 (CH_2_), 31.1 (CH_2_), 28.3 (CH_2_), 28.3 (CH_2_), 27.3 (CH_2_), 26.9 (CH_2_), 22.9 (CH_2_), 22.0 (CH_2_), 13.9 (CH_3_); IR (ATR): *ν*_max_ 3365, 3161, 2970, 2925, 2753, 2611, 2494, 2343, 2058, 1919, 1633, 1600, 1528, 1491, 1393, 1334, 1259, 1203, 1140, 1083, 1021, 919, 868, 823, 761 cm^−1^; HRMS (+ ESI): Found *m/z* 492.2325 [M + H] ^+^, C_25_H_35_FN_3_O_4_S required 492.2327.

##### *N*-(3-Aminopropyl)-2-(4’-chloro-4-(octylsulfonamido)-[1,1’-biphenyl]-3-yl)-2-oxoacetamide hydrochloride (**19c**)

The titled compound was synthesised from *tert-*butyl (3-(2-(4’-chloro-4-(octylsulfonamido)-[1,1’-biphenyl]-3-yl)-2-oxoacetamido)propyl) carbamate **18c** (0.38 g, 0.62 mmol) following general synthetic procedure G. The product was obtained as a yellow solid (0.28 g, 83%); mp 118.8–119.1 °C; ^1^H NMR (400 MHz, DMSO-*d*_6_): δ 10.19 (bs, 1H, NH), 9.04 (t, *J* = 6.0 Hz, 1H, NH), 8.12–7.88 (m, 5H, NH, ArH), 7.71–7.60 (m, 3H, ArH), 7.57 (d, *J* = 8.5 Hz, 2H, ArH), 3.38–3.29 (m, 2H, CH_2_), 3.28–3.21 (m, 2H, CH_2_), 2.92–2.81 (m, 2H, CH_2_), 1.89–1.79 (m, 2H, CH_2_), 1.72–1.61 (m, 2H, CH_2_), 1.39–1.13 (m, 10H, CH_2_), 0.82 (t, *J* = 7.1 Hz, 3H, CH_3_); ^13^C NMR (100 MHz, DMSO-*d*_6_): δ 192.2 (CO), 163.7 (CO), 138.4 (ArC), 137.0 (ArC), 134.3 (ArC), 133.0 (ArCH), 132.8 (ArC), 130.4 (ArCH), 129.2 (ArCH), 128.3 (ArCH), 124.8 (ArC), 121.9 (ArCH), 51.5 (CH_2_), 36.7 (CH_2_), 35.9 (CH_2_), 31.1 (CH_2_), 28.4 (CH_2_), 28.3 (CH_2_), 27.3 (CH_2_), 26.9 (CH_2_), 22.9 (CH_2_), 22.0 (CH_2_), 13.9 (CH_3_); IR (ATR): *ν*_max_ 3808, 2924, 2855, 2360, 2038, 1657, 1636, 1578, 1527, 1509, 1483, 1397, 1340, 1270, 1201, 1139, 1095, 1046, 1010, 919, 875, 815, 772, 700, 668, 596, 562, 494 cm^−1^; HRMS (+ ESI): Found *m/z* 508.2033 [M + H] ^+^, C_25_H_35_ClN_3_O_4_S required 508.2031.

##### *N*-(3-Aminopropyl)-2-(5-(naphthalen-2-yl)-2-(octylsulfonamido)phenyl)-2-oxoacetamide hydrochloride (**19d**)

The titled compound was synthesised from *tert*-Butyl (3-(2-(5-(naphthalen-2-yl)-2-(octylsulfonamido)phenyl)-2-oxoacetamido)propyl) carbamate **18d** (0.37 g, 0.59 mmol) following general synthetic procedure G. The product was obtained as a yellow solid (0.22 g, 67%); mp 85.2–87.3 °C; ^1^H NMR (400 MHz, DMSO-*d*_6_): δ 10.23 (bs, 1H, NH), 9.07 (t, *J* = 5.9 Hz, 1H, NH), 8.22 (s, 1H, ArH), 8.18–8.12 (m, 2H, ArH), 8.10–7.93 (m, 6H, NH, ArH), 7.81 (dd, *J* = 8.6, 1.8 Hz, 1H, ArH), 7.68 (dd, *J* = 6.4, 2.8 Hz, 1H, ArH),7.60–7.52 (m, 2H, ArH), 3.40–3.31 (m, 2H, CH_2_), 3.29–3.22 (m, 2H, CH_2_), 2.92–2.82 (m, 2H, CH_2_), 1.92–1.82 (m, 2H, CH_2_), 1.74–1.63 (m, 2H, CH_2_), 1.39–1.14 (m, 10H, CH_2_), 0.81 (t, *J* = 7.0 Hz, 3H, CH_3_); ^13^C NMR (100 MHz, DMSO-*d*_6_): δ 192.3 (CO), 163.8 (CO), 138.2 (ArC), 135.6 (ArC), 133.2 (ArC), 133.2 (ArCH), 132.3 (ArC), 130.6 (ArCH), 128.9 (ArCH), 128.3 (ArCH), 127.6 (ArC), 127.6 (ArCH), 126.6 (ArCH), 126.4 (ArCH), 125.2 (ArC), 125.2 (ArCH), 124.6 (ArCH), 122.1 (ArCH), 51.4 (CH_2_), 36.7 (CH_2_), 35.9 (CH_2_), 31.1 (CH_2_), 28.4 (CH_2_), 28.3 (CH_2_), 27.3 (CH_2_), 26.9 (CH_2_), 22.9 (CH_2_), 22.0 (CH_2_), 13.9 (CH_3_); IR (ATR): *ν*_max_3312, 3176, 2921, 2852, 2321, 2050, 1922, 1635, 1494, 1463, 1396, 1333, 1264, 1200, 1138, 1016, 916, 812, 747 cm^−1^; HRMS ( + ESI): Found *m/z* 524.2579 [M + H] ^+^, C_29_H_38_N_3_O_4_S required 524.2578.

##### General Synthetic Procedure H for Boc-Protected Guanidine Glyoxamides

To a solution of aminoglyoxamides (1.0 equivalent) and *N*,*N*’-di-Boc-1*H*-pyrazole-1- carboxamidine (1.3 equivalents) in acetonitrile (10 mL), triethylamine (2.5 equivalents) in acetonitrile (5 mL) was added dropwise with stirring at 0 °C under nitrogen atmosphere. The reaction mixture was stirred at room temperature for 18 h. After completion of the reaction, the reaction mixture was concentrated in vacuo. The product was purified by flash chromatography on silica using ethyl acetate/n-hexane (1:4) as eluent to afford the product.

##### (*E*)-1-*tert*-Butyl-*N*-(*N*’-((*tert*-butyloxidanyl)carbonyl)-*N*-(3-(2-(4-(octylsulfonamido)-[1,1’-biphenyl]-3-yl)-2-oxoacetamido)propyl)carbamimidoyl)-1-oxidanecarboxamide (**21a**)

The titled compound was synthesised from *N*-(3-aminopropyl)-2-(4-(octylsulfonamido)-[1,1’-biphenyl]-3-yl)-2-oxoacetamide hydrochloride **19a** (0.16 g, 0.30 mmol), *N*,*N*’-di-Boc-1*H*-pyrazole-1-carboxamidine (0.14 g, 0.45 mmol) and triethylamine (0.12 mL, 0.83 mmol) following general synthetic procedure H. The product was obtained as a yellow solid (68 mg, 31%); mp 69.9–70.1 °C; ^1^H NMR (400 MHz, CDCl_3_): δ 11.47 (bs, 1H, NH), 10.62 (bs, 1H, NH), 8.68–8.50 (m, 3H, NH, ArH), 7.89–7.80 (m, 2H, ArH), 7.57 (d, *J* = 7.5 Hz, 2H, ArH), 7.45 (t, *J* = 7.9 Hz, 2H, ArH), 7.36 (t, *J* = 7.1 Hz, 1H, ArH), 3.62–3.52 (m, 2H, CH_2_), 3.46 (q, *J* = 6.4 Hz, 2H, CH_2_), 3.21–3.13 (m, 2H, CH_2_), 1.87–1.76 (m, 4H, CH_2_), 1.50 (s, 9H, CH_3_), 1.38 (s, 9H, CH_3_), 1.43–1.18 (m, 10H, CH_2_), 0.85 (t, *J* = 7.1 Hz, 3H, CH_3_); ^13^C NMR (150 MHz, CDCl_3_): δ 192.6 (CO), 163.7 (CO), 163.0 (CN), 157.4 (CO), 153.3 (CO), 141.2 (ArC), 139.1 (ArC), 135.6 (ArC), 135.0 (ArCH), 133.5 (ArCH), 129.1 (ArCH), 127.9 (ArCH), 127.0 (ArCH), 119.0 (ArC), 118.4 (ArCH), 83.8 (C), 79.9 (C), 52.8 (CH_2_), 37.3 (CH_2_), 35.9 (CH_2_), 31.8 (CH_2_), 30.1 (CH_2_), 29.1 (CH_2_), 29.0 (CH_2_), 28.4 (CH_2_), 28.3 (CH_3_), 28.2 (CH_3_), 23.6 (CH_2_), 22.7 (CH_2_), 14.2 (CH_3_); IR (ATR): *ν*_max_ 3323, 2928, 2360, 1979, 1720, 1638, 1571, 1508, 1483, 1450, 1410, 1366, 1328, 1284, 1228, 1195, 1131, 1051, 1026, 978, 907, 855, 806, 760, 697, 681, 616, 587, 562, 537, 418 cm^−1^; HRMS (+ ESI): Found *m/z* 716.3684 [M + H] ^+^, C_36_H_54_N_5_O_8_S required 716.3688.

##### (*E*)-1-*tert*-Butyl-*N*-(*N*’-((*tert*-butyloxidanyl)carbonyl)-*N*-(3-(2-(4’-fluoro-4-(octylsulfonamido)-[1,1’-biphenyl]-3-yl)-2-oxoacetamido)propyl)carbamimidoyl)-1-oxidanecarboxamide (**21b**)

The titled compound was synthesised from *N*-(3-aminopropyl)-2-(4’-fluoro-4-(octylsulfonamido)-[1,1’-biphenyl]-3-yl)-2-oxoacetamide hydrochloride **19b** (0.24 g, 0.45 mmol), *N*,*N*’-di-Boc-1*H*-pyrazole-1-carboxamidine (0.20 g, 0.64 mmol) and triethylamine (0.16 mL, 1.11 mmol) following general synthetic procedure H. The product was obtained as a yellow solid (0.25, 77%); mp 62.8–65.0 °C; ^1^H NMR (400 MHz, CDCl_3_): δ 11.47 (bs, 1H, NH), 10.61 (bs, 1H, NH), 8.64 (t, *J* = 6.0 Hz, 1H, NH), 8.58 (bs, 1H, NH), 8.52 (d, *J* = 2.1 Hz, 1H, ArH), 7.85 (t, *J* = 7.9 Hz, 1H, ArH), 7.76 (dd, *J* = 8.7, 2.2 Hz, 1H, ArH), 7.56–7.49 (m, 2H, ArH), 7.13 (t, *J* = 8.6 Hz, 2H, ArH), 3.63–3.53 (m, 2H, CH_2_), 3.46 (q, *J* = 6.2 Hz, 2H, CH_2_), 3.20–3.13 (m, 2H, CH_2_), 1.86–1.76 (m, 4H, CH_2_), 1.50 (s, 9H, CH_3_), 1.38 (s, 9H, CH_3_), 1.43–1.16 (m, 10H, CH_2_), 0.85 (t, *J* = 7.1 Hz, 3H, CH_3_); ^13^C NMR (100 MHz, CDCl_3_): δ 192.3 (CO), 163.6 (CO), 162.8 (ArC), 162.7 (CN), 157.3 (CO), 153.3 (CO), 141.1 (ArC), 135.3 (ArC), 134.7 (ArCH), 134.7 (ArC), 133.3 (ArCH), 128.6 (ArCH), 119.1 (ArC), 118.5 (ArCH), 116.1 (ArCH), 83.9 (C), 80.1 (C), 52.8 (CH_2_), 37.3 (CH_2_), 35.9 (CH_2_), 31.8 (CH_2_), 30.0 (CH_2_), 29.1 (CH_2_), 29.0 (CH_2_), 28.4 (CH_2_), 28.2 (CH_3_), 28.2 (CH_3_), 23.5 (CH_2_), 22.7 (CH_2_), 14.2 (CH_3_); IR (ATR): *ν*_max_ 3324, 2926, 2855, 2322, 1890, 1720, 1638, 1570, 1487, 1408, 1326, 1258, 1225, 1128, 1049, 906, 858, 800, 731, 669 cm^−1^; HRMS ( + ESI): Found *m/z* 734.3594 [M + H] ^+^, C_36_H_53_FN_5_O_8_S required 734.3593.

##### (*E*)-1-*tert*-Butyl-*N*-(*N*’-((*tert*-butyloxidanyl)carbonyl)-*N*-(3-(2-(4’-chloro-4-(octylsulfonamido)-[1,1’-biphenyl]-3-yl)-2-oxoacetamido)propyl)carbamimidoyl)-1-oxidanecarboxamide (**21c**)

The titled compound was synthesised from *N*-(3-aminopropyl)-2-(4’-chloro-4-(octylsulfonamido)-[1,1’-biphenyl]-3-yl)-2-oxoacetamide hydrochloride **19c** (0.16 g, 0.29 mmol), *N*,*N*’-di-Boc-1*H*-pyrazole-1-carboxamidine (0.12 g, 0.38 mmol) and triethylamine (0.10 mL, 0.72 mmol) following general synthetic procedure H. The product was obtained as a yellow solid (0.14 g, 64%); mp 77.1–77.4 °C; ^1^H NMR (400 MHz, CDCl_3_): δ 11.47 (bs, 1H, NH), 10.63 (bs, 1H, NH), 8.65 (t, *J* = 6.3 Hz, 1H, NH), 8.61–8.51 (m, 2H, NH, ArH), 7.86 (d, *J* = 8.7 Hz, 1H, ArH), 7.77 (dd, *J* = 8.7, 2.2 Hz, 1H, ArH), 7.52–7.47 (m, 2H, ArH), 7.43–7.39 (m, 2H, ArH), 3.62–3.52 (m, 2H, CH_2_), 3.46 (q, *J* = 6.3 Hz, 2H, CH_2_), 3.20–3.13 (m, 2H, CH_2_), 1.86–1.75 (m, 4H, CH_2_), 1.50 (s, 9H, CH_3_), 1.38 (s, 9H, CH_3_), 1.43–1.18 (m, 10H, CH_2_), 0.85 (t, *J* = 7.1 Hz, 3H, CH_3_); ^13^C NMR (150 MHz, CDCl_3_): δ 192.3 (CO), 163.5 (CO), 163.1 (CN), 157.5 (CO), 153.4 (CO), 141.5 (ArC), 137.6 (ArC), 134.7 (ArCH), 134.3 (ArC), 134.1 (ArC), 133.4 (ArCH), 129.3 (ArCH), 128.2 (ArCH), 118.9 (ArC), 118.4 (ArCH), 83.8 (C), 79.8 (C), 52.9 (CH_2_), 37.2 (CH_2_), 35.9 (CH_2_), 31.8 (CH_2_), 30.1 (CH_2_), 29.1 (CH_2_), 29.0 (CH_2_), 28.4 (CH_2_), 28.3 (CH_3_), 28.2 (CH_3_), 23.6 (CH_2_), 22.7 (CH_2_), 14.2 (CH_3_); IR (ATR): *ν*_max_ 3322, 2929, 1720, 1637, 1577, 1507, 1482, 1409, 1367, 1328, 1285, 1253, 1228, 1195, 1131, 1094, 1050, 1026, 1012, 979, 907, 856, 817, 770, 698, 657, 592, 562, 489, 419 cm^−1^; HRMS (+ ESI): Found *m/z* 772.3120 [M + Na] ^+^, C_36_H_52_ClN_5_O_8_SNa required 772.3117.

##### (*E*)-1-*tert*-Butyl-*N*-(*N*’-((*tert-*butyloxidanyl)carbonyl)-*N*-(3-(2-(5-(naphthalen-2-yl)-2-(octylsulfonamido)phenyl)-2-oxoacetamido)propyl)carbamimidoyl)-1-oxidanecarboxamide (**21d**)

The titled compound was synthesised from *N*-(3-Aminopropyl)-2-(5-(naphthalen-2-yl)-2-(octylsulfonamido)phenyl)-2-oxoacetamide hydrochloride **19d** (0.18 g, 0.33 mmol), *N*,*N*’-di-Boc-1*H*-pyrazole-1-carboxamidine (0.13 g, 0.40 mmol) and triethylamine (0.12 mL, 0.83 mmol) following general synthetic procedure H. The product was obtained as a yellow solid (0.13 g, 52%); mp 71.6–73.5 °C; ^1^H NMR (400 MHz, CDCl_3_): δ 11.47 (bs, 1H, NH), 10.66 (bs, 1H, NH), 8.71–8.53 (m, 3H, NH, ArH), 8.01 (s, 1H, ArH), 7.98–7.84 (m, 5H, ArH), 7.71 (dd, *J* = 8.5, 1.7 Hz, 1H, ArH), 7.55–7.46 (m, 2H, ArH), 3.64–3.54 (m, 2H, CH_2_), 3.48 (q, *J* = 6.2 Hz, 2H, CH_2_), 3.23–3.15 (m, 2H, CH_2_), 1.89–1.76 (m, 4H, CH_2_), 1.50 (s, 9H, CH_3_), 1.39 (s, 9H, CH_3_), 1.44–1.17 (m, 10H, CH_2_), 0.85 (t, *J* = 7.1 Hz, 3H, CH_3_); ^13^C NMR (100 MHz, CDCl_3_): δ 192.6 (CO), 163.8 (CO), 162.8 (CN), 157.3 (CO), 153.3 (CO), 141.2 (ArC), 136.4 (ArC), 135.6 (ArC), 135.2 (ArC), 133.7 (ArCH), 133.7 (ArCH), 132.9 (ArC), 128.9 (ArCH), 128.3 (ArCH), 127.8 (ArCH), 126.7 (ArCH), 126.4 (ArCH), 125.7 (ArCH), 125.1 (ArCH), 119.2 (ArC), 118.5 (ArCH), 83.9 (C), 80.2 (C), 52.8 (CH_2_), 37.5 (CH_2_), 35.9 (CH_2_), 31.8 (CH_2_), 30.0 (CH_2_), 29.1 (CH_2_), 29.0 (CH_2_), 28.3 (CH_2_), 28.3 (CH_3_), 28.2 (CH_3_), 23.6 (CH_2_), 22.7 (CH_2_), 14.2 (CH_3_); IR (ATR): *ν*_max_ 3323, 2928, 2360, 1979, 1720, 1638, 1571, 1508, 1483, 1450, 1410, 1366, 1328, 1284, 1228, 1195, 1131, 1051, 1026, 978, 907, 855, 806, 760, 697, 681, 616, 587, 562, 537, 418 cm^−1^; HRMS (+ ESI): Found *m/z* 766.3845 [M + H] ^+^, C_40_H_56_N_5_O_8_S required 766.3844.

##### General Synthetic Procedure I for Guanidinium Hydrochloride Salts

To a solution of Boc-protected guanidine glyoxamide (1.0 equivalent) in dichloromethane (1 mL), trifluoroacetic acid (1 mL) was added. The reaction mixture was stirred at room temperature for 3 h. After completion of the reaction, the reaction mixture was concentrated in vacuo and washed thrice with diethyl ether. To the residue in dichloromethane (1 mL), 4 M HCl/dioxane (1 mL) was added. The reaction mixture was stirred at room temperature for 30 min. After completion of reaction, the reaction mixture was concentrated in vacuo, washed thrice with diethyl ether and freeze-dried to afford the product.

##### *N*-(3-Guanidinopropyl)-2-(4-(octylsulfonamido)-[1,1’-biphenyl]-3-yl)-2-oxoacetamide hydrochloride (**22a**)

The titled compound was synthesised from (*E*)-1-tert-Butyl-*N*-(*N*’-((tert-butyloxidanyl)carbonyl)-*N*-(3-(2-(4-(octylsulfonamido)-[1,1’-biphenyl]-3-yl)-2-oxoacetamido)propyl)carbamimidoyl)-1-oxidanecarboxamide **21a** (40 mg, 0.056 mmol) following general synthetic procedure I. The product was obtained as a yellow sticky solid (0.15 g, 50%); ^1^H NMR (600 MHz, DMSO-*d*_6_): δ 10.20 (bs, 1H, NH), 9.01 (t, *J* = 5.7 Hz, 1H, NH), 8.03–7.99 (m, 2H, ArH), 7.69–7.61 (m, 4H, NH, ArH), 7.57–6.78 (m, 7H, NH, ArH), 3.30 (q, *J* = 6.5 Hz, 2H, CH_2_), 3.28–3.23 (m, 2H, CH_2_), 3.19 (q, *J* = 6.5 Hz, 2H, CH_2_), 1.79–1.63 (m, 4H, CH_2_), 1.37–1.29 (m, 2H, CH_2_), 1.26–1.15 (m, 8H, CH_2_), 0.82 (t, *J* = 7.1 Hz, 3H, CH_3_); ^13^C NMR (150 MHz, DMSO-*d*_6_): δ 192.6 (CO), 163.8 (CO), 156.9 (CN), 138.3 (ArC), 138.2 (ArC), 135.6 (ArC), 133.2 (ArCH), 130.5 (ArCH), 129.2 (ArCH), 127.9 (ArCH), 126.4 (ArCH), 124.5 (ArC), 121.6 (ArCH), 51.4 (CH_2_), 38.4 (CH_2_), 36.1 (CH_2_), 31.1 (CH_2_), 28.4 (CH_2_), 28.4 (CH_2_), 28.3 (CH_2_), 27.3 (CH_2_), 22.9 (CH_2_), 22.0 (CH_2_), 13.9 (CH_3_); IR (ATR): *ν*_max_ 3364, 3163, 2924, 2654, 2360, 1626, 1581, 1528, 1510, 1485, 1459, 1395, 1345, 1265, 1198, 1139, 1067, 924, 909, 849, 759, 683, 621, 587, 560, 530, 481, 424 cm^−1^; HRMS (+ ESI): Found *m/z* 516.2636 [M + H] ^+^, C_26_H_38_N_5_O_4_S required 516.2639.

##### 2-(4’-Fluoro-4-(octylsulfonamido)-[1,1’-biphenyl]-3-yl)-*N*-(3-guanidinopropyl)-2-oxoacetamide hydrochloride (**22b**)

The titled compound was synthesised from (*E*)-1-*tert*-Butyl-*N*-(*N*’-((*tert*-butyloxidanyl)carbonyl)-*N*-(3-(2-(4’-fluoro-4-(octylsulfonamido)-[1,1’-biphenyl]-3-yl)-2-oxoacetamido)propyl)carbamimidoyl)-1-oxidanecarboxamide **21b** (0.10 g, 0.13 mmol) following general synthetic procedure I. The product was obtained as a yellow sticky solid (49 mg, 61%); ^1^H NMR (600 MHz, DMSO-*d*_6_): δ 10.19 (bs, 1H, NH), 9.01 (t, *J* = 5.9 Hz, 1H, NH), 8.00–7.96 (m, 2H, ArH), 7.74 (t, *J* = 5.8 Hz, 1H, NH), 7.71–7.66 (m, 2H, ArH), 7.64–7.60 (m, 1H, ArH), 7.58–6.80 (m, 6H, NH, ArH), 3.30 (q, *J* = 6.7 Hz, 2H, CH_2_), 3.26–3.22 (m, 2H, CH_2_), 3.20 (q, *J* = 6.7 Hz, 2H, CH_2_), 1.77–1.71 (m, 2H, CH_2_), 1.70–1.63 (m, 2H, CH_2_), 1.36–1.30 (m, 2H, CH_2_), 1.26–1.15 (m, 8H, CH_2_), 0.82 (t, *J* = 7.2 Hz, 3H, CH_3_); ^13^C NMR (150 MHz, DMSO-*d*_6_): δ 192.4 (CO), 163.7 (CO), 162.1 (ArC), 157.0 (CN), 138.2 (ArC), 134.7 (ArC), 134.6 (ArC), 133.0 (ArCH), 130.4 (ArCH), 128.5 (ArCH), 124.6 (ArC), 121.8 (ArCH), 116.0 (ArCH), 51.4 (CH_2_), 38.4 (CH_2_), 36.1 (CH_2_), 31.1 (CH_2_), 28.4 (CH_2_), 28.4 (CH_2_), 28.3 (CH_2_), 27.3 (CH_2_), 22.9 (CH_2_), 22.0 (CH_2_), 13.9 (CH_3_); IR (ATR): *ν*_max_ 3313, 3155, 2923, 2853, 2292, 1910, 1639, 1487, 1396, 1330, 1195, 1137, 913, 826, 722 cm^−1^; HRMS (+ ESI): Found *m/z* 534.2544 [M + H] ^+^, C_26_H_37_FN_5_O_4_S required 534.2545.

##### 2-(4’-Chloro-4-(octylsulfonamido)-[1,1’-biphenyl]-3-yl)-*N*-(3-guanidinopropyl)-2-oxoacetamide hydrochloride (**22c**)

The titled compound was synthesised from (*E*)-1-*tert*-butyl-*N*-(*N*’-((*tert*-butyloxidanyl)carbonyl)-*N*-(3-(2-(4’-chloro-4-(octylsulfonamido)-[1,1’-biphenyl]-3-yl)-2-oxoacetamido)propyl)carbamimidoyl)-1-oxidanecarboxamide **21c** (91 mg, 0.12 mmol) following general synthetic procedure I. The product was obtained as a yellow sticky solid (43 mg, 60%); ^1^H NMR (600 MHz, DMSO-*d*_6_): δ 10.20 (bs, 1H, NH), 9.00 (t, *J* = 5.8 Hz, 1H, NH), 8.03–7.98 (m, 2H, ArH), 7.71–7.66 (m, 3H, NH, ArH), 7.63 (dd, *J* = 7.6, 1.5 Hz, 1H, ArH), 7.58–7.54 (m, 2H, ArH), 7.52–6.84 (bs, 4H, NH), 3.30 (q, *J* = 6.7 Hz, 2H, CH_2_), 3.27–3.22 (m, 2H, CH_2_), 3.19 (q, *J* = 6.6 Hz, 2H, CH_2_), 1.77–1.71 (m, 2H, CH_2_), 1.70–1.63 (m, 2H, CH_2_), 1.36–1.30 (m, 2H, CH_2_), 1.26–1.15 (m, 8H, CH_2_), 0.82 (t, *J* = 7.2 Hz, 3H, CH_3_); ^13^C NMR (150 MHz, DMSO-*d*_6_): δ 192.3 (CO), 163.7 (CO), 156.9 (CN), 138.5 (ArC), 137.1 (ArC), 134.2 (ArC), 133.0 (ArCH), 132.8 (ArC), 130.4 (ArCH), 129.2 (ArCH), 128.2 (ArCH), 124.7 (ArC), 121.8 (ArCH), 51.5 (CH_2_), 38.4 (CH_2_), 36.1 (CH_2_), 31.1 (CH_2_), 28.4 (CH_2_), 28.4 (CH_2_), 28.3 (CH_2_), 27.3 (CH_2_), 22.9 (CH_2_), 22.0 (CH_2_), 13.9 (CH_3_); IR (ATR): *ν*_max_ 3164, 2925, 2854, 2360, 1640, 1534, 1507, 1481, 1397, 1332, 1273, 1197, 1139, 1093, 1012, 915, 818, 758, 697, 657, 508 cm^−1^; HRMS (+ ESI): Found *m/z* 550.2252 [M + H] ^+^, C_26_H_37_ClN_5_O_4_S required 550.2249.

##### *N*-(3-Guanidinopropyl)-2-(5-(naphthalen-2-yl)-2-(octylsulfonamido)phenyl)-2-oxoacetamide hydrochloride (**22d**)

The titled compound was synthesised from (*E*)-1-*tert*-butyl-*N*-(*N*’-((*tert*-butyloxidanyl)carbonyl)-*N*-(3-(2-(5-(naphthalen-2-yl)-2-(octylsulfonamido)phenyl)-2-oxoacetamido)propyl)carbamimidoyl)-1-oxidanecarboxamide **21d** (0.10 g, 0.13 mmol) following general synthetic procedure I. The product was obtained as a yellow sticky solid (49 mg, 61%); ^1^H NMR (600 MHz, DMSO-*d*_6_): δ 10.22 (bs, 1H, NH), 9.03 (t, *J* = 5.8 Hz, 1H, NH), 8.21 (s, 1H, ArH), 8.17–8.14 (m, 2H, ArH), 8.07–7.94 (m, 3H, ArH), 7.81 (dd, *J* = 8.5, 1.9 Hz, 1H, ArH), 7.73 (t, *J* = 6.0 Hz, 1H, NH), 7.69–7.66 (m, 1H, ArH), 7.62–6.80 (m, 6H, NH, ArH), 3.32 (q, *J* = 6.8 Hz, 2H, CH_2_), 3.28–3.24 (m, 2H, CH_2_), 3.21 (q, *J* = 6.8 Hz, 2H, CH_2_), 1.79–1.72 (m, 2H, CH_2_), 1.72–1.65 (m, 2H, CH_2_), 1.38–1.31 (m, 2H, CH_2_), 1.27–1.15 (m, 8H, CH_2_), 0.81 (t, *J* = 7.2 Hz, 3H, CH_3_); ^13^C NMR (150 MHz, DMSO-*d*_6_): δ 192.6 (CO), 163.8 (CO), 157.0 (CN), 138.2 (ArC), 135.6 (ArC), 135.5 (ArC), 133.3 (ArCH), 133.2 (ArC), 132.4 (ArC), 130.6 (ArCH), 128.8 (ArCH), 128.2 (ArCH), 127.6 (ArCH), 126.7 (ArCH), 126.4 (ArCH), 125.2 (ArCH), 125.0 (ArC), 124.6 (ArCH), 122.0 (ArCH), 51.4 (CH_2_), 38.4 (CH_2_), 36.1 (CH_2_), 31.1 (CH_2_), 28.4 (CH_2_), 28.4 (CH_2_), 28.3 (CH_2_), 27.3 (CH_2_), 22.9 (CH_2_), 22.0 (CH_2_), 13.9 (CH_3_); IR (ATR): *ν*_max_ 3324, 3159, 3055, 2923, 2853, 2321, 2112, 1924, 1747, 1639, 1493, 1463, 1396, 1328, 1267, 1139, 1189, 913, 814 cm^−1^; HRMS (+ ESI): Found *m/z* 566.2793 [M + H] ^+^, C_30_H_40_N_5_O_4_S required 566.2796.

##### 1-(2-Naphthoyl)-5-phenylindoline-2,3-dione (**24**)

To a suspension of sodium hydride (90 mg, 2.25 mmol) in dimethylformamide (5 mL), slowly dropwise, a solution of 5-phenylindoline-2,3-dione **7a** (0.43 g, 1.92 mmol) in dimethylformamide (5 mL) was added at 0 °C under nitrogen atmosphere. The reaction mixture was stirred at 0 °C for 15 min. A solution of 2-naphthoyl chloride (0.38 g, 2.01 mmol) in dimethylformamide (7 mL) was then added slowly dropwise to the reaction mixture at 0 °C with stirring. The reaction mixture was then stirred at room temperature for 3 h. After completion of the reaction, the resulting mixture was poured into 1:1 ice-water mixture. The yellow precipitate was then collected via vacuum filtration and washed with methanol to afford the product as yellow solid (0.25 g, 35%); mp 127.4–127.5 °C; ^1^H NMR (600 MHz, DMSO-*d*_6_): δ 8.60 (d, *J* = 1.2 Hz, 1H, ArH), 8.16 (dd, *J* = 8.5, 2.2 Hz, 1H, ArH), 8.09–8.03 (m, 5H, ArH), 7.95 (dd, *J* = 8.5, 1.7 Hz, 1H, ArH), 7.80–7.77 (m, 2H, ArH), 7.73–7.69 (m, 1H, ArH), 7.67–7.63 (m, 1H, ArH), 7.54–7.50 (m, 2H, ArH), 7.45–7.41 (m, 1H, ArH); ^13^C NMR (150 MHz, DMSO-*d*_6_): δ 180.2 (CO), 168.0 (CO), 157.4 (CO), 147.3 (ArC), 138.2 (ArC), 137.2 (ArC), 135.6 (ArCH), 135.0 (ArC), 131.8 (ArC), 131.2 (ArCH), 131.1 (ArC), 129.2 (ArCH), 129.2 (ArCH), 128.7 (ArCH), 128.1 (ArCH), 127.8 (ArCH), 127.6 (ArCH), 127.0 (ArCH), 126.6 (ArCH), 125.5 (ArCH), 121.9 (ArCH), 120.7 (ArC), 116.7 (ArCH); IR (ATR): *ν*_max_ 3854, 3675, 2987, 2900, 1948, 1762, 1739, 1692, 1615, 1589, 1508, 1473, 1458, 1406, 1393, 1357, 1306, 1285, 1223, 1193, 1161, 1122, 1066, 1057, 1027, 990, 967, 953, 928, 904, 890, 868, 856, 828, 793, 776, 760, 717, 704, 691, 622, 584, 541, 516, 481, 463 cm^−1^; HRMS (+ ESI): Found *m/z* 400.0945 [M + Na] ^+^, C_25_H_15_NO_3_Na required 400.0944.

##### *N*-(3-(2-((3-(Dimethylamino)propyl)amino)-2-oxoacetyl)-[1,1’-biphenyl]-4-yl)-2-naphthamide (**25**)

To a solution of 1-(2-naphthoyl)-5-phenylindoline-2,3-dione **24** (0.11 g, 0.30 mmol) in dichloromethane (5 mL), 3-dimethylaminopropylamine (38 μL, 0.30 mmol) was added at 0 °C. The reaction mixture was stirred at room temperature for 6 h. After completion of the reaction, water was added to the reaction mixture and the product was extracted into dichloromethane (3 × 30 mL), washed with brine, dried over anhydrous sodium sulphate and concentrated in vacuo to afford the product as a yellow solid (0.13 g, 93%); mp 151.6–151.8 °C; ^1^H NMR (600 MHz, CDCl_3_): δ 12.21 (bs, 1H, NH), 9.05 (d, *J* = 9.0 Hz, 1H, ArH), 8.71 (d, *J* = 2.2 Hz, 1H, ArH), 8.60 (bs, 1H, NH), 8.58 (d, *J* = 1.3 Hz, 1H, ArH), 8.10 (dd, *J* = 8.6, 2.0 Hz, 1H, ArH), 8.02 (dd, *J* = 7.2, 0.6 Hz, 1H, ArH), 7.98 (d, *J* = 8.6 Hz, 1H, ArH), 7.95–7.90 (m, 2H, ArH), 7.64–7.56 (m, 4H, ArH), 7.48–7.44 (m, 2H, ArH), 7.39–7.35 (m, 1H, ArH), 3.57 (q, *J* = 5.7 Hz, 2H, CH_2_), 2.54 (t, *J* = 6.4 Hz, 2H, CH_2_), 2.32 (s, 6H, CH_3_), 1.87–1.81 (m, 2H, CH_2_); ^13^C NMR (150 MHz, CDCl_3_): δ 193.1 (CO), 166.1 (CO), 163.4 (CO), 141.8 (ArC), 139.5 (ArC), 135.7 (ArC), 135.2 (ArC), 135.2 (ArCH), 133.1 (ArCH), 132.9 (ArC), 131.9 (ArC), 129.5 (ArCH), 129.1 (ArCH), 128.9 (ArCH), 128.6 (ArCH), 128.2 (ArCH), 127.9 (ArCH), 127.8 (ArCH), 127.0 (ArCH), 127.0 (ArCH), 123.8 (ArCH), 121.4 (ArCH), 119.6 (ArC), 58.5 (CH_2_), 45.3 (CH_3_), 39.6 (CH_2_), 25.6 (CH_2_); IR (ATR): *ν*_max_ 3854, 3675, 3309, 2972, 2900, 2780, 1762, 1735, 1679, 1644, 1627, 1585, 1522, 1492, 1472, 1449, 1394, 1341, 1301, 1285, 1222, 1191, 1132, 1065, 1027, 965, 910, 890, 858, 818, 775, 758, 697, 679, 609, 572, 539, 512, 483, 466, 446 cm^−1^; HRMS ( + ESI): Found *m/z* 480.2283 [M + H] ^+^, C_30_H_30_N_3_O_3_ required 480.2282.

##### 3-(2-(4-(2-Naphthamido)-[1,1’-biphenyl]-3-yl)-2-oxoacetamido)-*N*,*N*-dimethylpropan-1-aminium chloride (**26**)

To a solution of *N*-(3-(2-((3-(dimethylamino)propyl)amino)-2-oxoacetyl)-[1,1’-biphenyl]-4-yl)-2-naphthamide **25** (32 mg, 0.067 mmol) in dichloromethane (5 mL), 4 M HCl/dioxane (0.10 mL, 0.40 mmol) was added. The reaction mixture was stirred at room temperature for 20 min. After completion of reaction, the reaction mixture was concentrated in vacuo, washed thrice with diethyl ether and freeze-dried to afford the product as a yellow sticky solid (29 mg, 84%); ^1^H NMR (600 MHz, DMSO-*d*_6_): δ 11.43 (s, 1H, NH), 10.38 (bs, 1H, NH), 9.01 (t, *J* = 6.1 Hz, 1H, NH), 8.63 (s, 1H, ArH), 8.18–8.10 (m, 3H, ArH), 8.07–8.01 (m, 4H, ArH), 7.73–7.64 (m, 4H, ArH), 7.52 (t, *J* = 7.9 Hz, 2H, ArH), 7.41 (t, *J* = 7.3 Hz, 2H, ArH), 3.24 (q, *J* = 6.7 Hz, 2H, CH_2_), 3.02–2.97 (m, 2H, CH_2_), 2.63 (s, 6H, CH_3_), 1.87–1.79 (m, 2H, CH_2_); ^13^C NMR (150 MHz, DMSO-*d*_6_): δ 190.6 (CO), 165.5 (CO), 163.2 (CO), 138.6 (ArC), 137.6 (ArC), 135.6 (ArC), 134.5 (ArC), 132.1 (ArC), 131.9 (ArCH), 131.2 (ArC), 129.3 (ArCH), 129.2 (ArCH), 129.1 (ArCH), 128.5 (ArCH), 128.2 (ArCH), 128.2 (ArCH), 127.8 (ArCH), 127.8 (ArCH), 127.1 (ArCH), 126.5 (ArCH), 125.5 (ArC), 123.9 (ArCH), 123.0 (ArCH), 54.3 (CH_2_), 41.9 (CH_3_), 36.0 (CH_2_), 23.8 (CH_2_); IR (ATR): *ν*_max_ 3331, 3056, 2963, 2681, 2361, 1676, 1643, 1626, 1585, 1523, 1493, 1448, 1396, 1368, 1341, 1306, 1286, 1245, 1219, 1189, 1133, 1068, 967, 912, 891, 849, 819, 761, 699, 681, 572, 512, 487 cm^−1^; HRMS ( + ESI): Found *m/z* 480.2281 [M + H] ^+^, C_30_H_30_N_3_O_3_ required 480.2282.

##### 3-(2-(4-(2-Naphthamido)-[1,1’-biphenyl]-3-yl)-2-oxoacetamido)-*N*,*N*,*N*-trimethylpropan-1-aminium iodide (**27**)

To a solution of *N*-(3-(2-((3-(dimethylamino)propyl)amino)-2-oxoacetyl)-[1,1’-biphenyl]-4-yl)-2-naphthamide **25** (34 mg, 0.071 mmol) in THF (5 mL), iodomethane (11 μL, 0.18 mmol) was added. The reaction mixture was stirred at room temperature for 24 h. After completion of reaction, the reaction mixture was concentrated in vacuo, washed thrice with diethyl ether and freeze-dried to afford the product as a yellow sticky solid (35 mg, 79%); ^1^H NMR (600 MHz, DMSO-*d*_6_): δ 10.37 (bs, 1H, NH), 8.98 (t, *J* = 6.0 Hz, 1H, NH), 8.61 (d, *J* = 1.1 Hz, 1H, ArH), 8.14–8.08 (m, 3H, ArH), 8.07–8.01 (m, 4H, ArH), 7.73–7.64 (m, 4H, ArH), 7.54–7.49 (m, 2H, ArH), 7.44–7.40 (m, 1H, ArH), 3.30–3.25 (m, 2H, CH_2_), 3.23 (q, *J* = 6.6 Hz, 2H, CH_2_), 2.95 (s, 9H, CH_2_), 1.90–1.82 (m, 2H, CH_2_); ^13^C NMR (150 MHz, DMSO-*d*_6_): δ 190.3 (CO), 165.6 (CO), 163.0 (CO), 138.7 (ArC), 137.4 (ArC), 135.6 (ArC),134.5 (ArC), 132.1 (ArC), 131.8 (ArCH), 131.3 (ArC), 129.3 (ArCH), 129.2 (ArCH), 129.0 (ArCH), 128.5 (ArCH), 128.3 (ArCH), 128.2 (ArCH), 127.8 (ArCH), 127.8 (ArCH), 127.2 (ArCH), 126.5 (ArCH), 125.8 (ArC), 123.9 (ArCH), 123.1 (ArCH), 63.3 (CH_2_), 52.2 (CH_3_), 35.9 (CH_2_), 22.5 (CH_2_); IR (ATR): *ν*_max_ 3319, 2999, 2360, 2160, 1978, 1677, 1644, 1625, 1586, 1525, 1494, 1447, 1399, 1342, 1306, 1286, 1198, 1068, 966, 912, 868, 850, 825, 763, 698, 681, 609, 573, 517, 488, 472, 426 cm^−1^; HRMS (+ ESI): Found *m/z* 494.2439 [M] ^+^, C_31_H_32_N_3_O_3_ required 494.2438.

### 3.2. Minimum Inhibitory Concentration (MIC) Assay

The antimicrobial activity of the compounds was evaluated by MIC assay using the procedure described by Clinical and Laboratory Standards Institute (CLSI). A single colony of bacteria was cultured overnight in trypticase soy broth (TSB; Oxoid, Basingstoke, UK) at 37 °C with shaking. The resulting bacterial culture was collected by centrifugation and resuspended in TSB twice. The optical density (OD) of the resulting culture was adjusted to OD_660_ = 0.1 in TSB (which is equivalent to 10^8^ colony forming unit (CFU)/mL bacteria). It was further diluted to 10^6^ CFU/mL in TSB. 100 µL of the bacterial solution was then added to wells of a 96-well plate (Costar; Sigma-Aldrich, St Louis, MO, USA) containing 100 µL serially diluted peptide mimic, with final concentration ranging from 1 to 250 µM. Wells with bacteria but no compound were used as negative control while wells with only media were set as blank. The plates were then wrapped with parafilm to prevent evaporation and incubated with shaking at 120 rpm at 37 °C for 18–24 h, and the data were recorded by measuring the OD value at 660 nm using a FLUOstar Omega (BMG Labtech, Mornington, Victoria, Australia) microplate reader. The MIC value of each compound was determined as the lowest concentration that completely inhibited the growth of bacteria. Each experiment was performed in triplicate and was repeated in three independent experiments.

### 3.3. Biofilm Disruption Assay

Bacterial cultures (*S. aureus* and *E. coli*) were grown in Muller Hinton broth (MHB; Oxoid, Basingstoke, UK) media overnight at 37 °C with shaking at 120 rpm. Cultures were diluted (1:20) in MHB medium and 200 µL aliquots were dispensed to wells in a flat bottom 96-well plate (Costar; Sigma-Aldrich, St Louis, MO, USA). Biofilm was then grown in the 96-well plate at 37 °C overnight. After that, loosely bound cells were washed away with 1x phosphate-buffered saline (PBS) and cultures were then supplemented with different concentrations of test compounds dissolved in DMSO and incubated for a further 24 h with shaking at 120 rpm. Biofilms adhered on the plate substratum were quantified using crystal violet staining as described previously [18,63]. The experiment was performed in triplicate.

### 3.4. Cytoplasmic Membrane Permeability Assay

Bacterial cytoplasmic membrane permeability was determined using membrane potential-sensitive dye diSC3–5 (3,3′-dipropylthiadicarbocyanine iodide), which penetrates inside bacterial cells depending on the membrane potential gradient of the cytoplasmic membrane. The procedure of this assay follows the method previously described by Wu et al. [64] with slight modifications. Bacteria were grown in MHB to mid-log phase by incubating with shaking at 37 °C for 16 h. Following incubation, bacteria were washed with 5 mM HEPES containing 20 mM glucose at pH 7.2 and resuspended in the same buffer to an OD_600_ 0.05–0.06 (yielding a final concentration of 10^7^ CFU/mL). The dye diSC3–5 was added at 4 μM to the bacterial suspension. The suspensions were incubated at room temperature for 1 h in darkness for maximum dye take-up by the bacterial cells. 100 mM KCl was then added to balance the K^+^ outside and inside the bacterial cell. 100 μL of bacterial suspension was added in a 96-well microtiter plate and with equal volume of antimicrobial compounds. DMSO (10%) was set as a positive control while dye and only bacterial cells were set as negative control. Fluorescence due to release of dye was measured with a luminescence spectrophotometer at 3 min intervals at an excitation wavelength of 621 nm and an emission wavelength of 670 nm.

### 3.5. Cell Viability Count Assay

The number of viable cells was confirmed by serially diluting aliquots of bacteria in D/E neutralizing broth (Remel, Lenexa, KS, USA) and plating onto Tryptic Soy Agar (Oxoid, Basingstoke, UK) containing phosphatidylcholine (0.7 g/L) and Tween 80 (5 mL/L). The plates were incubated at 37 °C overnight and numbers of live bacteria were enumerated and expressed as CFU/mL. The experiment was performed in triplicate [36].

### 3.6. Tethered Bilayer Lipid Membranes (tBLMs) Assay

Changes in membrane conduction and capacitance were measured using tethered bilayer lipid membranes (tBLMs) in association with AC electrical impedance spectroscopy techniques [60,65]. Sparsely tethered tBLMs were prepared using a zwitterionic 1-palmitoyl-2-oleoyl-glycero-3-phosphocholine (POPC) (Avanti Lipids, USA) or a mixture of 30% palmitoyl-oleoyl-phosphatidylglycerol (POPG) (Avanti Lipids, USA) lipids with 70% POPC in order to create a negatively charged tBLM [66,67]. To do this, gold-patterned polycarbonate slides were coated with tethered benzyl-disulfide (tetra-ethyleneglycol) *n* = 2 C20-phytanyl tethers:benzyl-disulfide-tetra-ethyleneglycol-OH spacers in the ratio of 1:10 (SDx Tethered Membranes Pty Ltd., Australia). A 3 mM solution of a mobile lipid phase POPC, or a 3 mM solution of mobile phase POPC/POPG (70:30), (Avanti Lipids, USA) in 100% ethanol was then incubated with the tethering chemistries. Lipids were left to incubate with the tethering chemistries for 2 min before being washed with 3 × 400 mL of phosphate-buffered saline (PBS). Prior to the addition of the compounds, membrane stability was confirmed by 5 × 100 µL washes of the PBS vehicle. AC electrical impedance spectrometry (EIS) was then used to monitor any changes in membrane impedances as a result of adding the test compounds in increasing concentrations.

For EIS measures, a 50 mV peak-to-peak AC excitation from 0.1 to 2000 Hz with four steps per decade were recorded using a TethaPod™ operated with TethaQuick™ software (SDx Tethered Membranes Pty Ltd., Australia). The data were fitted to an equivalent circuit consisting of a Constant Phase Element (CPE), to represent the imperfect capacitance of the gold tethering electrode, in series with a Resistor/Capacitor network to represent the lipid bilayer membrane [60]. Fitting of the phase and impedance data to the equivalent circuit was completed with a proprietary adaptation of a Lev Mar fitting routine. To account for variations in basal membrane conditions, data are normalised to a baseline conduction and capacitance values directly before the addition of each compound.

### 3.7. Toxicity Assay

The toxicity of the compounds was measured using an MTT (3-(4,5-dimethylthiazol-2-yl)-2,5-diphenyltetrazolium bromide) (Sigma Aldrich, St Louis, MO, USA) colorimetric assay against normal human lung fibroblasts MRC-5 cells. The cells were cultured in minimal essential medium (MEM; Sigma Aldrich, St Louis, MO, USA) containing 10% foetal bovine serum (FBS). The cells were maintained at 37 °C in 5% CO_2_ as an adherent monolayer and was passaged upon reaching confluence by standard cell culture techniques. MRC-5 cells were seeded at 5000 cells/well in 96-well plates to ensure full confluence (quiescence) and left overnight to adhere to the plate wells. The adherent cells were treated with 1–500 µM of compounds by dissolving in DMSO and serially diluting with media. The final concentration of DMSO was 0.5% (*v/v*). After 72 h drug incubation, 20 mL stock MTT solution (5 mg/mL) was added and the cells were incubated for another 3.5–4 h. MTT solution-containing media was carefully aspirated without displacing the purple crystals from the bottom and 80 mL acid-isopropanol was added to all wells and mixed slowly by shaking with an orbital shaker to dissolve the dark blue crystals. The metabolic activity was detected by spectrophotometric analysis by assessing the read absorbance (590/620 nm) on an EnSight plate reader (PerkinElmer, Waltham, MA, USA) and cell viability was expressed as a percentage of untreated control cells. The determination of IC_50_ values was performed using GraphPad Prism 6 (GraphPad, San Diego, CA, USA). Each experiment was performed in triplicate and was repeated in three independent experiments.

## 4. Conclusions

A library of biphenylglyoxamide-based small molecular AMP mimics was successfully synthesised from different 5-arylisatins. Hydrophobicity was introduced to the AMP via *N-*sulfonylation of 5-arylisatins. The ring-opening reaction of *N*-sulfonylisatins followed by the conversion of the resulting glyoxamide derivatives into tertiary ammonium chloride, quaternary ammonium iodide or guanidinium hydrochloride salts conferred the cationicity of the AMP mimics. An in vitro antibacterial assay demonstrated that these AMP mimics possessed excellent antibacterial activities against Gram-positive *S. aureus*. Additionally, the quaternary ammonium iodide salts **15a**–**15c** and guanidinium hydrochloride salts **22a**–**22c** also showed moderate to high antibacterial activities against Gram-negative *P. aeruginosa* and *E. coli*. SAR studies revealed that the octanesulfonyl group was essential for Gram-positive antibacterial activity, while both the octanesulfonyl group and the biphenyl backbone were important for Gram-negative antibacterial activity. The most potent compounds, **15c, 22b** and **22d** (MIC = 8 µM against *S. aureus*), disrupted pre-established *S. aureus* biofilms by 35%, 39% and 50% respectively, at 32 µM (4× MIC). In a cytoplasmic membrane permeability study, the chloro-substituted quaternary ammonium iodide salt **15c** showed strong ability to disrupt and depolarise the bacterial cell membrane. These results were consistent with the tethered bilayer lipid membrane assay, where **15c** demonstrated its ability to disintegrate the bilayer lipid membrane. Finally, an in vitro toxicity assay performed against human MRC-5 fibroblast cells showed that the quaternary ammonium iodide salts and the guanidinium hydrochloride salts were of low cytotoxicity, with wide therapeutic windows for bacterial cells over human cells. Overall, this study demonstrated that biphenylglyoxamide-based quaternary ammonium iodide salts and guanidinium hydrochloride salts are new leads for the development of the next generation of small molecular AMP mimics that possess anti-biofilm and membrane disruption properties.

## Figures and Tables

**Table 1:** Antibacterial activity (minimum inhibitory concentration, MIC) of biphenylglyoxamide derivatives against different strains of bacteria.

	Compound	MIC (μM)		
		*S. aureus*	*P. aeruginosa*	*E. coli*
Glyoxamide derivatives (Series I)	**11a**	24	ND	ND
	**11b**	16	ND	ND
	**11c**	16	ND	ND
	**11d**	16	ND	ND
	**11e**	63	ND	ND
	**12a**	> 250	ND	ND
	**25**	> 250	ND	ND
Tertiary ammonium chloride salts (Series II)	**13a**	16	ND	ND
	**13b**	16	ND	ND
	**13c**	16	ND	ND
	**13d**	8	ND	ND
	**13e**	32	ND	ND
	**14a**	> 250	ND	ND
	**26**	> 250	ND	ND
Quaternary ammonium iodide salts (Series III)	**15a**	16	125	32
	**15b**	16	63	32
	**15c**	8	63	16
	**15d**	8	250	63
	**15e**	16	> 250	> 250
	**16a**	> 250	ND	ND
	**27**	32	ND	ND
Guanidinium hydrochloride salts (Series IV)	**22a**	8	63	63
	**22b**	8	63	16
	**22c**	8	250	63
	**22d**	8	> 250	> 250
	**pexiganan (MSI-78) ^a^**	3.2–6.5	3.2–6.5	3.2–6.5

ND = Not determined; ^a^ previously reported values. [51].

**Table 2:** IC_50_ value of compounds against MRC-5 normal human lung fibroblasts and their therapeutic indices with respect to different strains of bacteria.

	Compound	IC_50_ (μM)	Therapeutic Index		
			*S. aureus*	*P. aeruginosa*	*E. coli*
Glyoxamide derivatives	**11a**	28.2	1.18	N/A	N/A
	**11d**	14.2	0.89	N/A	N/A
Tertiary ammonium chloride salts	**13a**	25.0	1.56	N/A	N/A
	**13c**	25.5	1.59	N/A	N/A
	**13d**	19.7	2.46	N/A	N/A
	**13e**	23.6	0.74	N/A	N/A
Quaternary ammonium iodide salts	**15a**	50.7	3.17	0.41	1.59
	**15b**	190	11.9	3.04	5.94
	**15c**	95.8	12.0	1.53	5.99
	**15d**	112	14.0	0.45	1.79
	**15e**	101	6.31	N/A	N/A
Guanidinium hydrochloride salts	**22a**	75.5	9.44	1.21	1.21
	**22b**	76.0	9.50	1.21	4.75
	**22c**	95.0	11.9	0.38	1.52
	**22d**	49.5	6.19	N/A	N/A

N/A = Not applicable.

## Supplementary Materials

Supplementary materials can be found at https://www.mdpi.com/1422-0067/21/18/6789/s1, Figure S1. Percentage of remaining *S. aureus* biofilms after 24 h treatment with compounds **15d** (top), **22a** (middle) or **22c** (bottom) at 1×, 2× and 4× of their MIC. Error bars represent the standard error of triplicates (*n* = 3). Figure S2. Percentage of remaining *E. coli* biofilms after 24 h treatment with compounds **15c** or **22c** at 4× of their MIC (64 μM). Error bars represent the standard error of triplicates (*n* = 3).
