# Supplementary material for: Design, Synthesis and Biological Evaluation of Biphenylglyoxamide-Based Small Molecular Antimicrobial Peptide Mimics as Antibacterial Agents
Source: Int J Mol Sci. 2020 Sep 16;21(18):6789. doi: 10.3390/ijms21186789 (PMC7555970; doi:10.3390/ijms21186789)

Supporting information

Design, Synthesis and Biological Evaluation of Biphenylglyoxamide-based Small Molecular Antimicrobial Peptide Mimics as Antibacterial Agents

Tsz Tin Yu [1], Rajesh Kuppusamy [1], Muhammad Yasir [2], Md. Musfizur Hassan [1], Amani Alghalayini [3], Satyanarayana Gadde [1], Evelyne Deplazes [3], Charles Cranfield [3], Mark Willcox [2], David StC Black [1,*] and Naresh Kumar [1,*]

[1] School of Chemistry, The University of New South Wales, Sydney, NSW 2052, Australia
[2] School of Optometry and Vision Science, University of New South Wales, Sydney, NSW 2052, Australia
[3] School of Life Sciences, University of Technology Sydney, PO Box 123, Ultimo 2007, Australia
* Correspondence: d.black@unsw.edu.au; n.kumar@unsw.edu.au

Contents

Supplementary figures for the antibiofilm activity of compounds **15c-15d, 22a-22d** 4
^{1}H NMR and ^{13}C NMR spectra of synthesized compounds 6
5-Phenylindoline-2,3-dione (**7a**) 6
5-(4-Fluorophenyl)indoline-2,3-dione (**7b**) 7
5-(4-Chlorophenyl)indoline-2,3-dione (**7c**) 8
5-(Naphthalen-2-yl)indoline-2,3-dione (**7d**) 9
1-(Octylsulfonyl)-5-phenylindoline-2,3-dione (**8a**) 10
5-(4-Fluorophenyl)-1-(octylsulfonyl)indoline-2,3-dione (**8b**) 11
5-(4-Chlorophenyl)-1-(octylsulfonyl)indoline-2,3-dione (**8c**) 12
5-(Naphthalen-2-yl)-1-(octylsulfonyl)indoline-2,3-dione (**8d**) 13
5-Butyl-1-(octylsulfonyl)indoline-2,3-dione (**8e**) 14
1-(Butylsulfonyl)-5-phenylindoline-2,3-dione (**9a**) 15
N-(3-(Dimethylamino)propyl)-2-(4-(octylsulfonamido)-[1,1'-biphenyl]-3-yl)-2-oxoacetamide (**11a**) 16
N-(3-(Dimethylamino)propyl)-2-(4'-fluoro-4-(octylsulfonamido)-[1,1'-biphenyl]-3-yl)-2-oxoacetamide (**11b**) 17
2-(4'-Chloro-4-(octylsulfonamido)-[1,1'-biphenyl]-3-yl)-*N*-(3-(dimethylamino)propyl)-2-oxoacetamide (**11c**) 18
N-(3-(Dimethylamino)propyl)-2-(5-(naphthalen-2-yl)-2-(octylsulfonamido)phenyl)-2-oxoacetamide (**11d**) 19
2-(5-Butyl-2-(octylsulfonamido)phenyl)-*N*-(3-(dimethylamino)propyl)-2-oxoacetamide (**11e**) ... 20
2-(4-(Butylsulfonamido)-[1,1'-biphenyl]-3-yl)-*N*-(3-(dimethylamino)propyl)-2-oxoacetamide (**12a**) 21

N,*N*-Dimethyl-3-(2-(4-(octylsulfonamido)-[1,1'-biphenyl]-3-yl)-2-oxoacetamido)propan-1-aminium chloride (**13a**)....22
3-(2-(4'-Fluoro-4-(octylsulfonamido)-[1,1'-biphenyl]-3-yl)-2-oxoacetamido)-*N*,*N*-dimethylpropan-1-aminium chloride (**13b**)....23
3-(2-(4'-Chloro-4-(octylsulfonamido)-[1,1'-biphenyl]-3-yl)-2-oxoacetamido)-*N*,*N*-dimethylpropan-1-aminium chloride (**13c**)....24
N,*N*-Dimethyl-3-(2-(5-(naphthalen-2-yl)-2-(octylsulfonamido)phenyl)-2-oxoacetamido)propan-1-aminium chloride (**13d**)....25
3-(2-(5-Butyl-2-(octylsulfonamido)phenyl)-2-oxoacetamido)-*N*,*N*-dimethylpropan-1-aminium chloride (**13e**)....26
3-(2-(4-(Butylsulfonamido)-[1,1'-biphenyl]-3-yl)-2-oxoacetamido)-*N*,*N*-dimethylpropan-1-aminium chloride (**14a**)....27
N,*N*,*N*-Trimethyl-3-(2-(4-(octylsulfonamido)-[1,1'-biphenyl]-3-yl)-2-oxoacetamido)propan-1-aminium iodide (**15a**)....28
3-(2-(4'-Fluoro-4-(octylsulfonamido)-[1,1'-biphenyl]-3-yl)-2-oxoacetamido)-*N*,*N*,*N*-trimethylpropan-1-aminium iodide (**15b**)....29
3-(2-(4'-Chloro-4-(octylsulfonamido)-[1,1'-biphenyl]-3-yl)-2-oxoacetamido)-*N*,*N*,*N*-trimethylpropan-1-aminium iodide (**15c**)....30
N,*N*,*N*-Trimethyl-3-(2-(5-(naphthalen-2-yl)-2-(octylsulfonamido)phenyl)-2-oxoacetamido)propan-1-aminium iodide (**15d**)....31
3-(2-(5-Butyl-2-(octylsulfonamido)phenyl)-2-oxoacetamido)-*N*,*N*,*N*-trimethylpropan-1-aminium iodide (**15e**)....32
3-(2-(4-(Butylsulfonamido)-[1,1'-biphenyl]-3-yl)-2-oxoacetamido)-*N*,*N*,*N*-trimethylpropan-1-aminium iodide (**16a**)....33
tert-Butyl (3-(2-(4-(octylsulfonamido)-[1,1'-biphenyl]-3-yl)-2-oxoacetamido)propyl)carbamate (**18a**)....34
tert-Butyl (3-(2-(4'-fluoro-4-(octylsulfonamido)-[1,1'-biphenyl]-3-yl)-2-oxoacetamido)propyl) carbamate (**18b**)....35
tert-Butyl (3-(2-(4'-Chloro-4-(octylsulfonamido)-[1,1'-biphenyl]-3-yl)-2-oxoacetamido)propyl) carbamate (**18c**)....36
tert-Butyl (3-(2-(5-(naphthalen-2-yl)-2-(octylsulfonamido)phenyl)-2-oxoacetamido)propyl) carbamate (**18d**)....37
N-(3-Aminopropyl)-2-(4-(octylsulfonamido)-[1,1'-biphenyl]-3-yl)-2-oxoacetamide hydrochloride (**19a**)....38
N-(3-Aminopropyl)-2-(4'-fluoro-4-(octylsulfonamido)-[1,1'-biphenyl]-3-yl)-2-oxoacetamide hydrochloride (**19b**)....39
N-(3-Aminopropyl)-2-(4'-chloro-4-(octylsulfonamido)-[1,1'-biphenyl]-3-yl)-2-oxoacetamide hydrochloride (**19c**)....40
N-(3-Aminopropyl)-2-(5-(naphthalen-2-yl)-2-(octylsulfonamido)phenyl)-2-oxoacetamide hydrochloride (**19d**)....41

(*E*)-1-tert-Butyl-*N*-(*N*'-((*tert*-butyloxidanyl)carbonyl)-*N*-(3-(2-(4-(octylsulfonamido)-[1,1'-biphenyl]-3-yl)-2-oxoacetamido)propyl)carbamimidoyl)-1-oxidanecarboxamide (**21a**)...............42
(*E*)-1-*tert*-Butyl-*N*-(*N*'-((*tert*-butyloxidanyl)carbonyl)-*N*-(3-(2-(4'-fluoro-4-(octylsulfonamido)-[1,1'-biphenyl]-3-yl)-2-oxoacetamido)propyl)carbamimidoyl)-1-oxidanecarboxamide (**21b**)......43
(*E*)-1-*tert*-Butyl-*N*-(*N*'-((*tert*-butyloxidanyl)carbonyl)-*N*-(3-(2-(4'-chloro-4-(octylsulfonamido)-[1,1'-biphenyl]-3-yl)-2-oxoacetamido)propyl)carbamimidoyl)-1-oxidanecarboxamide (**21c**).......44
(*E*)-1-*tert*-Butyl-*N*-(*N*'-((*tert*-butyloxidanyl)carbonyl)-*N*-(3-(2-(5-(naphthalen-2-yl)-2-(octylsulfonamido)phenyl)-2-oxoacetamido)propyl)carbamimidoyl)-1-oxidanecarboxamide (**21d**) 45
N-(3-Guanidinopropyl)-2-(4-(octylsulfonamido)-[1,1'-biphenyl]-3-yl)-2-oxoacetamide hydrochloride (**22a**) 46
2-(4'-Fluoro-4-(octylsulfonamido)-[1,1'-biphenyl]-3-yl)-*N*-(3-guanidinopropyl)-2-oxoacetamide hydrochloride (**22b**) 47
2-(4'-Chloro-4-(octylsulfonamido)-[1,1'-biphenyl]-3-yl)-*N*-(3-guanidinopropyl)-2-oxoacetamide hydrochloride (**22c**)..........48
N-(3-Guanidinopropyl)-2-(5-(naphthalen-2-yl)-2-(octylsulfonamido)phenyl)-2-oxoacetamide hydrochloride (**22d**) 49
1-(2-Naphthoyl)-5-phenylindoline-2,3-dione (**24**) 50
N-(3-(2-((3-(Dimethylamino)propyl)amino)-2-oxoacetyl)-[1,1'-biphenyl]-4-yl)-2-naphthamide (**25**)..........51
3-(2-(4-(2-Naphthamido)-[1,1'-biphenyl]-3-yl)-2-oxoacetamido)-*N*,*N*-dimethylpropan-1-aminium chloride (**26**)..........52
3-(2-(4-(2-Naphthamido)-[1,1'-biphenyl]-3-yl)-2-oxoacetamido)-*N*,*N*,*N*-trimethylpropan-1-aminium iodide (**27**)..........53

Supplementary figures for the antibiofilm activity of compounds 15c-15d, 22a-22d

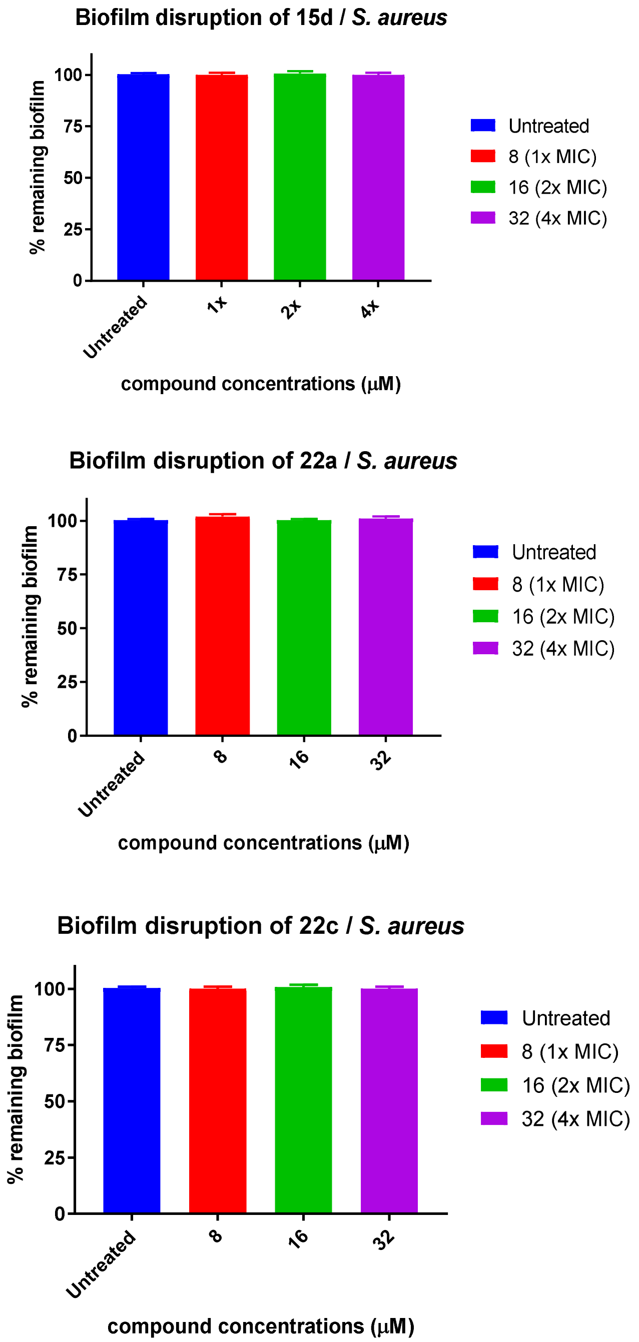

Figure S1. Percentage of remaining *S. aureus* biofilms after 24 h treatment with compounds **15d** (top), **22a** (middle) or **22c** (bottom) at 1×, 2× and 4× of their MIC. Error bars represent the standard error of triplicates (n = 3).

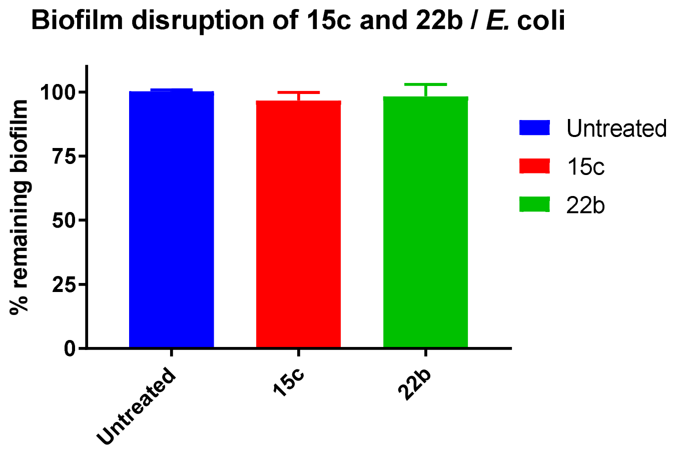

Figure S2. Percentage of remaining *E. coli* biofilms after 24 h treatment with compounds **15c** or **22c** at 4× of their MIC (64 μM). Error bars represent the standard error of triplicates (n = 3).

^{1}H NMR and ^{13}C NMR spectra of synthesized compounds

5-Phenylindoline-2,3-dione (**7a**)

^{1}H NMR (400 MHz, DMSO-d_6):

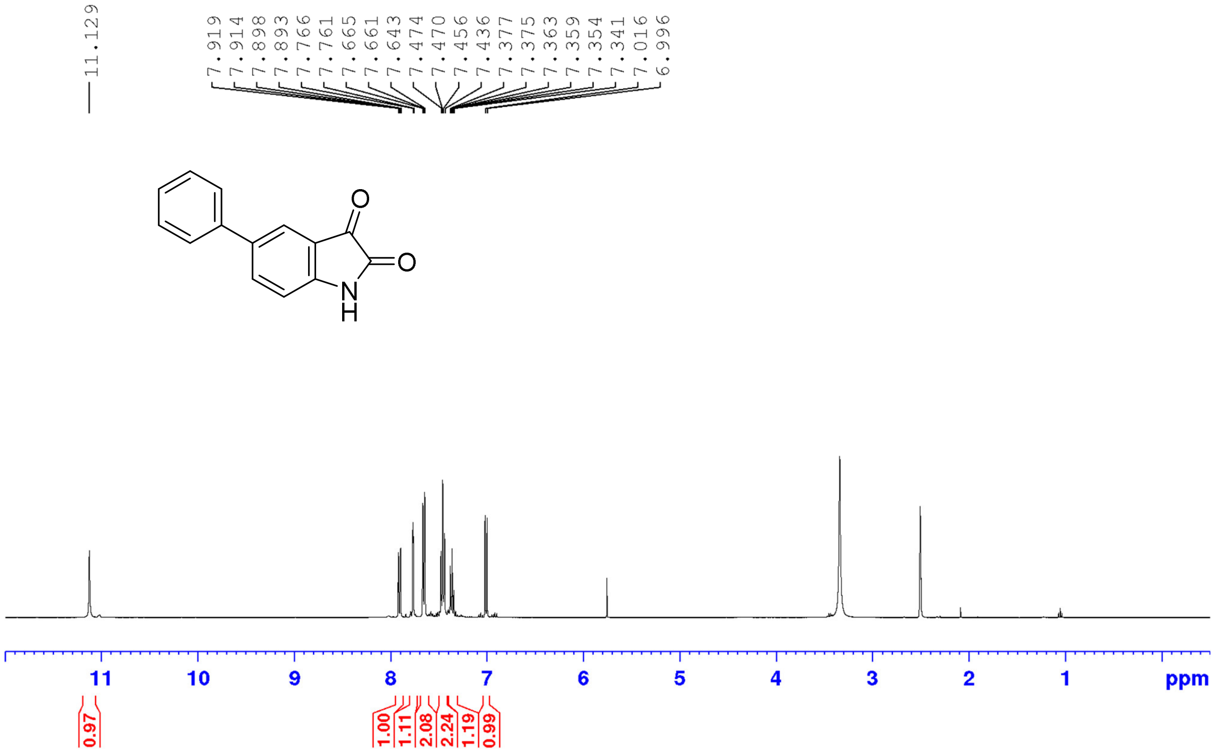

^{13}C NMR (100 MHz, DMSO-d_6):

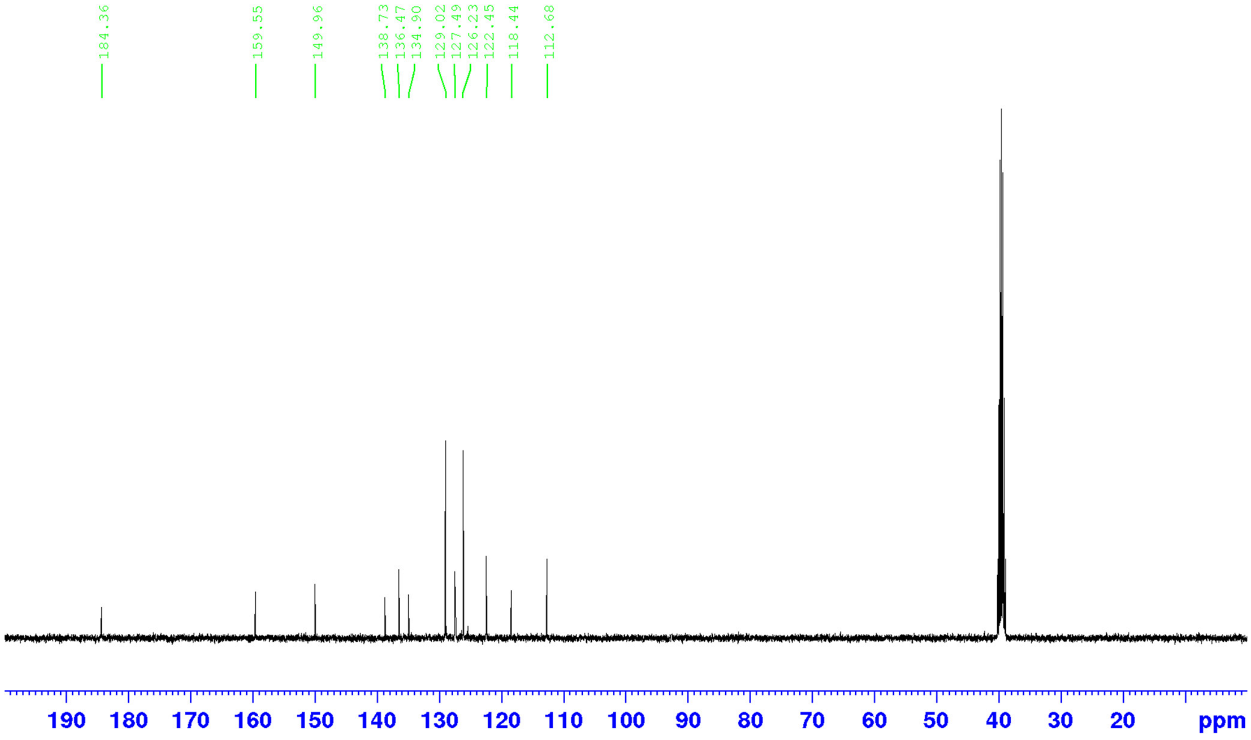

5-(4-Fluorophenyl)indoline-2,3-dione (**7b**)

^{1}H NMR (400 MHz, DMSO-d_6):

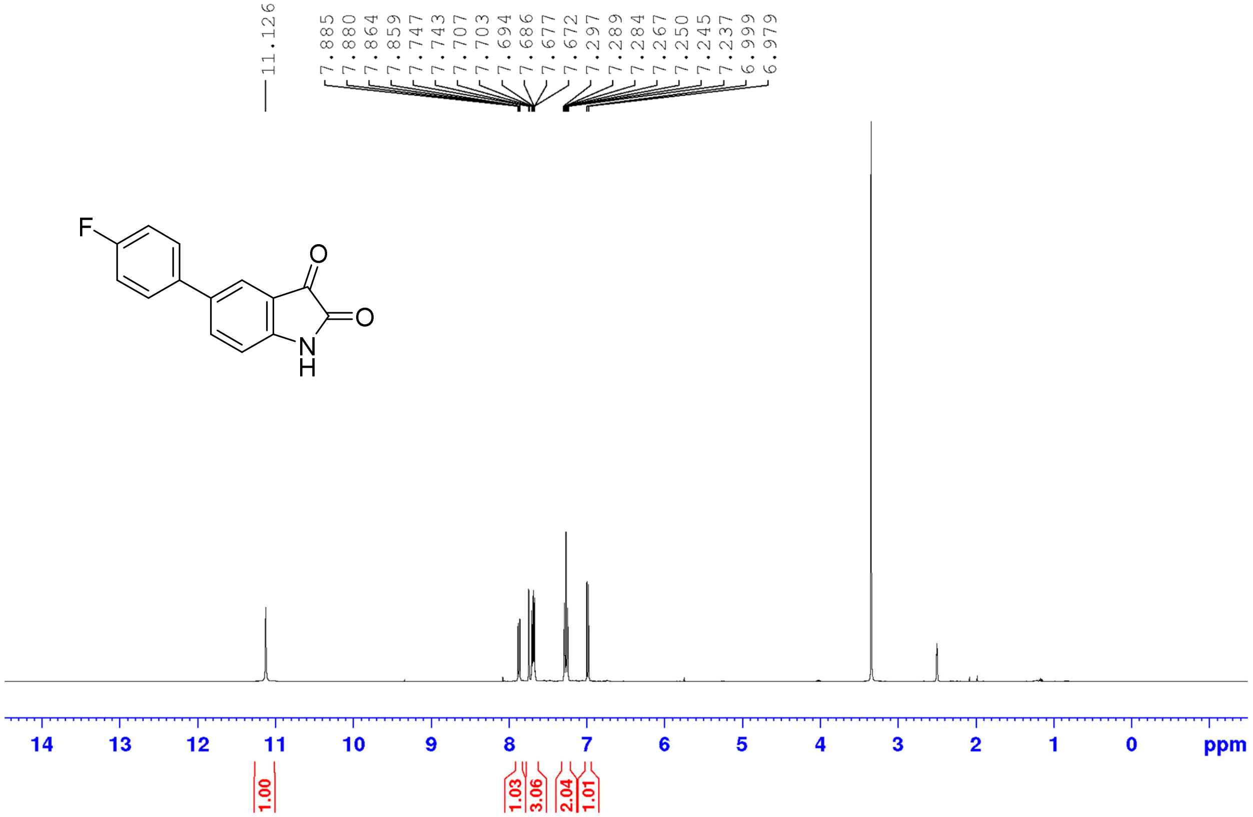

^{13}C NMR (100 MHz, DMSO-d_6):

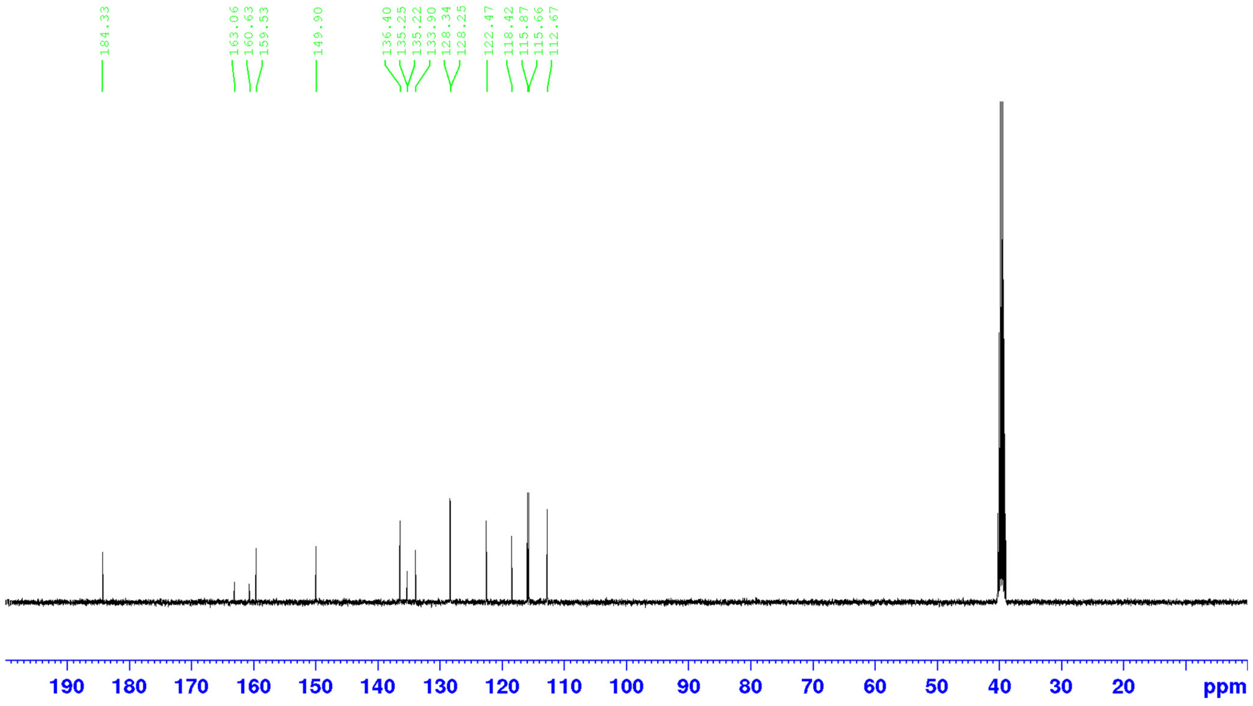

5-(4-Chlorophenyl)indoline-2,3-dione (**7c**)

^{1}H NMR (400 MHz, DMSO-d_6):

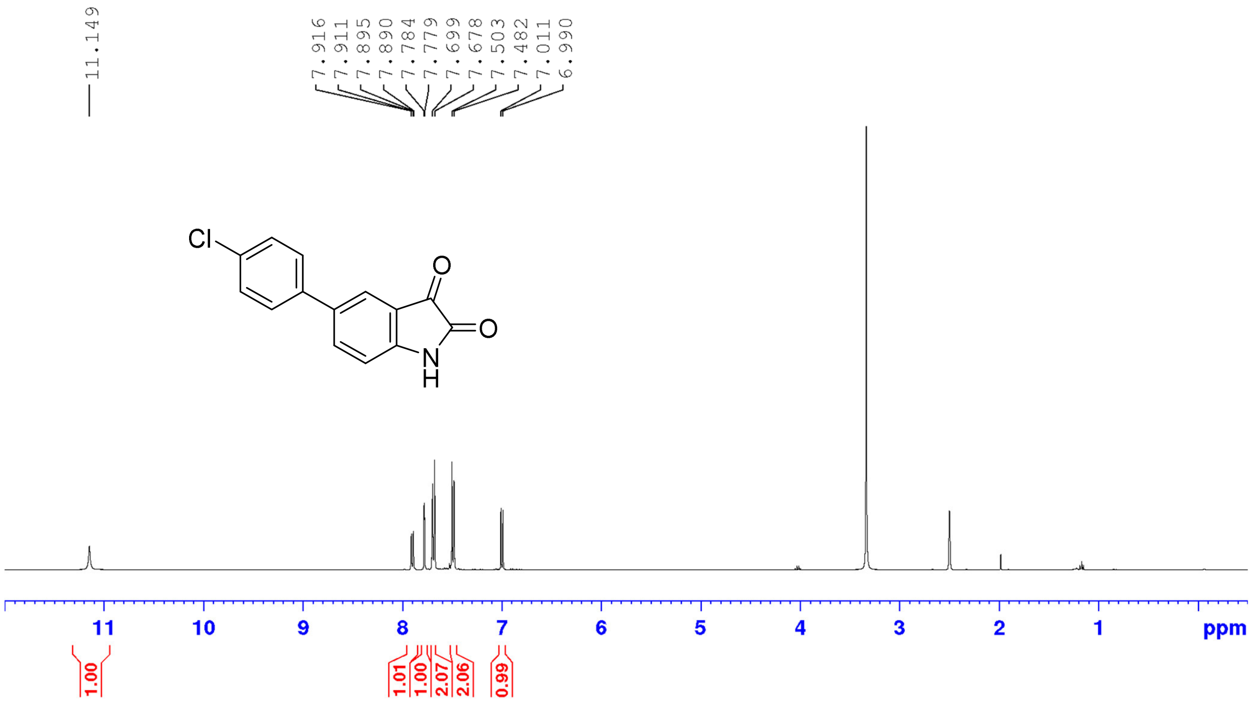

^{13}C NMR (100 MHz, DMSO-d_6):

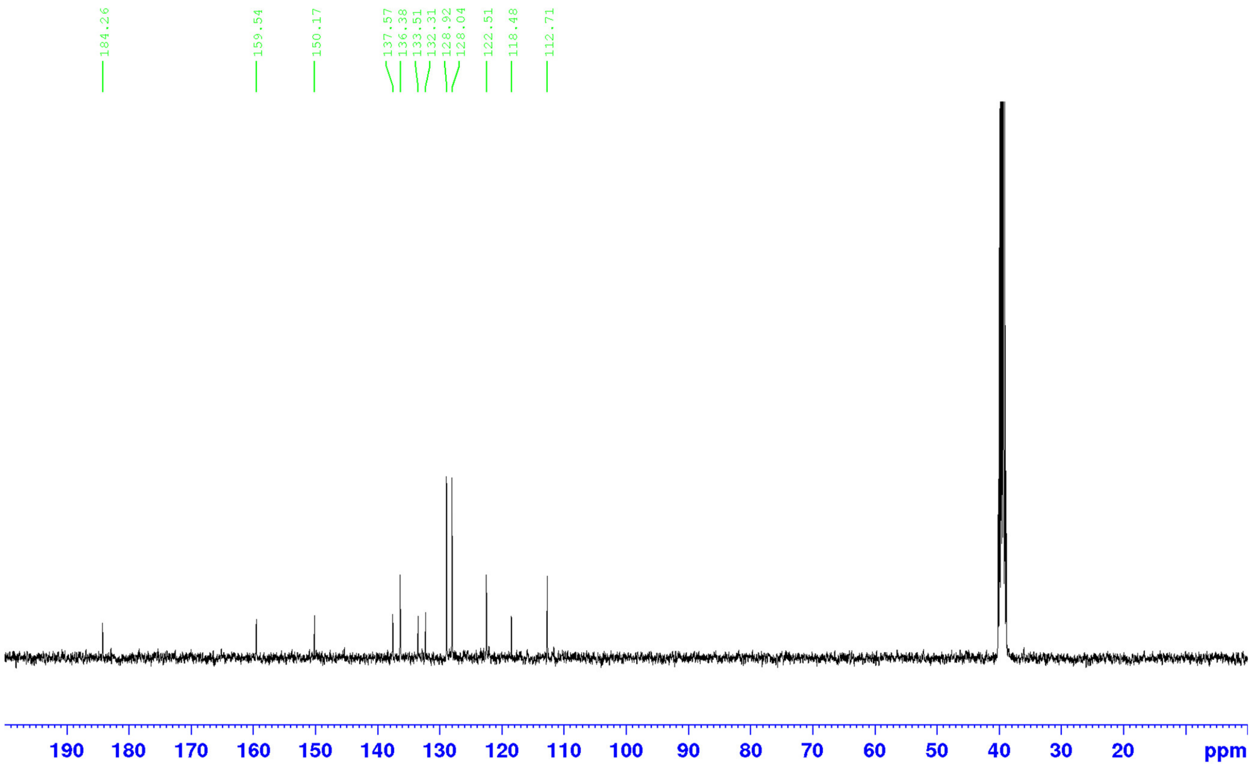

5-(Naphthalen-2-yl)indoline-2,3-dione (**7d**)

^{1}H NMR (600 MHz, DMSO-d_6):

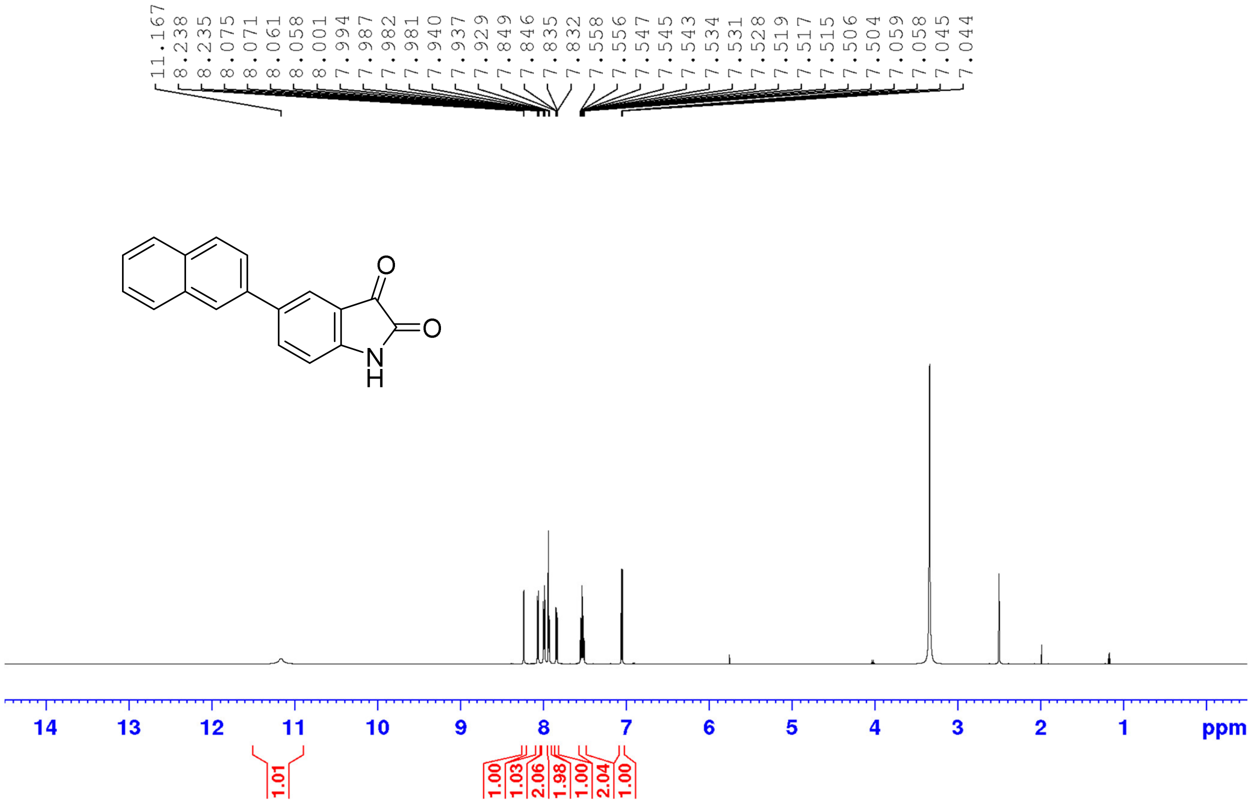

^{13}C NMR (150 MHz, DMSO-d_6):

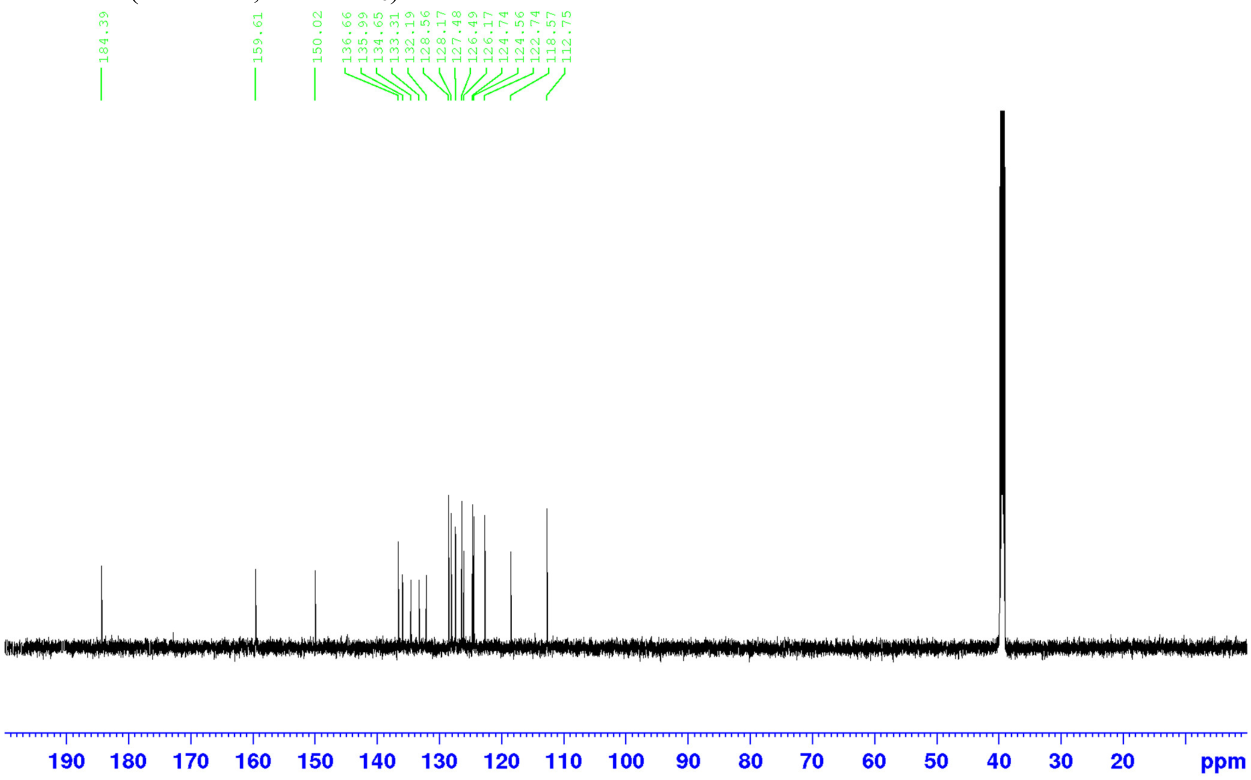

1-(Octylsulfonyl)-5-phenylindoline-2,3-dione (**8a**)

^{1}H NMR (400 MHz, DMSO-d_6):

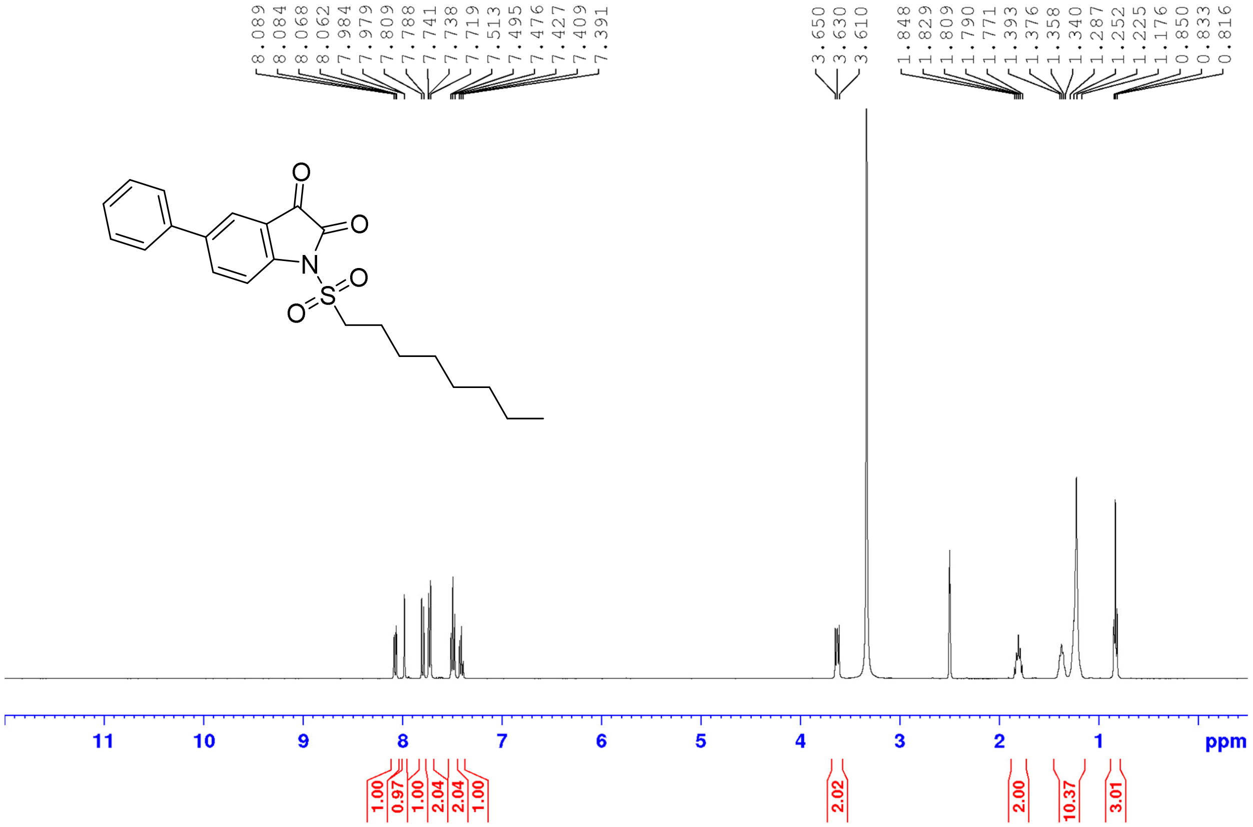

^{13}C NMR (100 MHz, DMSO-d_6):

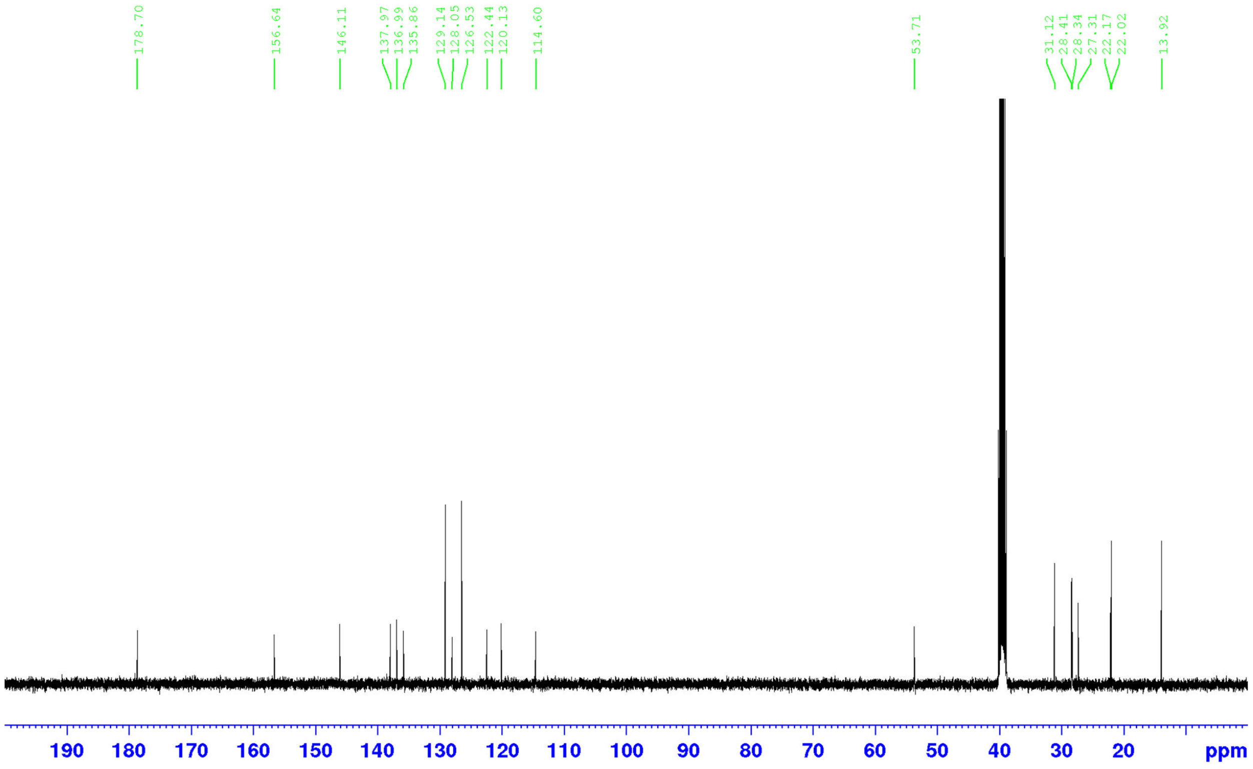

5-(4-Fluorophenyl)-1-(octylsulfonyl)indoline-2,3-dione (**8b**)

^{1}H NMR (400 MHz, DMSO-d_6):

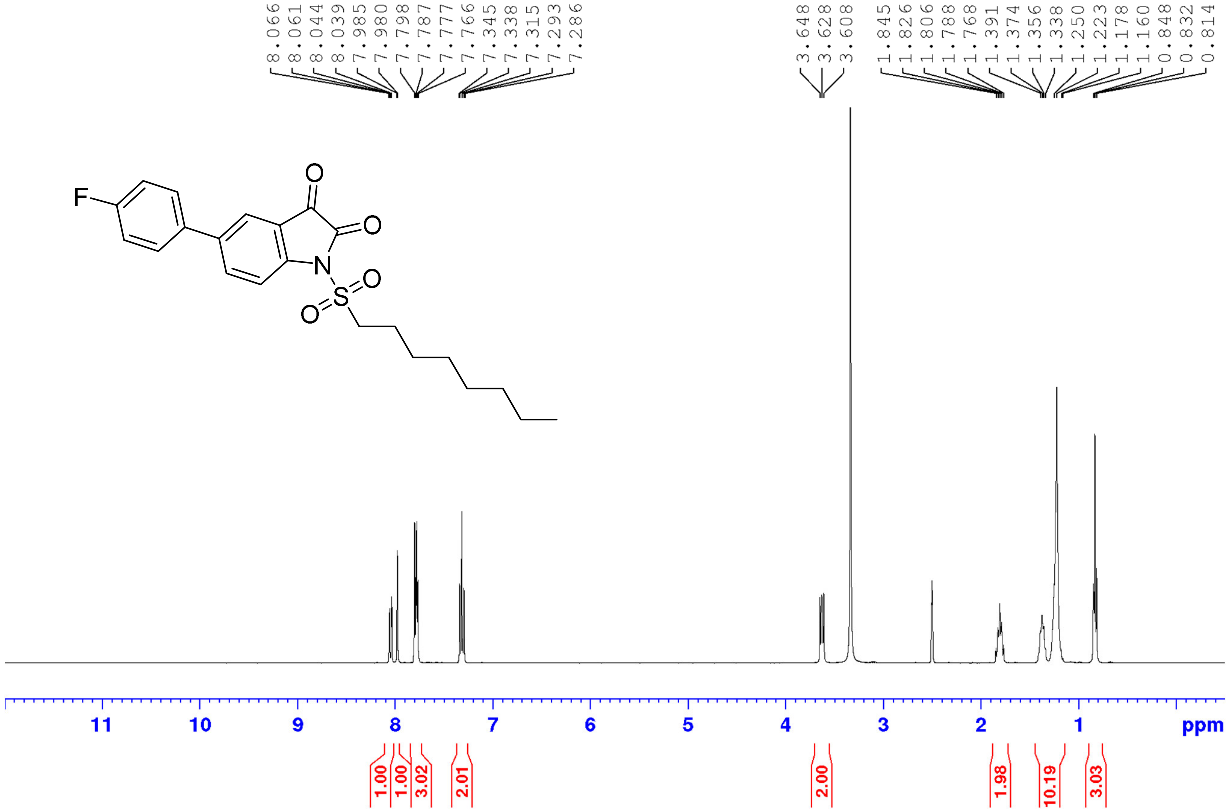

^{13}C NMR (100 MHz, DMSO-d_6):

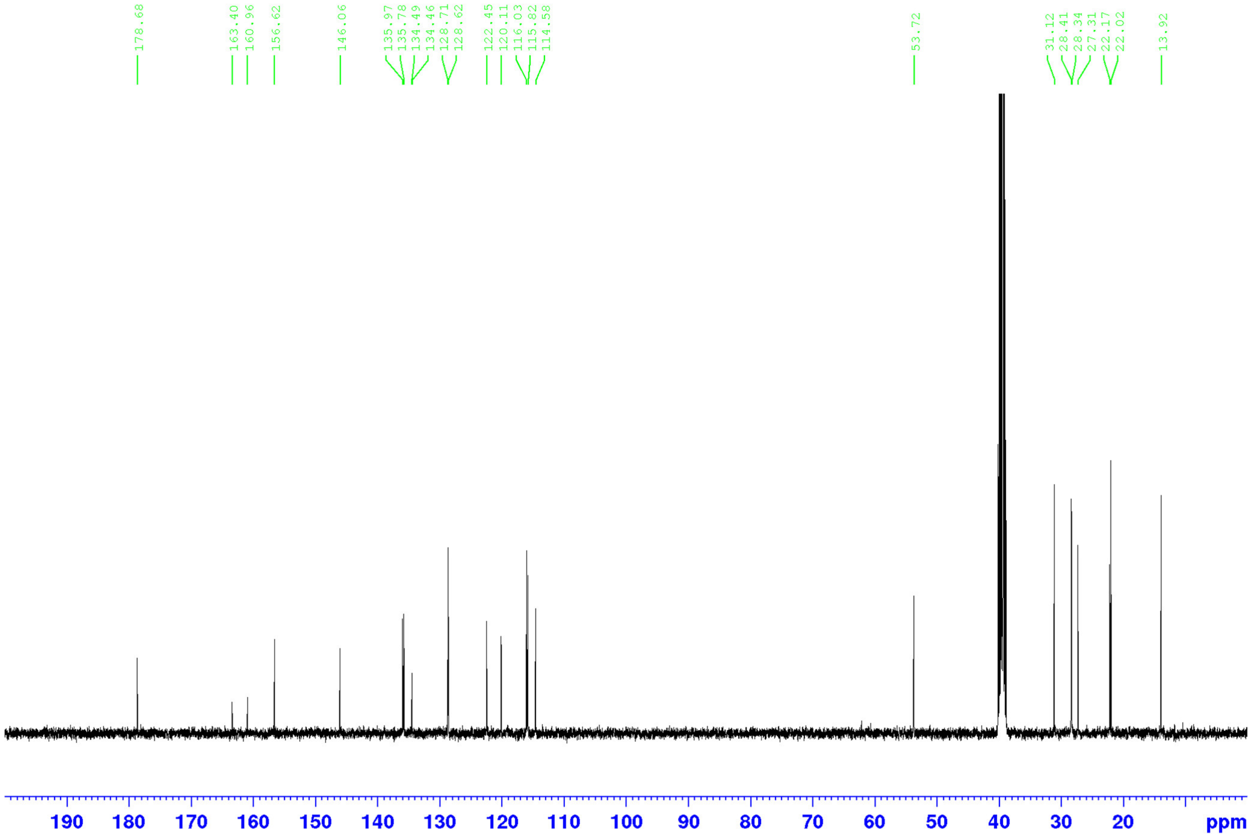

5-(4-Chlorophenyl)-1-(octylsulfonyl)indoline-2,3-dione (**8c**)

^{1}H NMR (400 MHz, DMSO-d_6):

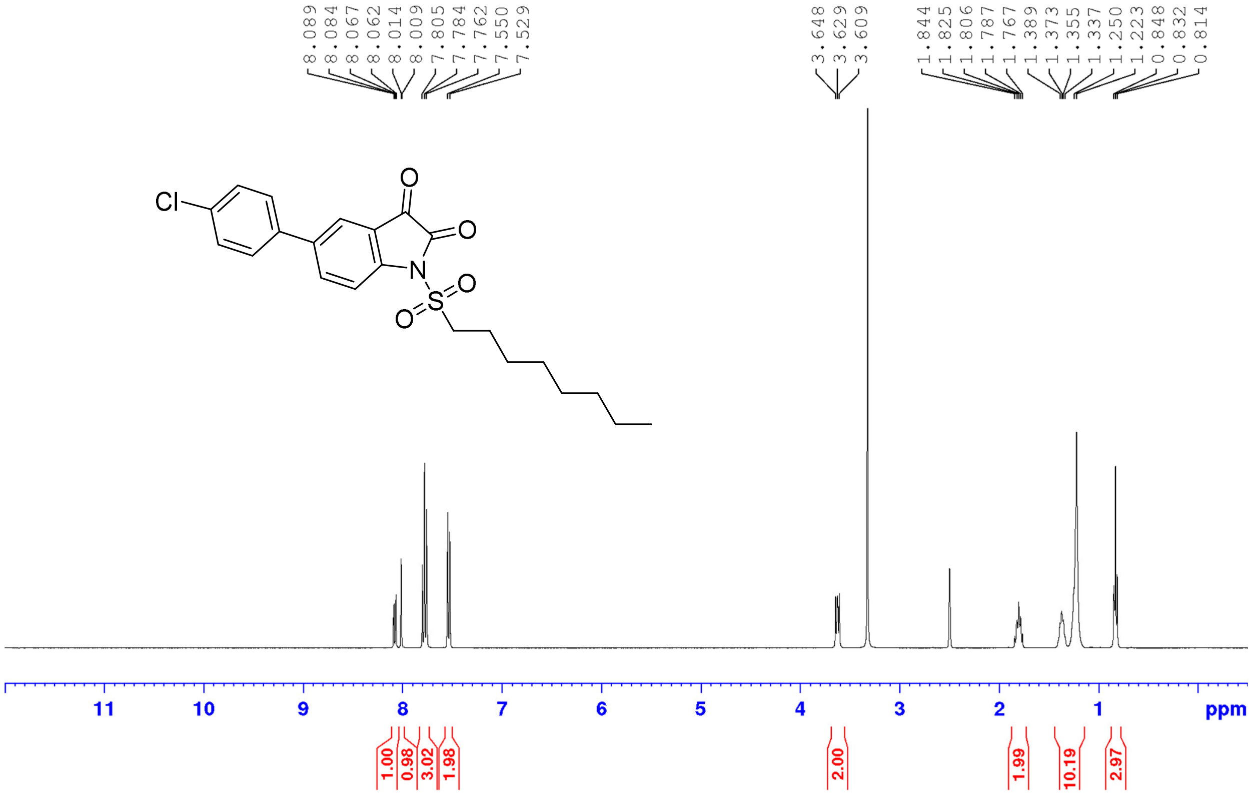

^{13}C NMR (100 MHz, DMSO-d_6):

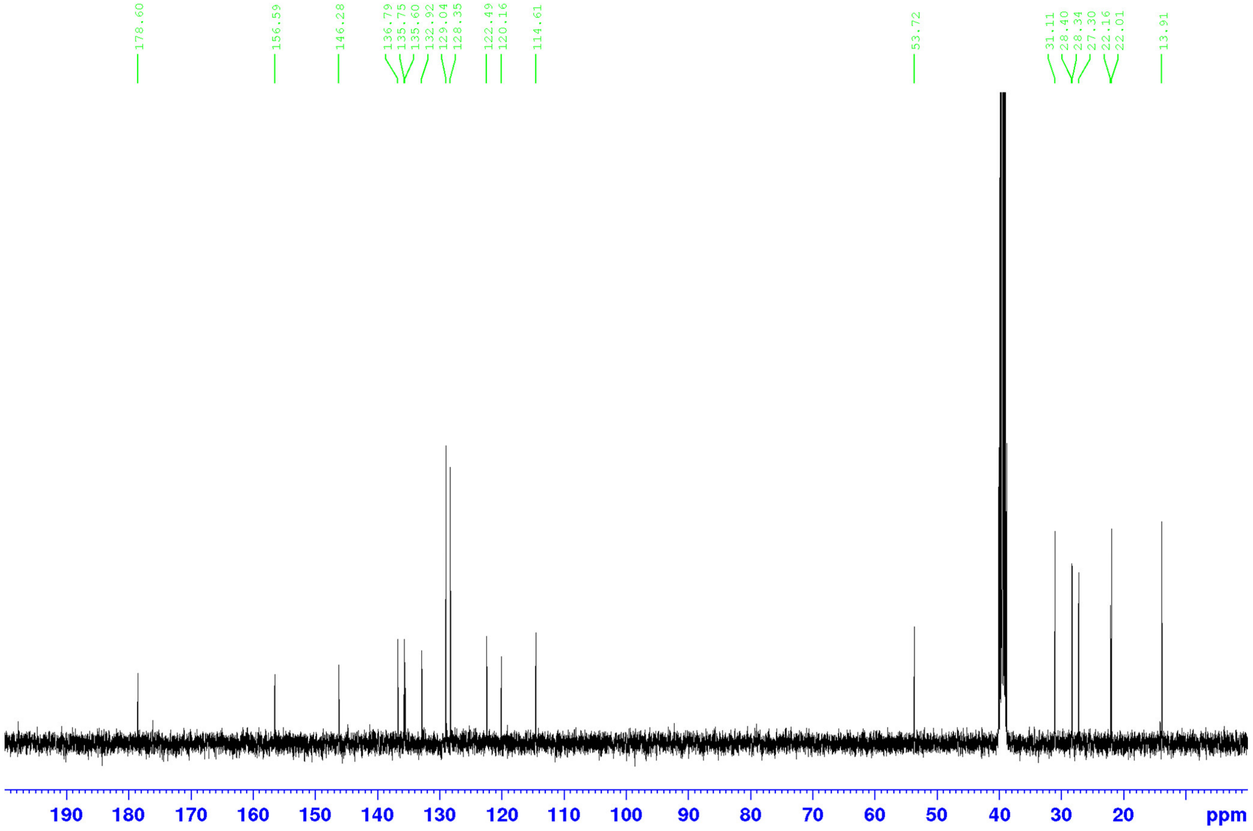

5-(Naphthalen-2-yl)-1-(octylsulfonyl)indoline-2,3-dione (**8d**)

^{1}H NMR (400 MHz, DMSO-d_6):

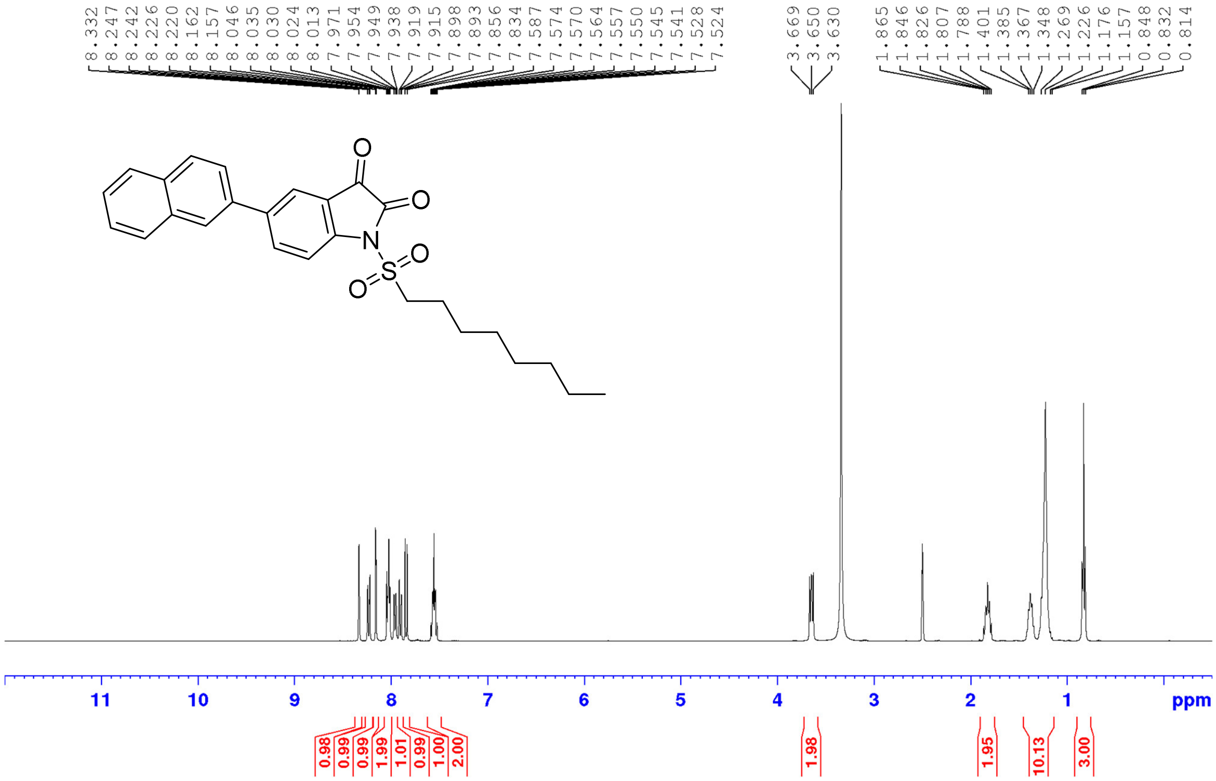

^{13}C NMR (100 MHz, DMSO-d_6):

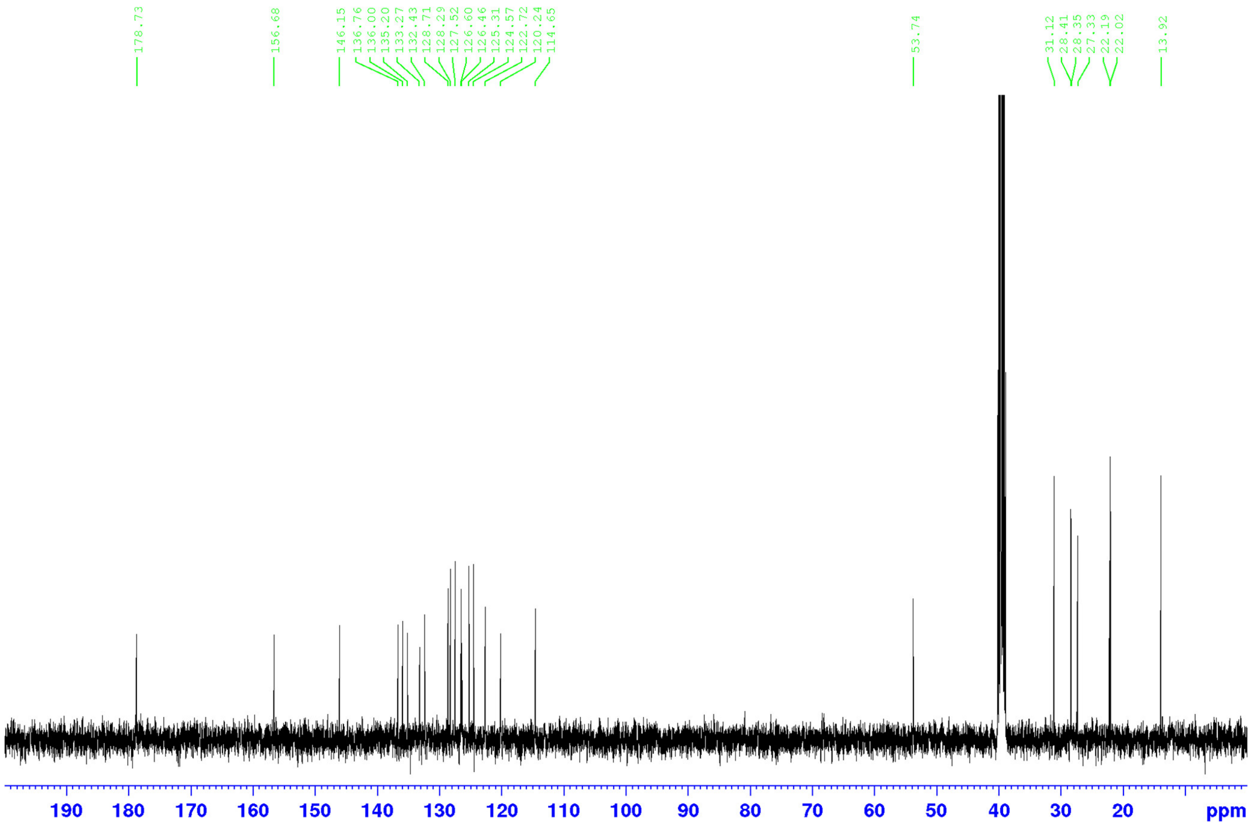

5-Butyl-1-(octylsulfonyl)indoline-2,3-dione (**8e**)

^{1}H NMR (400 MHz, DMSO-d_6):

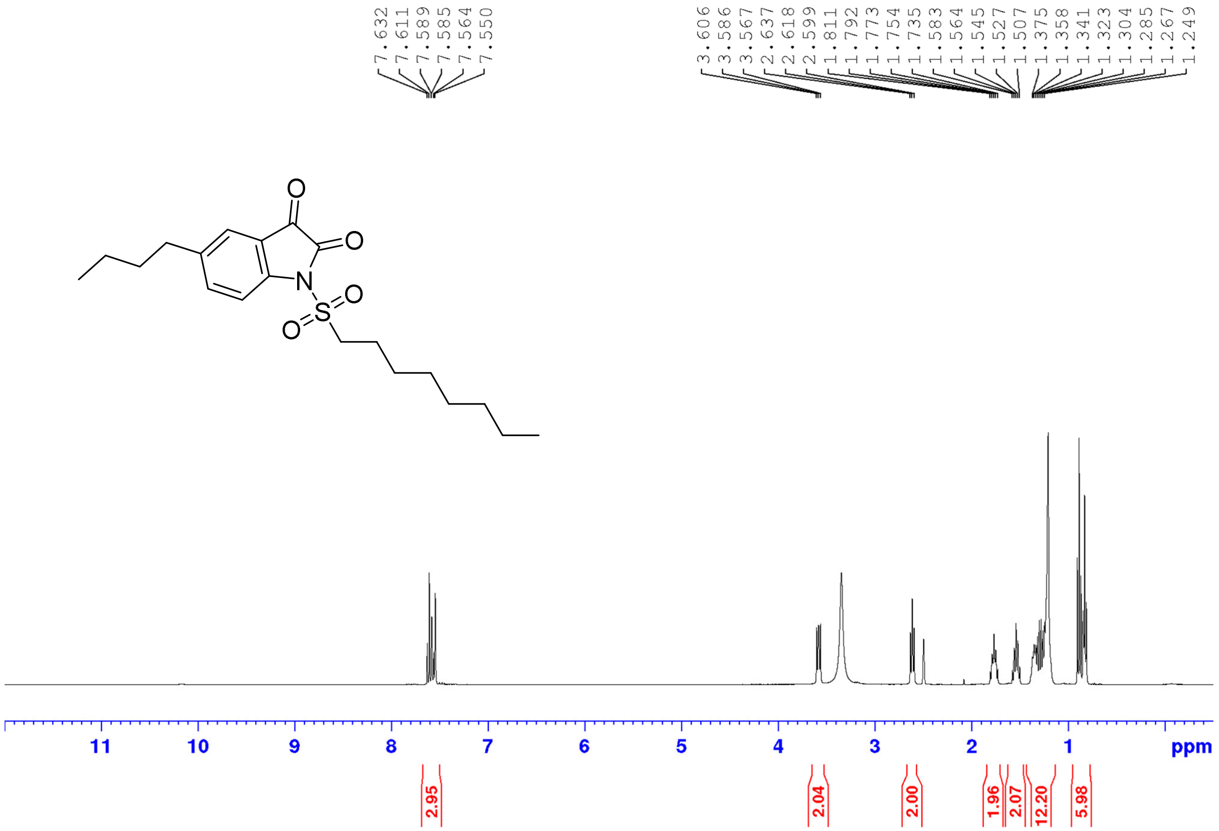

^{13}C NMR (100 MHz, DMSO-d_6):

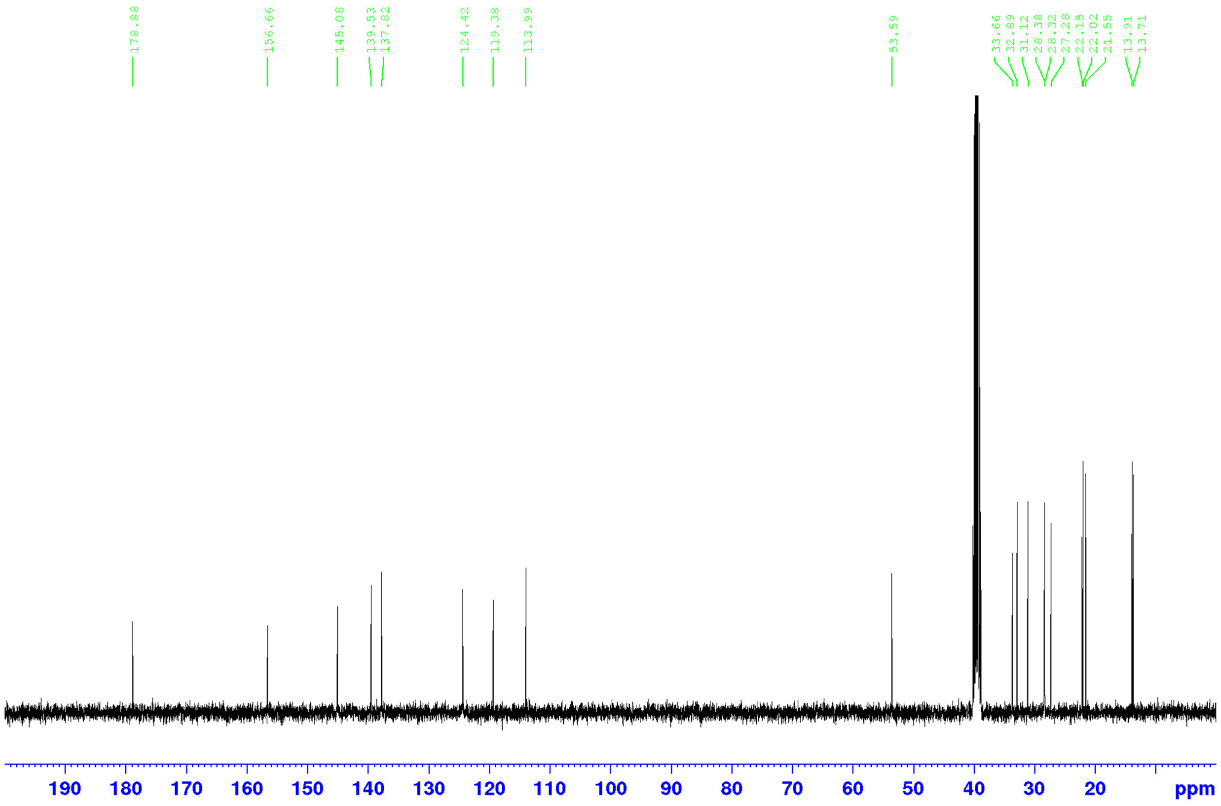

1-(Butylsulfonyl)-5-phenylindoline-2,3-dione (**9a**)

^{1}H NMR (400 MHz, DMSO-d_6):

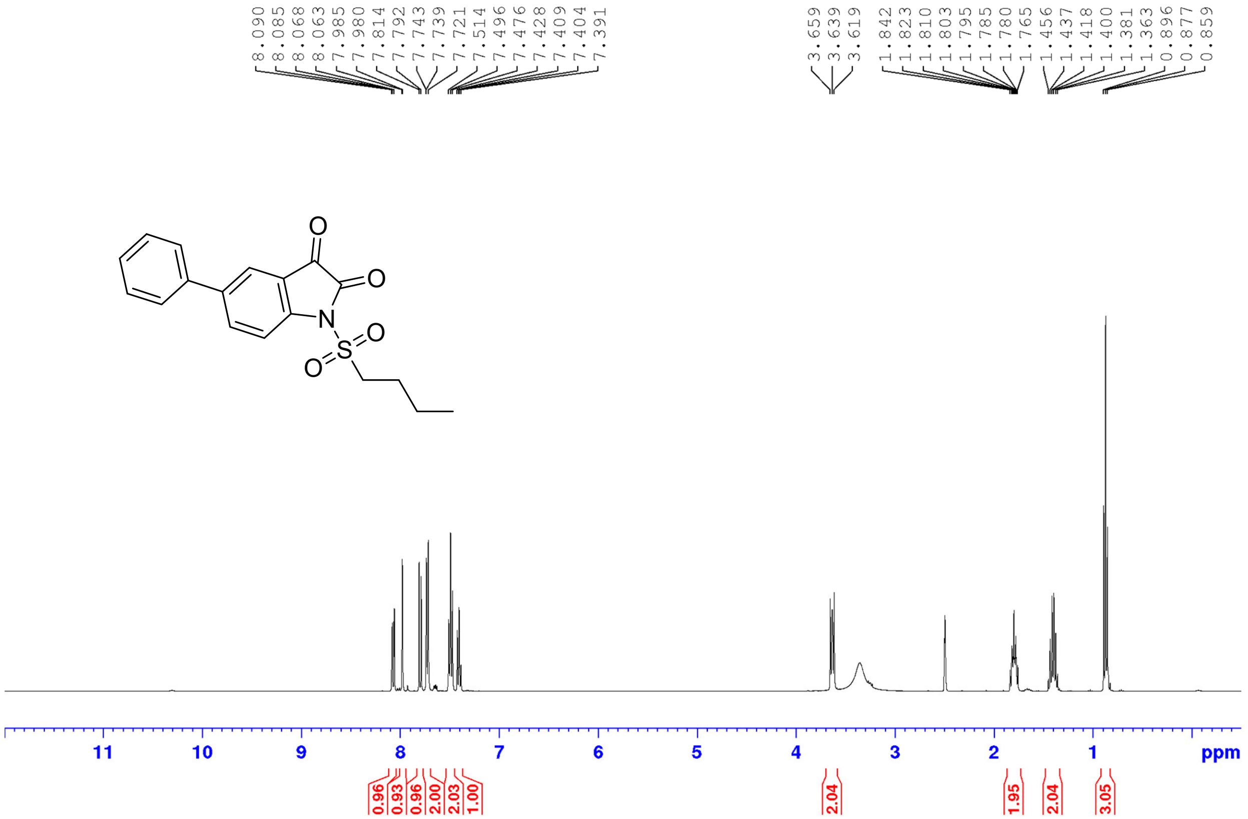

^{13}C NMR (100 MHz, DMSO-d_6):

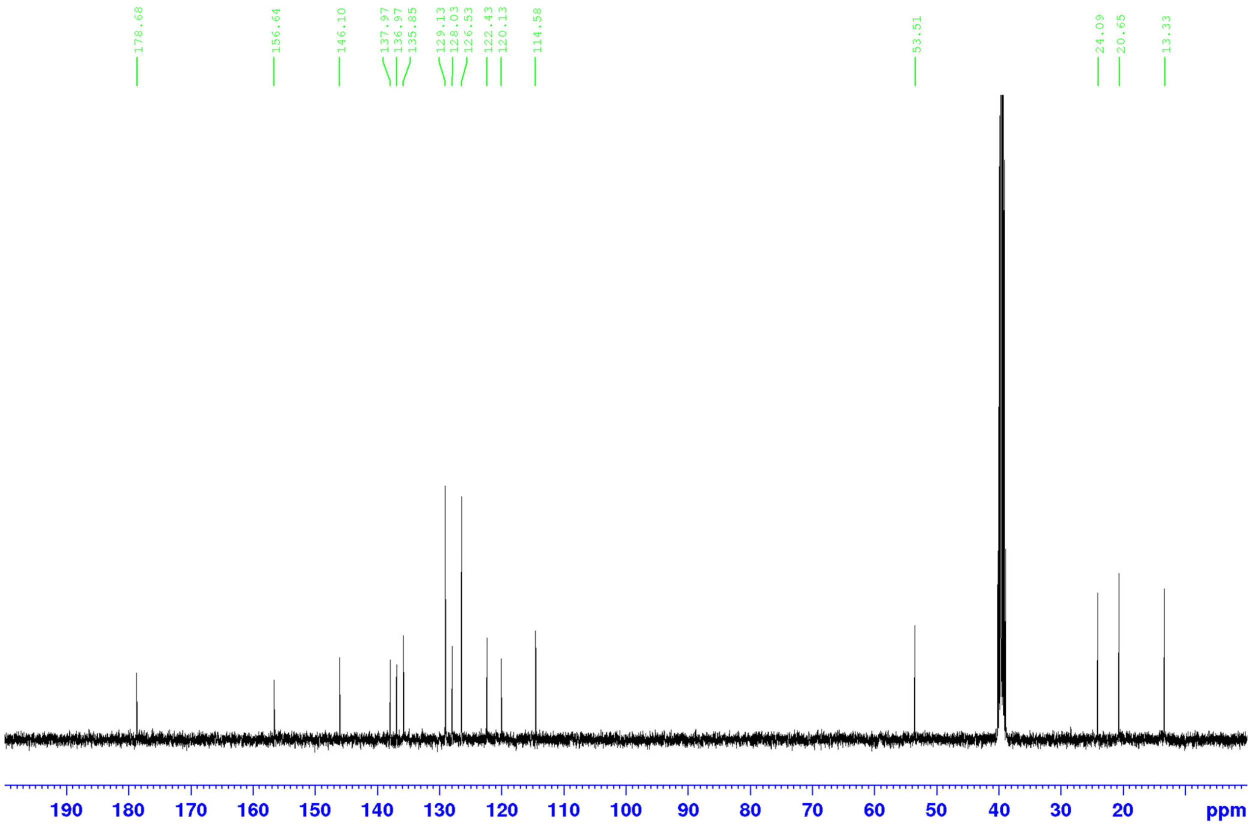

N-(3-(Dimethylamino)propyl)-2-(4-(octylsulfonamido)-[1,1'-biphenyl]-3-yl)-2-oxoacetamide (**11a**)

^{1}H NMR (400 MHz, $CDCl_3$):

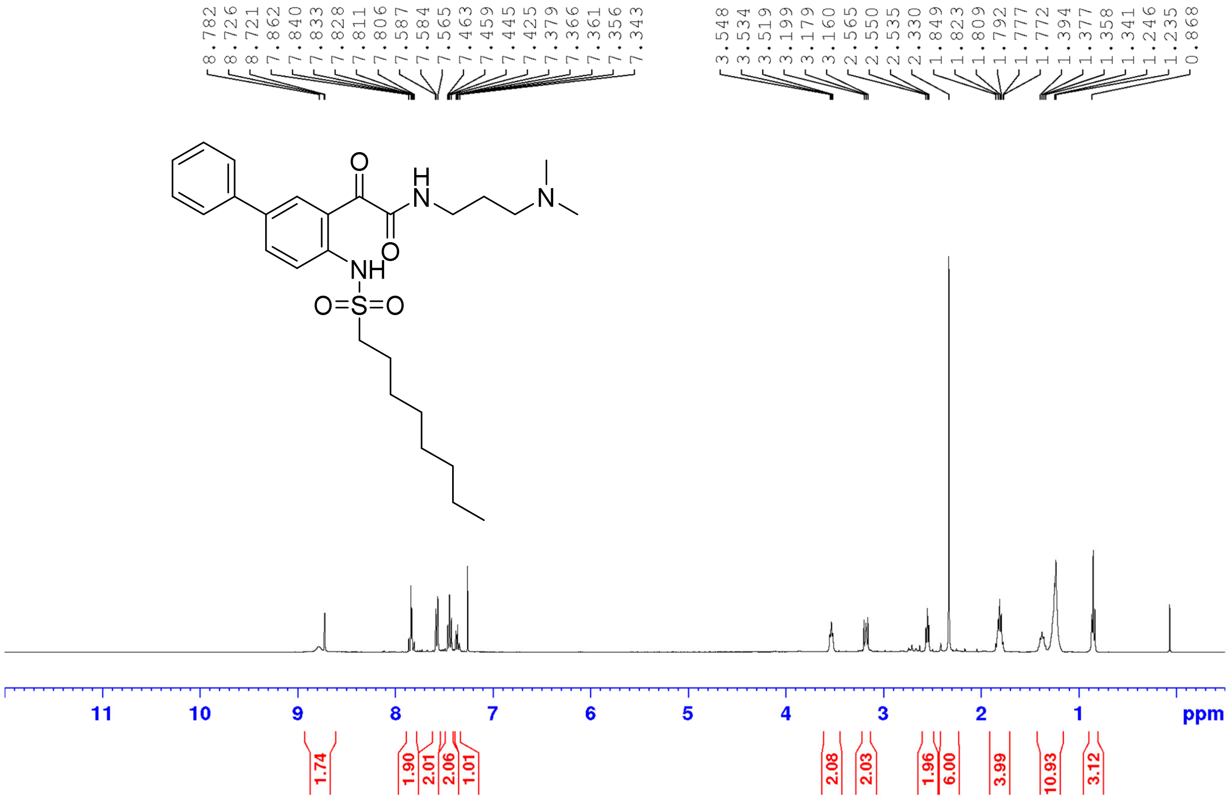

^{13}C NMR (100 MHz, $CDCl_3$):

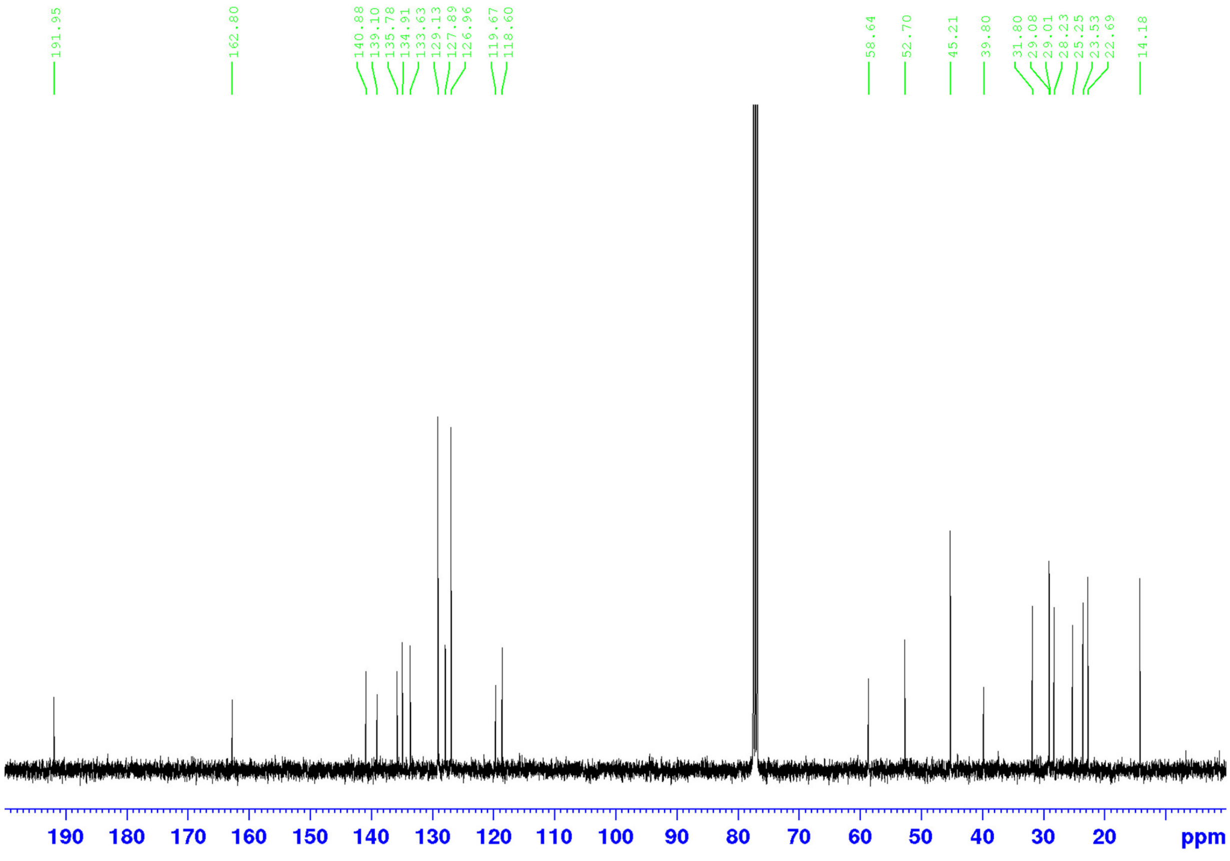

N-(3-(Dimethylamino)propyl)-2-(4'-fluoro-4-(octylsulfonamido)-[1,1'-biphenyl]-3-yl)-2-oxoacetamide (**11b**)

^{1}H NMR (400 MHz, $CDCl_3$):

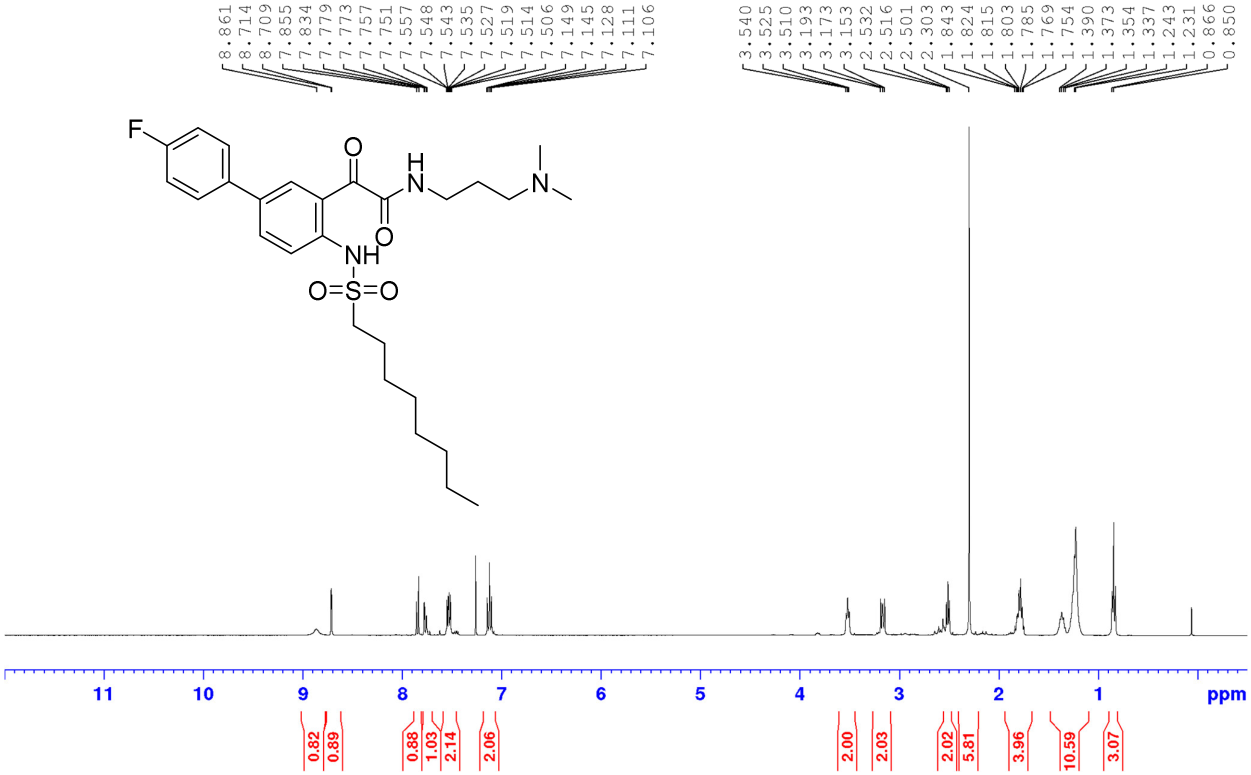

^{13}C NMR (100 MHz, $CDCl_3$):

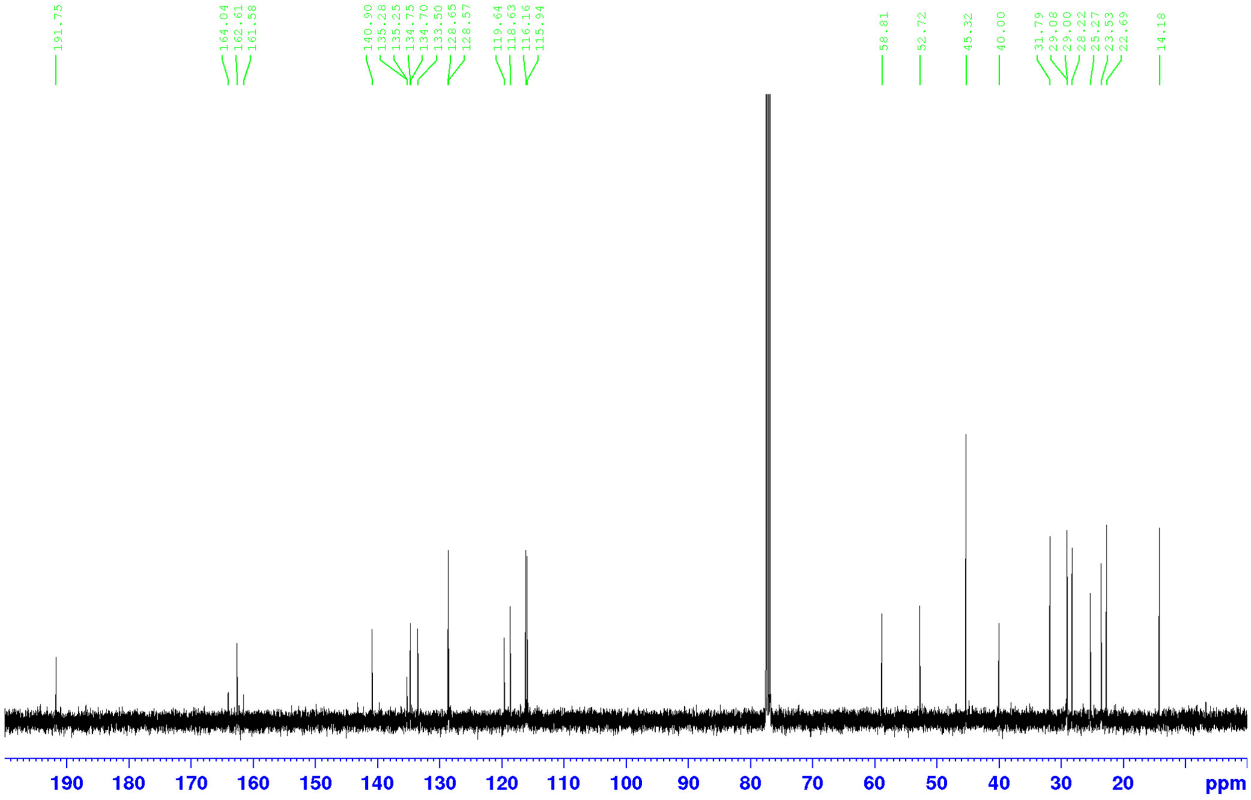

2-(4'-Chloro-4-(octylsulfonamido)-[1,1'-biphenyl]-3-yl)-*N*-(3-(dimethylamino)propyl)-2-oxoacetamide (**11c**)

^{1}H NMR (400 MHz, $CDCl_3$):

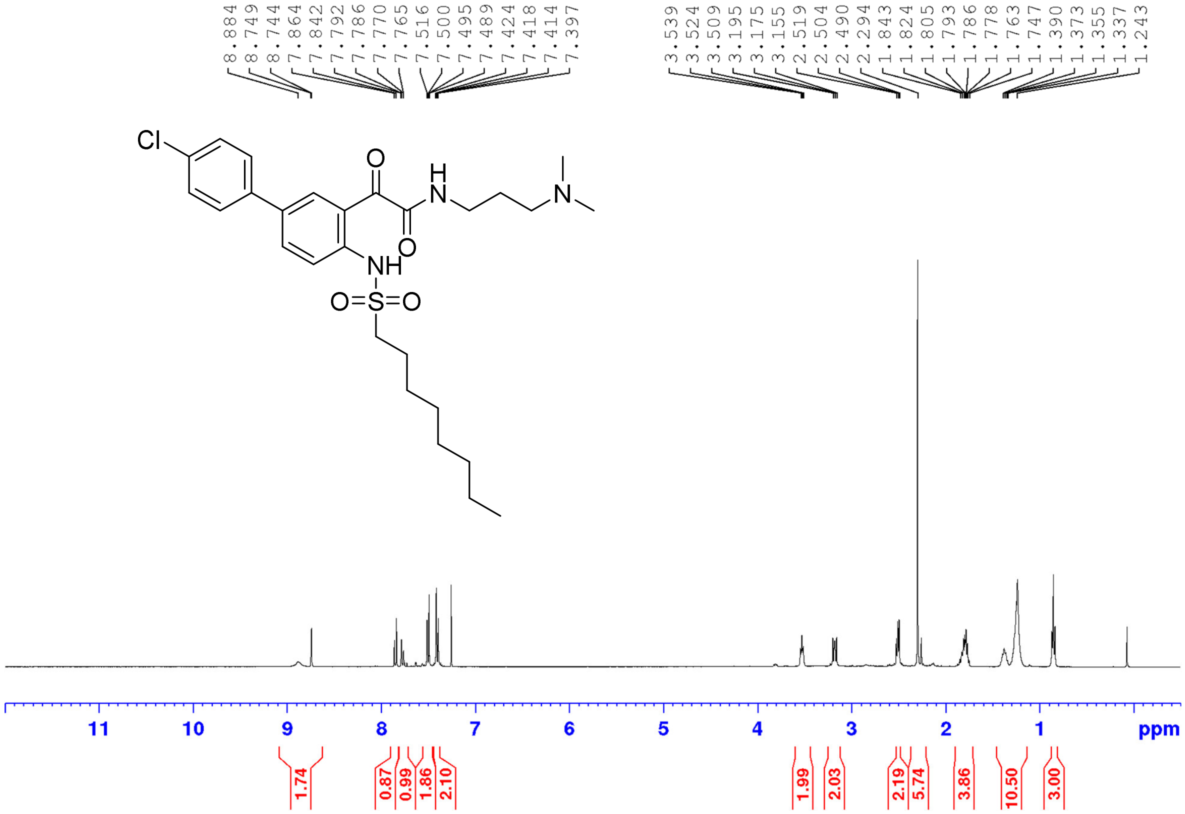

^{13}C NMR (100 MHz, $CDCl_3$):

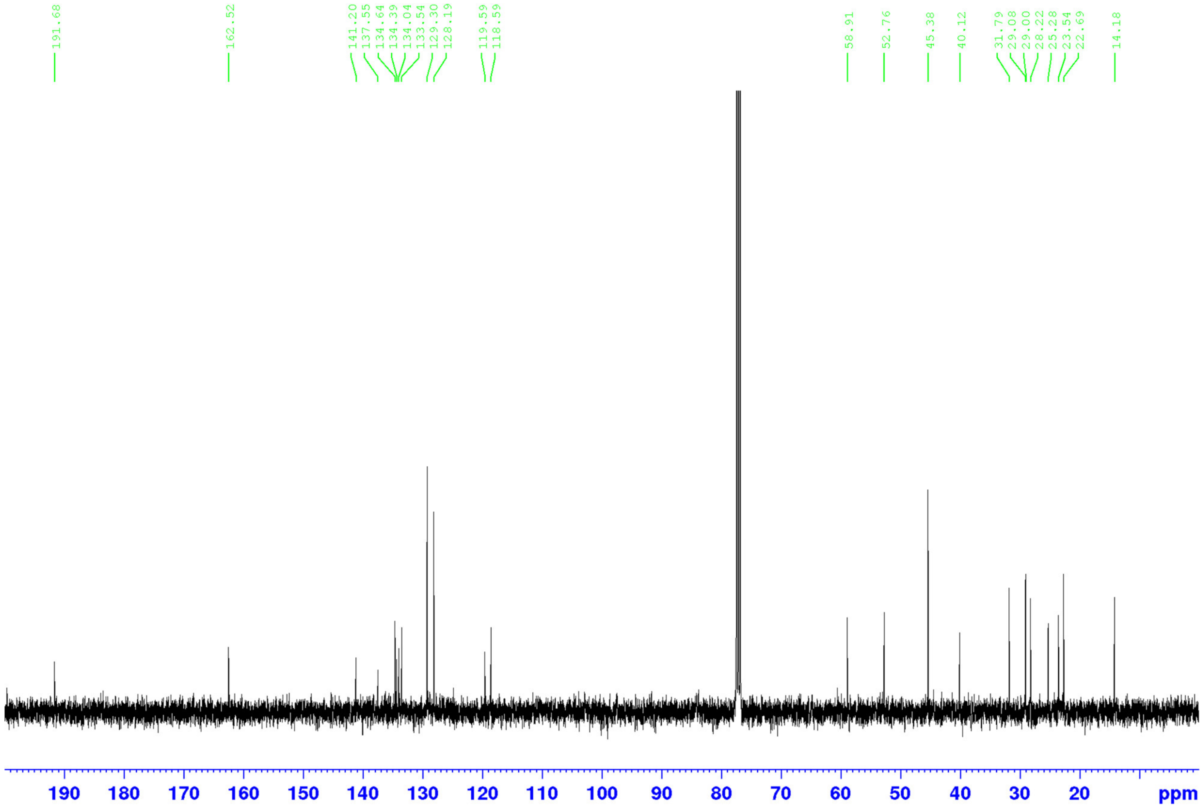

N-(3-(Dimethylamino)propyl)-2-(5-(naphthalen-2-yl)-2-(octylsulfonamido)phenyl)-2-oxoacetamide (**11d**)

^{1}H NMR (400 MHz, $CDCl_3$):

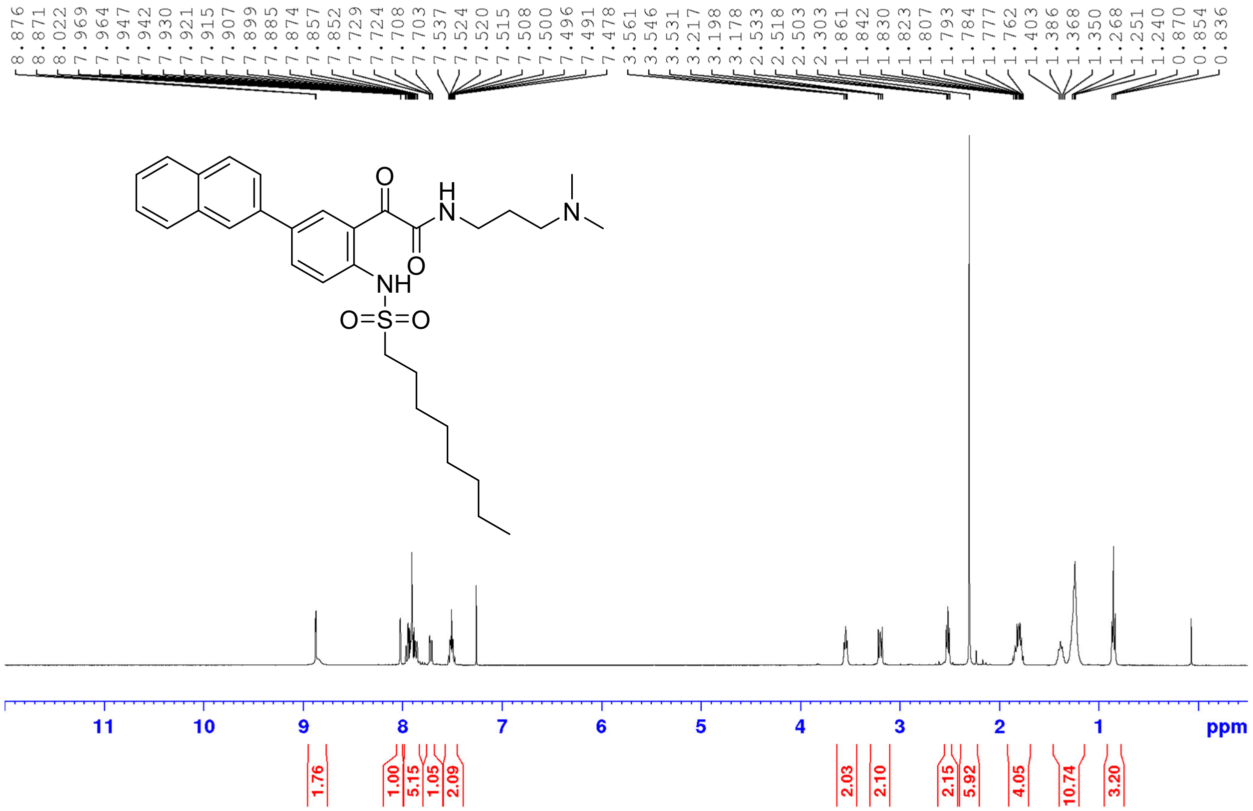

^{13}C NMR (100 MHz, $CDCl_3$):

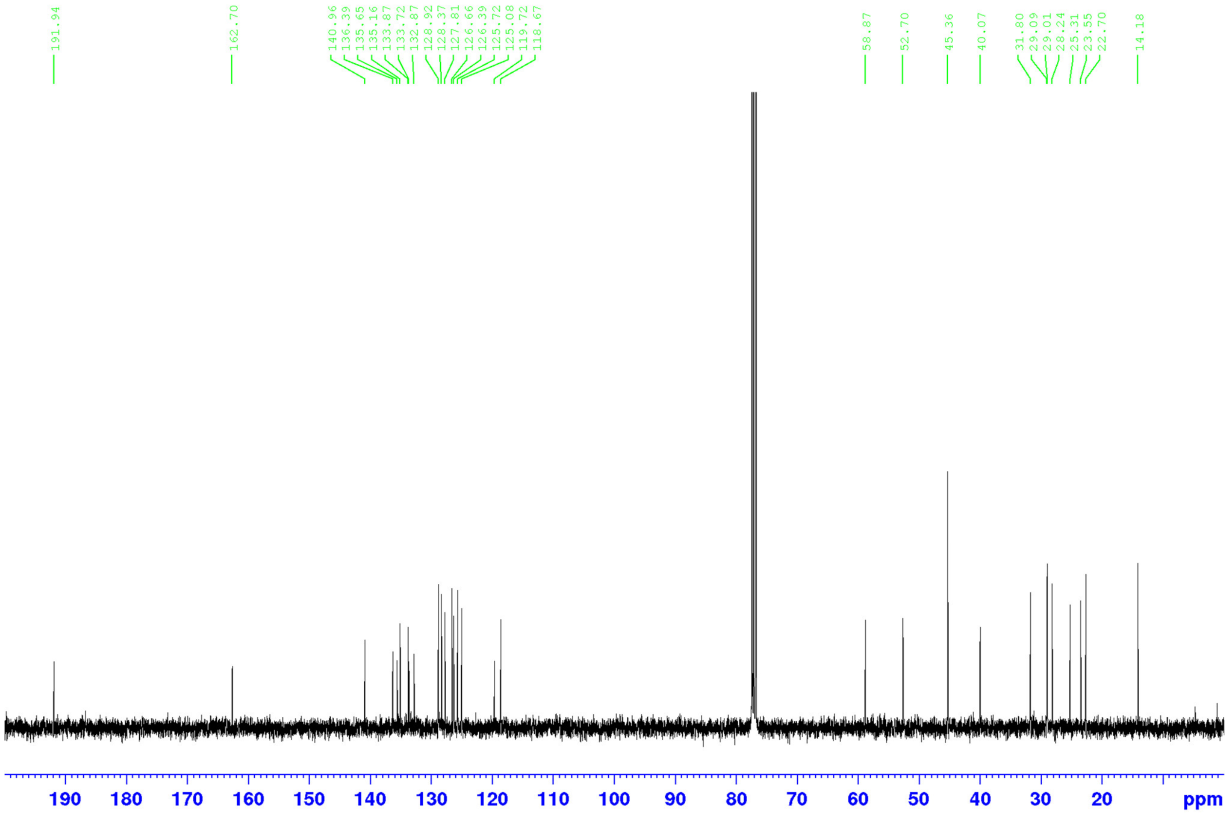

2-(5-Butyl-2-(octylsulfonamido)phenyl)-*N*-(3-(dimethylamino)propyl)-2-oxoacetamide (**11e**)

^{1}H NMR (400 MHz, $CDCl_3$):

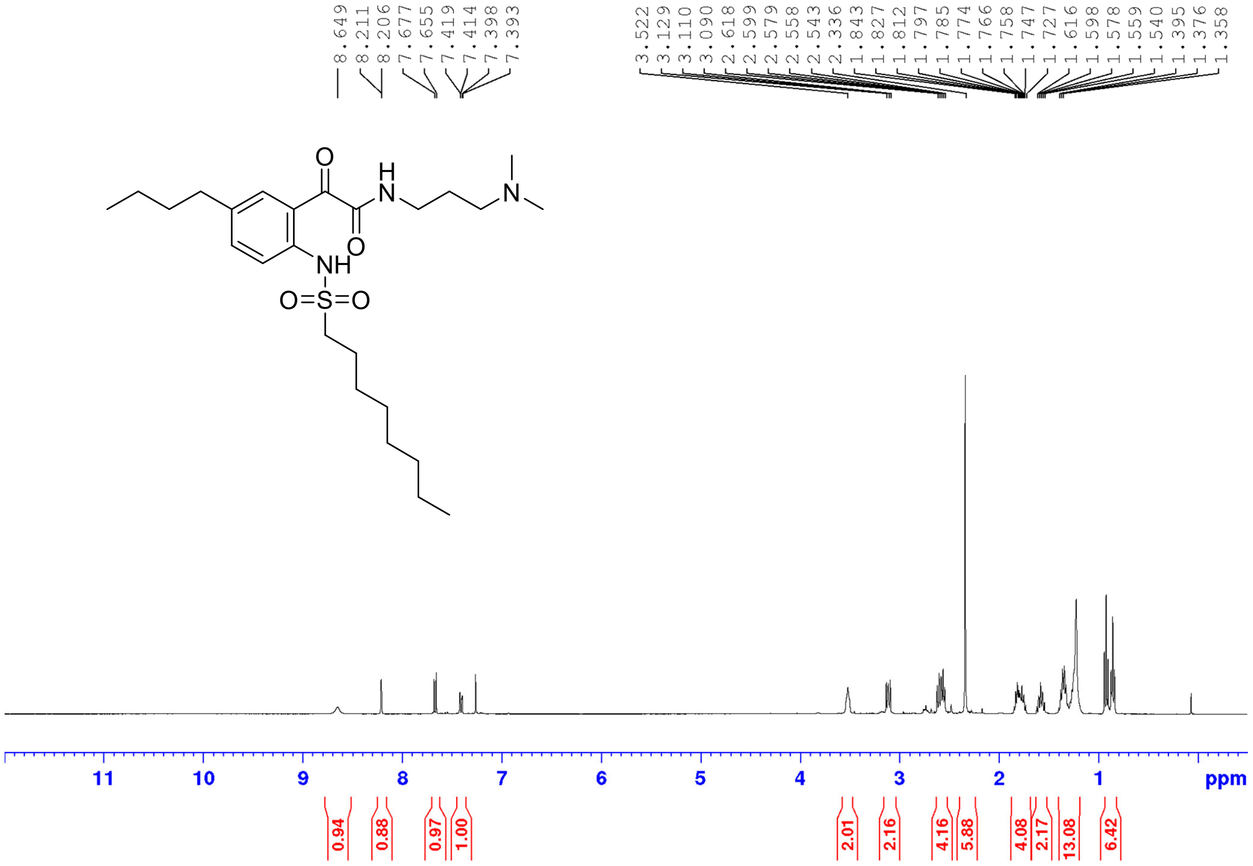

^{13}C NMR (100 MHz, $CDCl_3$):

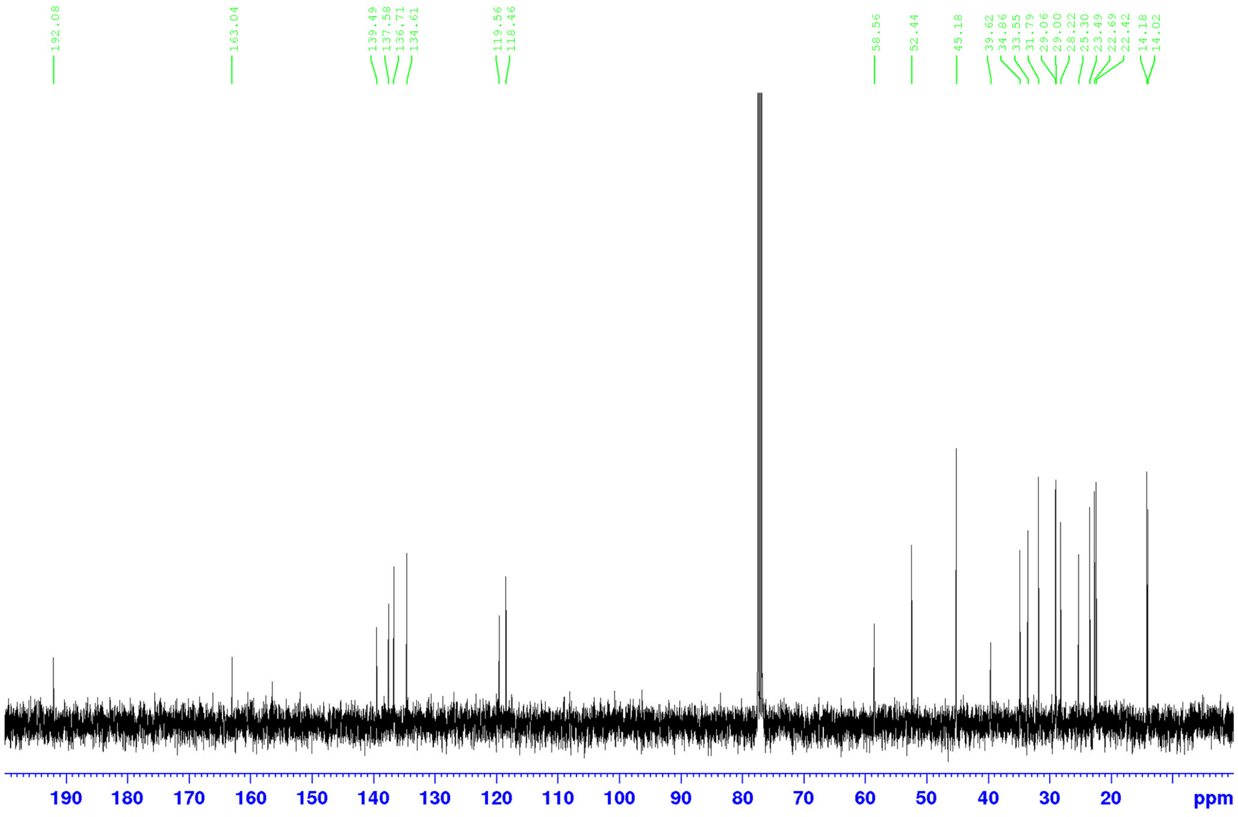

2-(4-(Butylsulfonamido)-[1,1'-biphenyl]-3-yl)-*N*-(3-(dimethylamino)propyl)-2-oxoacetamide (**12a**)

^{1}H NMR (400 MHz, $CDCl_3$):

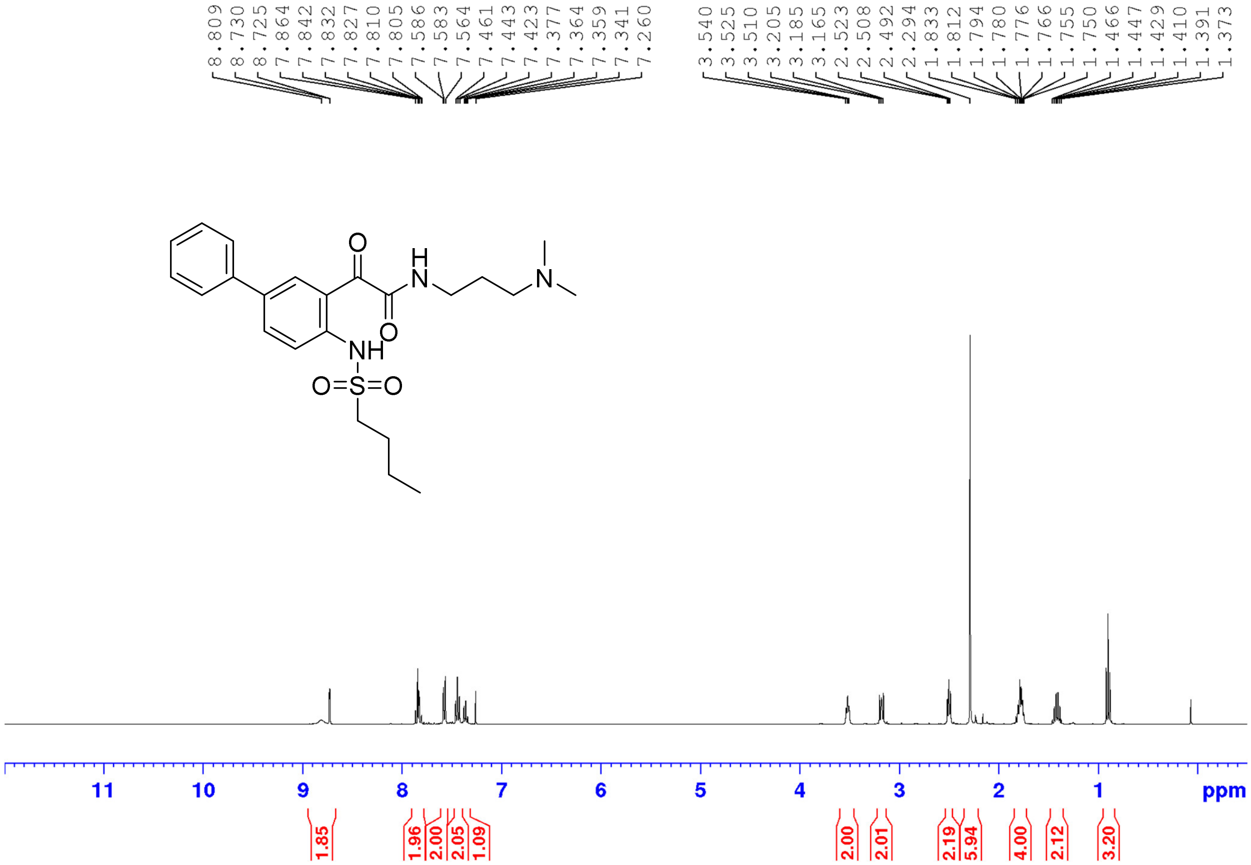

^{13}C NMR (100 MHz, $CDCl_3$):

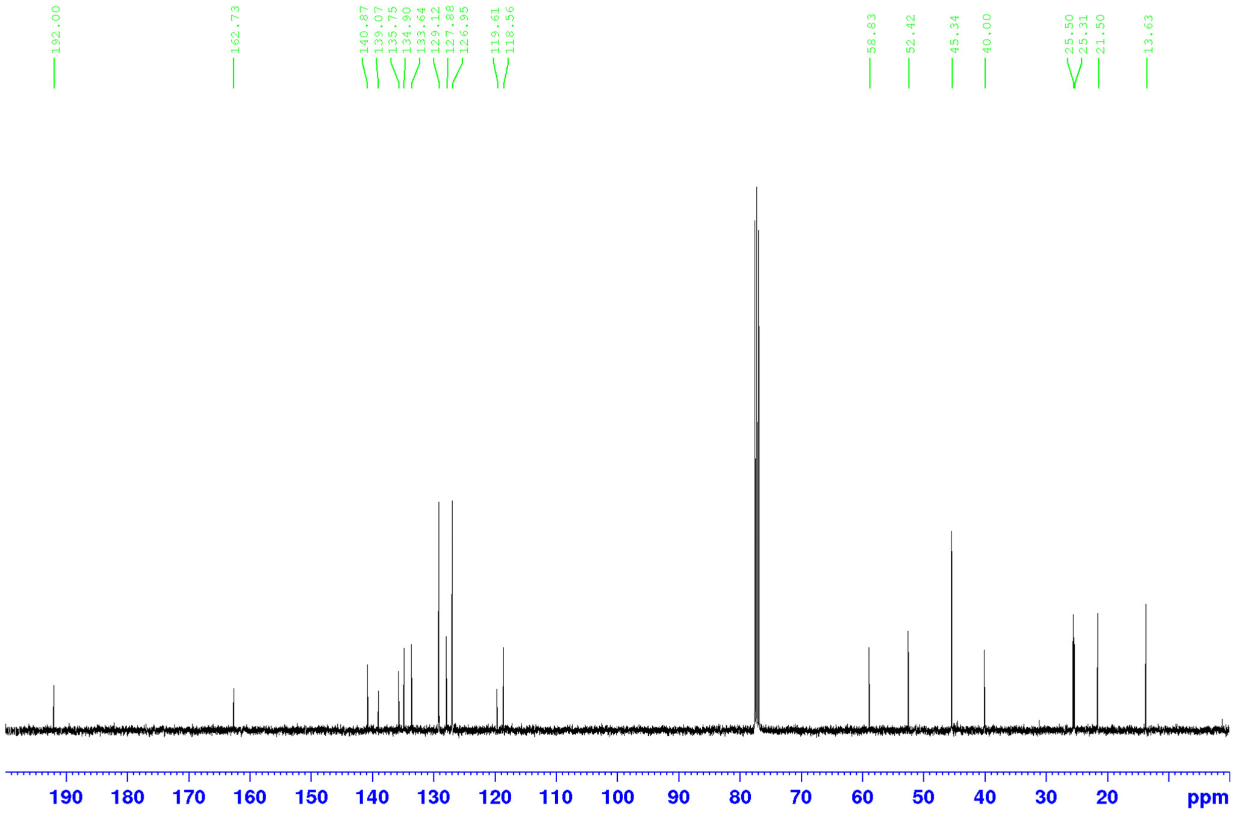

N,*N*-Dimethyl-3-(2-(4-(octylsulfonamido)-[1,1'-biphenyl]-3-yl)-2-oxoacetamido)propan-1-aminium chloride (**13a**)

^{1}H NMR (600 MHz, DMSO-d_6):

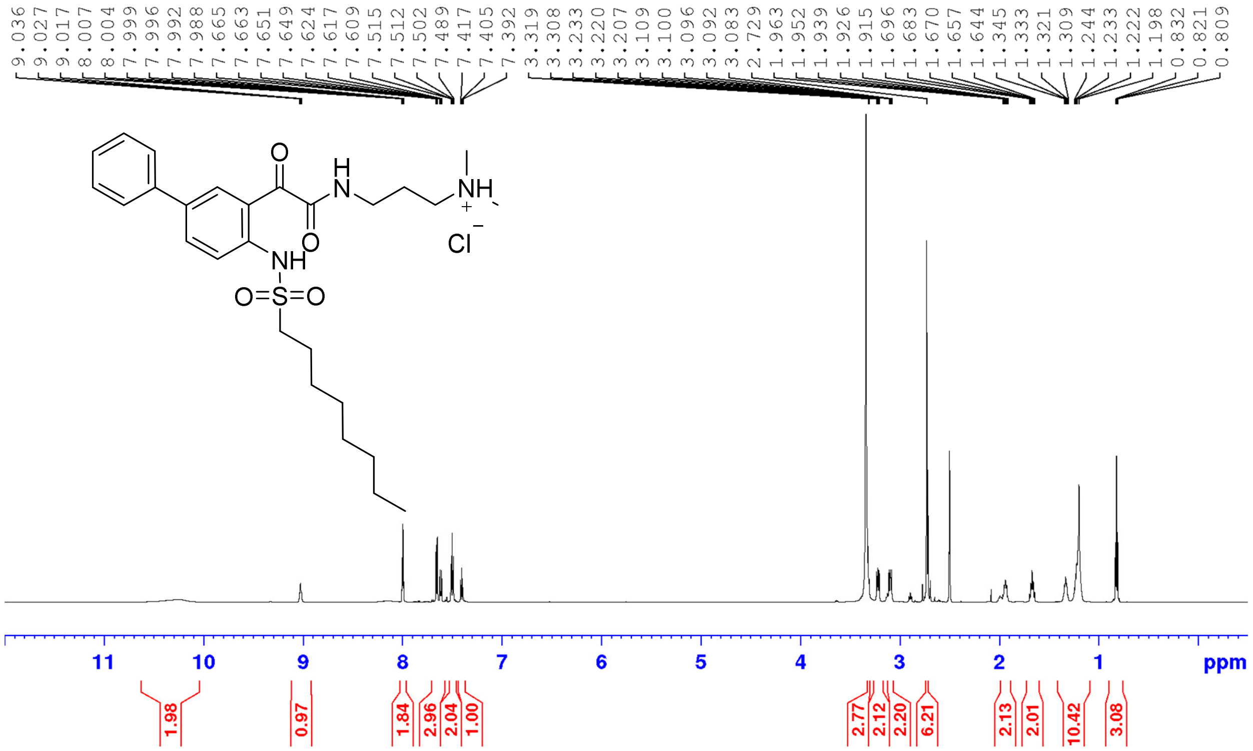

^{13}C NMR (150 MHz, DMSO-d_6):

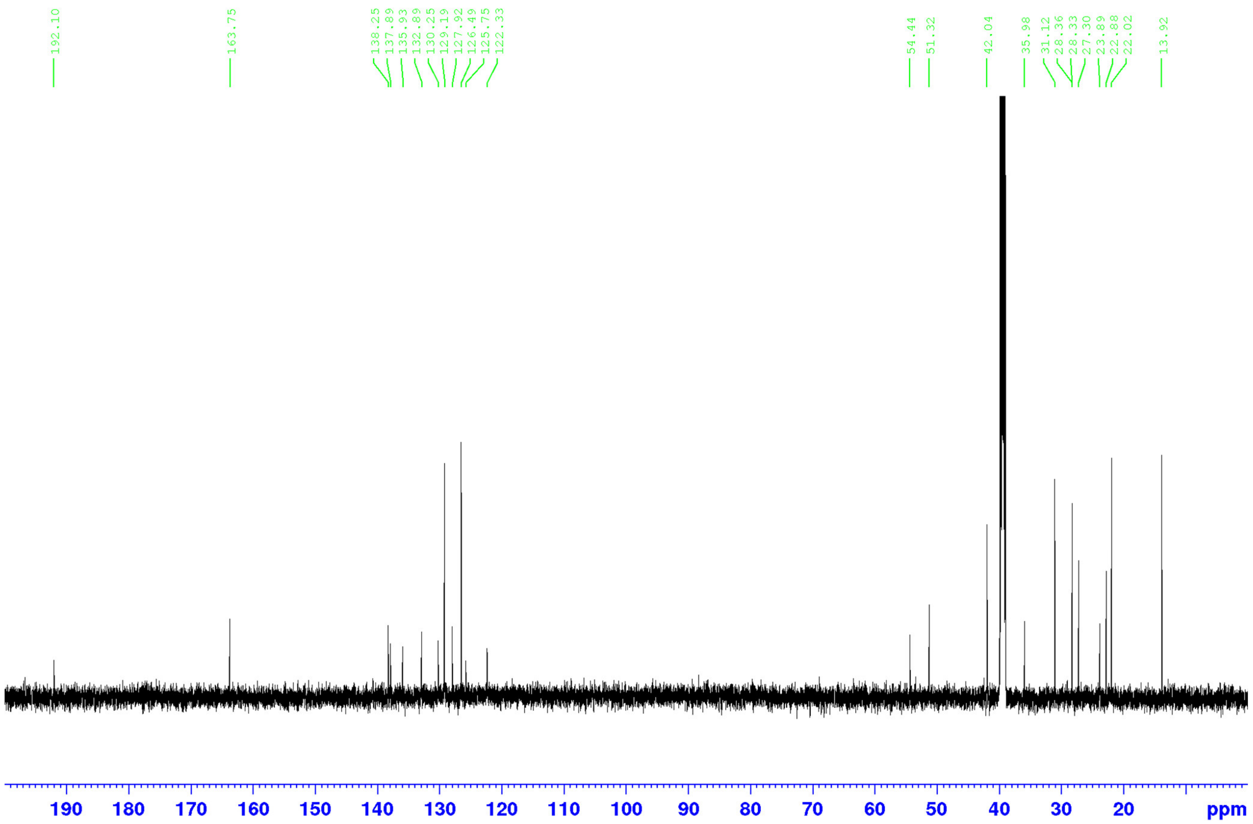

3-(2-(4'-Fluoro-4-(octylsulfonamido)-[1,1'-biphenyl]-3-yl)-2-oxoacetamido)-*N*,*N*-dimethylpropan-1-aminium chloride (**13b**)

^{1}H NMR (600 MHz, DMSO-d_6):

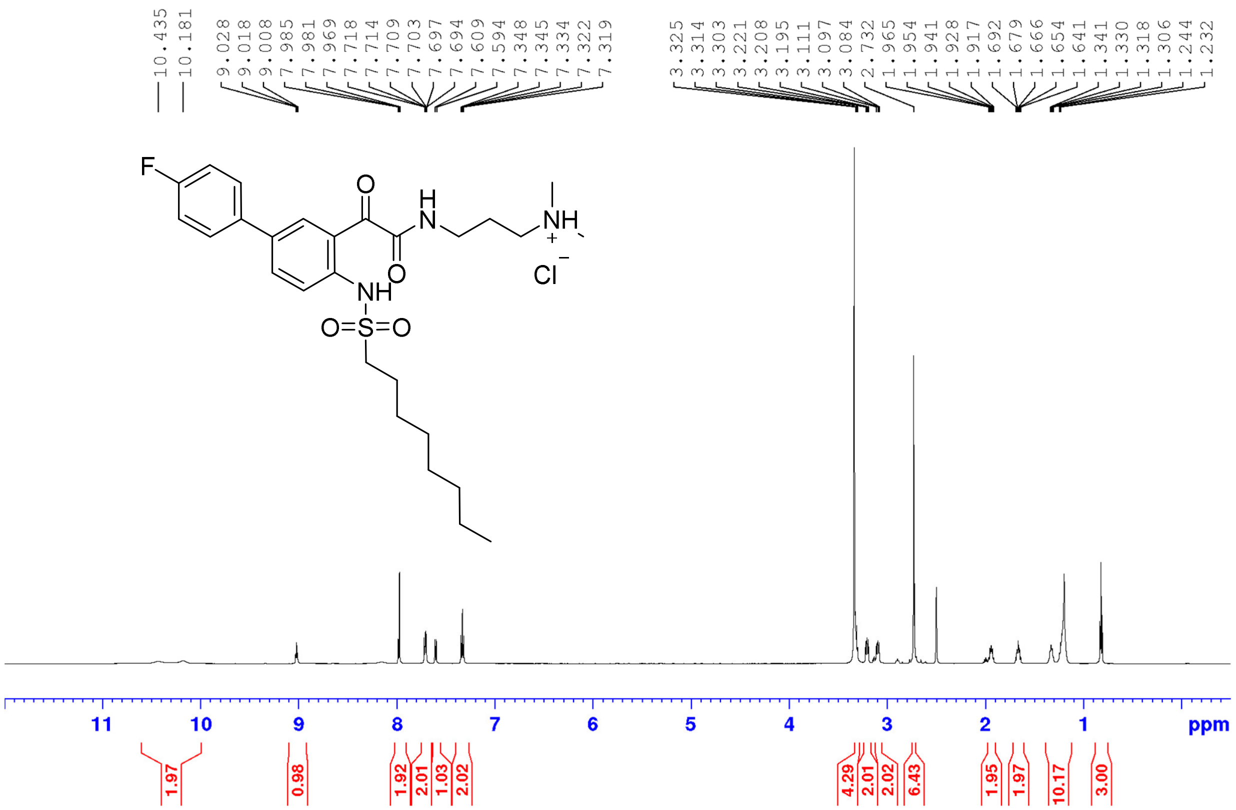

^{13}C NMR (150 MHz, DMSO-d_6):

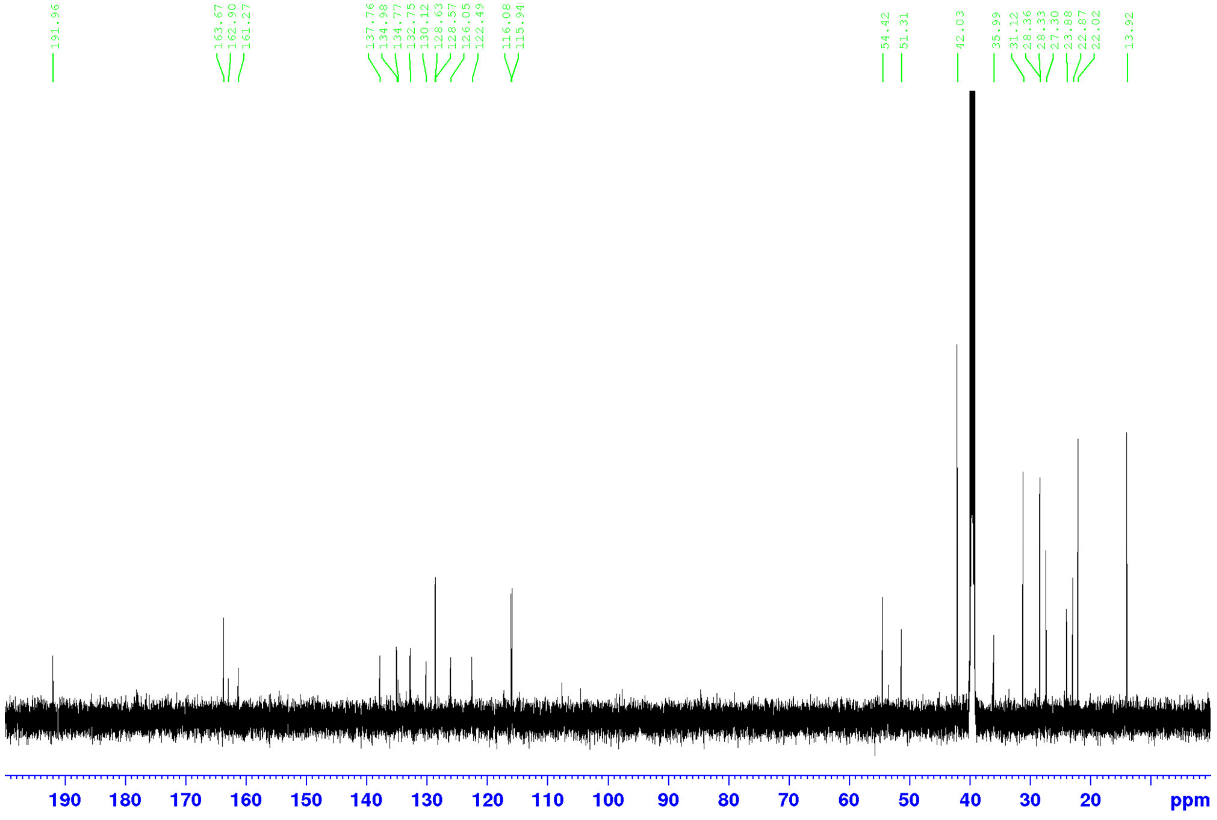

3-(2-(4'-Chloro-4-(octylsulfonamido)-[1,1'-biphenyl]-3-yl)-2-oxoacetamido)-*N*,*N*-dimethylpropan-1-aminium chloride (**13c**)

^{1}H NMR (600 MHz, DMSO-d_6):

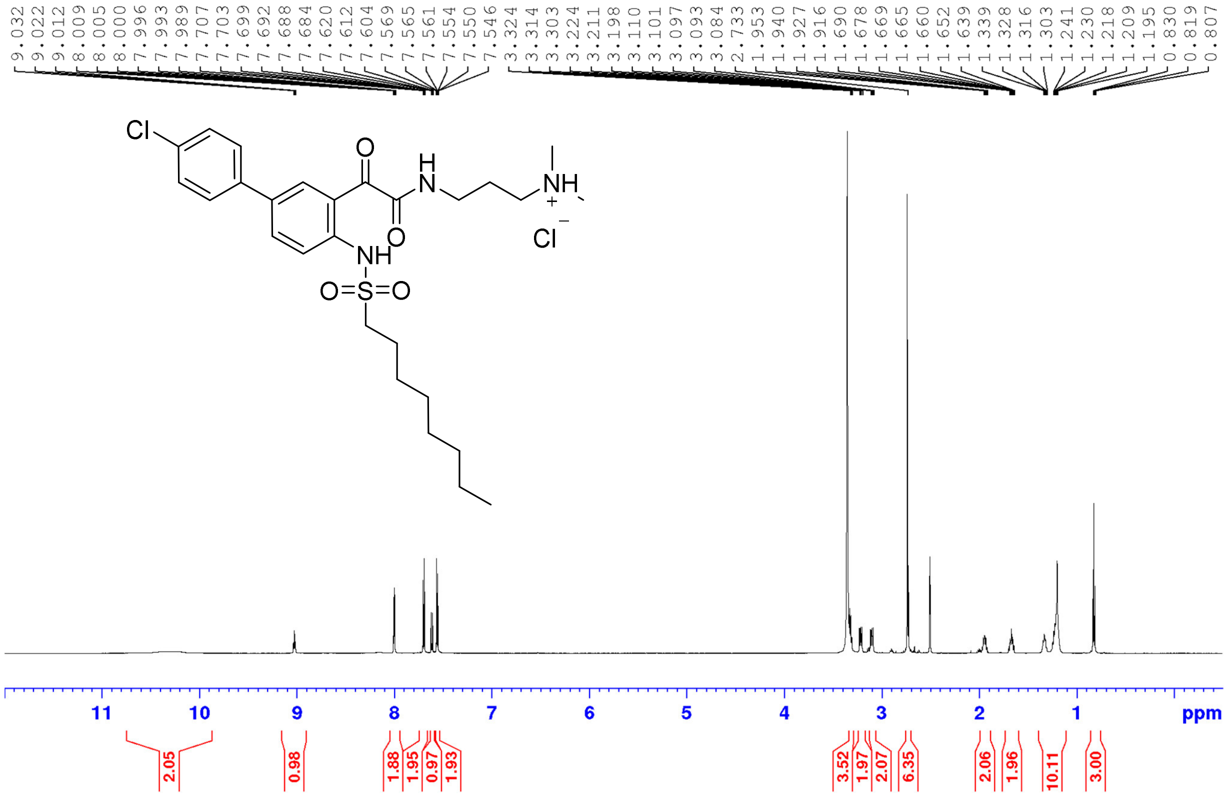

^{13}C NMR (150 MHz, DMSO-d_6):

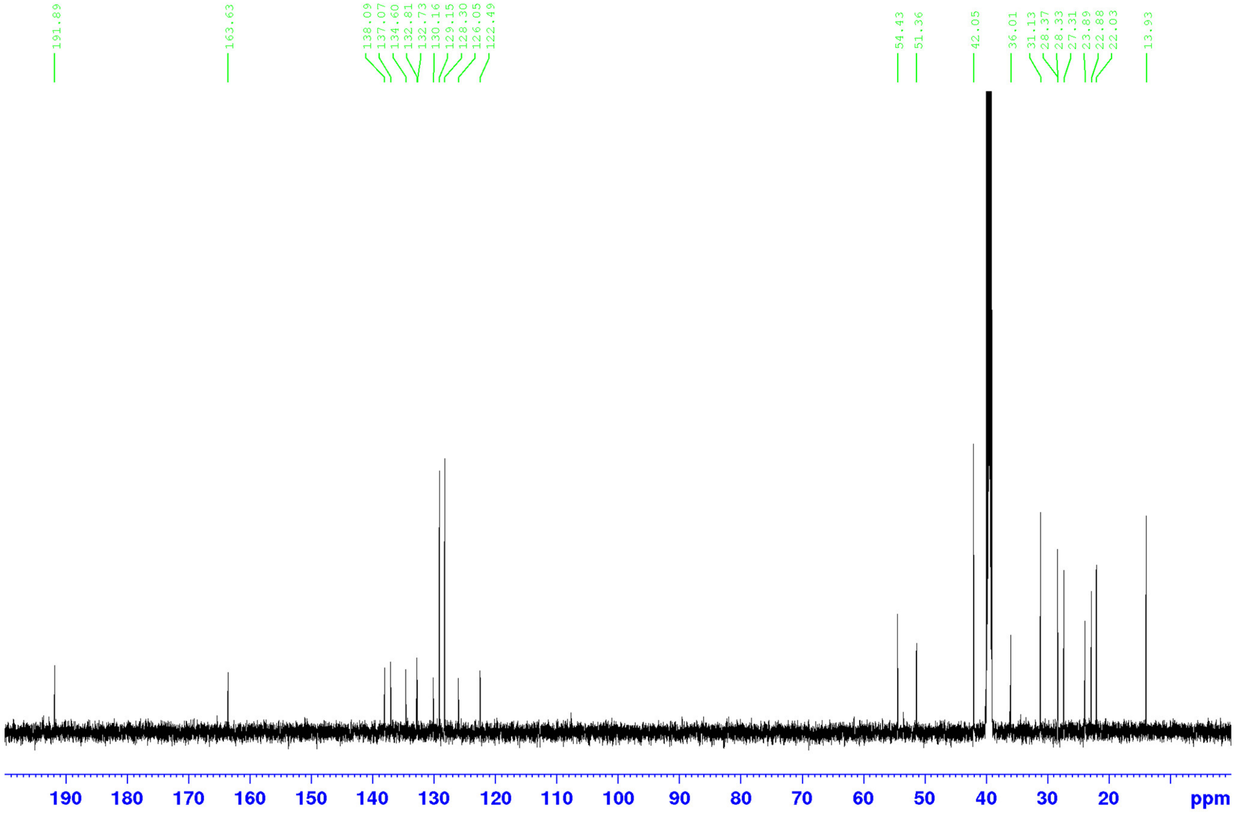

N,*N*-Dimethyl-3-(2-(5-(naphthalen-2-yl)-2-(octylsulfonamido)phenyl)-2-oxoacetamido)propan-1-aminium chloride (**13d**)

^{1}H NMR (600 MHz, DMSO-d_6):

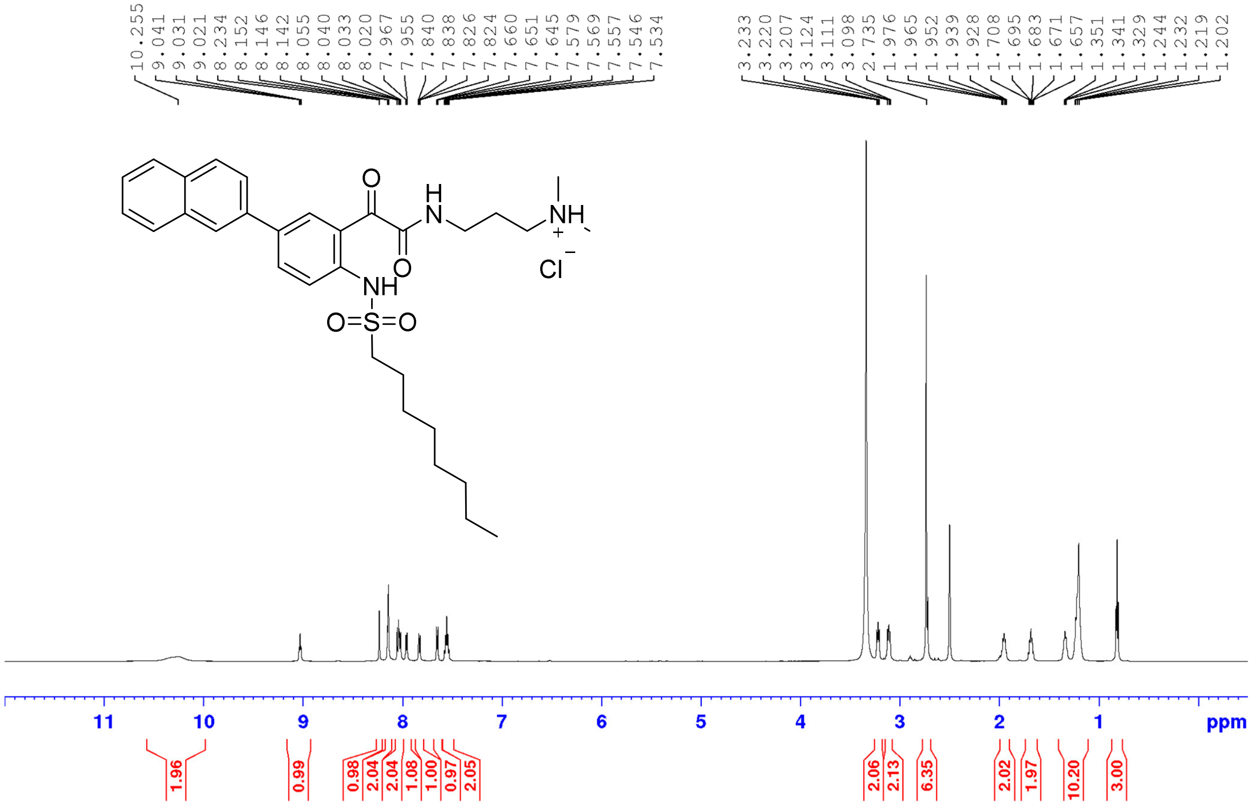

^{13}C NMR (150 MHz, DMSO-d_6):

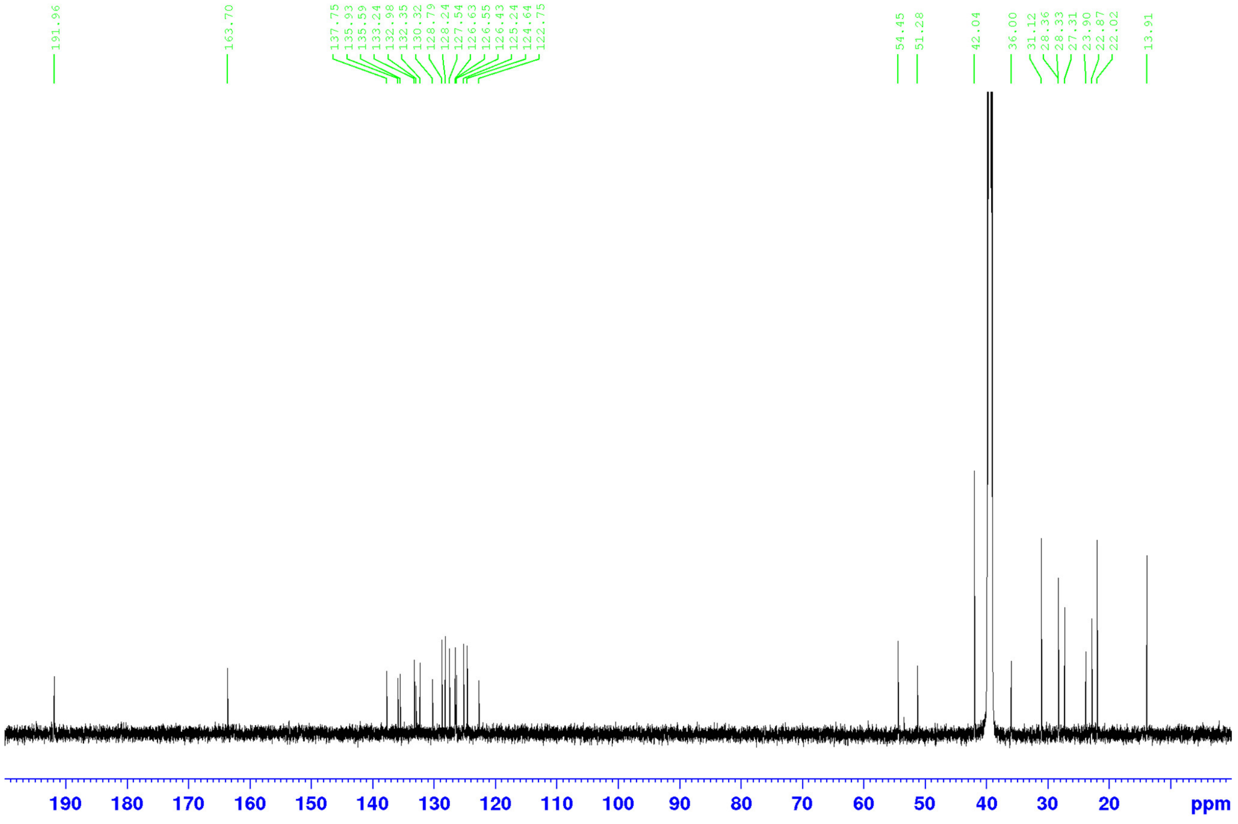

3-(2-(5-Butyl-2-(octylsulfonamido)phenyl)-2-oxoacetamido)-*N*,*N*-dimethylpropan-1-aminium chloride (**13e**)

^{1}H NMR (600 MHz, DMSO-d_6):

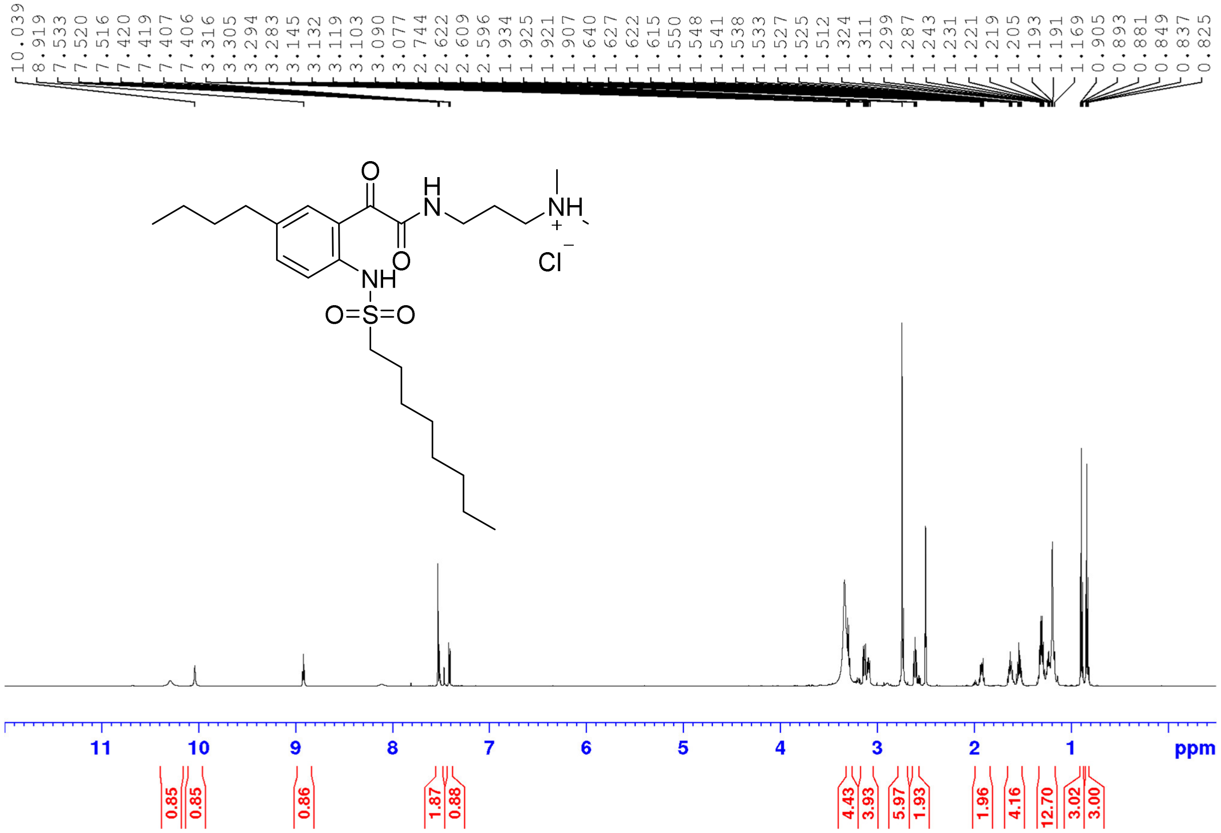

^{13}C NMR (150 MHz, DMSO-d_6):

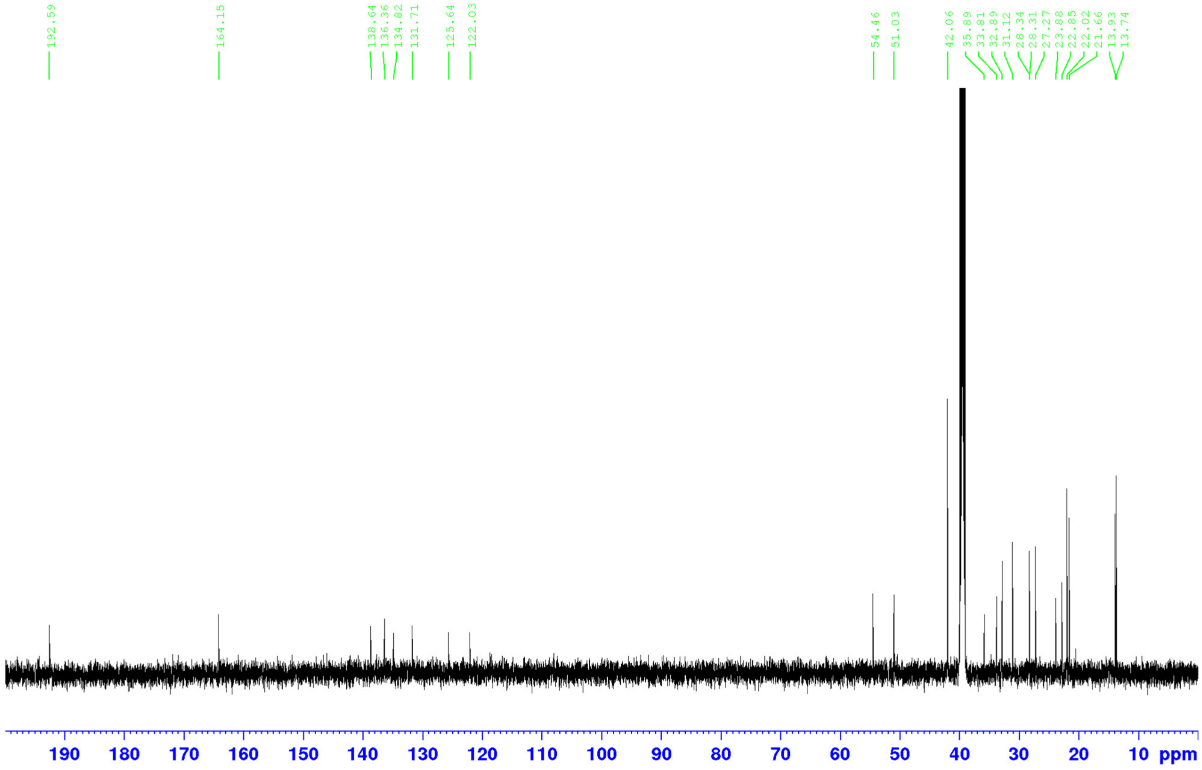

3-(2-(4-(Butylsulfonamido)-[1,1'-biphenyl]-3-yl)-2-oxoacetamido)-*N*,*N*-dimethylpropan-1-aminium chloride (**14a**)

^{1}H NMR (600 MHz, DMSO-d_6):

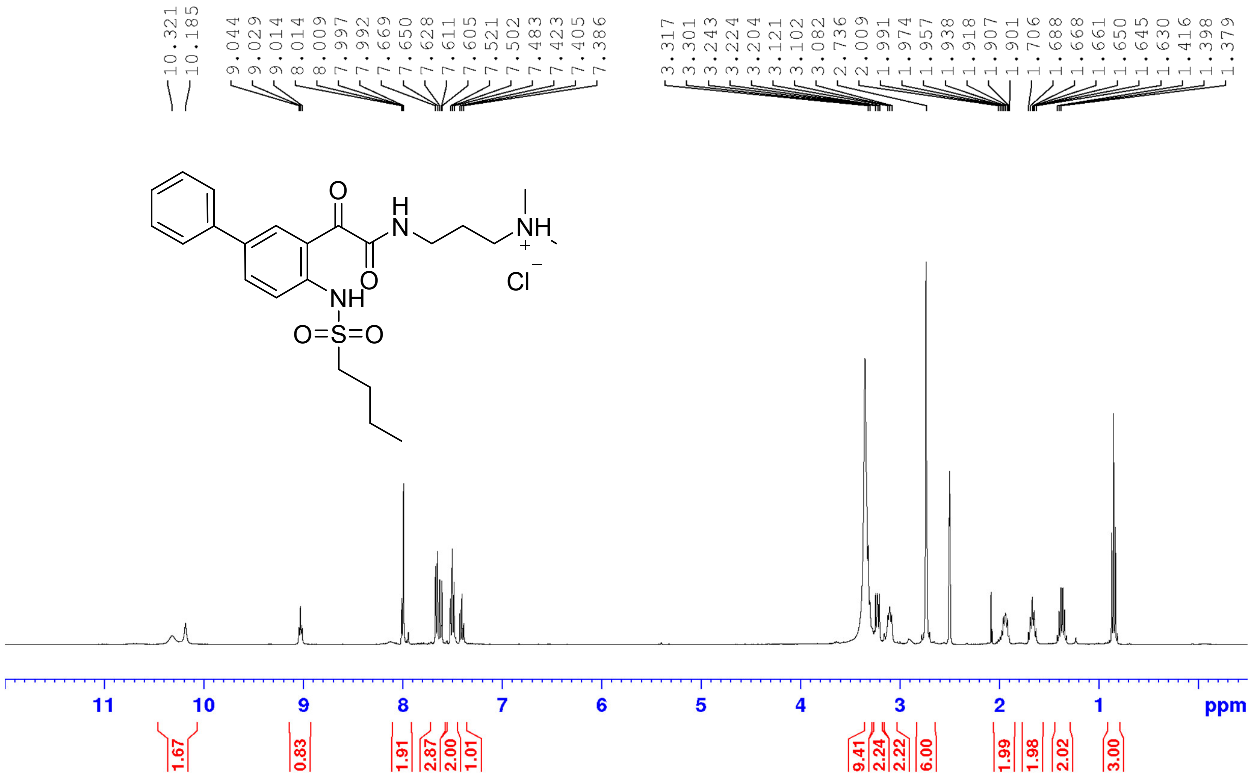

^{13}C NMR (150 MHz, DMSO-d_6):

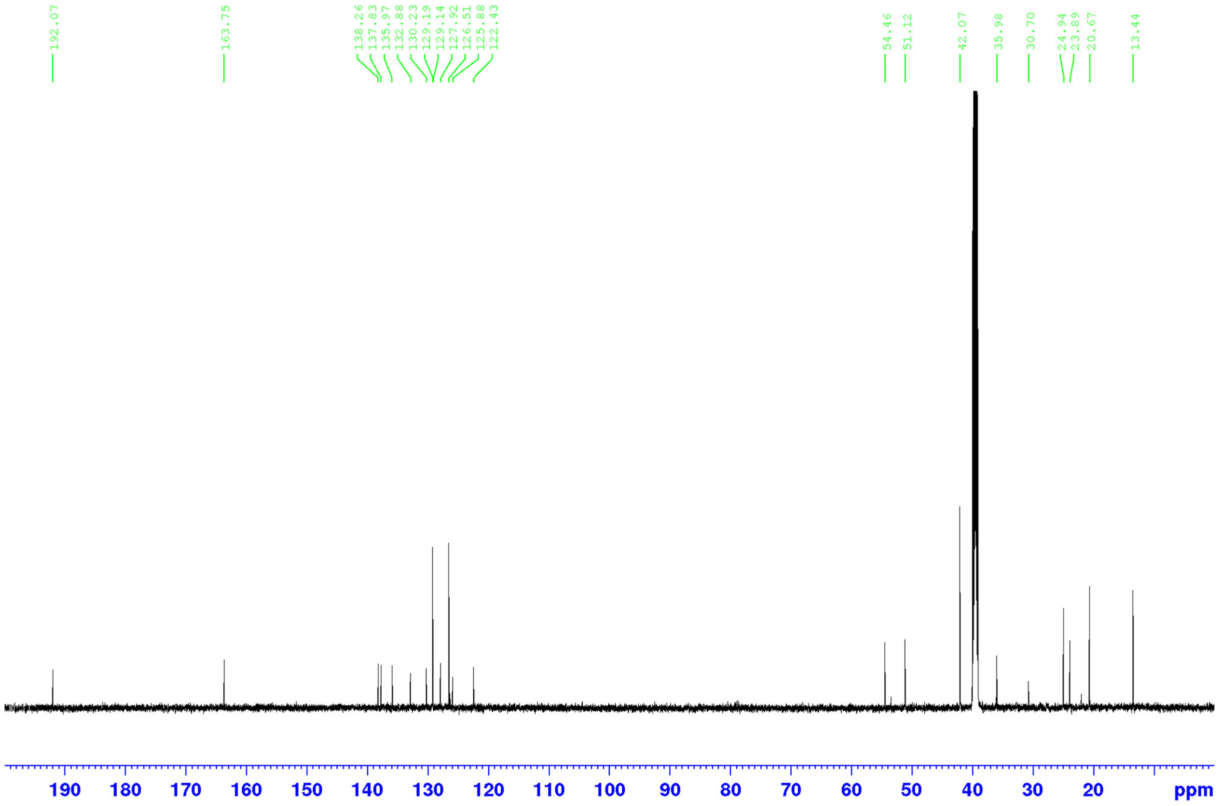

N,*N*,*N*-Trimethyl-3-(2-(4-(octylsulfonamido)-[1,1'-biphenyl]-3-yl)-2-oxoacetamido)propan-1-aminium iodide (**15a**)

^{1}H NMR (600 MHz, DMSO-d_6):

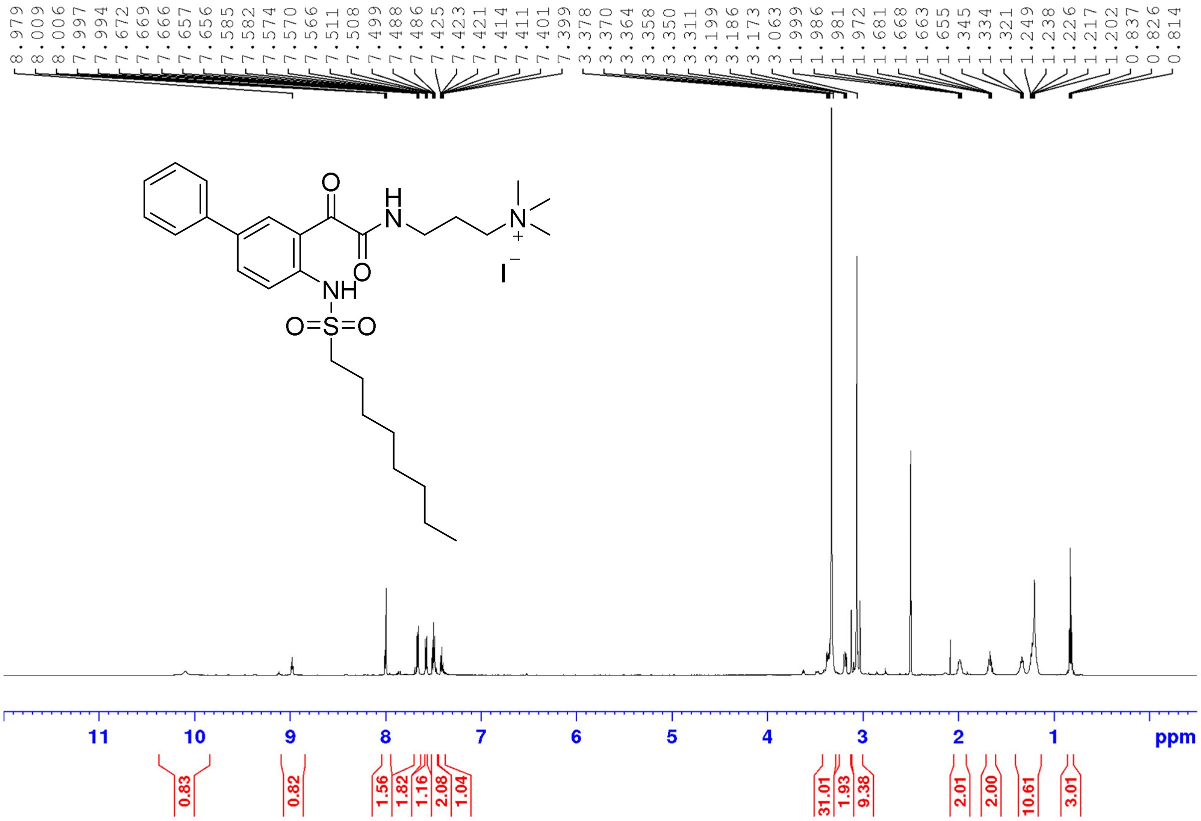

^{13}C NMR (150 MHz, DMSO-d_6):

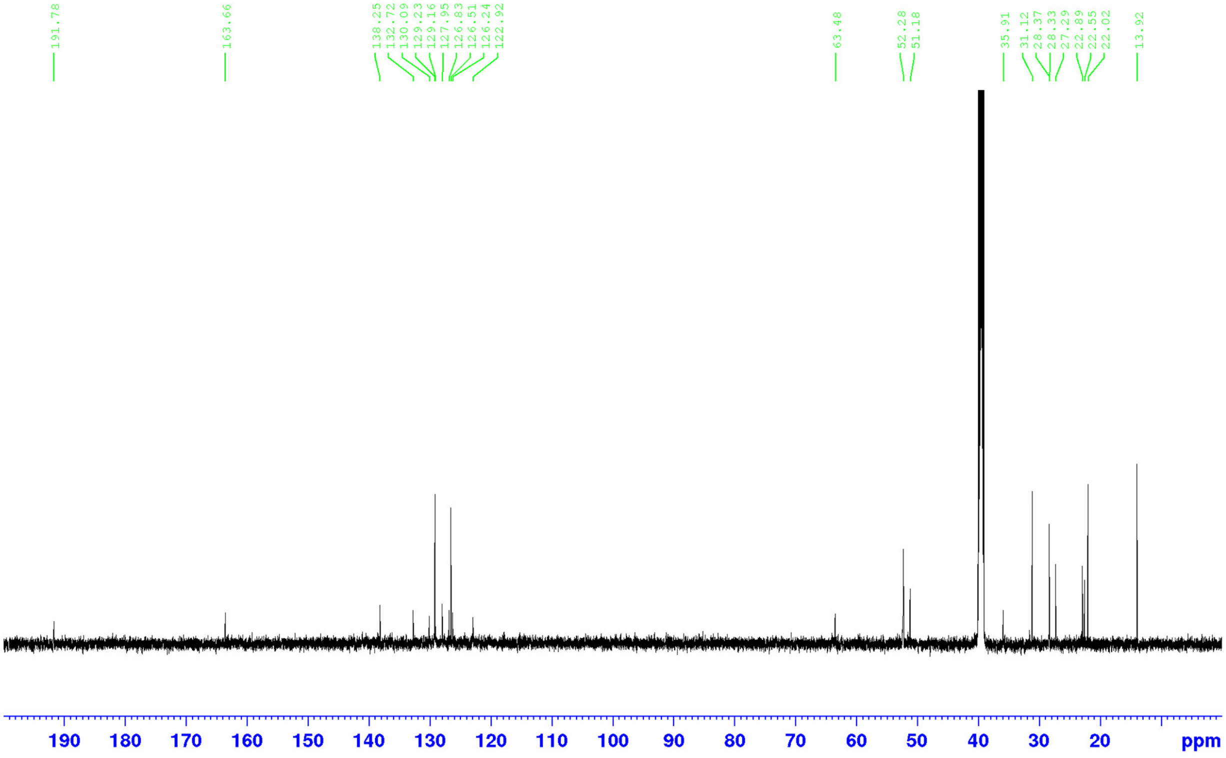

3-(2-(4'-Fluoro-4-(octylsulfonamido)-[1,1'-biphenyl]-3-yl)-2-oxoacetamido)-*N*,*N*,*N*-trimethylpropan-1-aminium iodide (**15b**)

^{1}H NMR (600 MHz, DMSO-d_6):

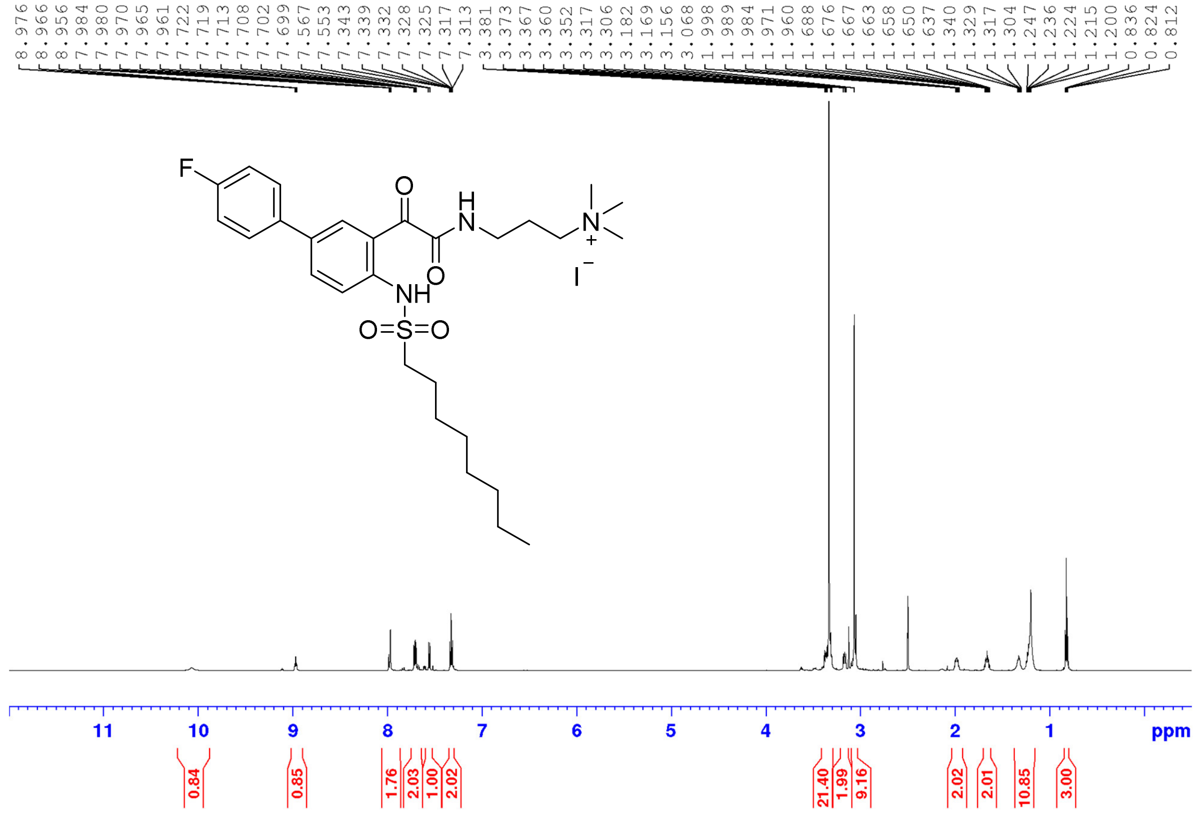

^{13}C NMR (150 MHz, DMSO-d_6):

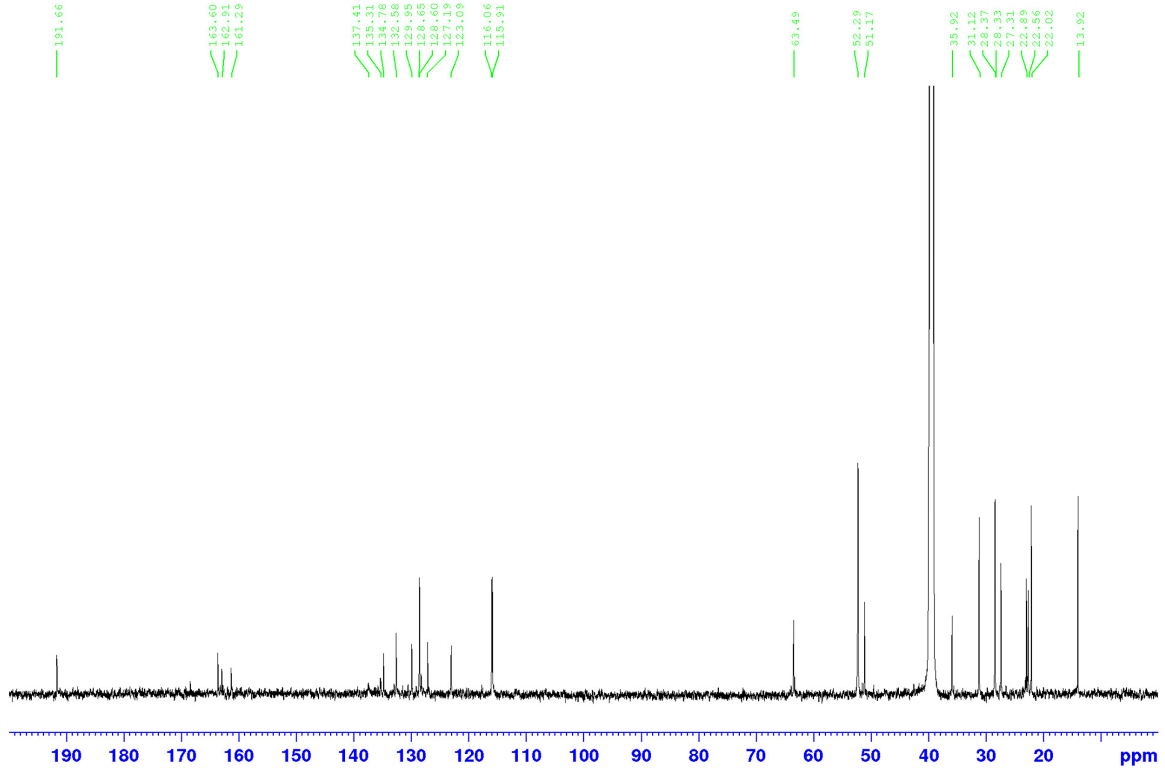

3-(2-(4'-Chloro-4-(octylsulfonamido)-[1,1'-biphenyl]-3-yl)-2-oxoacetamido)-*N*,*N*,*N*-trimethylpropan-1-aminium iodide (**15c**)

^{1}H NMR (600 MHz, DMSO-d_6):

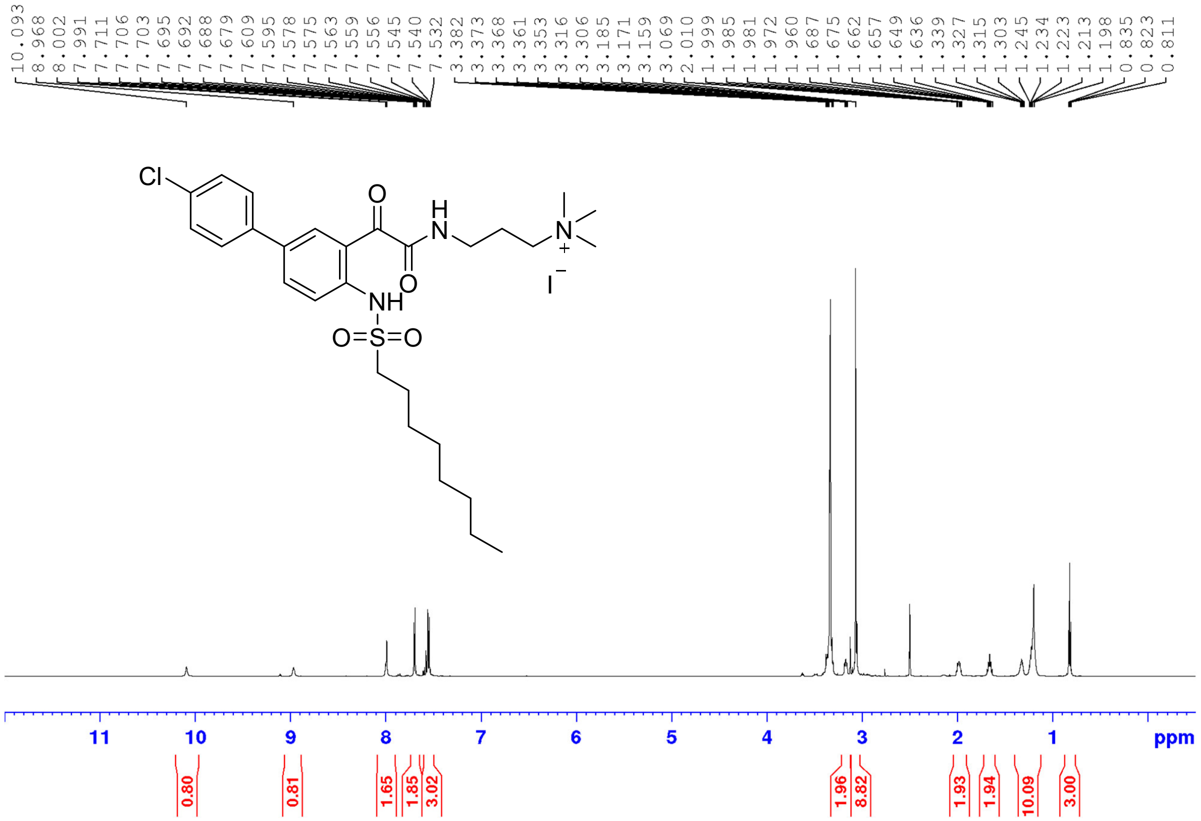

^{13}C NMR (150 MHz, DMSO-d_6):

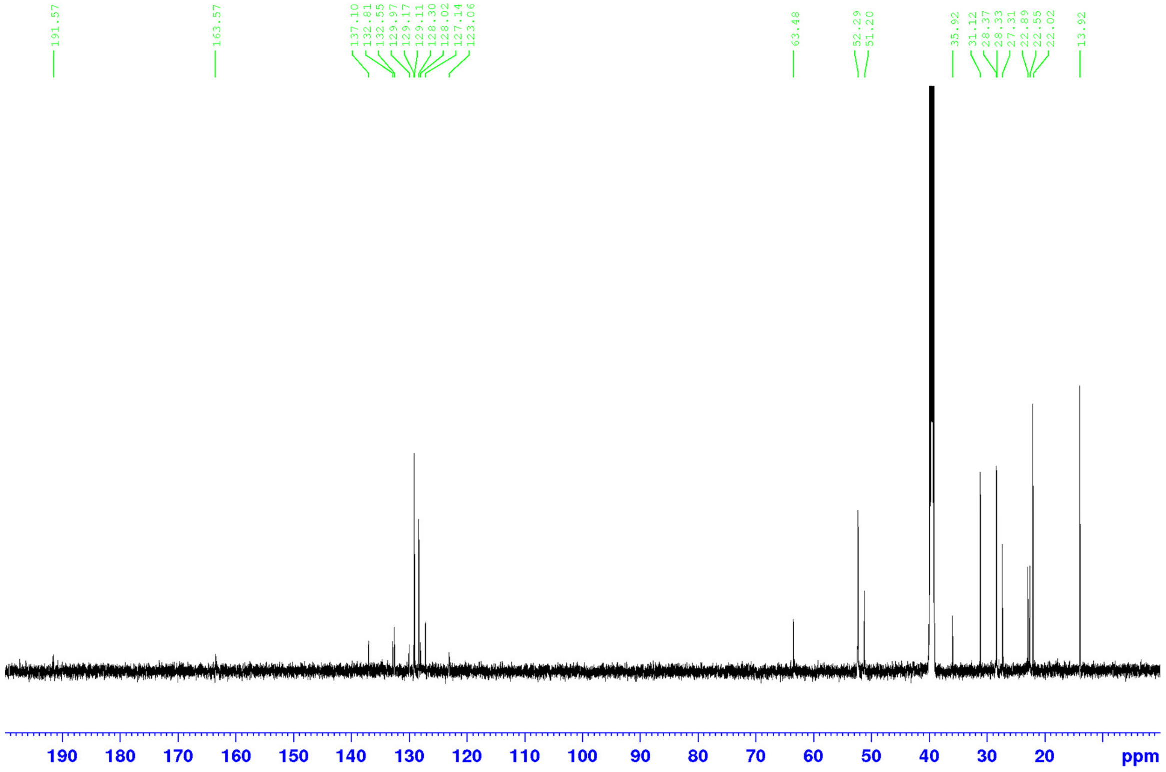

N,*N*,*N*-Trimethyl-3-(2-(5-(naphthalen-2-yl)-2-(octylsulfonamido)phenyl)-2-oxoacetamido)propan-1-aminium iodide (**15d**)

^{1}H NMR (600 MHz, DMSO-d_6):

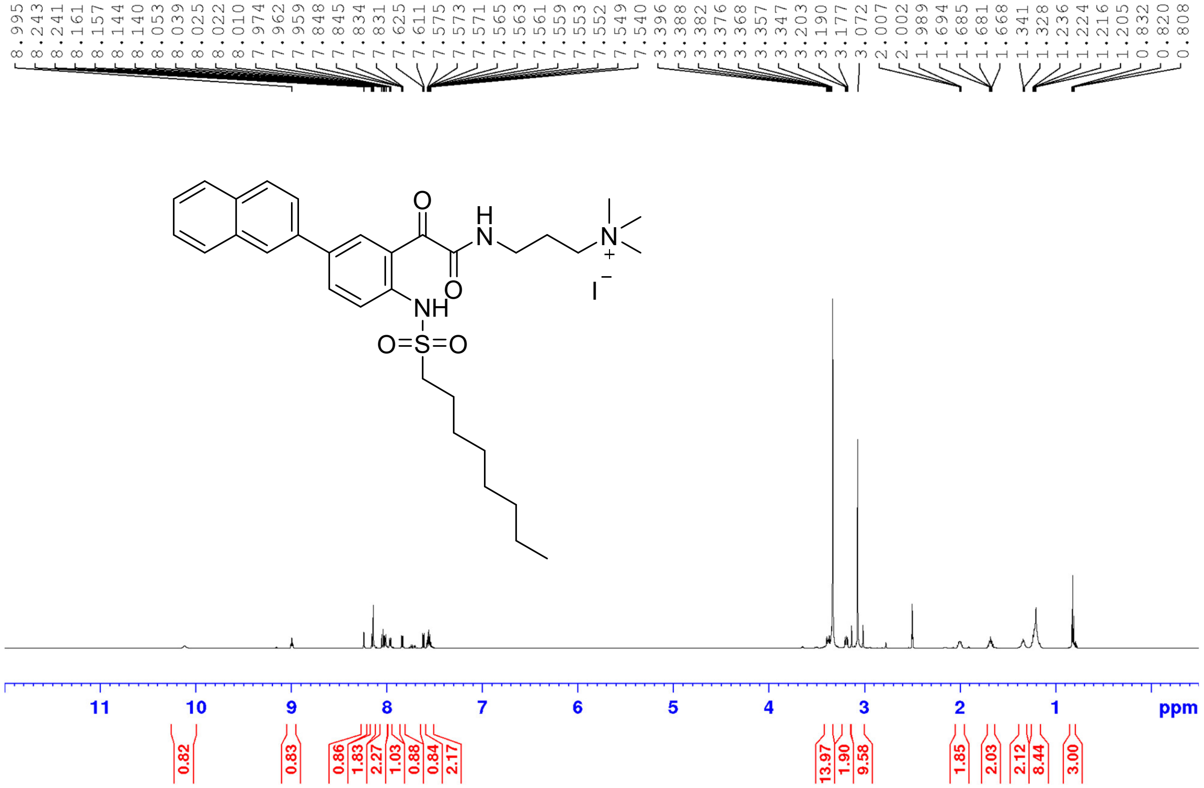

^{13}C NMR (150 MHz, DMSO-d_6):

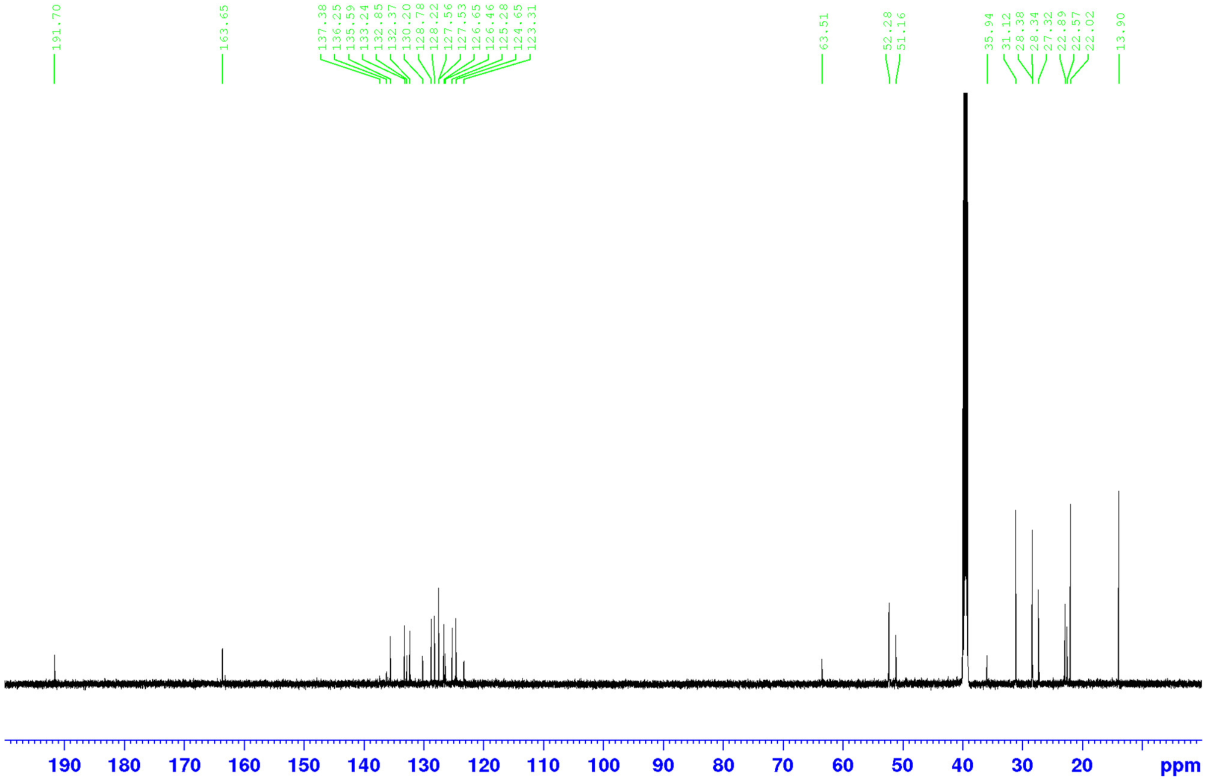

3-(2-(5-Butyl-2-(octylsulfonamido)phenyl)-2-oxoacetamido)-*N*,*N*,*N*-trimethylpropan-1-aminium iodide (**15e**)

^{1}H NMR (600 MHz, DMSO-d_6):

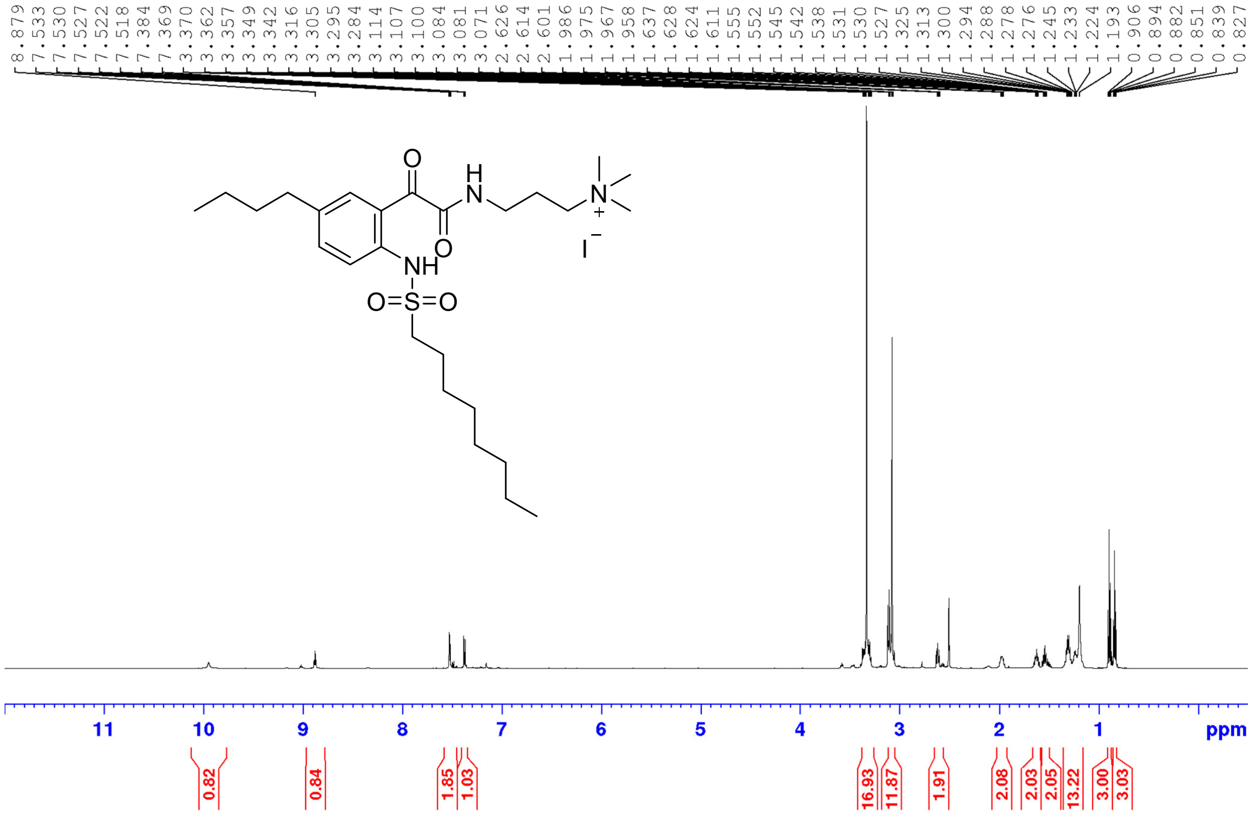

^{13}C NMR (150 MHz, DMSO-d_6):

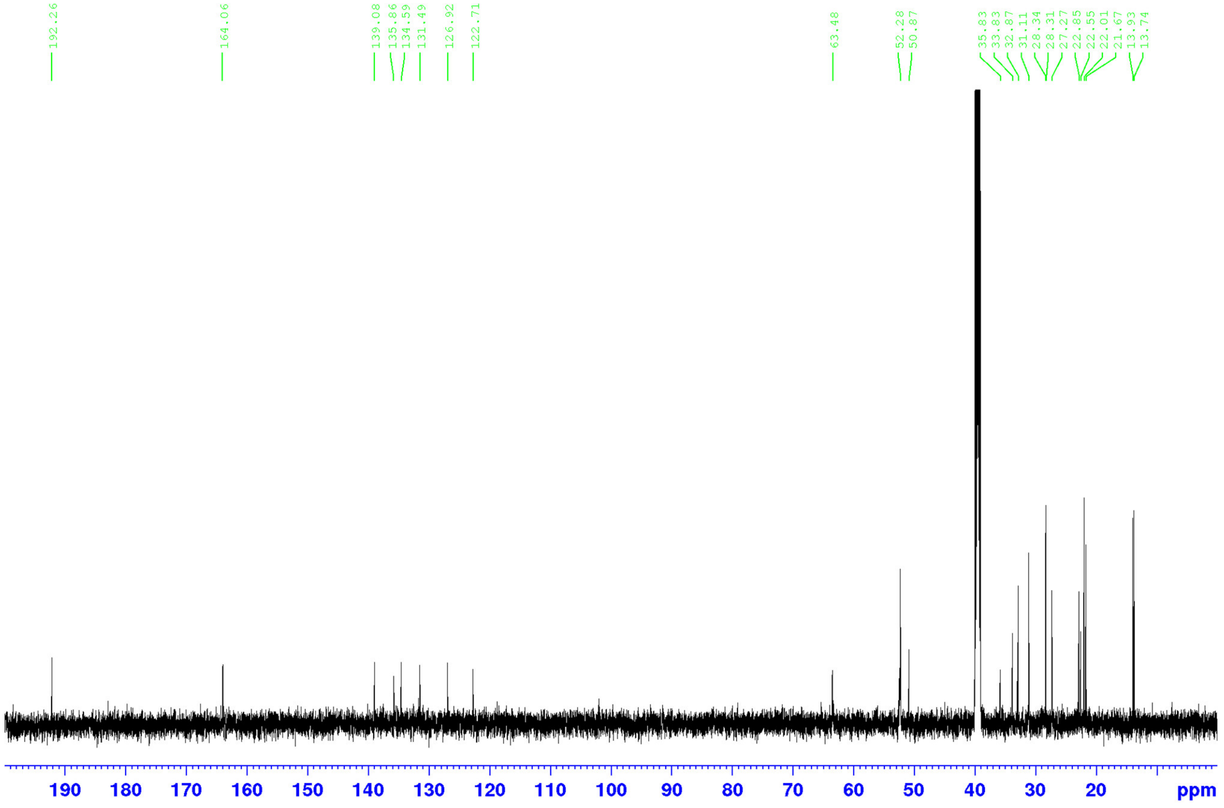

3-(2-(4-(Butylsulfonamido)-[1,1'-biphenyl]-3-yl)-2-oxoacetamido)-*N*,*N*,*N*-trimethylpropan-1-aminium iodide (**16a**)

^{1}H NMR (600 MHz, DMSO-d_6):

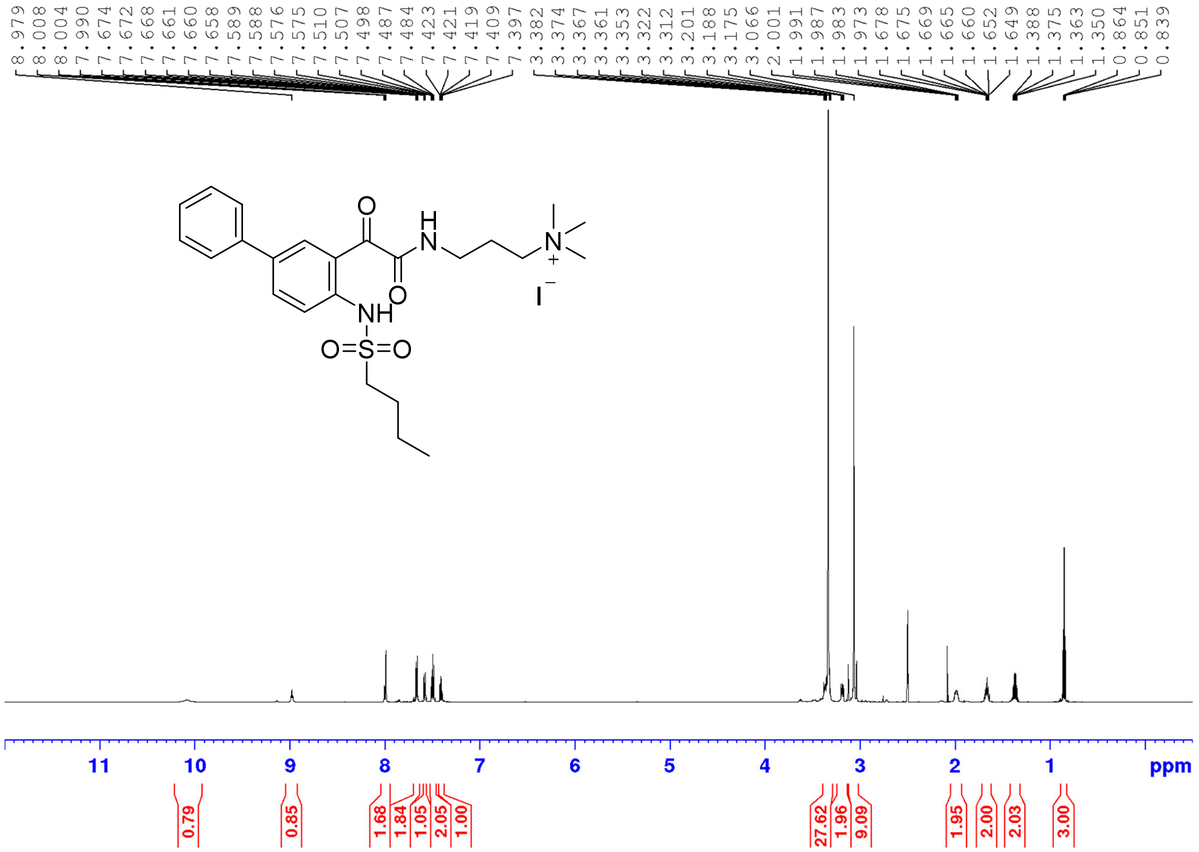

^{13}C NMR (150 MHz, DMSO-d_6):

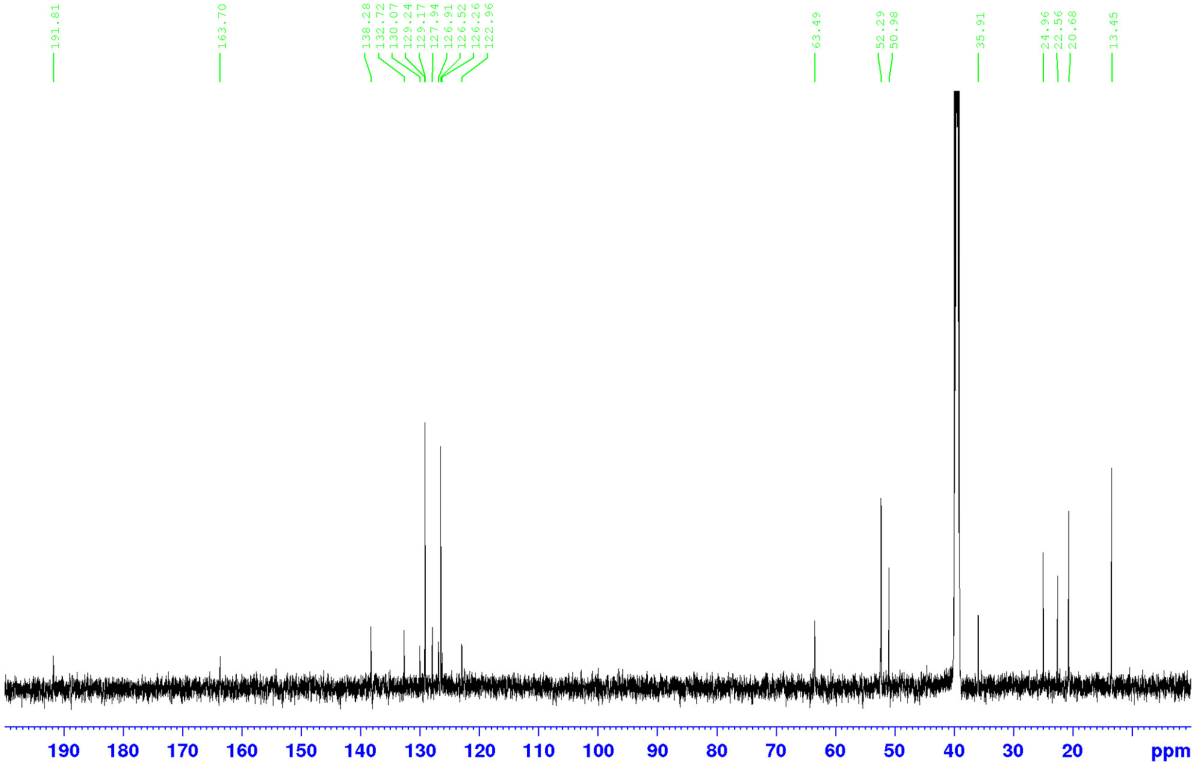

tert-Butyl (3-(2-(4-(octylsulfonamido)-[1,1'-biphenyl]-3-yl)-2-oxoacetamido)propyl)carbamate (**18a**)

^{1}H NMR (600 MHz, $CDCl_3$):

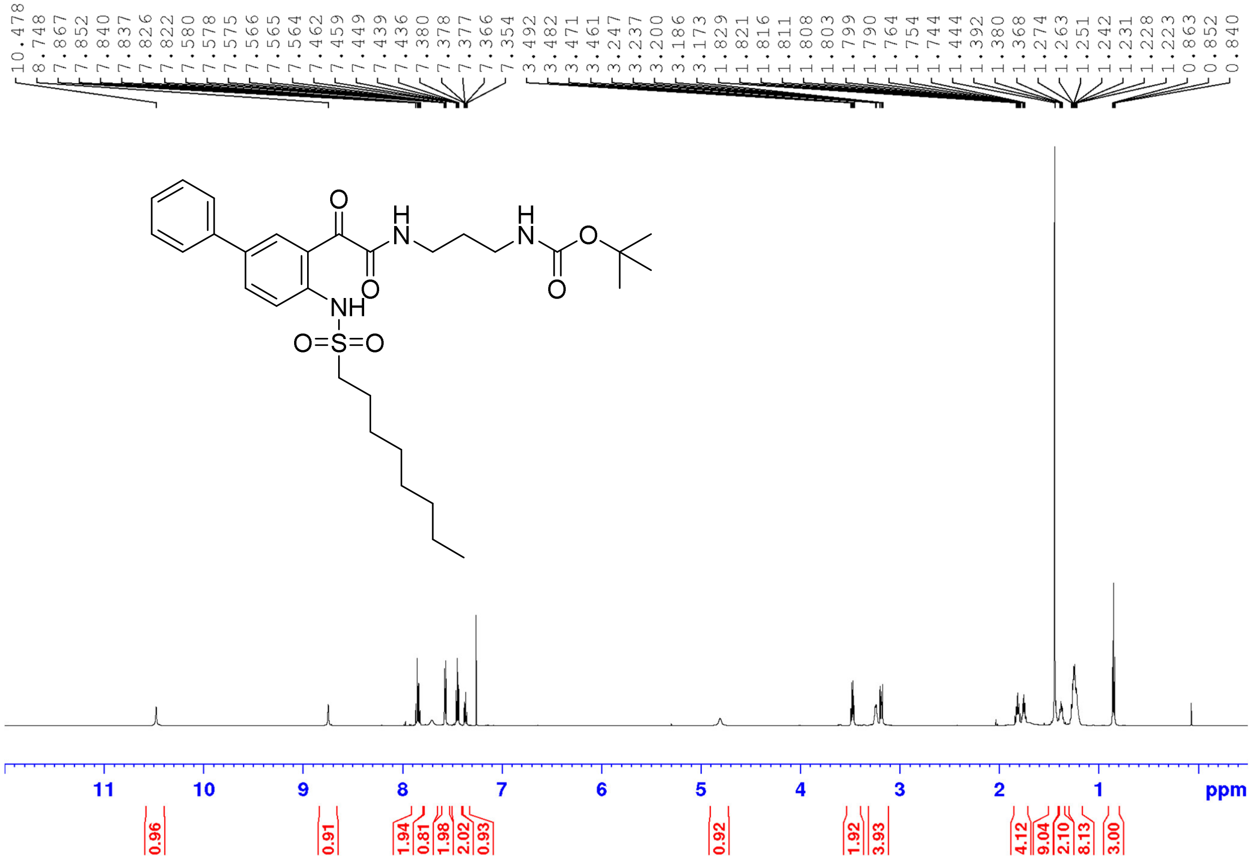

^{13}C NMR (150 MHz, $CDCl_3$):

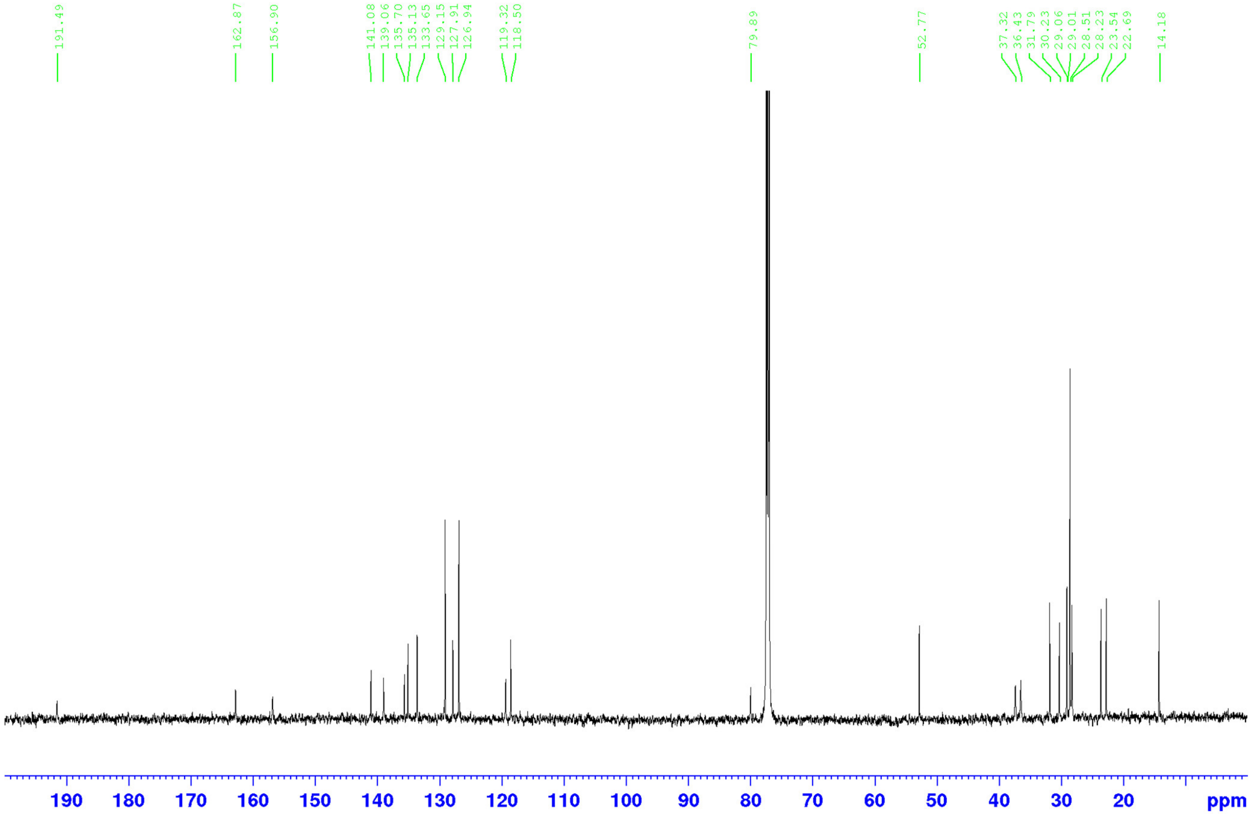

tert-Butyl (3-(2-(4'-fluoro-4-(octylsulfonamido)-[1,1'-biphenyl]-3-yl)-2-oxoacetamido)propyl) carbamate (**18b**)

^{1}H NMR (400 MHz, $CDCl_3$):

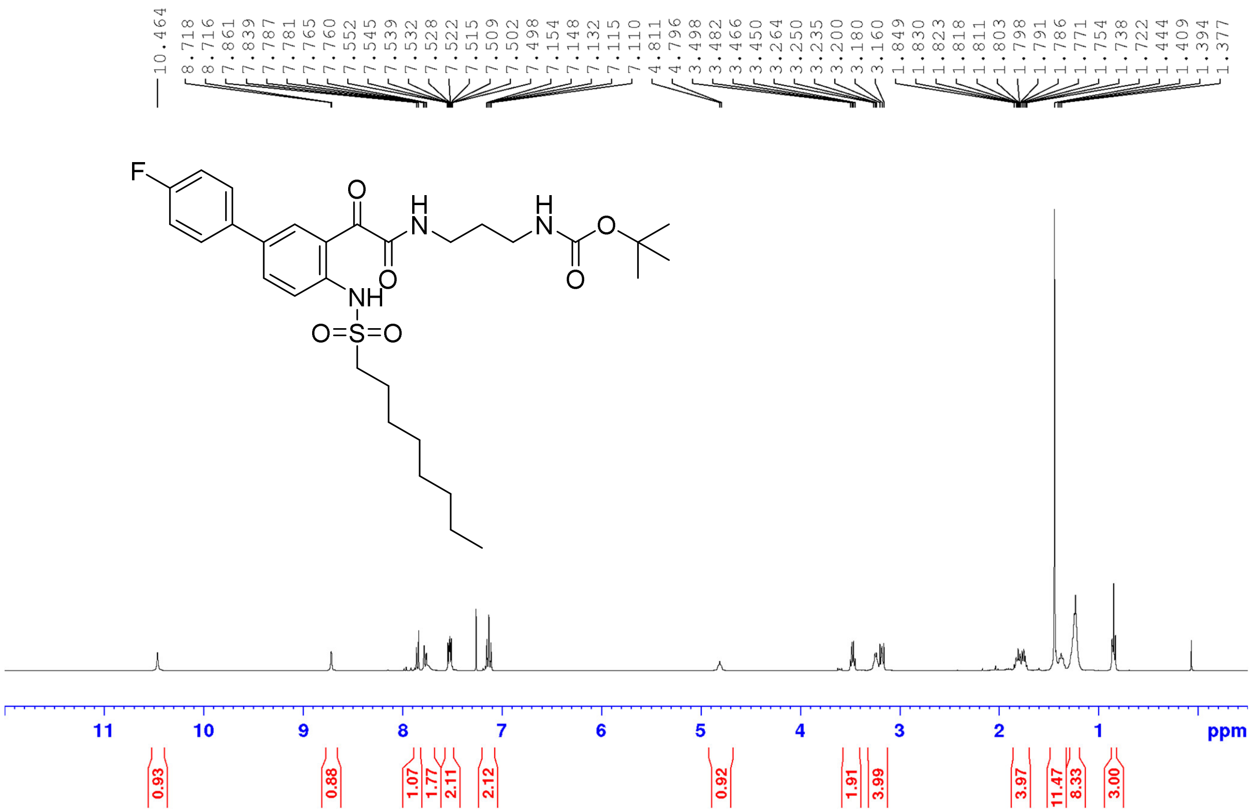

^{13}C NMR (100 MHz, $CDCl_3$):

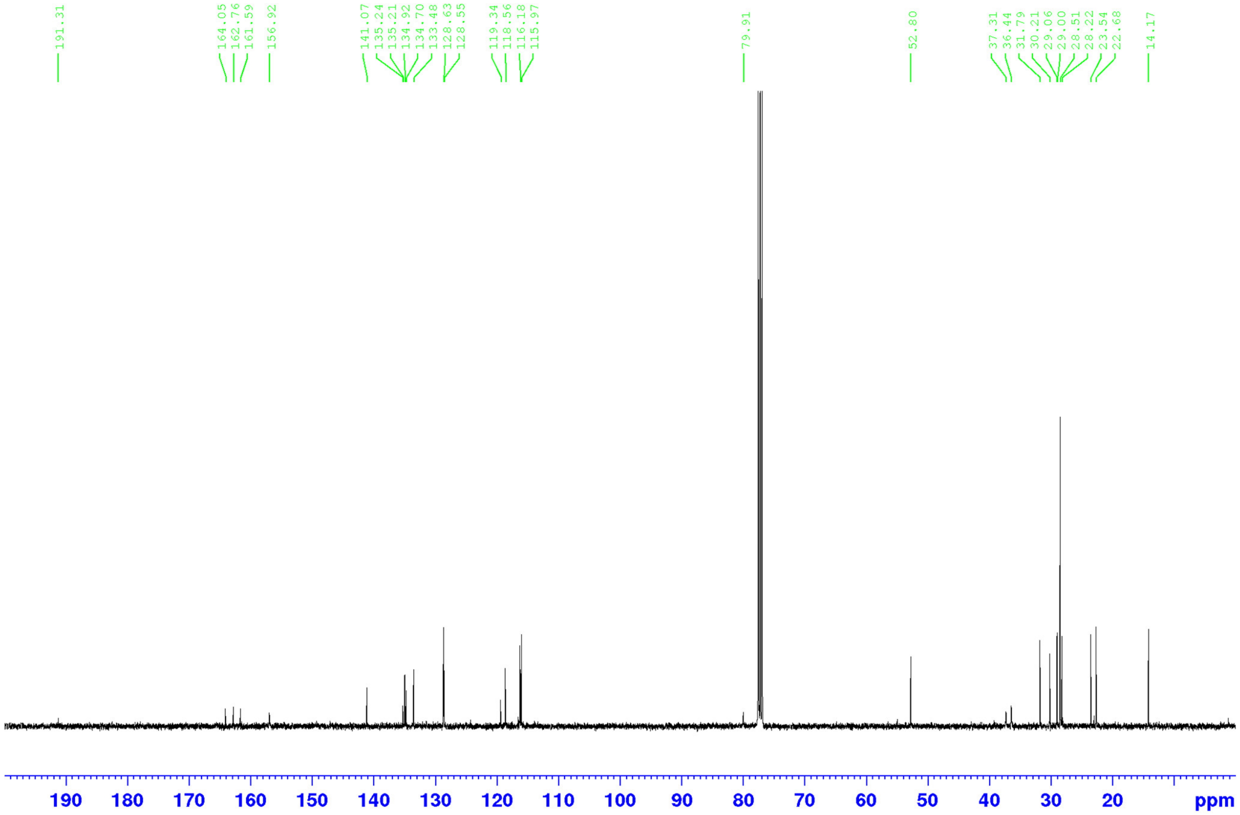

tert-Butyl (3-(2-(4'-Chloro-4-(octylsulfonamido)-[1,1'-biphenyl]-3-yl)-2-oxoacetamido)propyl) carbamate (**18c**)

^{1}H NMR (600 MHz, $CDCl_3$):

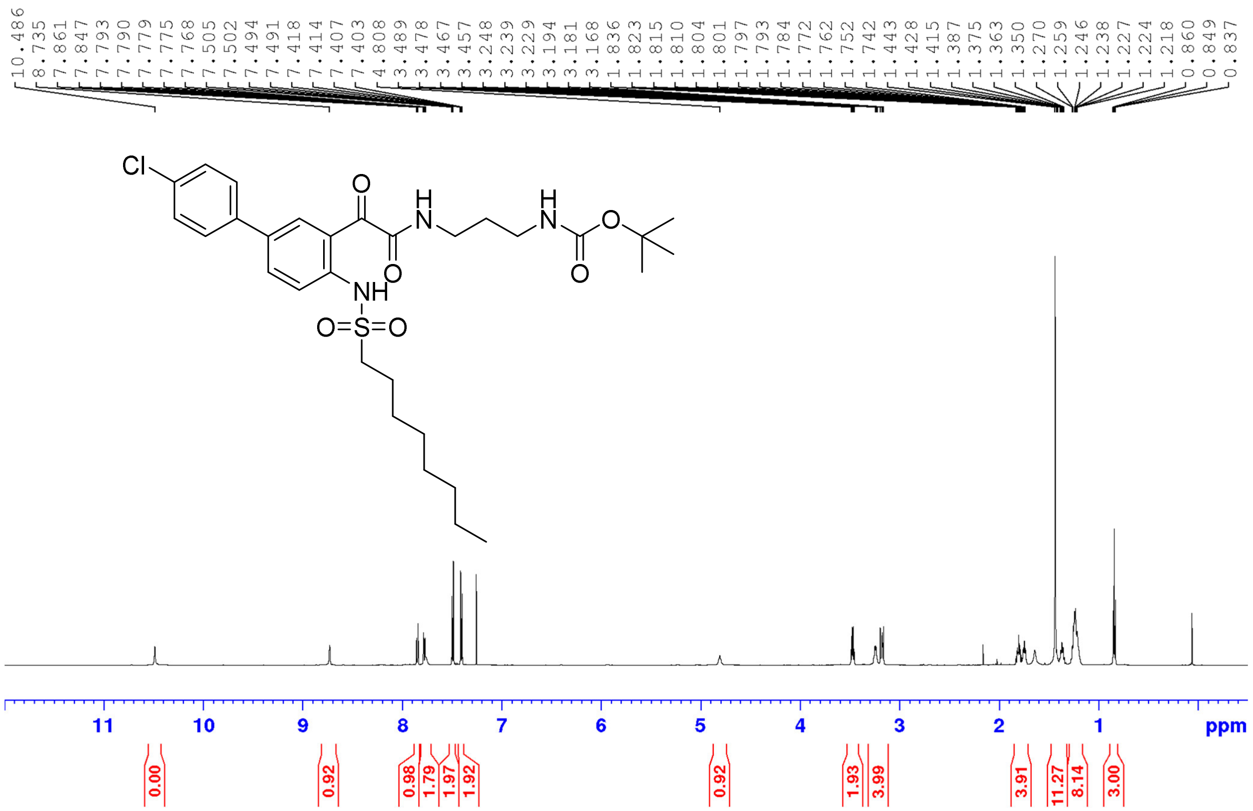

^{13}C NMR (150 MHz, $CDCl_3$):

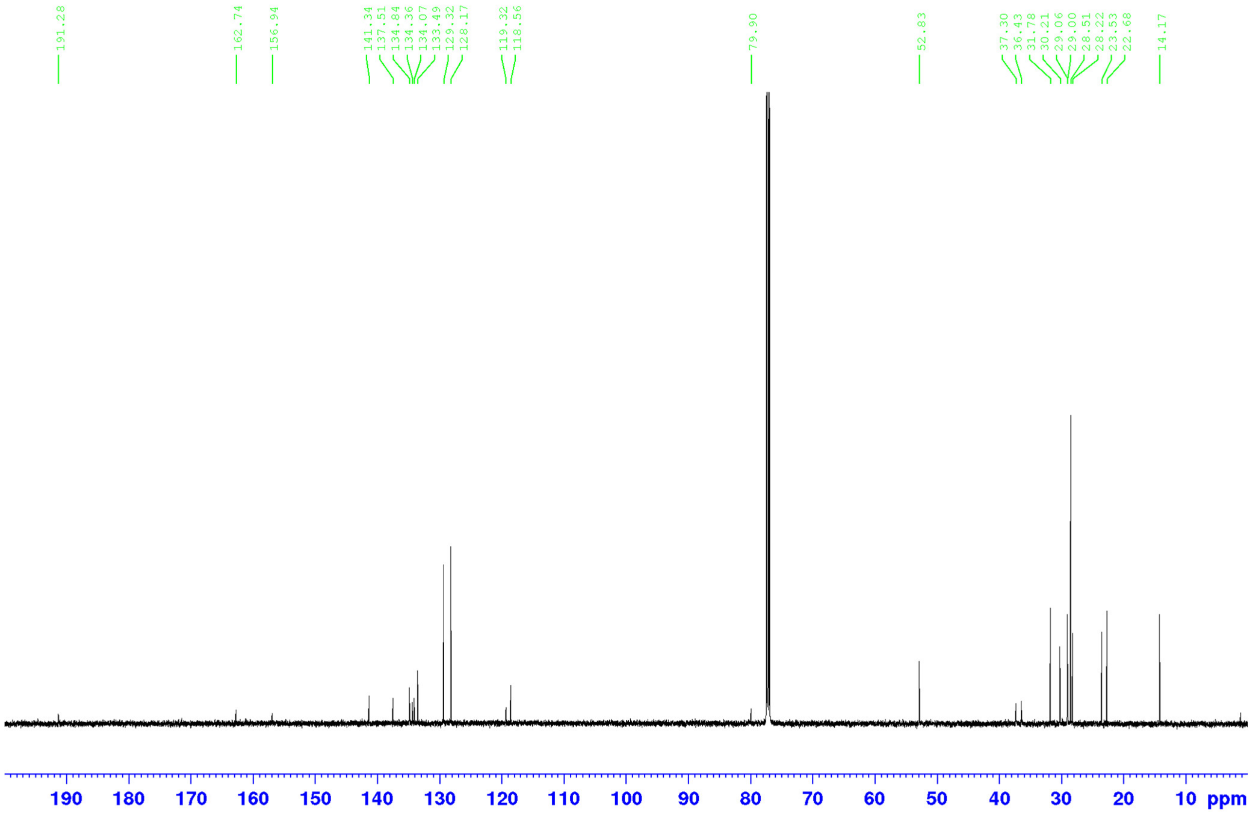

tert-Butyl (3-(2-(5-(naphthalen-2-yl)-2-(octylsulfonamido)phenyl)-2-oxoacetamido)propyl) carbamate (**18d**)

^{1}H NMR (600 MHz, $CDCl_3$):

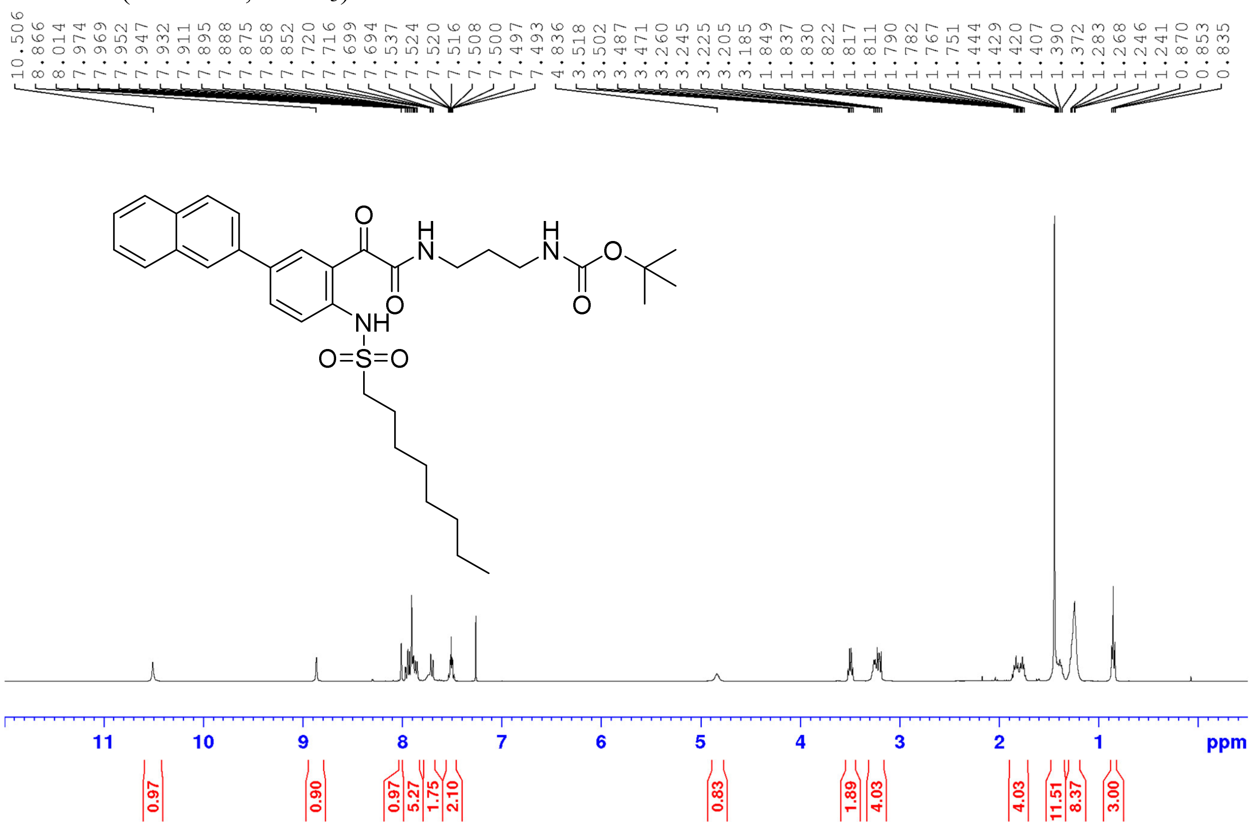

^{13}C NMR (150 MHz, $CDCl_3$):

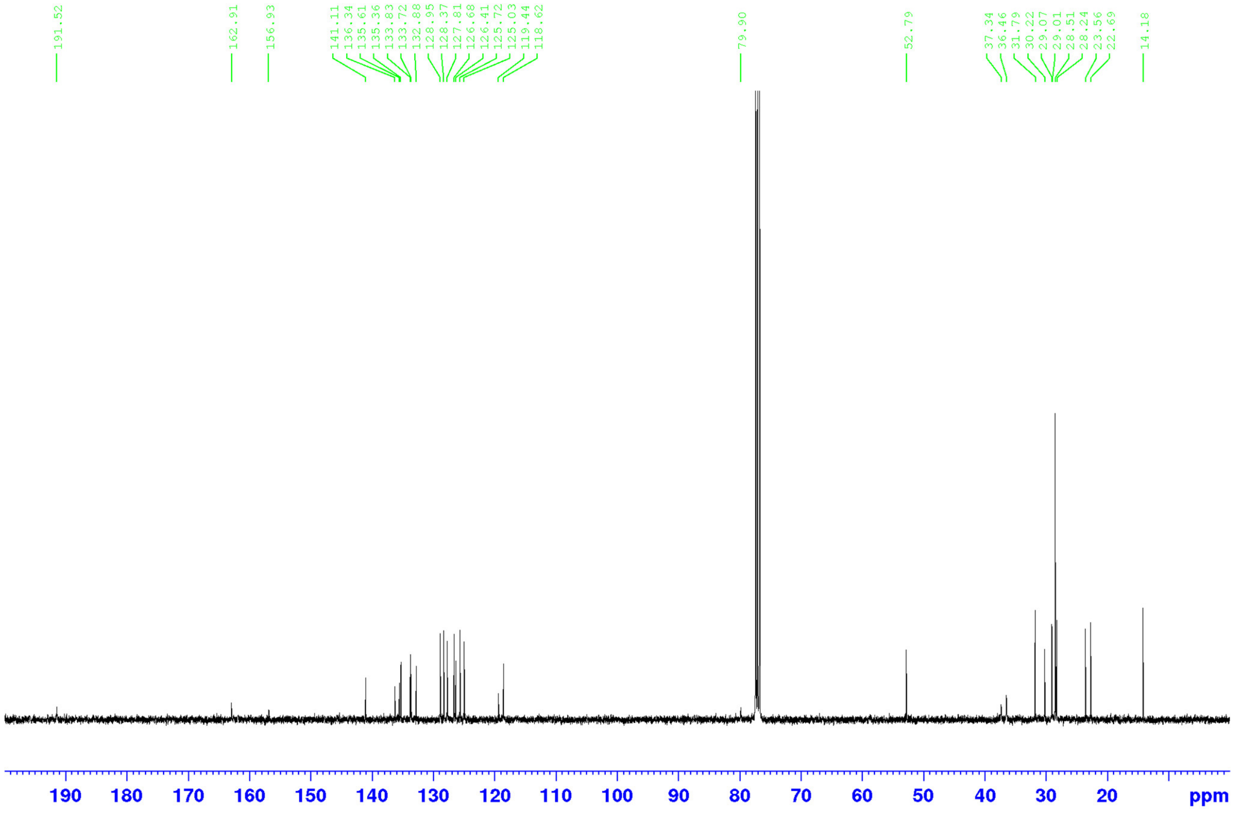

N-(3-Aminopropyl)-2-(4-(octylsulfonamido)-[1,1'-biphenyl]-3-yl)-2-oxoacetamide hydrochloride (**19a**)

^{1}H NMR (400 MHz, DMSO-d_6):

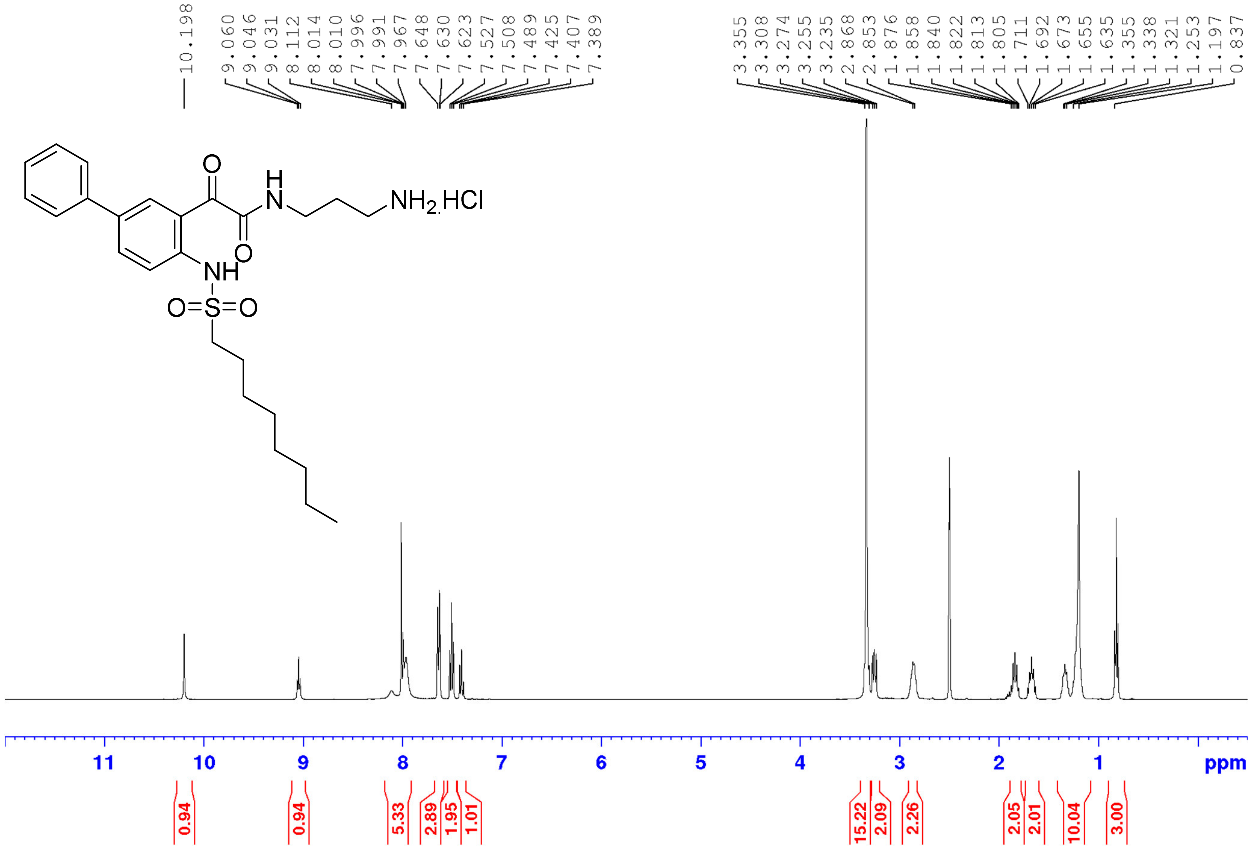

^{13}C NMR (100 MHz, DMSO-d_6):

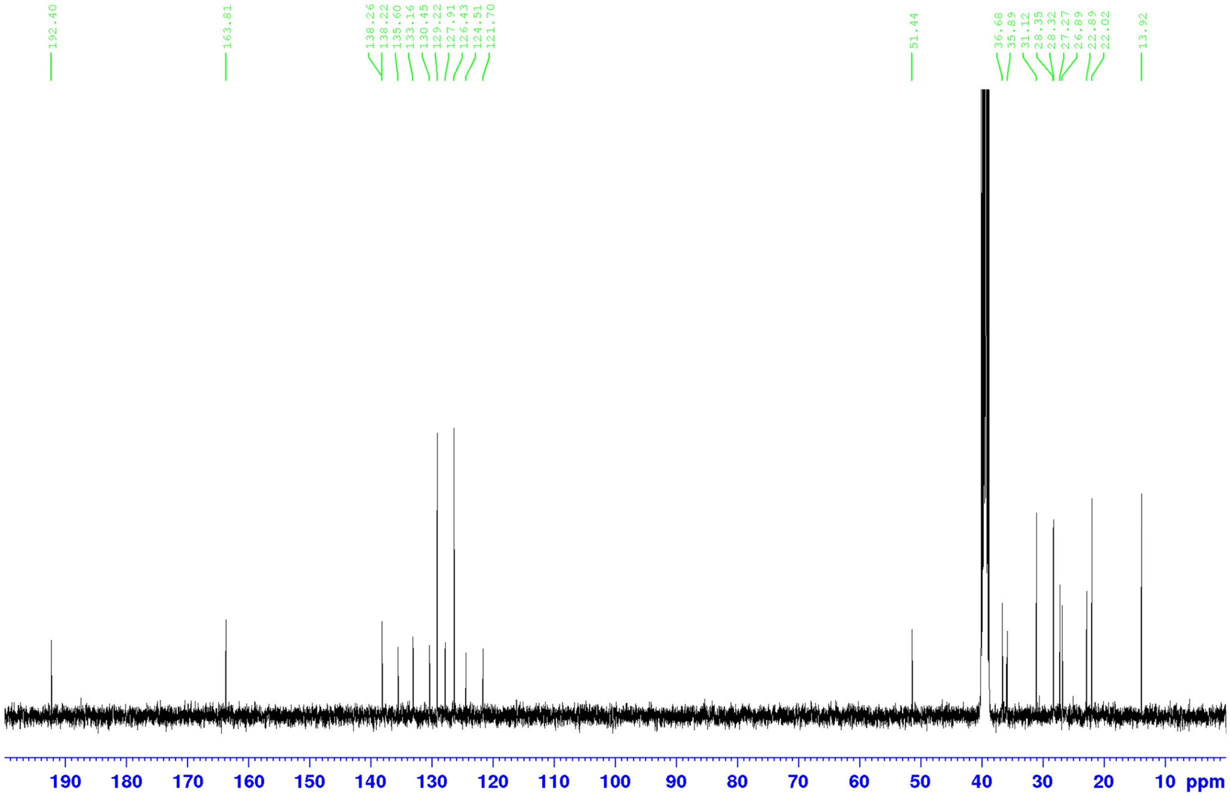

N-(3-Aminopropyl)-2-(4'-fluoro-4-(octylsulfonamido)-[1,1'-biphenyl]-3-yl)-2-oxoacetamide hydrochloride (**19b**)

^{1}H NMR (400 MHz, DMSO-d_6):

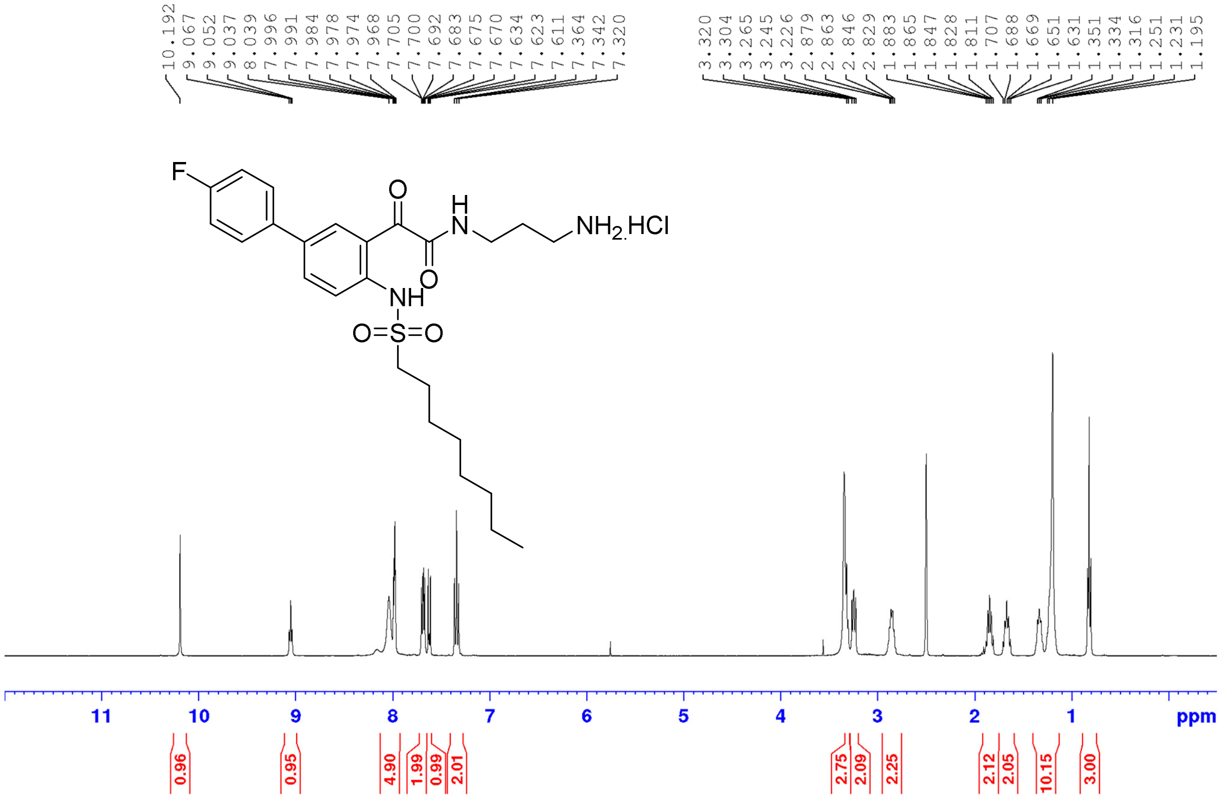

^{13}C NMR (100 MHz, DMSO-d_6):

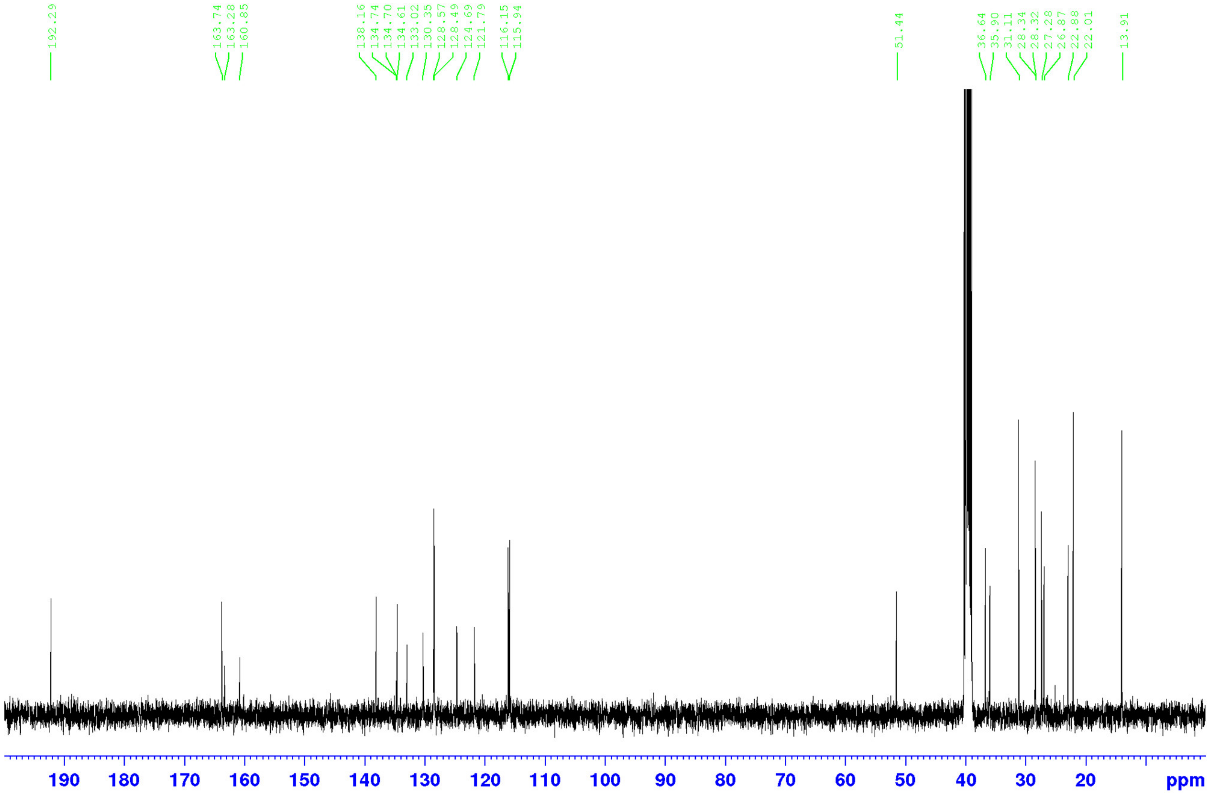

N-(3-Aminopropyl)-2-(4'-chloro-4-(octylsulfonamido)-[1,1'-biphenyl]-3-yl)-2-oxoacetamide hydrochloride (**19c**)

^{1}H NMR (400 MHz, DMSO-d_6):

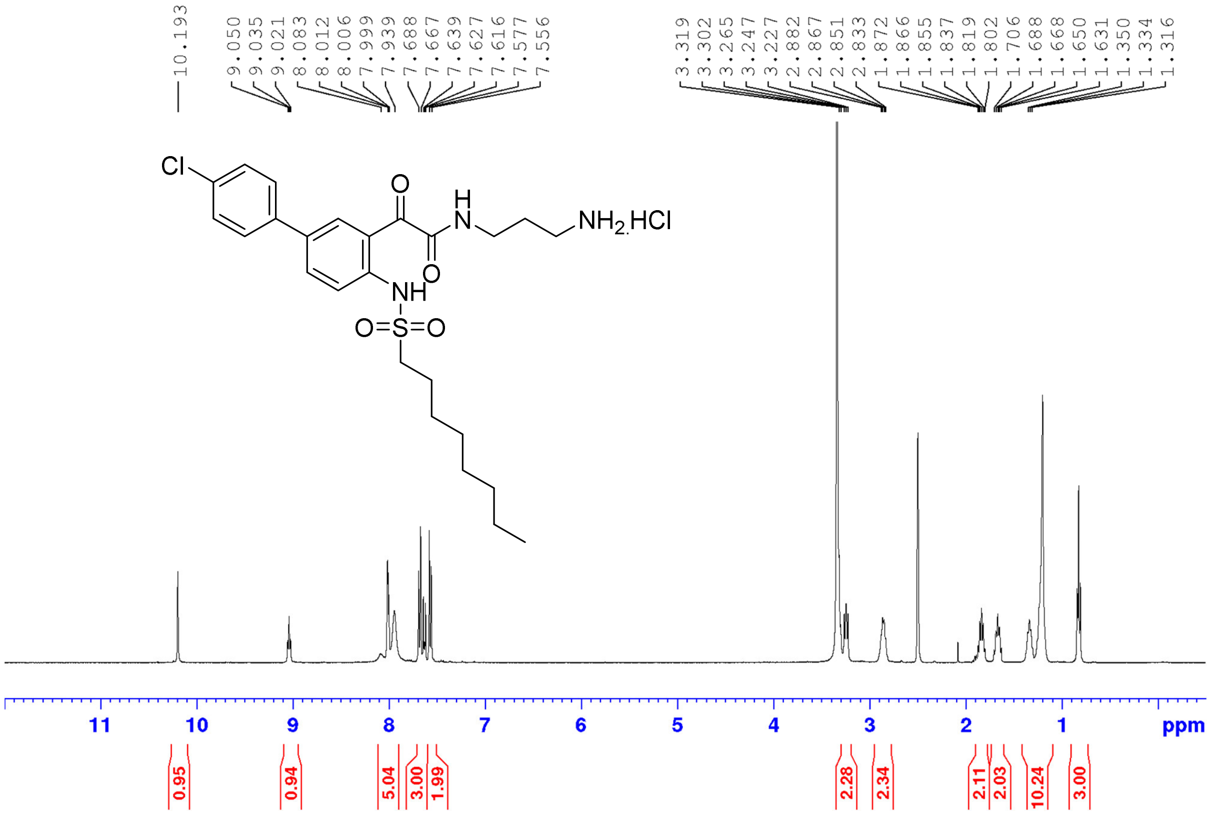

^{13}C NMR (100 MHz, DMSO-d_6):

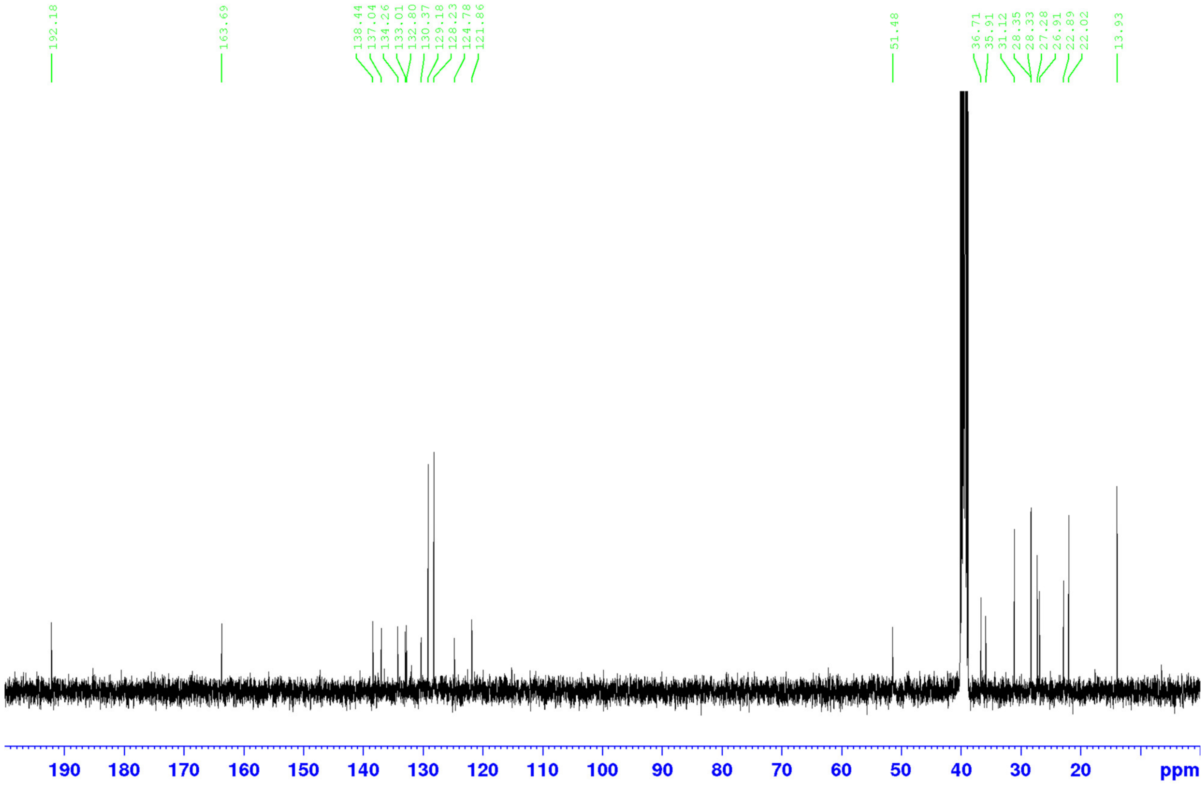

N-(3-Aminopropyl)-2-(5-(naphthalen-2-yl)-2-(octylsulfonamido)phenyl)-2-oxoacetamide hydrochloride (**19d**)

^{1}H NMR (400 MHz, DMSO-d_6):

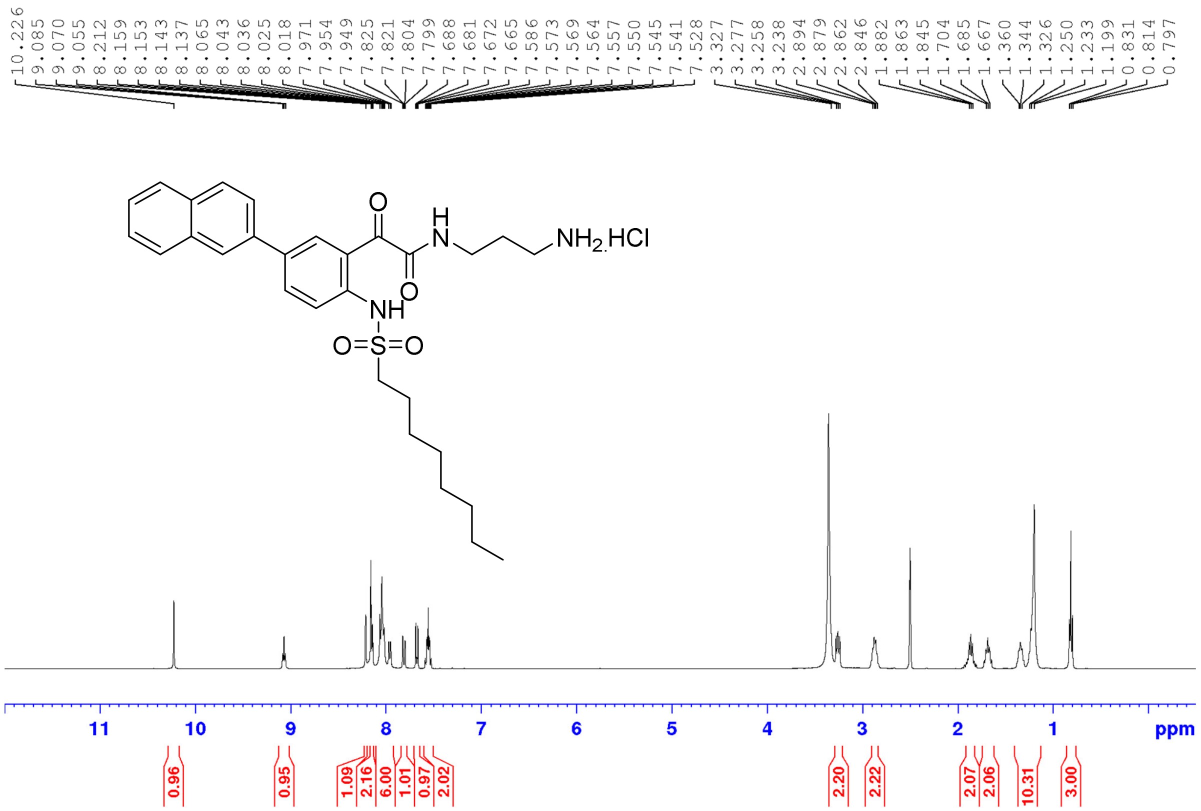

^{13}C NMR (100 MHz, DMSO-d_6):

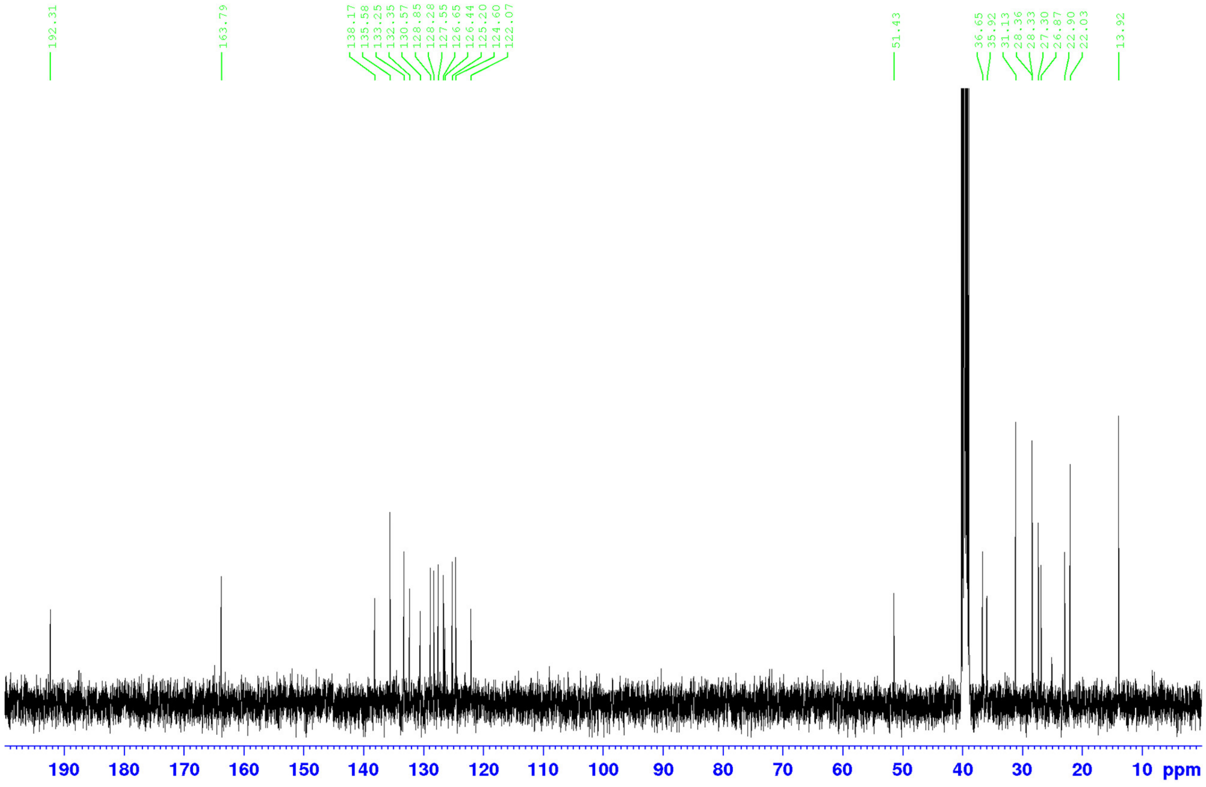

(*E*)-1-tert-Butyl-*N*-(*N*'-((*tert*-butyloxidanyl)carbonyl)-*N*-(3-(2-(4-(octylsulfonamido)-[1,1'-biphenyl]-3-yl)-2-oxoacetamido)propyl)carbamimidoyl)-1-oxidanecarboxamide (**21a**)

^{1}H NMR (400 MHz, $CDCl_3$):

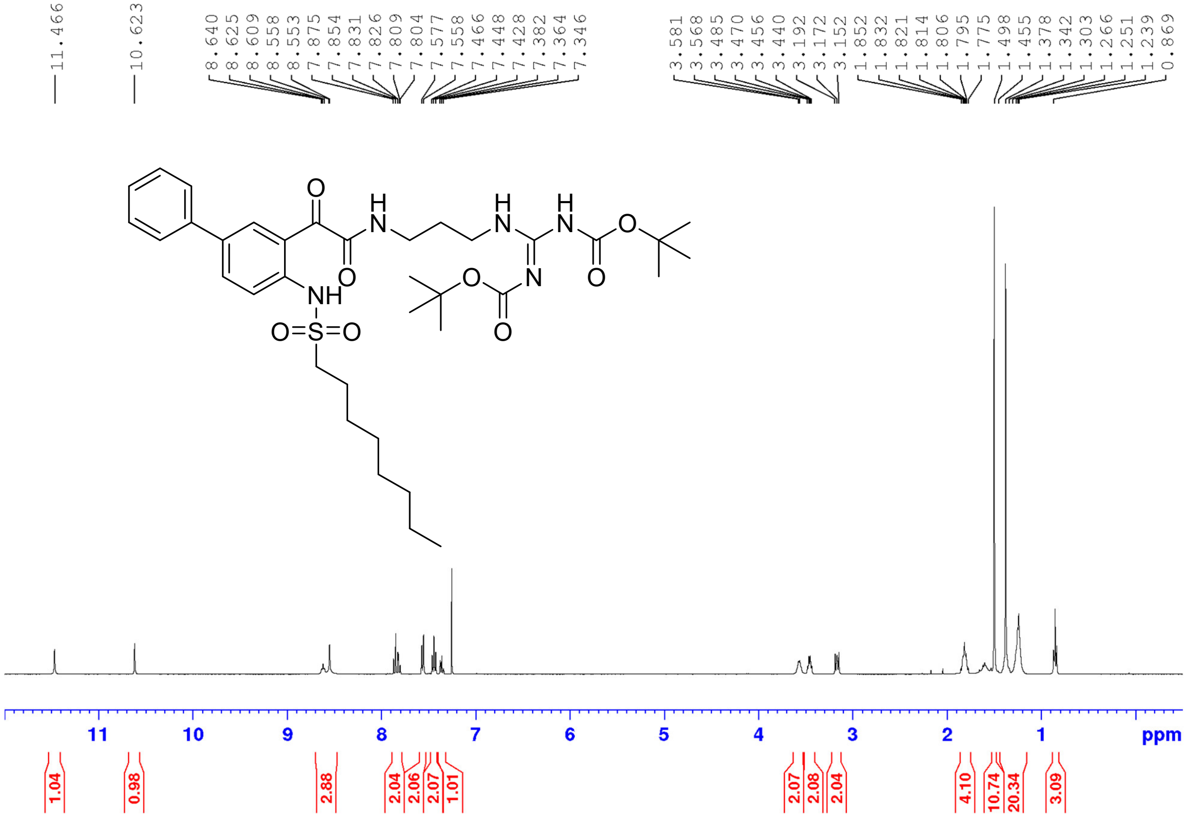

^{13}C NMR (150 MHz, $CDCl_3$):

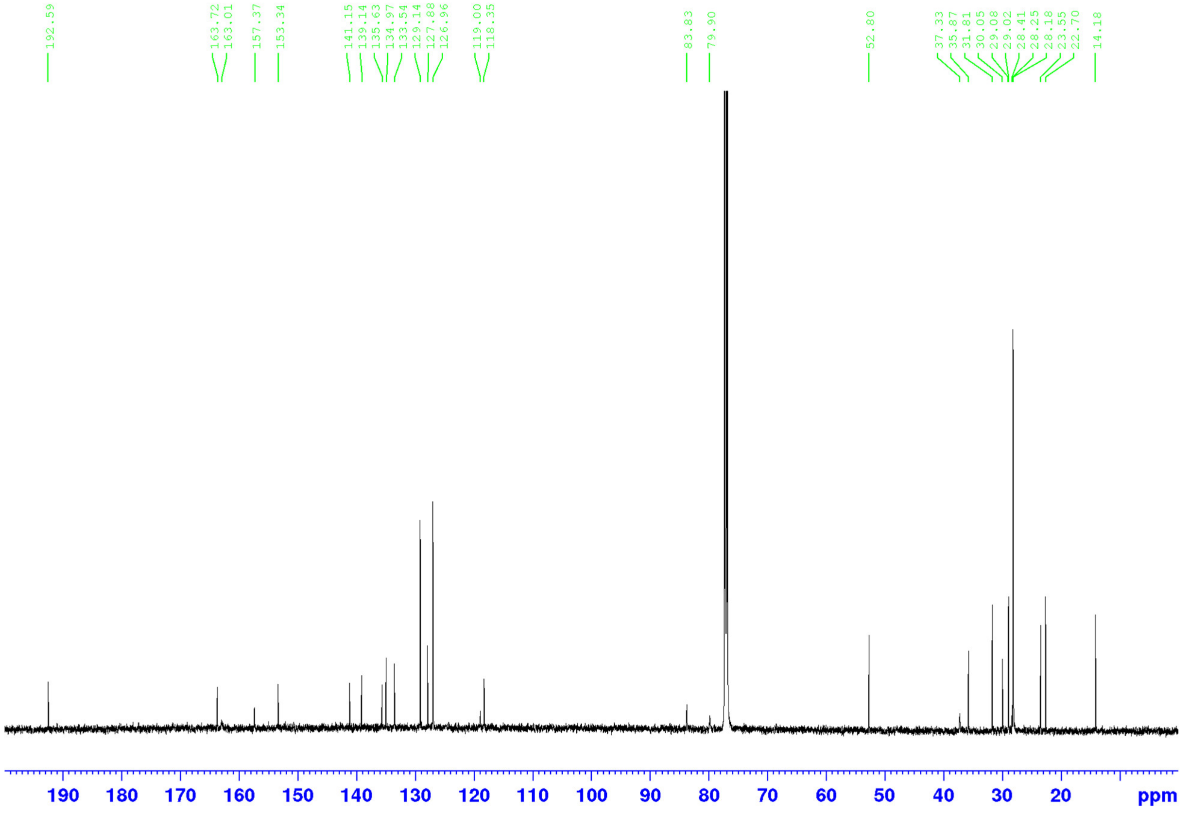

(*E*)-1-*tert*-Butyl-*N*-(*N'*-((*tert*-butyloxidanyl)carbonyl)-*N*-(3-(2-(4'-fluoro-4-(octylsulfonamido)-[1,1'-biphenyl]-3-yl)-2-oxoacetamido)propyl)carbamimidoyl)-1-oxidanecarboxamide (**21b**)

^{1}H NMR (400 MHz, $CDCl_3$):

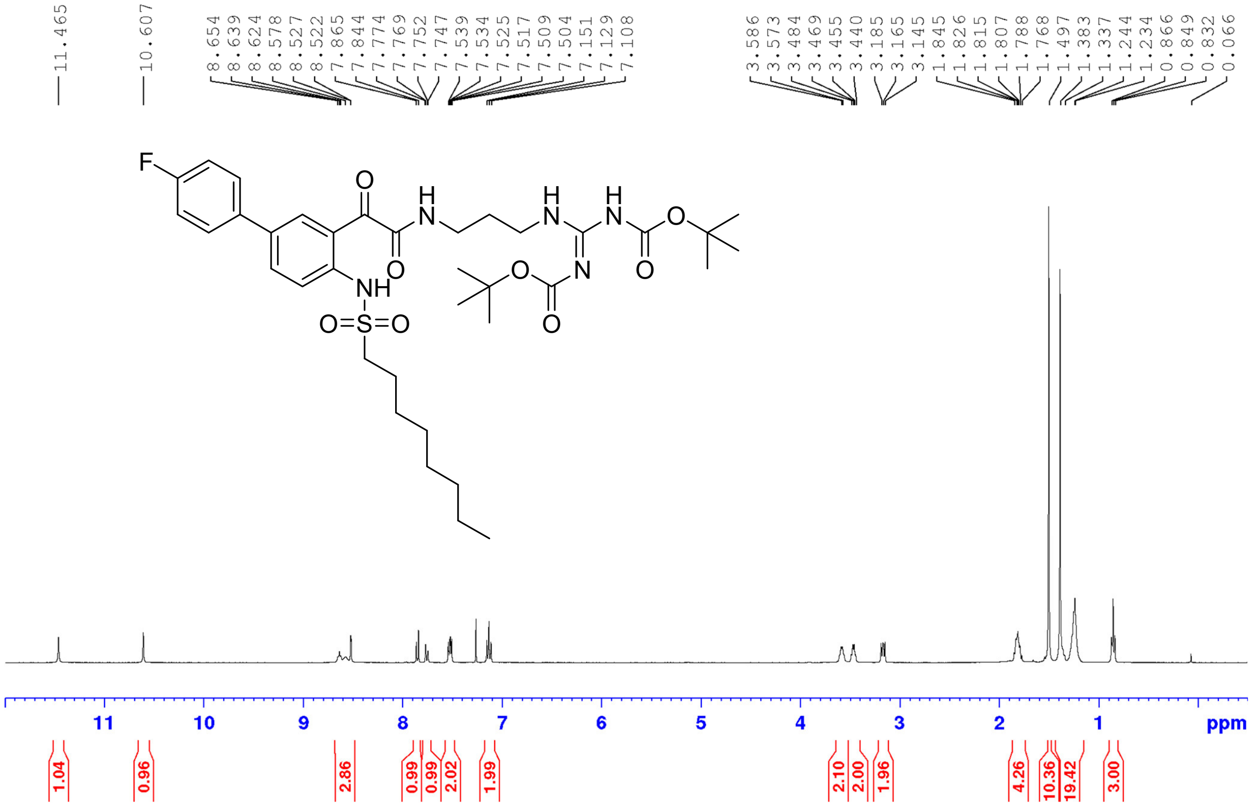

^{13}C NMR (100 MHz, $CDCl_3$):

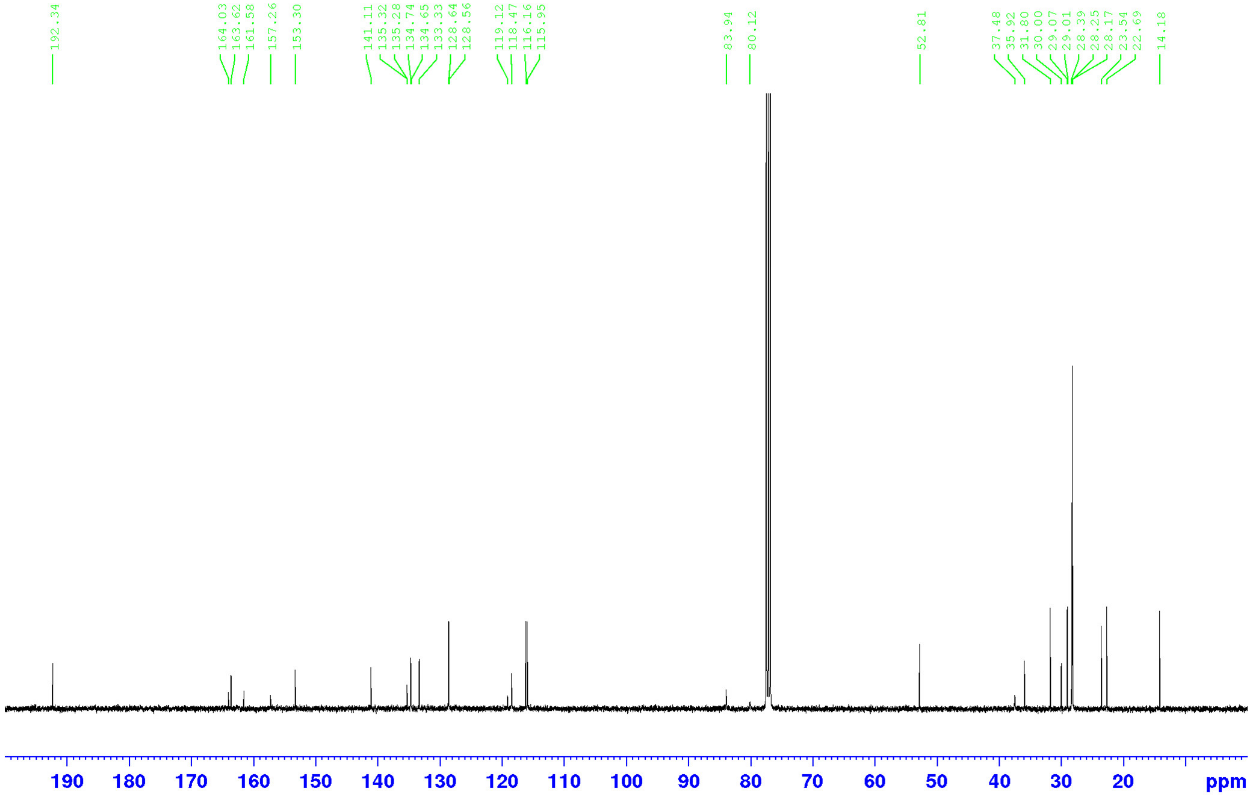

(*E*)-1-*tert*-Butyl-*N*-(*N'*-((*tert*-butyloxidanyl)carbonyl)-*N*-(3-(2-(4'-chloro-4-(octylsulfonamido)-[1,1'-biphenyl]-3-yl)-2-oxoacetamido)propyl)carbamimidoyl)-1-oxidanecarboxamide (**21c**)

^{1}H NMR (400 MHz, $CDCl_3$):

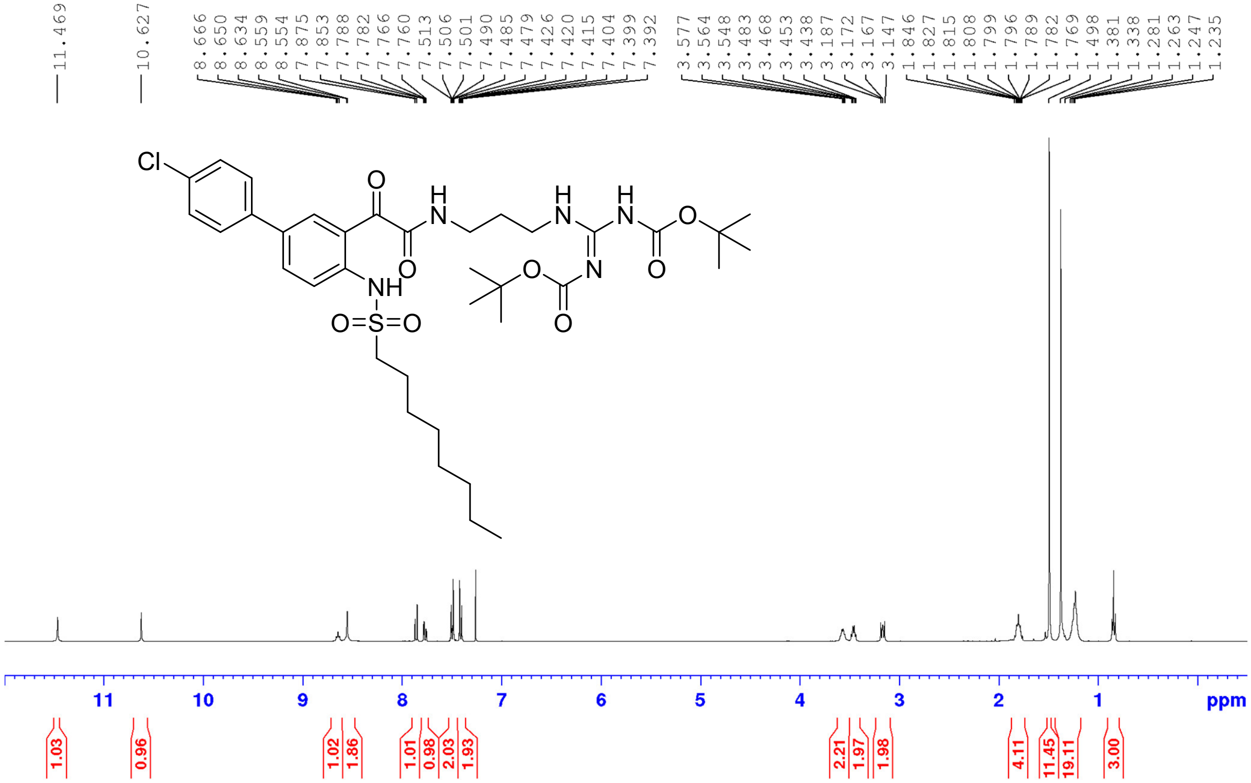

^{13}C NMR (150 MHz, $CDCl_3$):

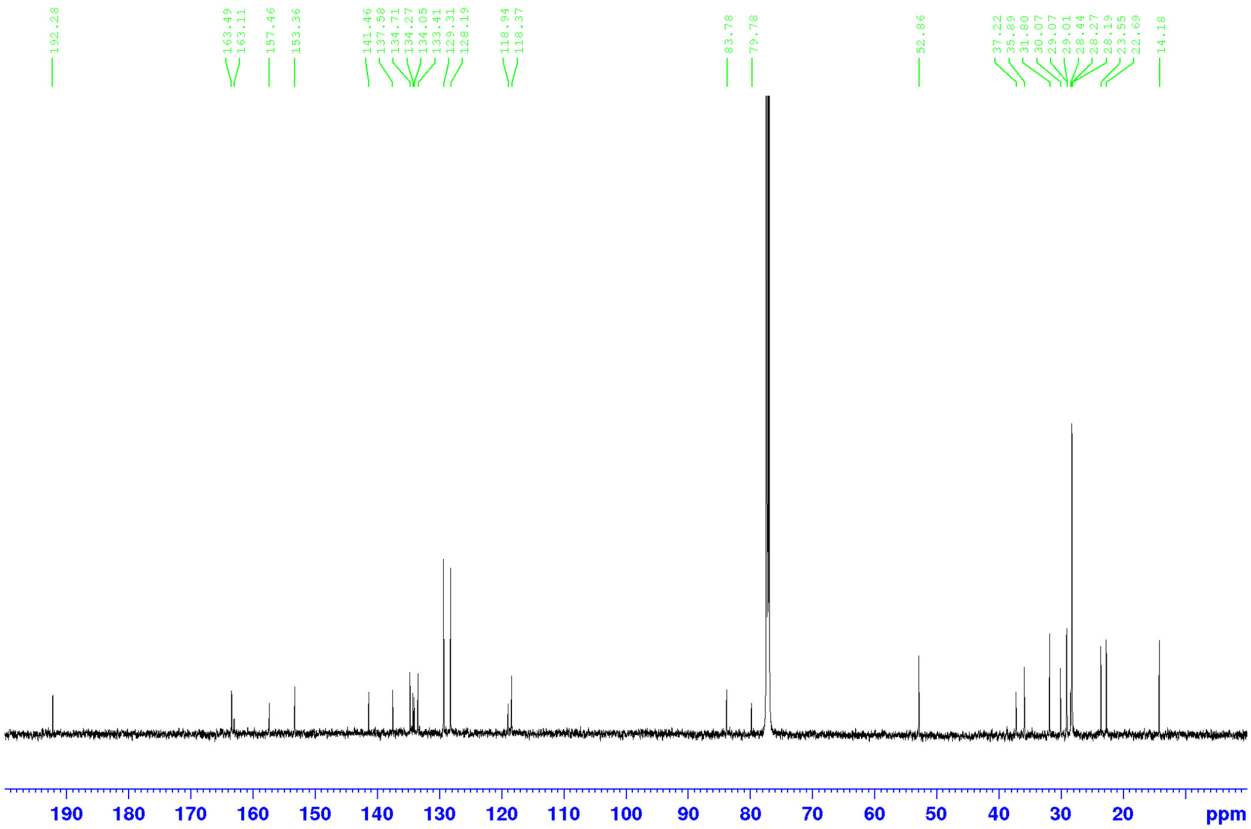

(*E*)-1-*tert*-Butyl-*N*-(*N'*-((*tert*-butyloxidanyl)carbonyl)-*N*-(3-(2-(5-(naphthalen-2-yl)-2-(octylsulfonamido)phenyl)-2-oxoacetamido)propyl)carbamimidoyl)-1-oxidanecarboxamide (**21d**)

^{1}H NMR (400 MHz, $CDCl_3$):

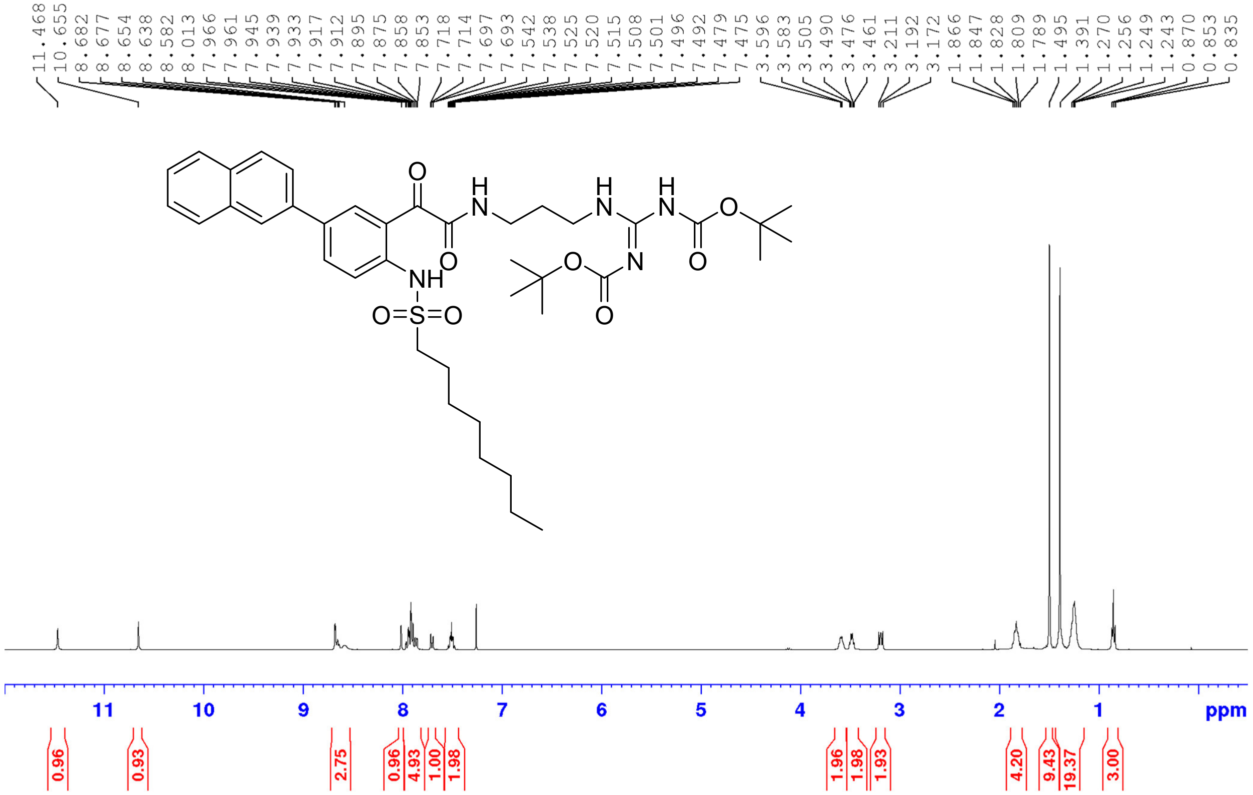

^{13}C NMR (100 MHz, $CDCl_3$):

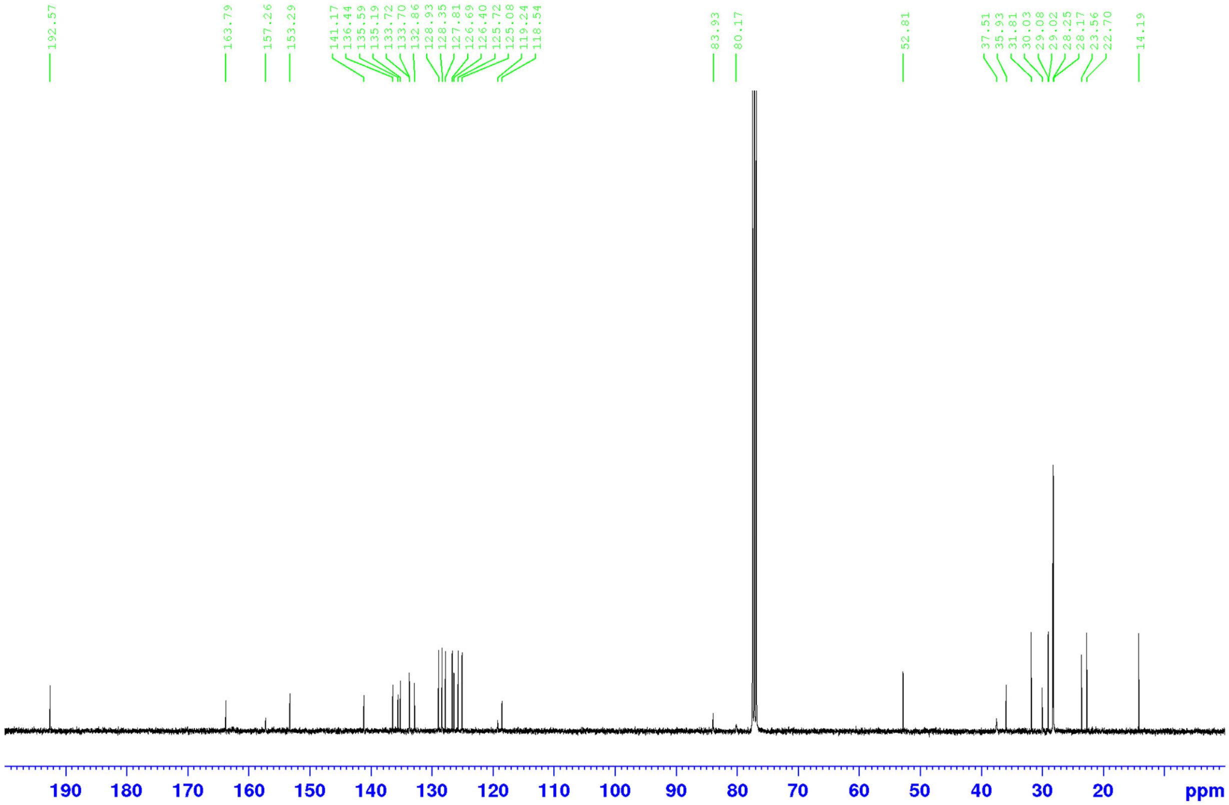

N-(3-Guanidinopropyl)-2-(4-(octylsulfonamido)-[1,1'-biphenyl]-3-yl)-2-oxoacetamide hydrochloride (**22a**)

^{1}H NMR (600 MHz, DMSO-d_6):

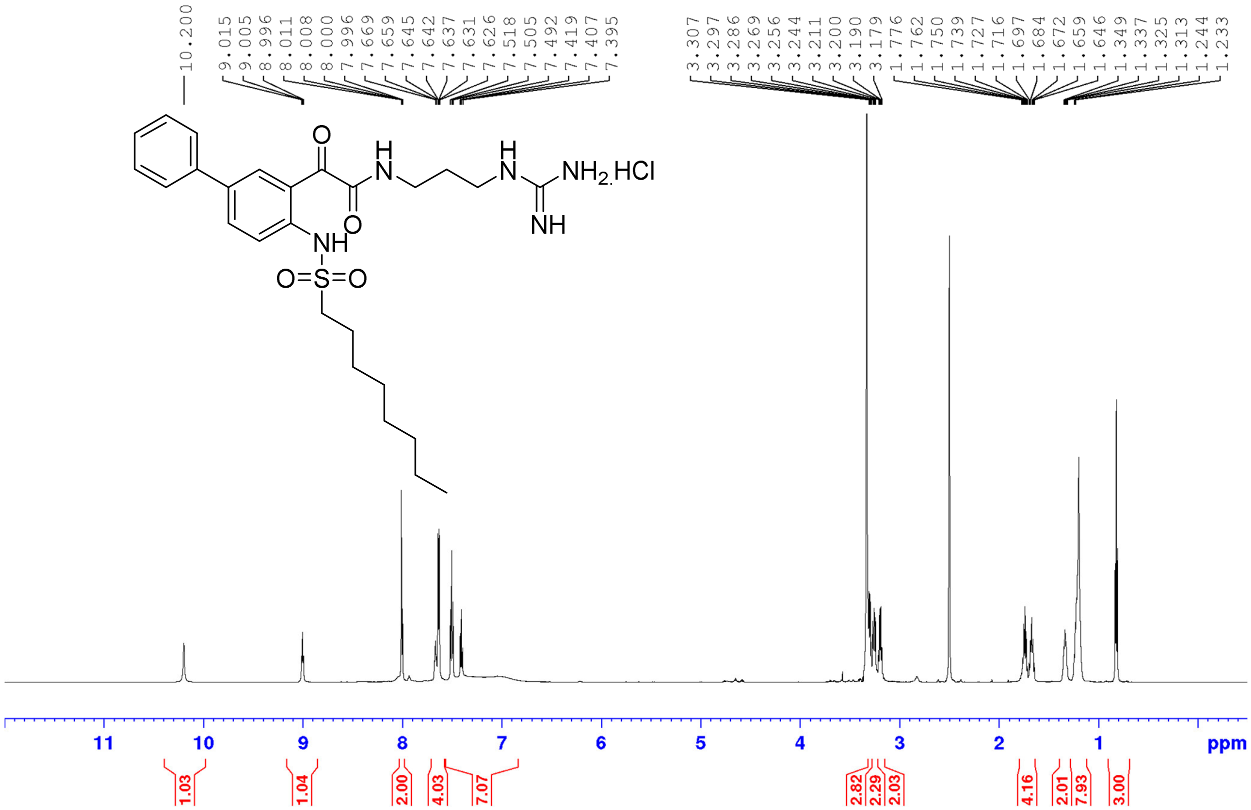

^{13}C NMR (150 MHz, DMSO-d_6):

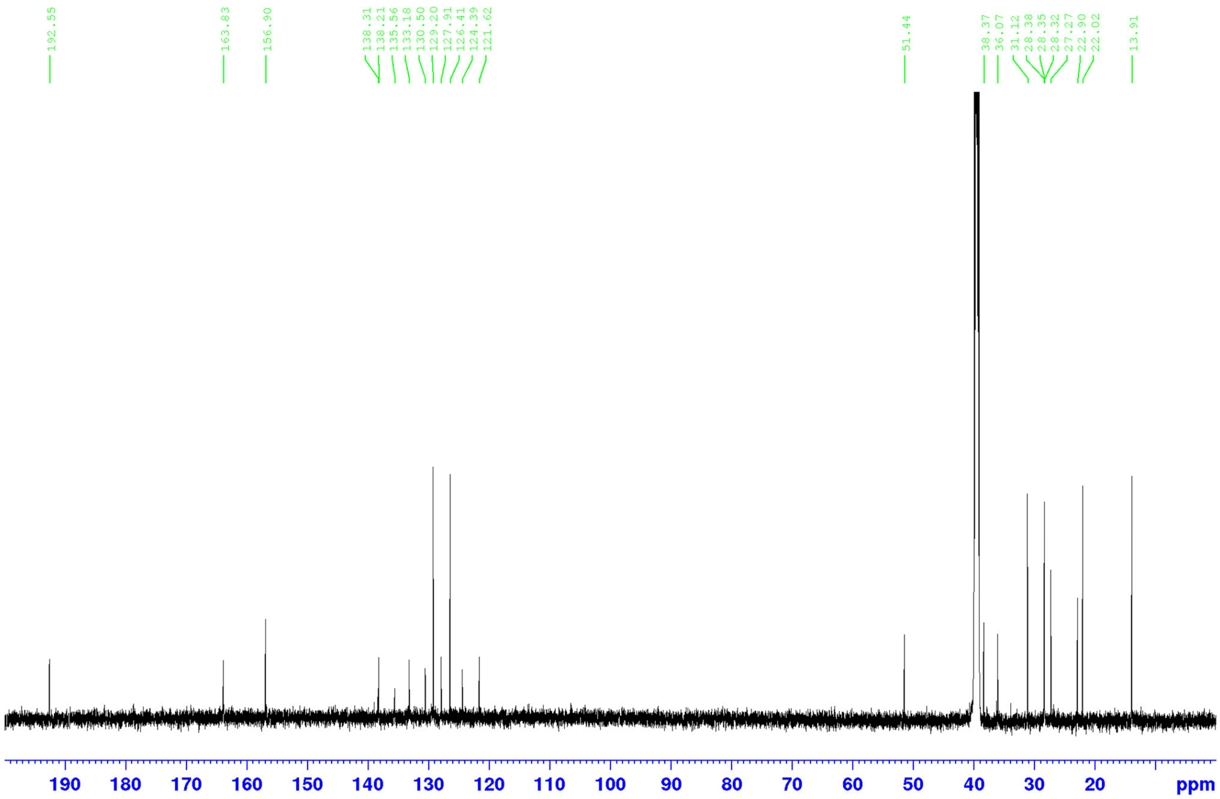

2-(4'-Fluoro-4-(octylsulfonamido)-[1,1'-biphenyl]-3-yl)-*N*-(3-guanidinopropyl)-2-oxoacetamide hydrochloride (**22b**)

^{1}H NMR (600 MHz, DMSO-d_6):

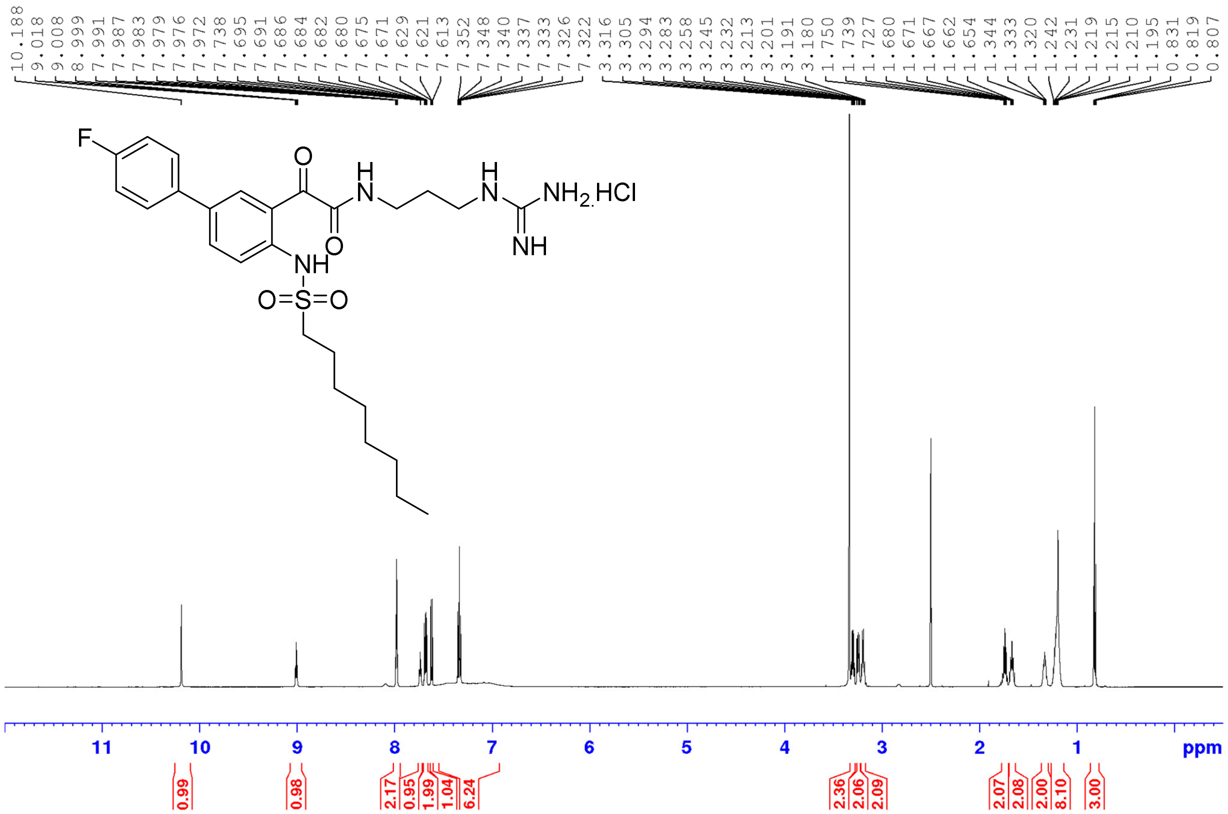

^{13}C NMR (150 MHz, DMSO-d_6):

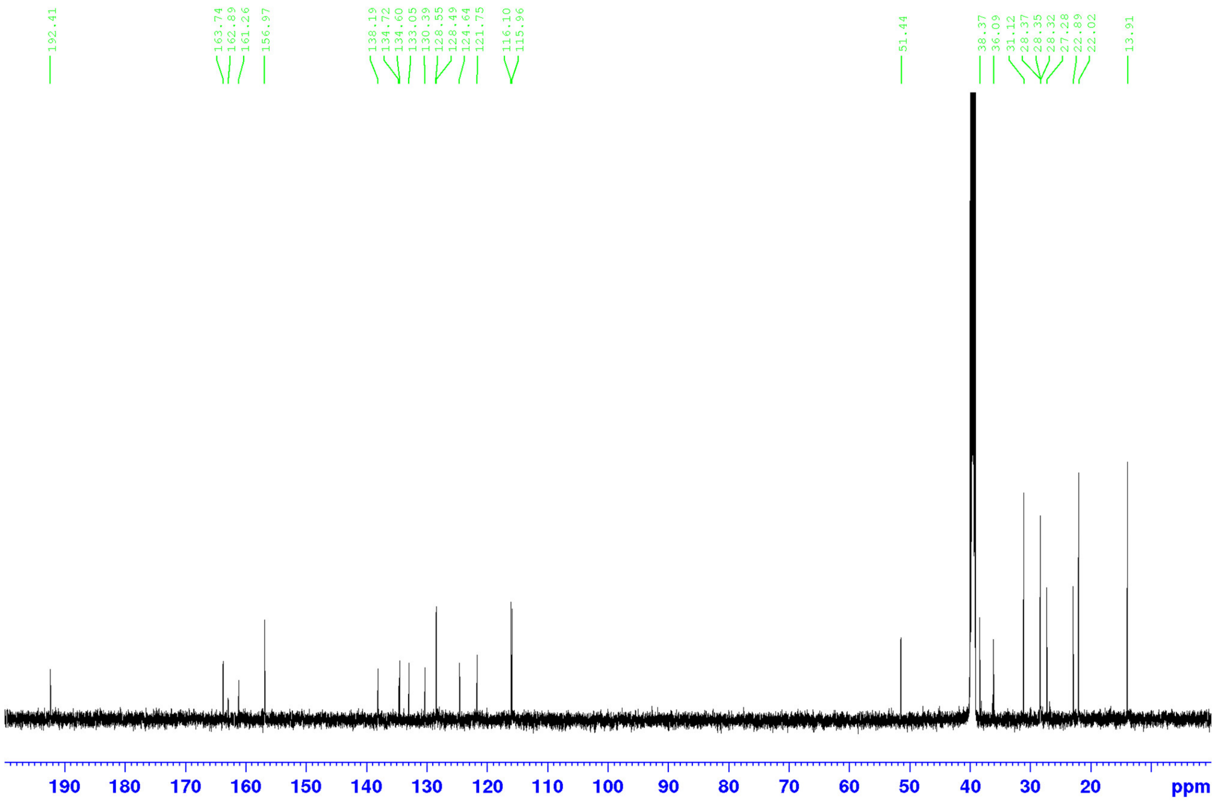

2-(4'-Chloro-4-(octylsulfonamido)-[1,1'-biphenyl]-3-yl)-*N*-(3-guanidinopropyl)-2-oxoacetamide hydrochloride (**22c**)

^{1}H NMR (600 MHz, DMSO-d_6):

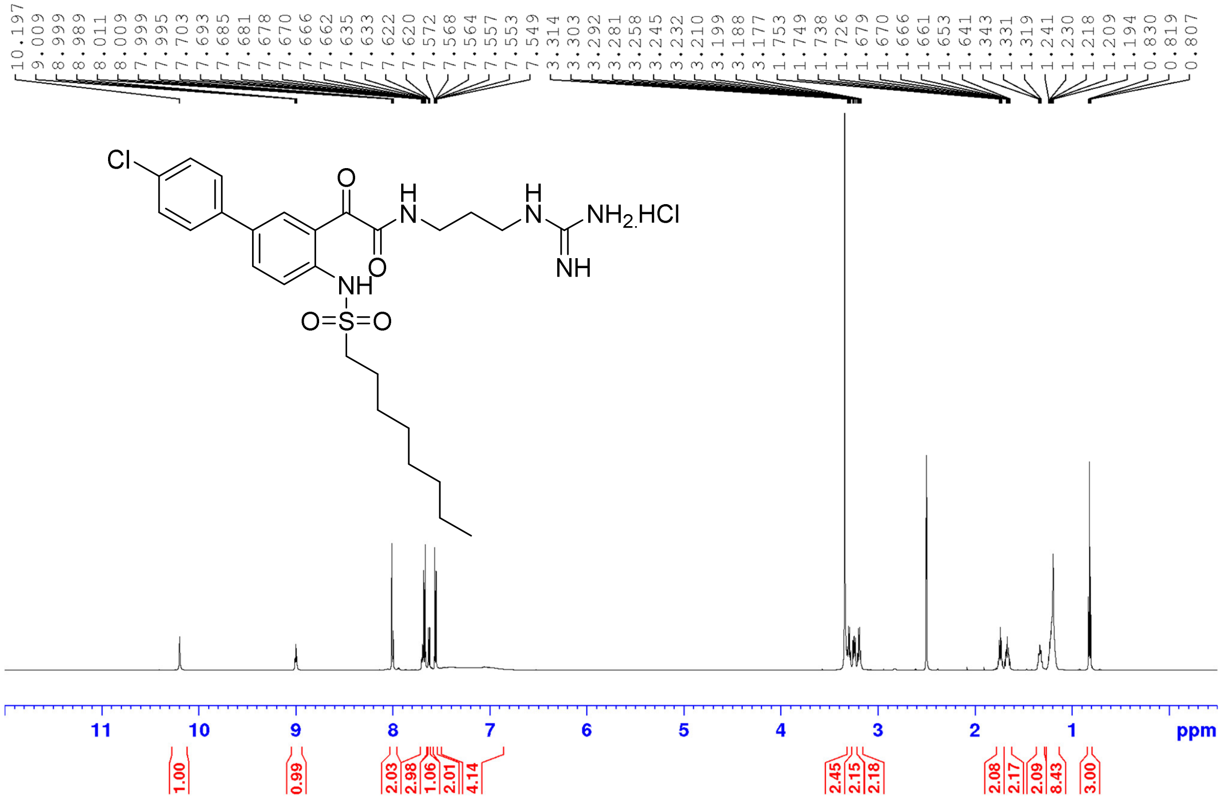

^{13}C NMR (150 MHz, DMSO-d_6):

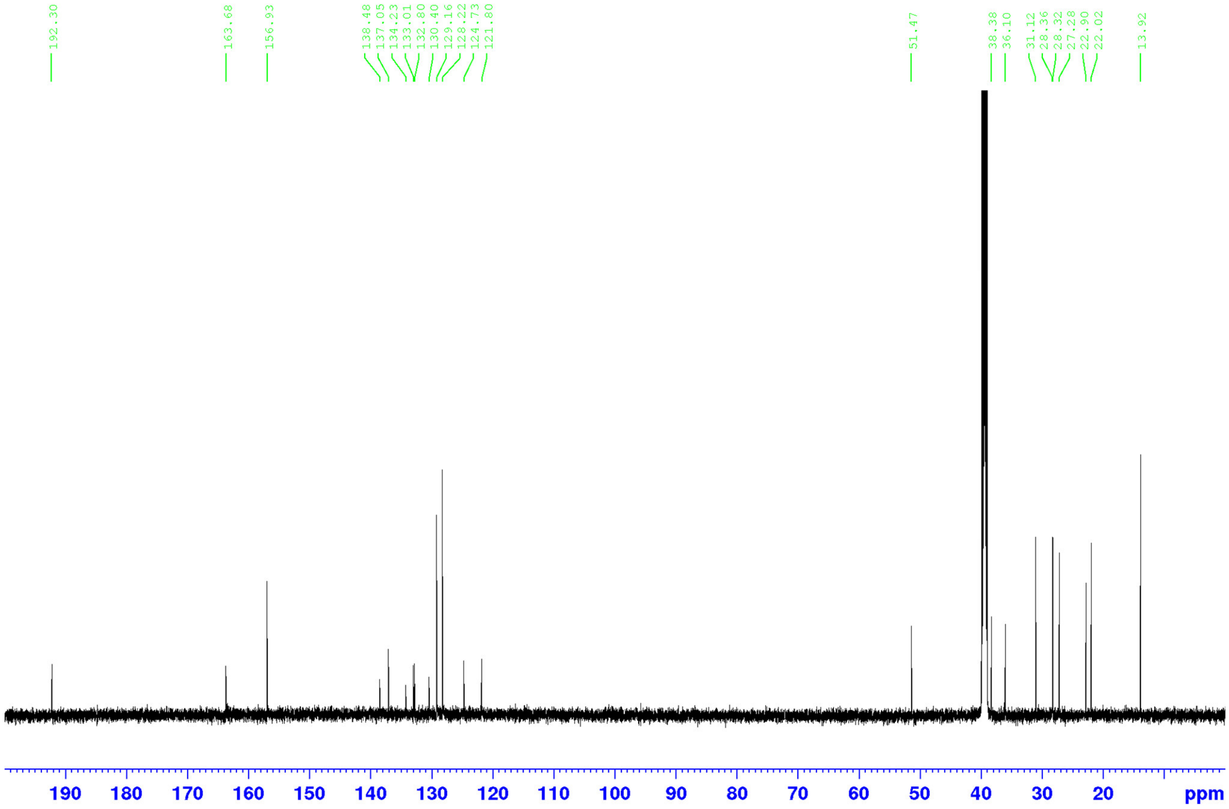

N-(3-Guanidinopropyl)-2-(5-(naphthalen-2-yl)-2-(octylsulfonamido)phenyl)-2-oxoacetamide hydrochloride (**22d**)

^{1}H NMR (600 MHz, DMSO-d_6):

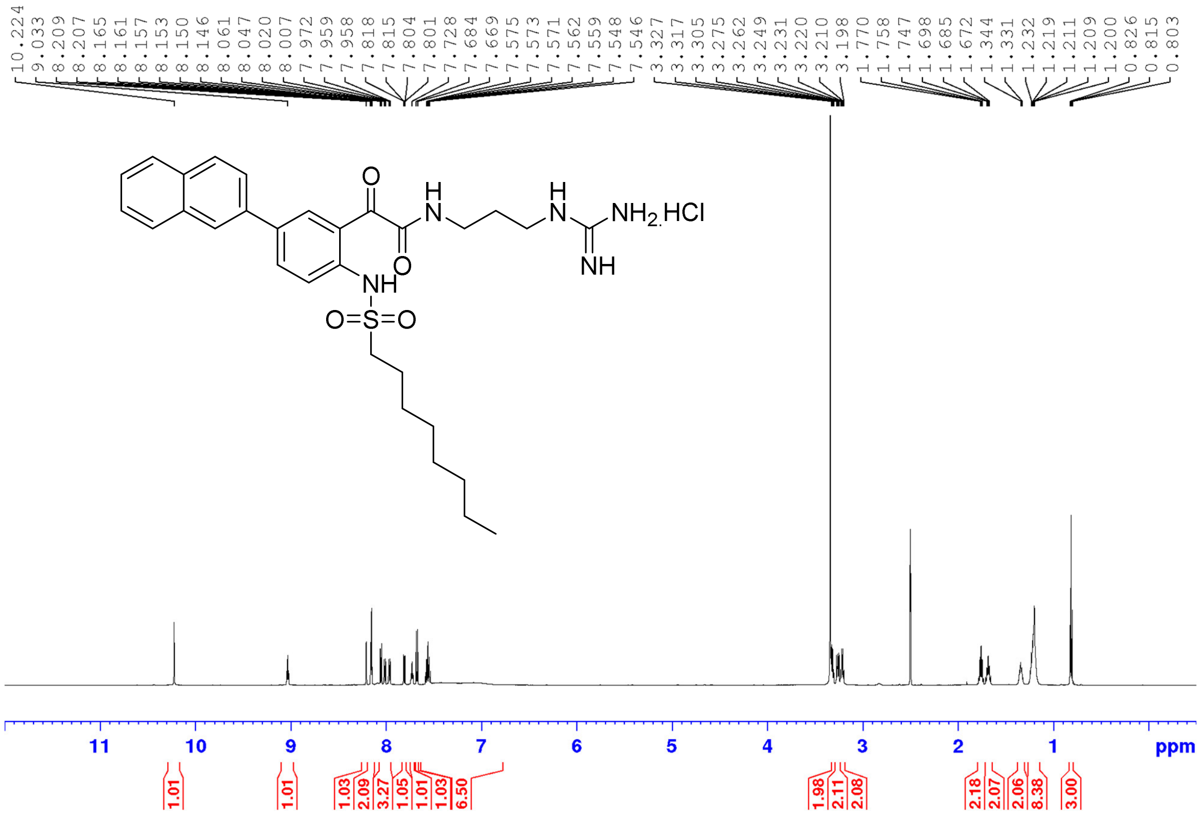

^{13}C NMR (150 MHz, DMSO-d_6):

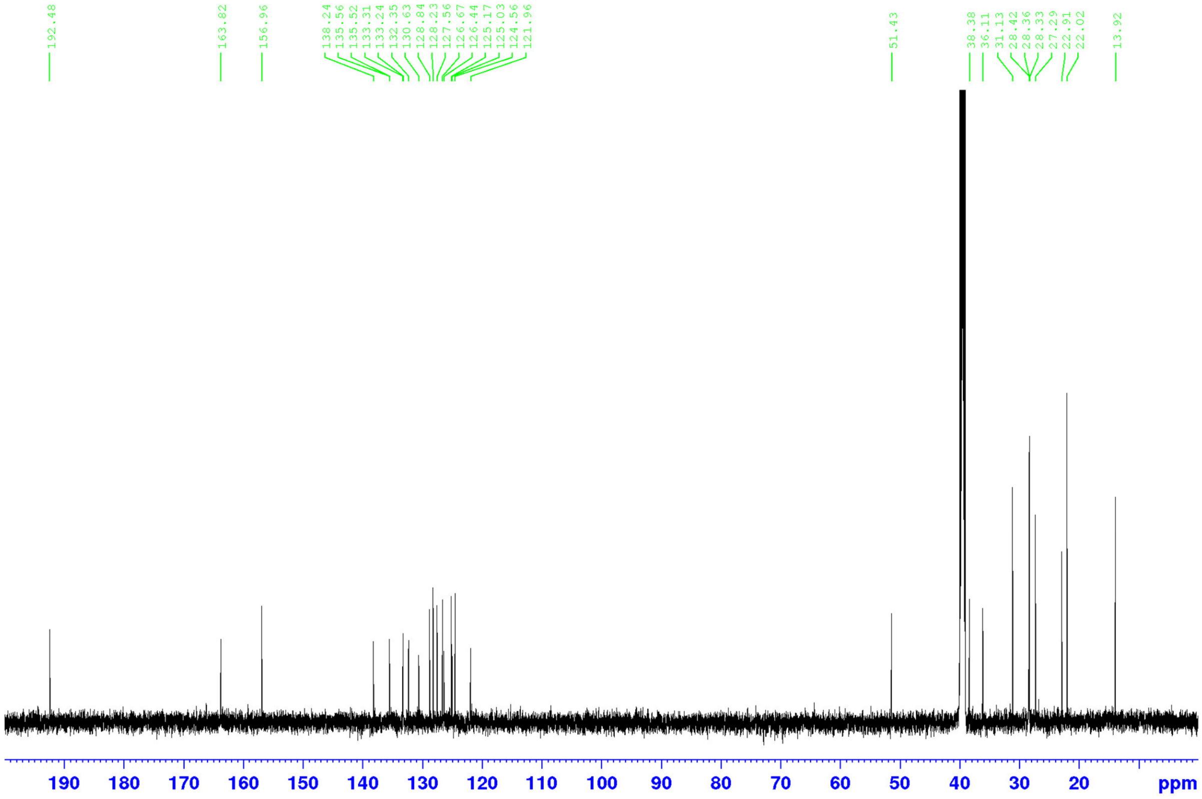

1-(2-Naphthoyl)-5-phenylindoline-2,3-dione (**24**)

^{1}H NMR (600 MHz, DMSO-d_6):

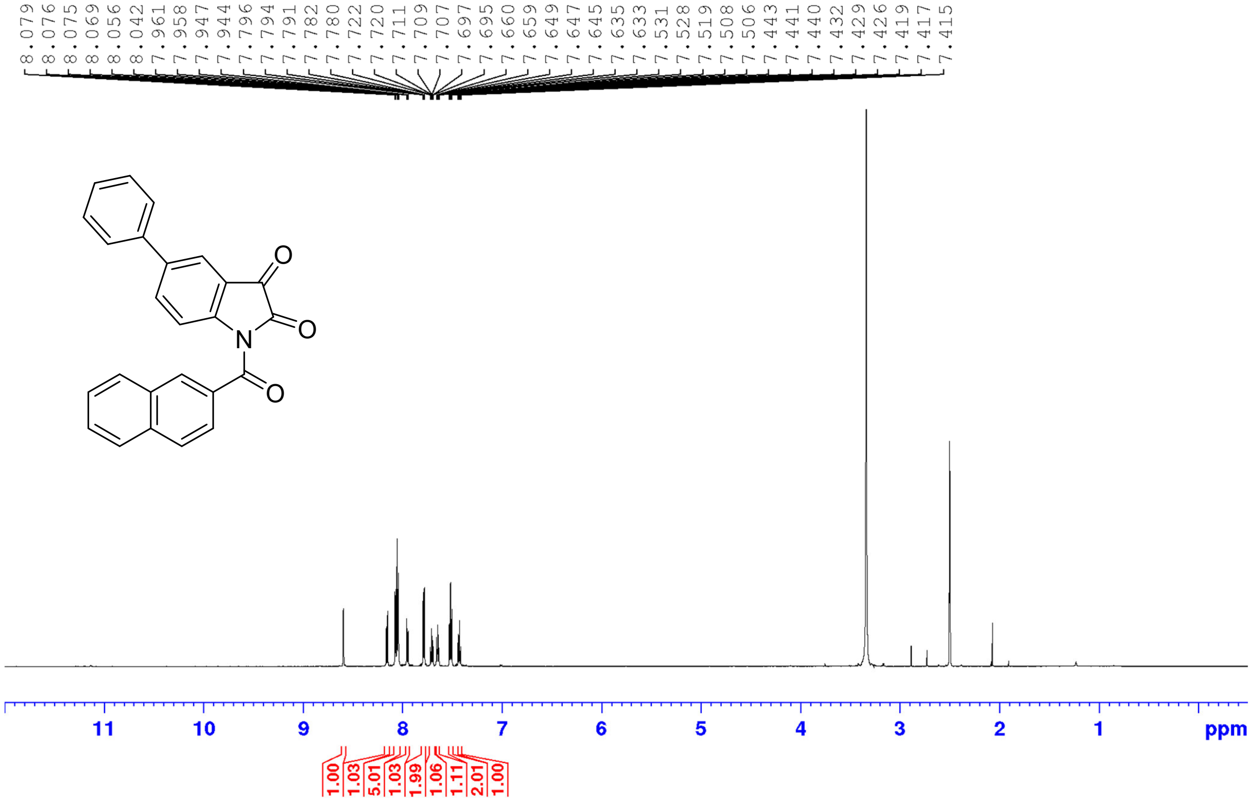

^{13}C NMR (150 MHz, DMSO-d_6):

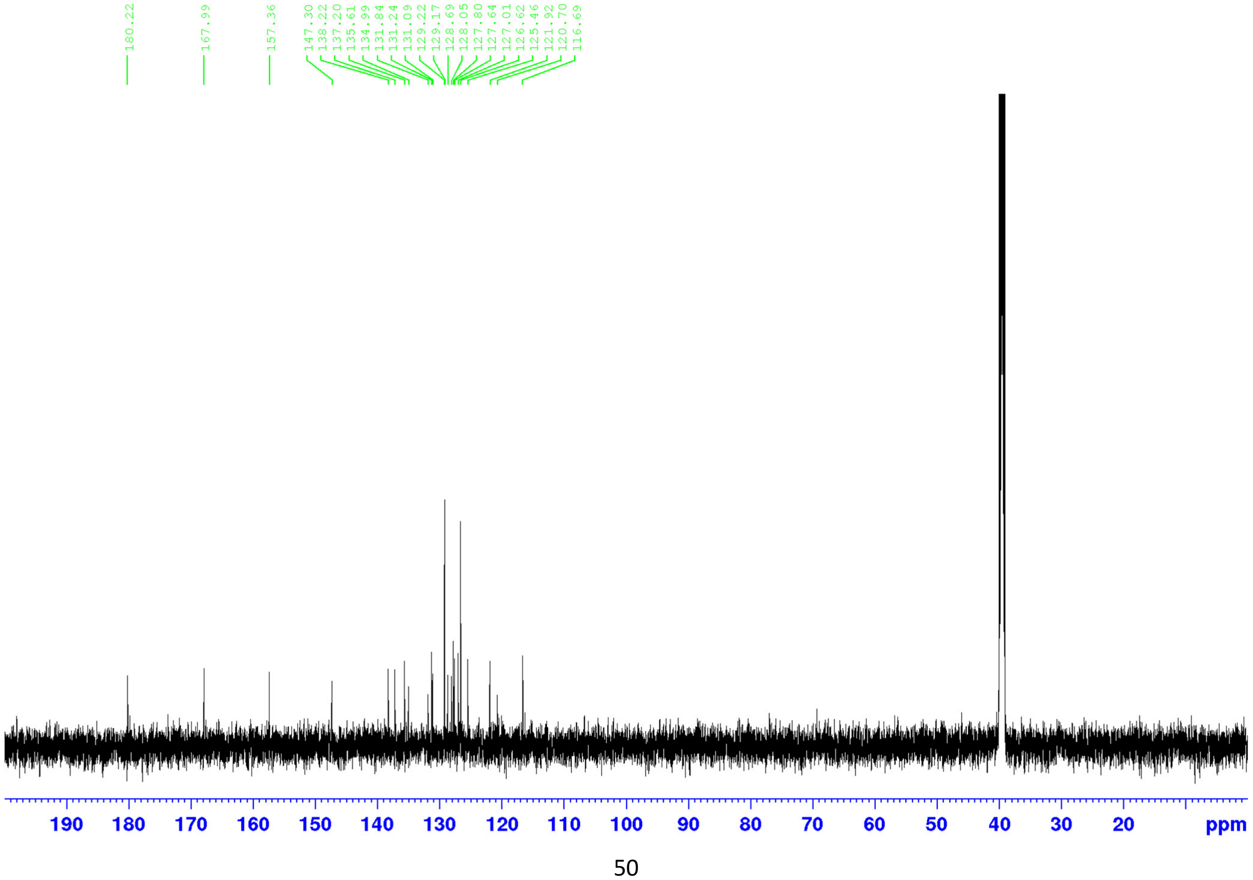

N-(3-(2-((3-(Dimethylamino)propyl)amino)-2-oxoacetyl)-[1,1'-biphenyl]-4-yl)-2-naphthamide (**25**)

^{1}H NMR (600 MHz, $CDCl_3$):

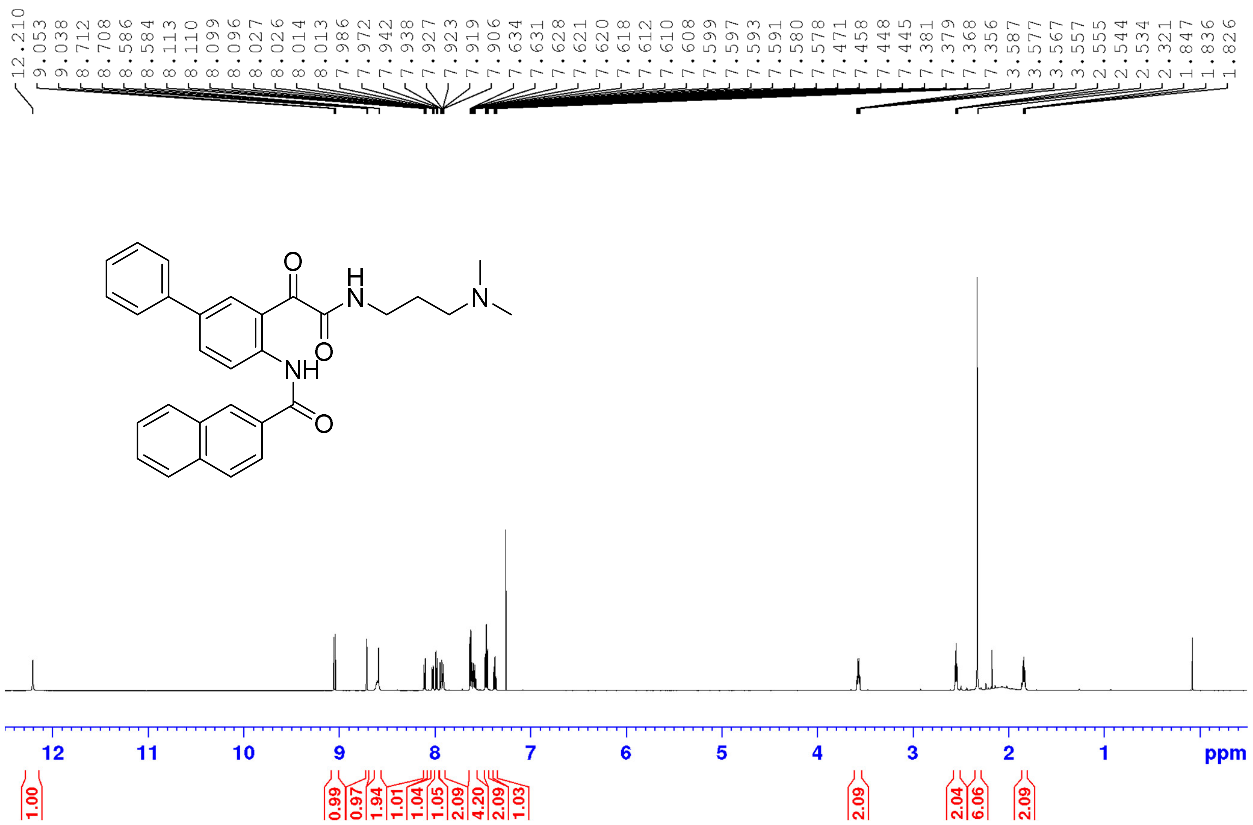

^{13}C NMR (150 MHz, $CDCl_3$):

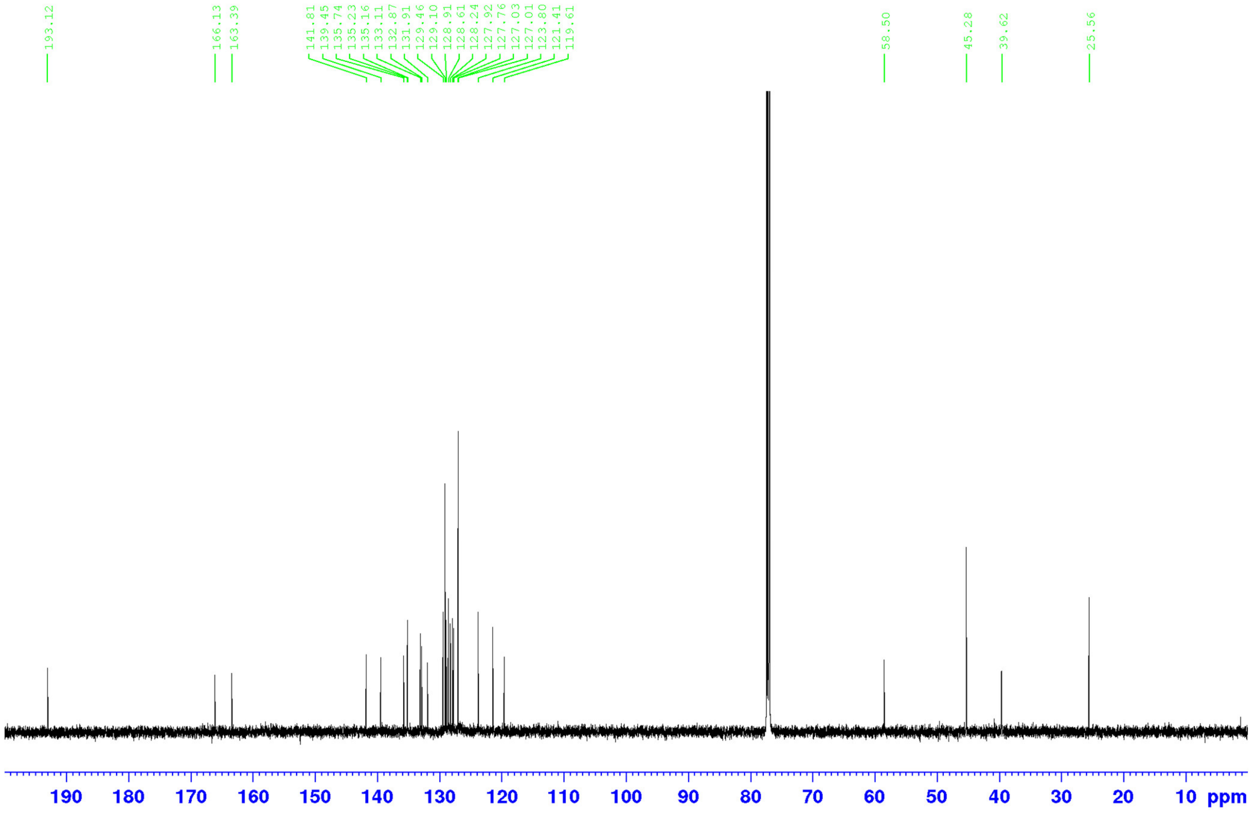

3-(2-(4-(2-Naphthamido)-[1,1'-biphenyl]-3-yl)-2-oxoacetamido)-*N*,*N*-dimethylpropan-1-aminium chloride (**26**)

^{1}H NMR (600 MHz, DMSO-d_6):

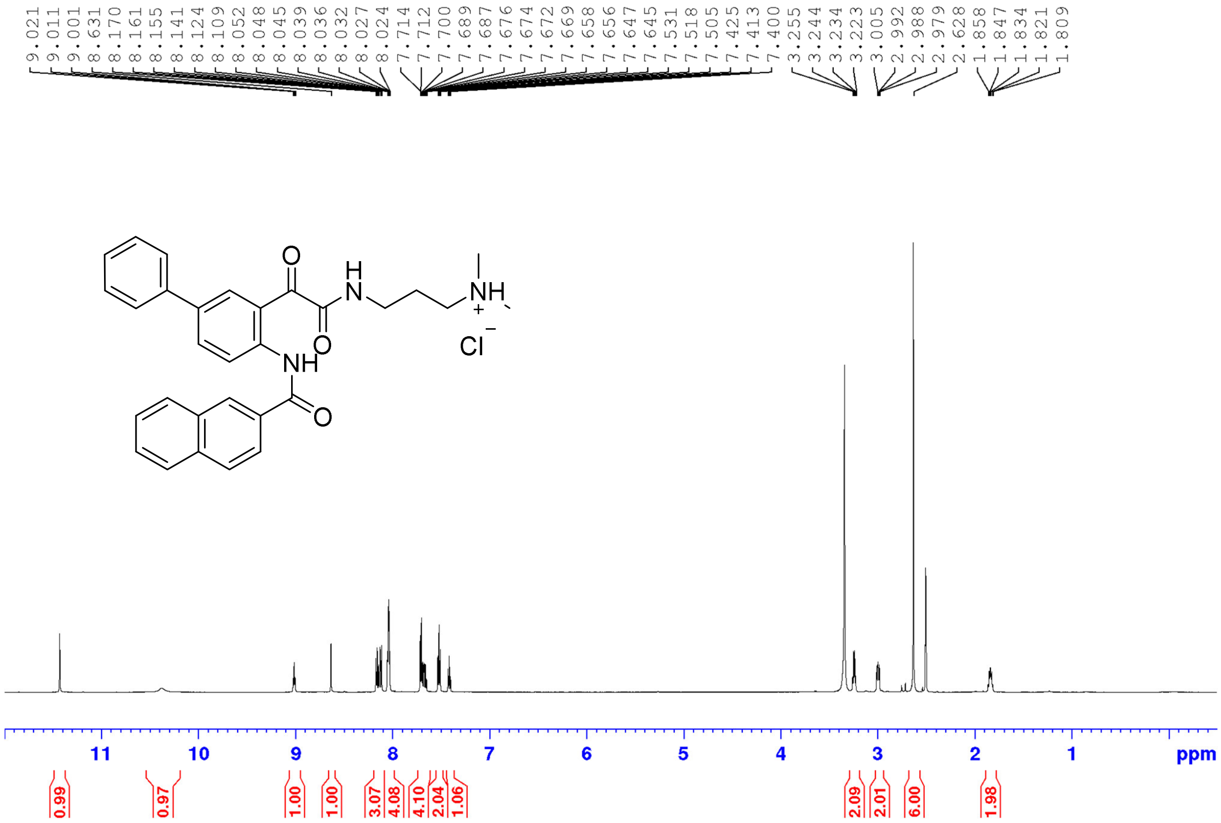

^{13}C NMR (150 MHz, DMSO-d_6):

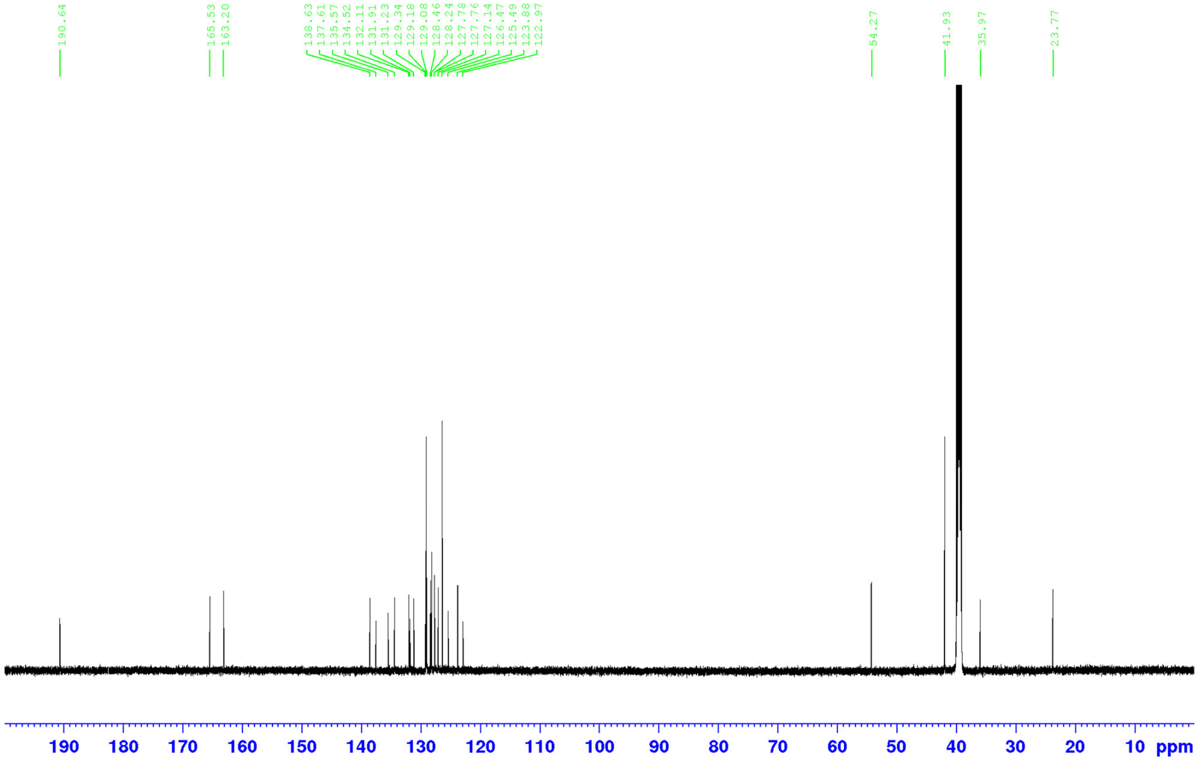

3-(2-(4-(2-Naphthamido)-[1,1'-biphenyl]-3-yl)-2-oxoacetamido)-*N*,*N*,*N*-trimethylpropan-1-aminium iodide (**27**)

^{1}H NMR (600 MHz, DMSO-d_6):

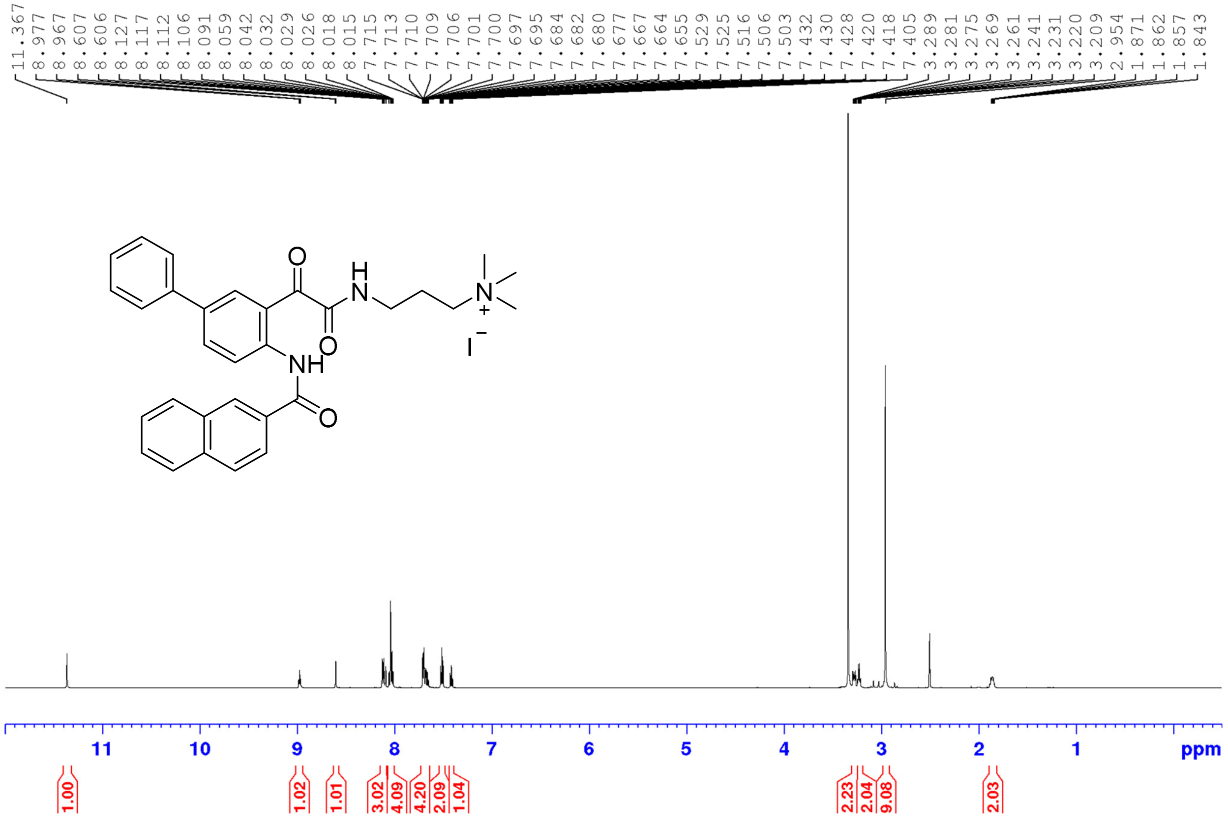

^{13}C NMR (150 MHz, DMSO-d_6):

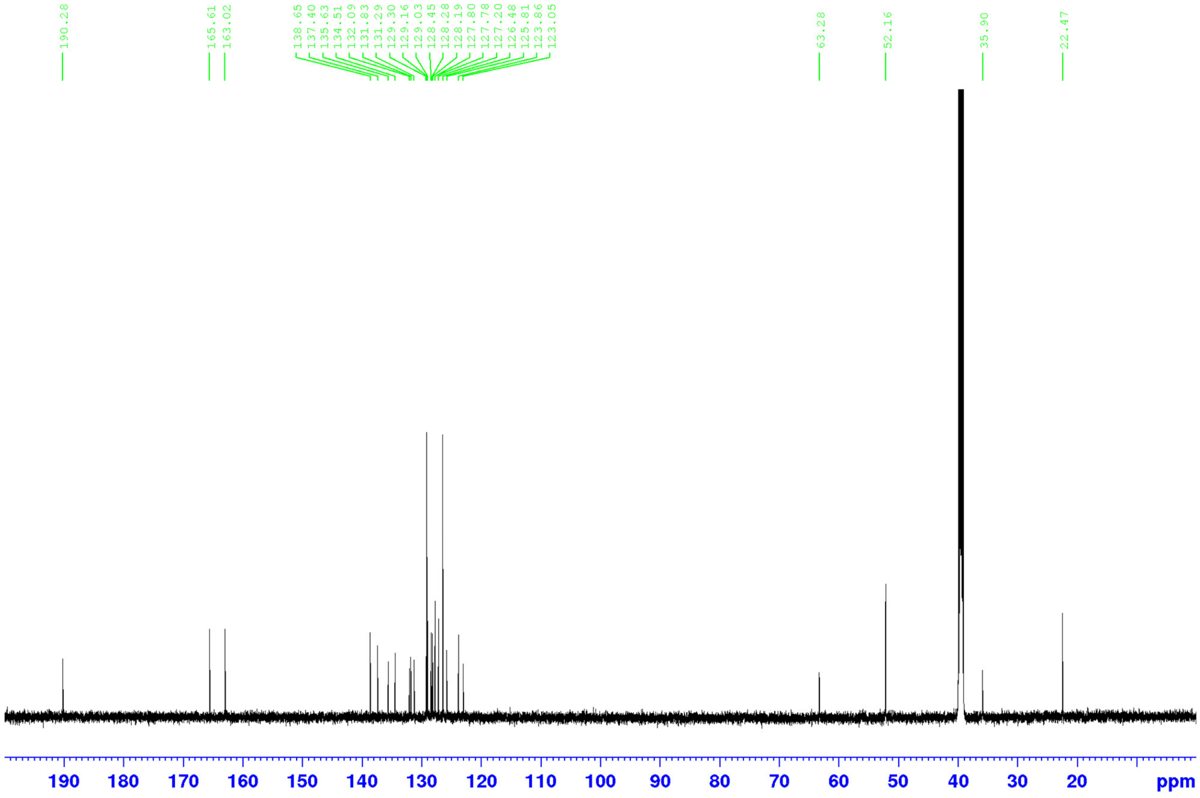

Supplement: Supplementary file 1 [file ijms-21-06789-s001.pdf]
